# A cell-based model system links chromothripsis with hyperploidy

**DOI:** 10.15252/msb.20156505

**Published:** 2015-09-28

**Authors:** Balca R Mardin, Alexandros P Drainas, Sebastian M Waszak, Joachim Weischenfeldt, Mayumi Isokane, Adrian M Stütz, Benjamin Raeder, Theocharis Efthymiopoulos, Christopher Buccitelli, Maia Segura-Wang, Paul Northcott, Stefan M Pfister, Peter Lichter, Jan Ellenberg, Jan O Korbel

**Affiliations:** 1 European Molecular Biology Laboratory, Genome Biology UnitHeidelberg, Germany; 2 European Molecular Biology Laboratory, Cell Biology and Biophysics UnitHeidelberg, Germany; 3 Division of Pediatric Neurooncology, German Cancer Research Center (DKFZ)Heidelberg, Germany; 4 Division of Molecular Genetics, German Cancer Research Center (DKFZ)Heidelberg, Germany

**Keywords:** chromothripsis, hyperploidy, DNA rearrangements, telomere damage, transformation

## Abstract

A remarkable observation emerging from recent cancer genome analyses is the identification of chromothripsis as a one-off genomic catastrophe, resulting in massive somatic DNA structural rearrangements (SRs). Largely due to lack of suitable model systems, the mechanistic basis of chromothripsis has remained elusive. We developed an integrative method termed “complex alterations after selection and transformation (CAST),” enabling efficient *in vitro* generation of complex DNA rearrangements including chromothripsis, using cell perturbations coupled with a strong selection barrier followed by massively parallel sequencing. We employed this methodology to characterize catastrophic SR formation processes, their temporal sequence, and their impact on gene expression and cell division. Our *in vitro* system uncovered a propensity of chromothripsis to occur in cells with damaged telomeres, and in particular in hyperploid cells. Analysis of primary medulloblastoma cancer genomes verified the link between hyperploidy and chromothripsis *in vivo*. CAST provides the foundation for mechanistic dissection of complex DNA rearrangement processes.

## Introduction

During tumorigenesis, a single genetic alteration (or “hit”) is generally thought to be insufficient for a cell to develop into cancer. Instead, several gradually acquired mutations or SRs occurring in a stepwise process are required, mediating incremental tumor development (Knudson, 1971; Stratton *et al*, 2009). However, cancer genomes can evolve in rapid bursts. Recent cancer genomic surveys presented compelling evidence for a process involving massive *de novo* SR formation in a one-step catastrophic genomic event denoted chromothripsis (*chromo* for chromosome; *thripsis* for shattering into pieces) (Stephens *et al*, 2011; Korbel & Campbell, 2013). Chromothripsis can massively rearrange chromosomal arms or even entire chromosomes, leading to reshuffled genomic regions that frequently harbor clusters of deleted segments (Stephens *et al*, 2011; Korbel & Campbell, 2013) and occasionally clustered amplifications, in case chromothripsis is followed by double minute chromosome formation (Rausch *et al*, 2012a). This catastrophic SR process is observed in 2–3% of cancers (Stephens *et al*, 2011), linked with poor disease outcome (Kloosterman *et al*, 2014), and has been reported to exhibit increased rates in particular cancer types, including bone cancer, esophageal adenocarcinoma, glioblastoma, and Sonic-hedgehog-subtype medulloblastoma (Stephens *et al*, 2011; Rausch *et al*, 2012a; Kloosterman *et al*, 2014; Nones *et al*, 2014). Chromothripsis is also observed in the context of congenital disorders (Kloosterman *et al*, 2011; Liu *et al*, 2011) and can exhibit a curative effect on genetic disease (McDermott *et al*, 2015).

Following chromosome shattering mediated by chromothripsis, the resulting fragments are lost or become rejoined into derivative chromosomes, presumably through unfaithful DNA repair (Kloosterman *et al*, 2011; Stephens *et al*, 2011; Rausch *et al*, 2012a). Although cellular catastrophes arising during key stages of the cell cycle are suggested as a potential trigger (Stephens *et al*, 2011; Korbel & Campbell, 2013), few studies focused on the mechanistic origins and consequences of chromothripsis, presumably due to the lack of suitable experimental model systems. While genomic analysis of primary tumors or cancer cell lines can provide valuable snapshots long after chromothripsis has taken place (Kloosterman *et al*, 2011; Stephens *et al*, 2011; Rausch *et al*, 2012a), their utility for studying the initiating molecular process(es) is more limited.

In this study, we describe the approach CAST (complex alterations after selection and transformation) for studying chromothripsis and other complex SR formation processes *in vitro*, a methodology enabling reproducible generation of chromothripsis in a genetically stable cell line. We employ CAST in a proof-of-principle study, provide evidence for association of telomere stability as well as of hyperploidy with chromothripsis, and investigate the functional consequences of this catastrophic DNA rearrangement process.

## Results

### Development of the CAST approach

**Figure 1:**
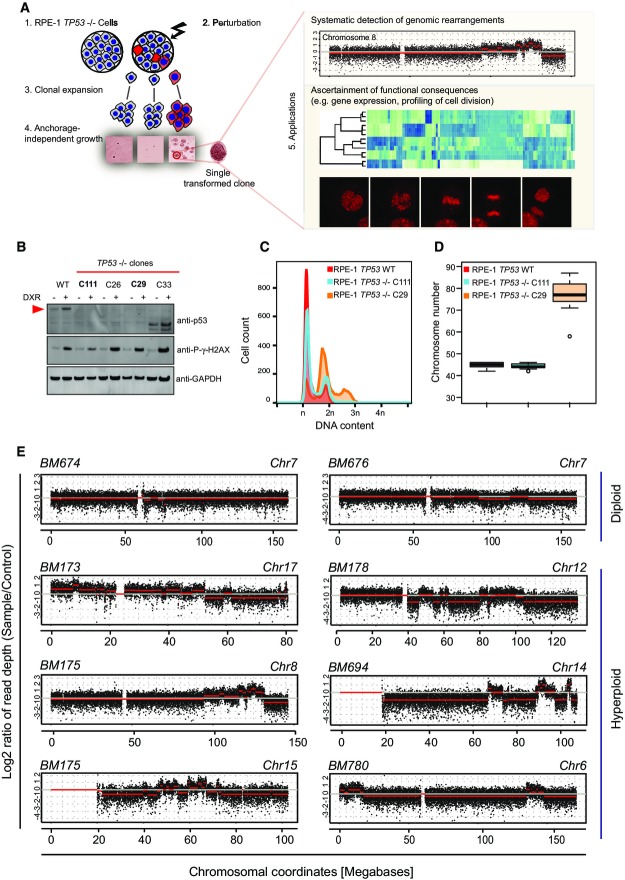
Methodological overview Flowchart depicting the complex alterations after selection and transformation (CAST) approach. After generating p53-deficient RPE-1 cell lines, these cells are challenged by perturbation. Following single cell sorting and clonal expansion on microtiter plates, surviving clones are subjected to soft agar assay to test for anchorage-independent growth (i.e., transformation) as a strong selective barrier. Transformed, single cell-derived colonies are then expanded and systematically analyzed by WGS to identify copy number alterations and SRs. The flexibility of CAST additionally allows ascertainment of functional consequences, for example, by RNA-Seq or live cell imaging.

The approach CAST for studying complex DNA rearrangement processes *in vitro* is based on: (i) an untransformed model cell line, (ii) application of genetic or chemical perturbations, (iii) selection of DNA alterations conferring a growth advantage by soft agar colony formation, (iv) screening for extensive copy number alterations using low-pass whole-genome sequencing, and (v) in-depth characterization of DNA structural rearrangements (SRs) by long-range paired-end sequencing (Korbel *et al*, 2007) (*i.e*., mate-pair sequencing) (Fig1A).

Generation of RPE-1 *TP53*^−/−^ cells. Exemplary immunoblot designed for detecting clones with loss of p53 expression. To generate cell lines with loss of function of *TP53*, cells were transfected with zinc finger nucleases and grown to colonies after single cell sorting in the absence of selective pressure. Cells were challenged with 1 h of doxorubicin to elevate p53 expression, before immunoblotting. P-γ-H2AX was used to confirm the DNA damage, and GAPDH antibody was used as a loading control. The red arrowhead indicates the full-length p53 gene product. Clones picked for further studies (C111, C29) are highlighted. The remaining two clones did not show loss of p53 function despite having low levels of p53.

Flow cytometry of DNA content showing increased ploidy for C29.

Chromosome counts based on metaphase spreads verifying C29 hyperploidy (Appendix Fig S2B shows representative images). At least 20 metaphases were counted for each sample. Box-and-whiskers plots: boxes show the upper and lower quartiles (25–75%) with a line at the median, whiskers extend from the 10 to the 90 percentile and dots correspond to the outliers.

Exemplary read-depth plots based on low-pass WGS data generated using CAST. Red lines indicate segmentation results. Upper panels: diploid samples with SRs; lower panel: hyperploid samples, frequently exhibiting copy number alterations of ≥ 10 on one chromosome.

**Figure EV1:**
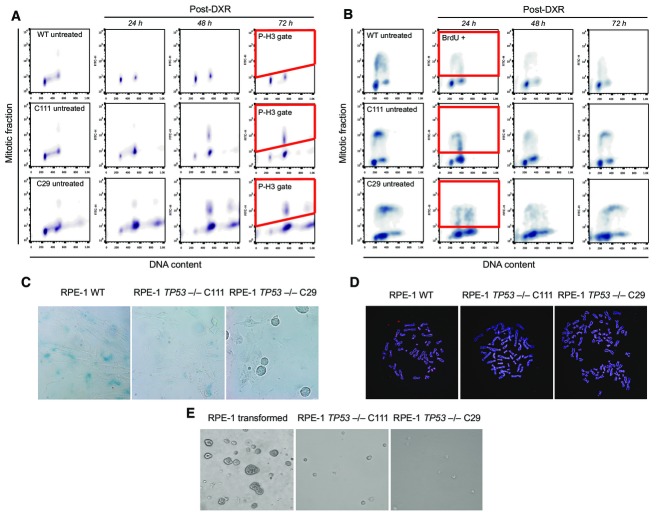
Characteristics of the RPE-1 *TP53*^−/−^ cell lines Assays for loss of function of p53 in RPE-1 WT and the *TP53*^−/−^ cell lines, C29 and C111. Cells were challenged with doxorubicin (DXR, 1.5 μM) for 1 h and then released into fresh media. The cells were released into medium containing 100 ng/ml nocodazole. Since p53 is a mediator of the DNA damage response in G2/M transition, cells with functional p53 are expected to be arrested in G2 due to massively damaged DNA (Bunz *et al*, 1998). When the DNA damage response is lost due to lack of p53 function, cells can enter mitosis where they are trapped with nocodazole. After 24, 48, and 72 h of incubation, cells were collected and stained with phosphohistone H3 antibody, a marker for mitotic cells. Cells were then analyzed by flow cytometry for their DNA and mitotic content. The gating for mitotic cells positive for P-H3 is exemplified in red after 72 h post-doxorubicin.

- Model cell line: We chose the human hTERT RPE-1 (retinal pigment epithelial) cell line as a model system for characterizing *de novo* SR formation. This telomerase immortalized cell line exhibits a genomically stable diploid karyotype. Though not tumor derived, RPE-1 cells can be transformed with elevated levels of γ-irradiation leading to gross SR formation detectable by karyotyping. We subjected hTERT RPE-1 (herein termed “RPE-1 wild type”) and previously generated (Riches *et al*, 2001) RPE-1-transformed cell lines to mate-pair sequencing, which revealed the occurrence of several SRs only in the transformed lines (Appendix Fig S1A–C). We recently described a link between *TP53* mutations and chromothripsis, implying that abnormal p53 function may be necessary for the induction, or tolerance, of catastrophic SRs (Rausch *et al*, 2012a). To establish a model amenable to study chromothripsis, we thus used zinc finger nucleases to generate an RPE-1 derivative deficient in p53. We confirmed p53 loss of function in two independent cell lines, C111 and C29 (Figs1A and EV1A–C). Interestingly, C29, but not C111, showed an increase in ploidy measured by both DNA content and chromosome counts from metaphase spreads (Figs1B and C, and EV1D), which may be explained with the previously noted tendency toward tetraploidization upon p53 inactivation (Bunz *et al*, 2002).
- Cellular perturbations: For a proof of concept of CAST, we employed sublethal doses of the chemical doxorubicin as a source of DNA double-strand breaks mediated through topoisomerase II inhibition in S phase (Fornari *et al*, 1994).
- Selection barrier: Complex genomic rearrangements resulting from chromothripsis can lead to the simultaneous acquisition of multiple tumor-promoting lesions (Stephens *et al*, 2011) (*e.g.,* loss of several tumor suppressors). Such lesions promote anchorage-independent cell growth *in vitro*, a hallmark of transformation and an established *in vitro* indicator of tumorigenicity (Hahn *et al*, 1999). We thus reasoned that such a strong selection barrier could be used to selectively enrich for cells harboring catastrophic SRs.
- Screening for extensive copy number switches: To design low-pass whole-genome sequencing experiments, we performed simulations to investigate what level of coverage is required to reliably detect large SRs; and we chose to use pooled, barcoded samples (up to 40 in one sequencing lane) to achieve around 0.05–0.1× genomic sequencing coverage. When assessing low-pass sequenced genomes, we used the circular binary segmentation algorithm on genomic read-depth data to infer copy number switches. We considered copy number switches of ≥ 500 kb when assessing low-pass whole-genome sequencing (WGS) data.
- Characterization of chromothripsis events: Whenever appropriate, cell lines were analyzed in-depth by mate-pair sequencing to high physical coverage (i.e., spanning coverage) in order to enable verification and characterization of chromo-thripsis events.

The cells were released into fresh medium. Since p53 also functions during G1/S transition, cells with functional p53 are expected to be arrest in G1 while the cells lacking p53 function continue cycling. After 24, 48, and 72 h of incubation, cells were pulsed for 1 h with BrdU in order to detect ongoing DNA replication. Cells were then analyzed by flow cytometry for their DNA and S phase content. The gating for mitotic cells positive for BrdU-FITC is exemplified in red after 24 h post-DXR. Please note the decrease in BrdU-positive cells in RPE-1 WT as well as the increase in G1 cell populations. In stark contrast, *TP53*^−/−^ cells have ongoing DNA replication even after massive DNA damage.

Detection of senescent cells through measurement of β-galactosidase activity at pH 6, reflecting a known characteristic of senescent cells not found in dividing, quiescent or immortal cells. Cells were released into fresh media. After 72 h, the activity of β-galactosidase was assessed. Experiments were done in triplicate, and exemplary images are shown. Note that the RPE-1 WT cells were stained positive for β-galactosidase indicating senescence. In contrast, very few cells in *TP53*^−/−^ cell lines were stained positive. Moreover, we frequently found dividing cells indicating a cycling population only in cells with non-functional p53.

Exemplary images of the metaphase spreads of the RPE-1 WT and the *TP53*^−/−^ cell lines. Cells were fixed and stained with the pan-centromere probe for probing the centromeric regions of all chromosomes, and Hoechst to mark chromosomes. Experiments were done in triplicate. The mean count distribution is plotted in Fig1C.

Exemplary images of RPE-1 cell lines after soft agar assay. Transformed RPE-1 cells as well as the two *TP53*^−/−^ cell lines were incubated in soft agar for 25 days and imaged afterwards. Experiments were done in triplicate. The transformed cells were able to form colonies in agar, whereas the untransformed C29 and the C111 clones were not.

Although a small number of isolated (or “simple”) SRs occurred upon p53 loss of function, we verified that in spite of *TP53* disruption neither C111 nor C29 showed signs of transformation (FigEV1E), suggesting their utility for CAST. Upon DNA damage induction, we sorted between 192 and 480 single cells into microtiter plates after 3 days to ensure that the cells go through at least one division following perturbations. We also ensured isolation of single cell-derived clones—by growing single colonies after cell sorting, and by isolating clones following transformation. Following DNA damage, sorting, and transformation, typically 3–16 clones were recovered per experiment, which were then subjected to low-pass WGS. Consistent with prior reports connecting tetraploidy to genomic instability (Fujiwara *et al*, 2005), we identified significantly more copy number switches in low-pass WGS data generated for 36 C29 hyperploid transformants compared to 40 C111 diploid transformants (C29 copy number switches, mean and standard deviation: 41.61 ± 17.31; C111 mean and standard deviation: 18.1 ± 10.55; *P* < 2 × 10^−9^; Welch two-sample *t*-test). To exclude the possibility that intrinsic differences between C29 and C111 other than the difference in ploidy were responsible for the increase in copy number alterations, we generated another tetraploid cell line directly from the C111 cell line by preventing cytokinesis (dihydrocytochalasin B). Reassuringly, the resulting tetraploid cell line (DCB2) (Appendix Fig S2) amounting to 22 additional transformed clones (Table EV1) exhibited a significantly higher level of copy number alterations as those derived from the diploid cell line C111 (DCB2, copy number switches, mean and standard deviation: 34.39 ± 17.60; *P *<* *3 × 10^−4^, when compared to C111 by Welch two-sample *t*-test).

### Generation of chromothripsis *in vitro* in hyperploid RPE-1 cells

**Figure 2:**
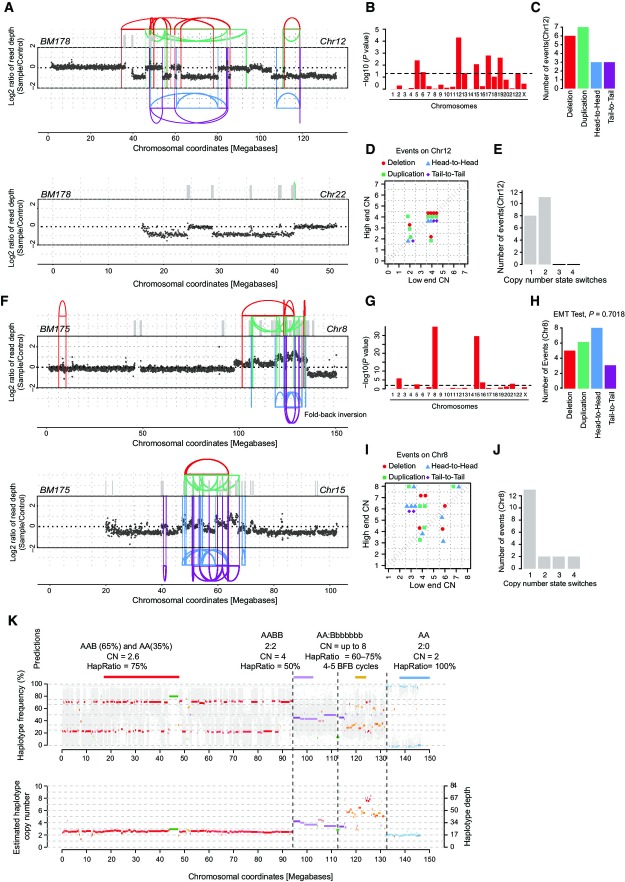
Link between chromothripsis and hyperploidy DNA alteration patterns of chromosomes 12 and 22 in BM178 based on mate-pair and WGS data, with highly oscillating copy number profiles consistent with the occurrence of chromothripsis. SRs are color-coded: red, deletion type (T-H); green, duplication type (H-T); blue, head-to-head (H-H) type; purple, tail-to-tail (T-T) type; gray, inter-chromosomal.

Notably, we observed individual examples of highly clustered copy number alterations in nine cases, all of which arose in hyperploid lineages (hyperploids: 9/58; diploids; 0/40; *P *<* *0.01; two-sided Fisher’s exact test; Fig1D, Dataset EV1), and none of which were observed during control arms of our experiments omitting the selection barrier (Appendix Fig S3). We used mate-pair sequencing for in-depth investigation of 29 hyperploid and 29 diploid transformants exhibiting copy number alterations (Table EV2). Subsequently, we employed recently published criteria (Li *et al*, 2014) and deductive approaches (Korbel & Campbell, 2013) for evaluating the occurrence and temporal ordering of clustered SRs and to test for chromothripsis events (details on the underlying reasoning (Korbel & Campbell, 2013; Li *et al*, 2014) are summarized in the Appendix). Applying stringent criteria, we verified the occurrence of chromothripsis in seven hyperploid transformants and additionally confirmed that there is no evidence for chromothripsis events in the diploid transformants. Hence, our analysis indicates an increased rate of chromothripsis events in hyperploids compared to diploids (*P *<* *0.05; two-sided Fisher’s exact test; Fig2 and Appendix Figs S4–S9). The tendency of chromothripsis events to occur in hyperploids was further supported by CAST experiments using Zeocin (rather than doxorubicin) as a DNA double-strand break inducing chemical, which revealed 2/12 chromothripsis in C29 hyperploid versus 0/12 chromothripsis in C111 diploid cell line (FigEV2). For example, chromosome 12 of the RPE-1 cell line BM178, which we derived from doxorubicin treatment of the hyperploid RPE-1 line C29, exhibited the prototypical chromothripsis pattern of oscillating copy numbers (Fig2A–E, Appendix Fig S4) that numerous studies previously reported in cancer genome surveys (e.g., Kloosterman *et al*, 2011; Stephens *et al*, 2011; Rausch *et al*, 2012a; Korbel & Campbell, 2013; Li *et al*, 2014; and references therein). Further analyses showed that chromosomes 12 and 22 underwent co-shattering in BM178, with abundant translocation calls connecting both chromosomes (Appendix Fig S4), an outcome of chromothripsis that has also been observed frequently in cancer genomes (Stephens *et al*, 2011; Korbel & Campbell, 2013).

Statistically significant deviation from null hypothesis of no breakpoint clustering in BM178 (*y*-axis depicts -log_10_(*P*-value) for KS test applied to each chromosome), in line with the occurrence of chromothripsis (see Appendix notes, inference of chromothripsis, criterion 1).

Randomness of DNA fragment joins (Korbel & Campbell, 2013) for BM178 chromosome 12 (EMT test, *P *=* *0.5041), additionally supporting the occurrence of chromothripsis. *P*-value derived from multinomial testing against null hypothesis “equal distribution of joins” (see Appendix notes, inference of chromothripsis, criterion 2).

Copy number jump distribution (Li *et al*, 2014) for chromosome 12 in BM178. Points on the diagonal indicate that chromothripsis occurred on a previously intact chromosome. (Appendix notes, inference of chromothripsis, criterion 3).

Copy number segment switches (Li *et al*, 2014) for chromosome 12 in BM178, consistent with chromothripsis occurring as the initial event (see Appendix notes, inference of chromothripsis, criterion 3).

DNA alteration patterns of chromosomes 8 and 15 in BM175 based on mate-pair and high-coverage WGS data, with highly oscillating copy number profiles consistent with the occurrence of chromothripsis.

Statistically significant deviation from null hypothesis of no breakpoint clustering in BM175 (*y*-axis depicts -log_10_(*P*-value) for KS test applied to each chromosome), in line with the occurrence of chromothripsis.

Randomness of DNA fragment joins (Korbel & Campbell, 2013) for BM175 chromosome 8 (EMT test, *P *=* *0.7018), additionally supporting the occurrence of chromothripsis.

Copy number jump distribution (Li *et al*, 2014) between joined fragments of chromosome 8 in BM175 (displayed for segments with confident copy number ascertainment). Most copy number jumps are off the diagonal, indicating that chromothripsis occurred on a previously rearranged chromosome.

Copy number segment switches (Li *et al*, 2014) for chromosome 8 in BM175, consistent with BFBs occurring prior to chromothripsis.

Evidence for chromothripsis occurring on one single parental haplotype. Based on the haplotype frequencies, and the copy number estimates, we inferred that the majority of the chromosome 8 is present in 2 copies, but possibly due to an early subclonal event, two subclones exhibiting separate haplotype ratios (AA and AAB) exist. The shift in the haplotype ratio and the increase in copy number suggest that the B haplotype underwent several (up to 5) cycles of BFB as well as a chromothripsis event, resulting in amplified segments of as high as 8 copy numbers. The sharp drop close to the chromosome end is associated with a shift to a haplotype ratio of 0, *that is*, complete loss of the B haplotype (in line with the BFB and chromothripsis events occurring on the same haplotype). Predictions of haplotype segments are indicated in the above panel.

**Figure EV2:**
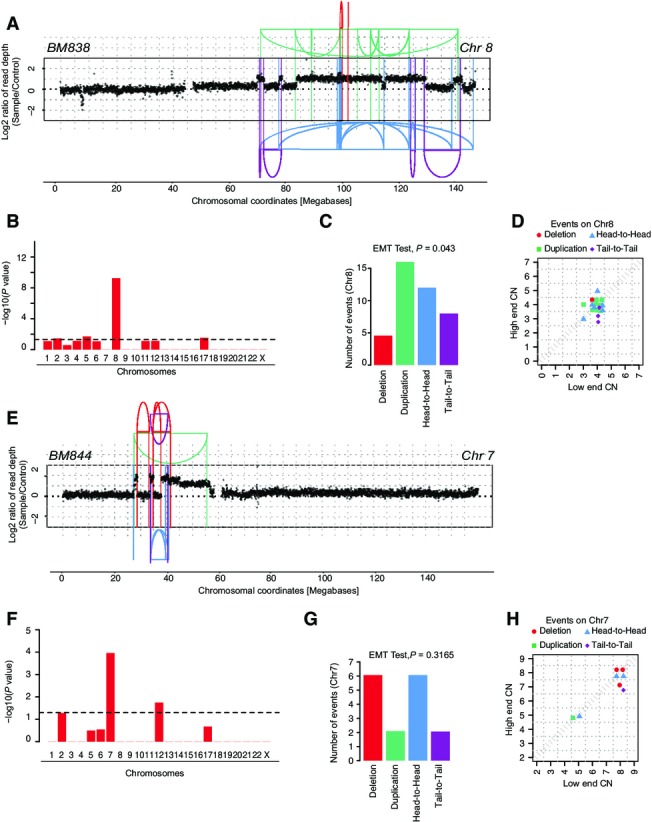
Analysis of chromothripsis cell lines identified post-Zeocin treatment and agar selection In BM838, derived from hyperploid C29 clone, chromothripsis was observed on chromosome 8. The oscillating pattern of copy numbers and mostly copy number jumps of 0 resulted from a “classical” chromothripsis event with 40 breakpoints, which occurred on a previously unrearranged chromosome. SRs are color-coded based on their orientation: red, deletion type (T-H); green, duplication type (H-T); blue, head-to-head (H-H) type; purple, tail-to-tail (T-T) type.

Statistically significant deviation from null hypothesis of no rearrangement breakpoint clustering in BM838, in line with the occurrence of chromothripsis on chromosome 8. The *y*-axis shows the -log_10_(*P*-value) of KS test applied to each chromosome.

Randomness of SR joins on chromosome 8 (EMT test, *P *=* *0.043). In this sample, we found an apparent tendency of rearrangements to be slightly biased toward duplication-type rearrangements. In the context of the classical pattern of oscillating copy numbers with mostly zero copy number jumps and the strong genome-wide concentration of rearrangements to only this chromosome arm, we did not regard this apparent tendency as evidence against the occurrence of chromothripsis.

Copy number jump distribution between joined fragments of chromosome 8, indicating that chromothripsis occurred on a previously unrearranged chromosome (Li *et al*, 2014).

In BM844 derived from hyperploid clone C29, we detect chromothripsis on chromosome 7 with 16 breakpoints. A stepwise increase in copy number segments followed by a sharp drop toward the chromosome end was additionally observed, suggesting occurrence of a BFB cycle. SRs are color-coded based on their orientation: red, deletion type (T-H); green, duplication type (H-T); blue, head-to-head (H-H) type; purple, tail-to-tail (T-T) type; gray, inter-chromosomal.

Statistically significant deviation from null hypothesis of no rearrangement breakpoint clustering in BM844, in line with the occurrence of chromothripsis on chromosome 7. The *y*-axis shows the -log_10_(*P*-value) of KS test applied to each chromosome.

Randomness of SR joins on chromosome 7 (EMT test, *P *=* *0.3165) further supporting evidence for chromothripsis.

Copy number jump distribution between joined fragments of chromosome 7. The high CN step distribution might suggest that a number of duplications occurred following chromothripsis, presumably involving 1-2 BFB cycles.

An additional example of chromothripsis, detected in BM175 which also resulted from doxorubicin treatment of the hyperploid line C29, is shown in Fig2F–K. Chromosome 8 in this cell line exhibited an amplified region adjacent to a sharp drop in copy number toward the chromosome end. Notably, the boundaries of the amplified region were further demarcated by a fold-back inversion. These genomic patterns indicate the emergence of breakage–fusion–bridge cycles (BFBs) (Campbell *et al*, 2010), which are thought to underlie a genomic instability process initiated by a double-strand break exposing the end of the affected chromosome (Bunting & Nussenzweig, 2013). During replication, the unstable chromosome ends can fuse by non-homologous end joining, creating a dicentric chromosome that can break during cell division. This characteristically leads to a stepwise increase in copy number toward the chromosome end, followed by a sharp drop (Campbell *et al*, 2010), as detected in BM175 (Fig2F–K). In the case of BM175, however, copy number oscillations resulting from chromothripsis overlaid the characteristic genomic patterns of BFBs (Fig2F–K). On the basis of our analysis, the architectural characteristics of rearrangements on BM175 chromosome 8 indicate that chromothripsis occurred subsequent to initiating BFBs, as when chromothripsis occurs on a previously rearranged chromosome, the resulting segment joins will connect genomic pieces exhibiting different copy number states (see Appendix, inference of chromothripsis events (Li *et al*, 2014; Rausch *et al*, 2012a; Stephens *et al*, 2011)). The extensive translocations to chromosomes 15 and 2 suggest that these chromosomes were most likely co-shattered by chromothripsis, which resulted in highly rearranged derivative chromosomes (Dataset EV1). In addition to BM175, our analysis uncovered other examples of chromothripsis events arising subsequently to BFB cycles (Appendix Figs S5, S7, and S12). Together with earlier observations on the occasional co-occurrence of BFBs and chromothripsis in acute lymphoblastic leukemia and esophageal adenocarcinoma (Li *et al*, 2014; Nones *et al*, 2014), these data suggest that the temporal pattern of BFBs followed by chromothripsis is a more common feature of massively rearranged genomes.

### Evidence for a link between telomere attrition and chromothripsis

**Figure 3:**
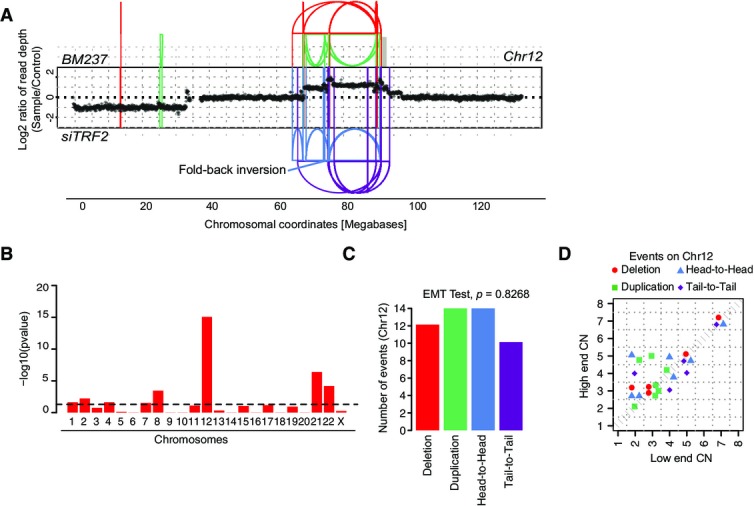
Evidence for chromothripsis in TRF2-depleted cells DNA alteration patterns of chromosome 12 in BM237 based on mate-pair data. Highly oscillating copy number profiles are consistent with the occurrence of chromothripsis. SRs are color-coded: red, deletion type (T-H); green, duplication type (H-T); blue, head-to-head (H-H) type; purple, tail-to-tail (T-T) type; gray, inter-chromosomal.

**Figure EV3:**
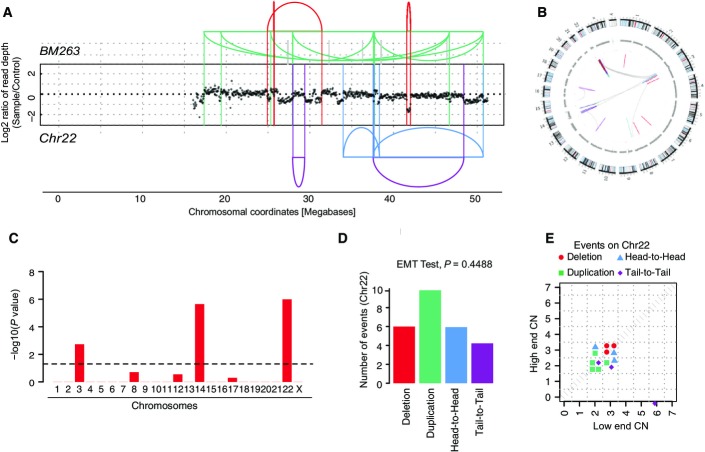
Further evidence for chromothripsis in TRF2-depleted cells DNA alteration patterns of chromosome 22 in BM263 based on mate-pair data. Highly oscillating copy number profiles are consistent with the occurrence of chromothripsis. SRs are color-coded: red, deletion type (T-H); green, duplication type (H-T); blue, head-to-head (H-H) type; purple, tail-to-tail (T-T) type; gray, inter-chromosomal.

BFB cycles can be triggered through losses of telomeric ends of chromosomes (Bunting & Nussenzweig, 2013). In order to test whether telomeric end loss can give rise to chromothripsis using our CAST system, we depleted cells of the shelterin complex protein TRF2 (de Lange, 2005) and further challenged the siRNA-treated cells with chemicals used to help overcome mitotic checkpoint barriers in TRF2-depleted cells (i.e., reversine and hesperadine (Santaguida *et al*, 2010)). Notably, we identified two chromothripsis events (Figs3 and EV3) one of which additionally exhibited the genomic alteration patterns of a BFB cycle (Fig3). To our knowledge, these data provide, for the first time, experimental evidence that telomere attrition can mediate chromothripsis. Hence, our data add to and significantly extend prior observations based on cancer genome analysis that BFBs, which are thought to frequently arise as a consequence of telomere shortage, occasionally occur in conjunction with chromothripsis events (see Li *et al*, 2014; Nones *et al*, 2014; as well as our genomic data in Fig2F–K and Appendix Figs S5, S7, and S12). Furthermore, following *TRF2* depletion, the RPE-1 cells notably became hyperploid and stayed in hyperploid condition (data not shown), in support of the association of hyperploidy with chromothripsis that we observed for doxorubicin-treated RPE-1 cells.

Statistically significant deviation from null hypothesis of no breakpoint clustering in BM237 (*y*-axis depicts -log_10_(*P*-value) for KS test applied to each chromosome), in line with the occurrence of chromothripsis.

Randomness of DNA fragment joins for BM237, additionally supporting the occurrence of chromothripsis. *P*-value is derived from multinomial testing against null hypothesis “equal distribution of joins.”

Copy number jump distribution between joined fragments of chromosomes undergoing chromothripsis (displayed for segments with confident copy number ascertainment). On chromosome 12 in BM237, chromothripsis occurred most likely in conjunction with breakage–fusion–bridge cycles.

Circos plot depicting inter- and intra-chromosomal connections in BM263.

Statistically significant deviation from null hypothesis of no breakpoint clustering in BM263 (*y*-axis depicts -log_10_(*P*-value) for KS test applied to each chromosome), in line with the occurrence of chromothripsis.

Randomness of DNA fragment joins for BM263, additionally supporting the occurrence of chromothripsis. *P*-value derived from multinomial testing against null hypothesis “equal distribution of joins.”

Copy number jump distribution between joined fragments of chromosomes undergoing chromothripsis (displayed for segments with confident copy number ascertainment). On chromosome 22 in BM263, chromothripsis occurred most likely in isolation.

### Link between hyperploidy and chromothripsis in medulloblastoma

**Figure 4:**
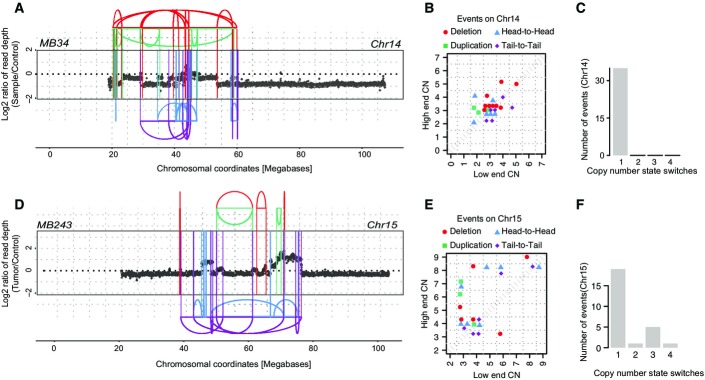
Evidence for hyperploidy being a risk factor for chromothripsis in SHH-type medulloblastoma Oscillating copy number states and SRs on chromosome 14 in MB34 (based on WGS data), resulting from chromothripsis. SRs are color-coded: red, deletion type (T-H); green, duplication type (H-T); blue, head-to-head (H-H) type; purple, tail-to-tail (T-T) type; gray, inter-chromosomal.

To determine whether hyperploidy is also linked to chromothripsis *in vivo*, we performed an analysis of WGS data from pediatric medulloblastoma tumor specimens (Jones *et al*, 2012; Rausch *et al*, 2012a; Kool *et al*, 2014), since chromothripsis is common and a presumed early event in medulloblastoma tumorigenesis (Rausch *et al*, 2012a). Chromothripsis and tetraploidy have been reported to occur in medulloblastoma (Jones *et al*, 2012; Rausch *et al*, 2012a), but a statistical association between chromothripsis and hyperploidy has to date not been established. We focused on the Sonic-hedgehog pathway-driven medulloblastoma subtype (SHH-MB), a subtype in which chromothripsis is associated with inactivating germ line *TP53* mutations (Rausch *et al*, 2012a), and which hence exhibits genetic similarity to our *TP53*-deficient model cell line. We reanalyzed cancer genomes from 44 previously published SHH-MBs (Jones *et al*, 2012; Rausch *et al*, 2012a; Kool *et al*, 2014) and additionally generated 30× coverage tumor and matched blood WGS data for a recently diagnosed SHH-MB patient with a germline mutation in *TP53* (MB243). Our analyses demonstrate that chromothripsis indeed occurs significantly more often in hyperploid compared to diploid SHH-MBs (hyperploids: 5/11; diploids: 2/34; *P *<* *0.01; one-tailed Fisher’s exact test; Table EV3). An exemplary chromothripsis event for MB34, showing excessive oscillating copy number alterations, is shown in Fig4A–C and Appendix Figs S10 and S11. Similar to our cell-based model system, we also observed chromothripsis events preceded by BFB cycles in these data from tumors *in vivo*, for instance on chromosome 15 in MB243 (Fig4D–F and Appendix Fig S11). Notably, based on our analyses of SRs, copy number states, and haplotype-based profiling of allelic imbalances, patterns of somatic alteration in SHH-MB are consistent with hyperploidization preceding chromothripsis, implicating hyperploidization as a “risk factor” for chromothripsis *in vivo* (FigEV4).

Copy number jump distribution for chromosome 14 in MB34, with diagonal points indicating chromothripsis occurred on a previously unrearranged chromosome.

Distribution of copy number segment switches for chromosome 14 in MB34.

Oscillating copy number states and SRs on chromosome 15 in MB243 (based on WGS data), resulting from chromothripsis and BFBs.

Copy number jump distribution for chromosome 15 in MB243, with off-diagonal points indicating chromothripsis occurred on a previously rearranged chromosome.

Distribution of copy number segment switches for chromosome 15 in MB243.

**Figure EV4:**
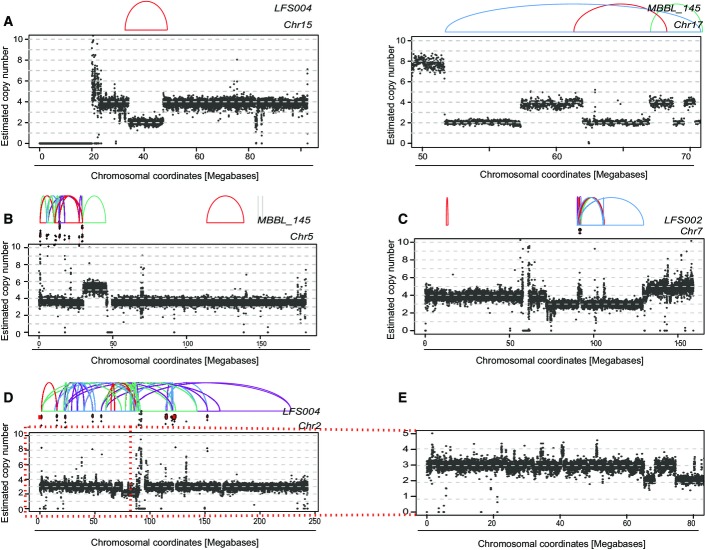
Evidence for tetraploidy being an initiating event in SHH-MBs The plots display read depth from raw read counts with estimated copy numbers together with SR graphs. SRs are color-coded as indicated in FigEV3 earlier. Two exemplary SR patterns, underlying presumably sequential SRs, exhibiting copy number state switches of 2 (indicating that these sequential SRs arose before tetraploidy).

Estimated copy number of MB145, chromosome 5 together with SRs. Double minute chromosomes resulting from chromothripsis are indicated off-scale.

Estimated copy number of LFS02, chromosome 7 together with SRs. (Double minute chromosomes resulting from chromothripsis indicated off-scale).

Estimated copy number of LFS04, chromosome 2 together with SRs. (Double minute chromosomes resulting from chromothripsis indicated off-scale). The region with numerous copy number switches of magnitude of 1 is highlighted and further examined in (E).

Zoom-in of chromosome 2, 0–80-Mb region. (Note the copy number switches of magnitude of 1). Data information: These three cases (B–D) all show examples of tumors bearing the hallmarks of chromothripsis with double minute formation (Stephens *et al*, 2011; Rausch *et al*, 2012a). Except the massively amplified double minute chromosomes (which typically undergo repeated duplication after chromothripsis has occurred (Rausch *et al*, 2012a; Stephens *et al*, 2011)), all SRs were associated with copy number segment switches of 1, based on which we predict that tetraploidy (hyperploidy) preceded chromothripsis.

### Functional consequences of chromothripsis in RPE-1 cells

**Figure 5:**
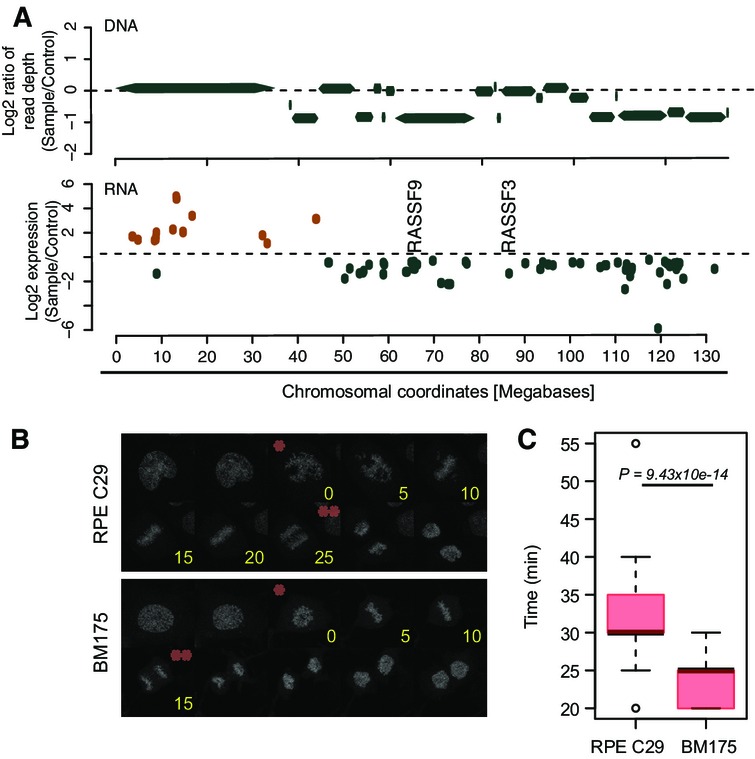
Transcriptional consequences of chromothripsis in BM178 and BM175 Differentially expressed genes on the chromothripsis-affected chromosome 12 of BM178, compared to its parental line C29 (FDR = 10%). Log_2_-fold changes of differentially expressed genes are depicted. The above panel shows segmented DNA copy number for this chromosome. Orange, upregulated. Green, downregulated.

**Figure EV5:**
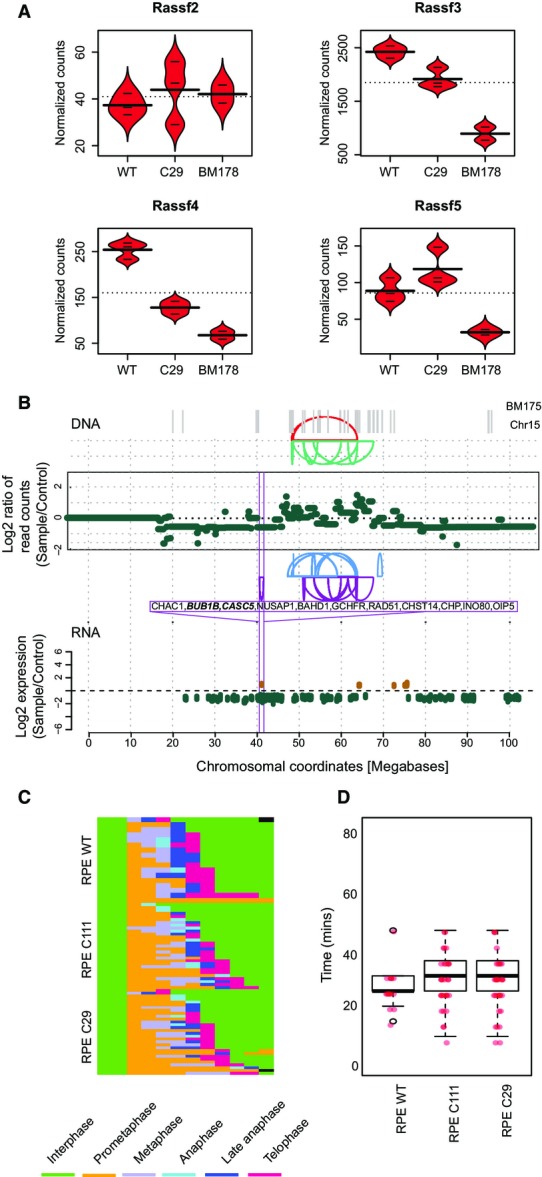
Transcriptional consequences of chromothripsis Transcriptional regulation of RASSF2/3/4/5. Bean plots from normalized counts of each mRNA are shown for RPE-WT, RPE-C29, and BM178. Horizontal lines represent values from individual samples, whereas thick horizontal lines indicate average values. Experiments were done in triplicates. Except for RASSF2, which exhibited very low read counts in our cell lines, all other members showed significant downregulation in BM178 (*P*_adj_ < 0.1).

Our method, in conjunction with the availability of isogenic cell lines prior to and subsequent to chromothripsis, will not only enable probing for chromothripsis initiating genetic factors, but also facilitate studies of the consequences of catastrophic SRs under controlled experimental conditions. To exemplify this, we performed transcriptome sequencing (mRNA-Seq) of BM175 and BM178 as well as of their parental cell lines and compared gene expression levels in pre- and post-chromothripsis stages. We observed appreciable expression changes in genomic regions affected by chromothripsis at a false discovery rate (FDR) of 10%. For instance, in BM178, a number of significantly downregulated genes were observed on the chromosome arm rearranged by chromothripsis, including two tumor suppressors (the *RASSF3* and *RASSF9* members of the RAS-associated family of tumor suppressors (Volodko *et al*, 2014); Fig5A). Additional members of this tumor suppressor family (RASSF4/5) residing on different chromosomes were likewise downregulated (Fig EV5A), potentially due to coordinated regulation of these interacting partners (Behrends *et al*, 2010) subsequent to the rearrangement. For BM175, we observed pronounced downregulation of *BUBR1* and *CASC5* residing on chromosome 15, which was the most significantly affected chromosome in terms of expression deregulation, with 14% of the expressed genes showing significant deregulation (chi-squared outlier test, *P *=* *0.0048). As a result of chromothripsis, both genes were brought into a new genomic context (FigEV5B–D). The products of both genes are involved in faithful kinetochore–microtubule attachments (Foley & Kapoor, 2013), and their downregulation was previously reported to result in reduced mitotic timing (Kittler *et al*, 2007). Further supporting our gene expression measurements, high-resolution live cell imaging showed significantly decreased mitotic timing in BM175 relative to its parental line, presumably due to reduced expression of *BUBR1* and *CASC5* (*P *<* *9.4e-14; Welch two-sample *t*-test; Fig5B and C). Taken together, these analyses exemplify investigation of the consequences of chromothripsis as an additional use case of CAST.

Mitotic progression analysis consistent with downregulation of *BUBR1* and *CASC5* on the chromothripsis-affected chromosome 15 in BM175. Histone H2B was constitutively tagged with red fluorescent protein mCherry to visualize chromatin in BM175 and C29. Exemplary images of live cell experiments are shown. Asterisks indicate chromosome condensation (*) and segregation (**) time points. Time is indicated in minutes (yellow).

Timing from chromosome condensation to chromosome segregation (quantification of data in (B)) was plotted for dividing cells of C29 (*n *=* *54) and BM175 (*n *=* *33; *P*-value based on Welch two-sample *t*-test). Box-and-whiskers plots: boxes show the upper and lower quartiles (25–75%) with a line at the median, whiskers extend from the 10 to the 90 percentile and dots correspond to the outliers.

Significantly differentially expressed genes on chromosome 15 of BM175 (compared to the parental cell line C29) at an FDR of 10%. Log_2_-fold changes of each gene are shown. In the above panel, copy number segments and SRs affecting chromosome 15 are depicted. Genes significantly affected by this SR are highlighted in green (downregulation) and brown (upregulation).

Individual tracks of mitotic cells that were imaged and analyzed automatically by CellCognition (www.cellcognition.org). Each mitotic class is color-coded as indicated in the legend (*n*^WT^ = 15, *n*^C^^111^ = 31, *n*^C^^29^ = 33).

Distribution of timing of mitotic progression (prometaphase, metaphase and anaphase in minutes) in RPE-1 WT and RPE-1 *TP53*^−/−^ cell lines derived from Fig EV5C. We did not detect any significant changes between WT and *TP53*^−/−^ cell lines. Box-and-whiskers plots: boxes show the upper and lower quartiles (25–75%) with a line at the median, whiskers extend from the 10 to the 90 percentile and transparent dots correspond to the outliers. Red dots represent individual samples.

## Discussion

CAST makes one of the most striking outcomes of cancer genome analyses identified to date—the discovery of chromothripsis as a one-off catastrophic rearrangement process (Stephens *et al*, 2011)—amenable to laboratory studies, enabling investigations of causes and consequences of chromothripsis. CAST utilizes initially untransformed cell lines together with a strong selection barrier that can be overcome following genetic and chemical perturbations. Our experimental data based on CAST do not only demonstrate the reproducible generation of chromothripsis events, but also implicate hyperploidy and telomere attrition as predisposing factors for chromothripsis. Along with the link between *TP53* germ line mutations and chromothripsis that we previously reported for medulloblastoma (Rausch *et al*, 2012a), and links between constitutional Robertsonian translocations and chromothripsis in acute lymphoblastic leukemia (Li *et al*, 2014), our *in vitro* findings reinforce the notion that rather than occurring in isolation chromothripsis is prone to arise in cellular contexts which facilitate genomic instability—such as in the context of hyperploidy, which may mediate instability by “buffering” against haploinsufficiency or by causing an increased rate of mitotic failures promoting SR formation. Indeed, hyperploidy is frequently observed in human cancers (Davoli & de Lange, 2011), can increase resistance to chemotherapy and radiotherapy (Castedo *et al*, 2006), and has previously been suggested as a source of genetic instability (Storchova & Kuffer, 2008; Davoli & de Lange, 2011), albeit not in the context of chromothripsis. Interestingly, an important feature of external granular layer (EGL) cells, the cells of origin of SHH-MB, is their capacity to actively proliferate in a tissue where most other cell types do not divide (Schuller *et al*, 2008; Westra *et al*, 2008). Potentially relevant to the observations reported here, a previous study using cultured EGL progenitors identified aneuploidy in 15% of cells, with hyperploidy being the most commonly observed form (Westra *et al*, 2008).

In addition to identifying hyperploidy as a risk factor for chromothripsis, our experiments implicate telomere attrition (mediated by siRNA-based *TRF2* depletion) in chromothripsis. Of note, telomere deprotection has previously been hypothesized to be associated with chromothripsis (Stephens *et al*, 2011; Li *et al*, 2014) as this can result in dicentric chromosomes that may be prone to breakage during cell division (van Steensel *et al*, 1998), and given the occasional co-occurrence of BFBs and chromothripsis in cancer genomes. Although TRF2 depletion appears to be less efficient for generating DNA rearrangements than doxorubicin, perhaps due to additionally required genetic components, our *in vitro* data demonstrate how CAST may be used for future research to uncover natural cellular, or extrinsic, triggers of chromothripsis.

CAST, which utilizes initially genetically stable cells as *in vitro* “model system,” can be employed using diverse chemical or genetic perturbations. While this paper was under review elsewhere, a different experimental approach, by Zhang and colleagues, was described using live cell imaging and single cell sequencing, based on which the authors elegantly showed that the physical isolation of chromosomes through micronuclei formation can mediate chromothripsis events (Zhang *et al*, 2015). Although we failed to detect micronuclei in our cell lines after chromothripsis occurred (Appendix Fig S12), this does not formally exclude a potential initiating role of micronuclei in chromothripsis in our RPE-1 cell system. Micronuclei can form as a consequence of chromosomes lagging at anaphase (Crasta *et al*, 2012), dicentric chromosomes are prone to be lagging at anaphase (Pampalona *et al*, 2010), and telomere attrition can mediate the formation of dicentric chromosomes (van Steensel *et al*, 1998). Hence, it is conceivable that micronuclei arose in our cells treated with TFR2 siRNA, presumably when double-strand breaks occurred following telomere attrition.

By comparison, and complementary to the approach by Zhang *et al*, CAST can be used to investigate the potential lethality of massive SRs resulting from chromothripsis, the ability of cells to benefit from these, and additional consequences of chromothripsis. By enabling research on these aspects for the first time, CAST provides a powerful tool for investigating natural triggers and consequences of chromothripsis, a catastrophic SR process that is likely to be initiated by more than one cellular pathway. In this context, the flexibility of our approach along with its capacity to reproducibly generate chromothripsis events will facilitate further studies on chromothripsis, by enabling the testing of specific hypotheses on pathways and mechanisms that may give rise to catastrophic SRs, including involvement of errors in mitosis, transcription, and DNA replication (Jones & Jallepalli, 2012).

## Materials and Methods

### Cell lines and treatments

RPE-1 cells were purchased from ATCC and checked for mycoplasma contamination. The cells were cultured in DMEM-F12 medium supplemented with 10% fetal bovine serum and antibiotics (Life Technologies). *TP53*^−/−^ cell lines were generated by predesigned zinc finger nucleases (Sigma) that target the second exon of the *TP53* gene. After electroporation of the cells with the nucleases (Neon Transfection system, Life Technologies), cells were incubated for 48 h, single sorted, and grown to single colonies in the absence of any selection marker. Loss of p53 was assayed by immunoblotting after doxorubicin treatment. For functionality assays, 1.5 μM doxorubicin was applied for 1 h.

In the context of the CAST approach, cells were perturbed with sublethal concentrations of doxorubicin (0.15 μM) and Zeocin (50 μg/ml) for 12 h or hesperadine (100 nM) and reversine (5 μM) after 48 h of TRF2 siRNA (Ambion), released into fresh media, and incubated for 3 days before they were sorted for single cells in 96-well plates.

To generate tetraploid cells from the C111 clone, 2 μM dihydrocytochalasin B (DCB, Sigma), a chemical compound preventing remodeling of the actin cytoskeleton, was applied. After 20 h of DCB treatment, cells were single sorted and grown to colonies. Tetraploidy was assessed by flow cytometry for DNA content using Hoechst (33342, Life Technologies).

RPE-1 WT, C111, C29, and BM175-H2B-mCherry cells were generated by retroviral transduction of the pQCXIP-H2B-mCherry plasmid. Cells positive for mCherry were then sorted in bulk.

### Immunoblotting

Cells were seeded on 6-well plates to reach 70–80% confluency. Prior to cell extraction, cells were either treated with 1.5 μM doxorubicin for 1 h or left untreated. Cell extracts were then separated on precast gradient SDS–PAGE (Life Technologies) and subsequently transferred to nitrocellulose membranes using an electro-blotting system (iBlot, LifeTechnologies). Membranes were blocked with 5% nonfat skimmed milk in TBS-Tween (0.1%), and the membrane was probed with antibodies directed against p53 (Santa Cruz, DO-1), P-γ-H2AX (for detection of DNA damage) (Milipore, JBW301), and GAPDH (as loading control) (Cell Signaling Technologies, 14C10).

### Flow cytometry

A MoFlo legacy cell sorter (Beckman Coulter Inc.) equipped with a 100-μm nozzle was used during the flow cytometry analysis and sorting of RPE cells. A Coherent Innova 90C argon ion laser (Coherent Inc.), tuned to 488 nm TEM_00_ mode (200 mW), was used as primary laser in the detection of forward and side scatter profiles. A Coherent Sabre krypton ion laser tuned to 568 nm TEM_00_ mode (200 mW) was used as a secondary laser for excitation of mCherry. Laser illumination, Moflo’s L-configuration optical layout, and sorting were optimized using Flow-Check™ Fluorospheres (Beckman Coulter Inc.). BD FACSFlow™ (Becton Dickinson GmbH), filtered in-line through a PALL Fluorodyne II filter 0.2 μm (Pall GmbH), was used as sheath fluid. Acquisition was triggered on FSC. mCherry fluorescence intensity was measured after passing collected light through a 630/40-nm band-pass (BP) filter. A holographic 568 notch filter was placed in the 568-nm laser optical path to prevent laser spillover into the fluorescence detectors. MoFlo Summit software (Beckman Coulter) was used in sample analysis and sort gate definition during sorts.

Cell cycle profiles with P-H3 were determined by measuring cellular DNA content versus mitotic content by flow cytometry. Briefly, cells were washed twice with ice-cold PBS and resuspended in 1.5 ml of 70% ice-cold ethanol. Fixed cells were collected by centrifugation, washed twice with PBS, and stained with P-H3 antibody (Cell Signaling) for 1 h. After 2 washes with PBS, a secondary antibody coupled to Alexa488 (LifeTechnologies) dye was incubated with the cells for 30 min. After one washing step with PBS, cells were treated with 0.1 mg/ml of RNaseA (Sigma) for 30 min at 37°C, and stained with 0.01 mg/ml propidium iodide solution. For replication-associated bromodeoxyuridine (BrdU) immunofluorescent staining, cells were cultured on 10-cm-diameter plates up to a confluence of 90% and then were either treated with 1.5 μM doxorubicin for 1 h or left untreated. Treated and untreated cells were seeded on 6-well plates (80% confluency) and were pulsed labeled with 10 μΜ BrdU for 1, 24, 48, and 72 h post-doxorubicin. Cells were then fixed, treated with DNase to expose BrdU epitopes, and incubated with FITC-conjugated anti-BrdU antibodies and with 7-AAD total DNA staining dye following instruction of the manual (BD Pharmingen™ BrdU Flow Kit). Profiles were determined with an LSR-Fortessa SORP instrument (BD Biosciences) using a 355-nm laser (450/50 BP) as well as a 488-nm laser (530/30 BP). All post-acquisition analysis was done with FlowJo 9.6. (Tree Star, Inc).

### Senescence-associated β-galactosidase staining

Cells were cultured on 10-cm-diameter plates up to a confluence of 90% and then were either treated with 1.5 μM doxorubicin for 1 h or left untreated. Treated and untreated cells were seeded on 6-well plates (40–50% confluency) and were stained for senescence-associated β-galactosidase activity after 24, 48, and 72 h using the Senescence-βGal Staining Kit (Cell Signaling Technology) following the manufacturer’s instructions. All the experiments were repeated three times, and one of the representative results is shown.

### Transformation assays

After sorting, single cells were grown into colonies, before subjecting them to soft agar consisting of 0.5% bottom layer agar and 0.35% top layer agarose to assay for *in vitro* transformation. As an alternative, 96-well soft agar assays were purchased from CellBiolabs and used according to manufacturer’s instructions. The transformed colonies (observed after 30 days with our in-house protocol or after 10-15 days with the commercial kit) were recovered from 96-well plates, and recultured on 6-well plates. Single colonies were isolated and grown for further analysis.

### DNA libraries and sequencing

Genomic DNA was extracted from the cells using the DNA Blood Mini Kit (Qiagen). In the context of CAST, library preparation was performed with a Beckman Biomek FX automated liquid handling system, with 500 ng starting material using SPRIworks HT chemistry (Beckman Coulter). For low-coverage sequencing, samples were prepared with custom 6 base pair barcodes to enable pooling. Library quantification and quality control were performed using a Fragment Analyzer (Advanced Analytics Technologies, Ames, USA). WGS was pursued on an Illumina HiSeq 2500 platform (Illumina, San Diego, USA), using 50 base pair single reads for low-pass sequencing.

Mate-pair DNA library preparation was performed using the Nextera Mate Pair Sample Preparation Kit (Illumina). In brief, 4 μg of high molecular weight genomic DNA was fragmented by the tagmentation reaction in 400 μl, followed by strand displacement. Samples were size-selected to 4–5 kb following the Gel-Plus path of the protocol. A total of 300–550 ng of size-selected DNA was circularized in 300 μl for 16 h at 30°C. After an exonuclease digestion step to get rid of remaining linear DNA, fragmentation to 300–700 bp with a Covaris S2 instrument (LGC Genomics), and binding to streptavidin beads, the libraries were completed via End Repair, A-Tailing, and Illumina Truseq adapter ligation. The final sequencing library was obtained after PCR for 1 min at 98°C, followed by nine cycles of 30 s at 98°C, 30 s at 60°C, 1 min at 72°C, and a final elongation step of 5 min at 72°C. Sequencing was carried out with Illumina HiSeq2000 (2 × 101 bp reads), MiSeq (2 × 75 bp reads), or NextSeq (2 × 150 bp reads) instrument using v3 or v4 chemistry to reach an average spanning coverage of 20–30× for the mate-pair libraries and an average sequencing coverage of 20–30× for deep WGS libraries. After sequencing, the reads were aligned to the hg19 build of the human reference genome using the Illumina-provided alignment software ELAND (version 2) for all RPE-1-derived sequencing data, and using BWA for the cancer genomic data.

### Analysis of DNA sequencing data

Deletions, tandem duplications, inversion, inter-chromosomal SRs, and combinations thereof (complex SRs) were inferred using DELLY v0.0.11 (Rausch *et al*, 2012b). For inference of SRs in cell lines, we considered all DELLY-inferred SRs as “somatic” that were not present in 8-fold coverage WGS-sequenced genomes from 1,106 healthy individuals sampled by the 1,000 Genomes Project (1000GP, phase 2/3) (Genomes Project *et al*, 2012)—*that is,* specimens obtained from normal individuals with ancestry from diverse human populations (Genomes Project C *et al*, 2012). SRs in primary tumor samples were inferred by comparing tumor with paired normal samples from the same patient and additionally through subtracting calls corresponding to SRs detected in the 1000GP. SRs were considered as identical if their start and end coordinates differed by less than 5.0 kb (approximate insert size of a long-range paired-end library) and if their reciprocal overlap was larger than 50%. Variants that were present in the control samples were either true germ line variants or represented artifacts caused by misalignment of reads (e.g., due to inaccuracies in the hg19 human reference genome assembly). To consider a variant prediction as high confident, we further required at least four supporting read pairs with a minimum median mapping quality of 20 for each event to exclude false-positive predictions caused by random mapping of low-quality DNA reads. Tetraploidy in medulloblastoma samples was inferred by clustering tumor B-allele frequency (BAF) assignments together with WGS coverage ratios (tumor versus normal) for all inferred heterozygous SNP sites.

### Haplotype-based profiling of allelic imbalances

We profiled haplotype-specific allelic imbalances and copy numbers in samples with WGS data (> 25-fold sequence coverage). We inferred germ line SNPs using the variant caller freebayes (Garrison, 2012) (v0.9.15 and v0.9.20; default parameters, -i -X –u; https://github.com/ekg/freebayes) and only retained high quality bi-allelic SNPs (QUAL > 20) that overlapped SNPs from the 1000 Genomes Project (2014 release). Heterozygous sites were statistically phased using SHAPEIT2 (Delaneau *et al*, 2013) (v2; parameters: -window 10 Mb, -states 5008, -no-mcmc, overlap between chunks: 1 Mb) with a haplotype reference panel based on the 1000GP October 2014 release. Phased heterozygous SNPs were genotyped in tumor genomes (or clones with SRs) using freebayes (default parameters, -l -@). We excluded rare variants (MAF < 1%, Ensembl 75) and variants that were covered by less than four reads from further analysis to minimize errors in phase estimation and quantification of allelic imbalance. We segmented phased allelic ratios with the CBS algorithm (*R* package DNACopy; parameters: data.type=“logratio,” undo.splits=“sdundo,” undo.SD=1) in order to infer haplotype-specific allelic imbalances. We only kept segments that were defined by at least five markers. Haplotype-specific coverage was calculated at phased SNPs, and diagnostic haplotypes with allelic ratios of 2:0 and 2:1 were used to estimate total haplotype-specific copy numbers.

### PCR validations

Primers were constructed with our in-house primer design pipeline, based on primer3 software (http://biotools.umassmed.edu/bioapps/primer3_www.cgi), and purchased from Sigma. PCR experiments were performed as follows: 10 ng of genomic DNA was used with the SequalPrep Long PCR Kit (Invitrogen) in 20 μl volumes applying the following PCR conditions in a MJ Mini thermocycler (BioRad): 94°C for 3 min, followed by 10 cycles of 94°C for 10 s, 62°C for 30 s and 68°C for 6 min and 25 cycles of 94°C for 10 s, 60°C for 30 s, and 68°C for 7 min, followed by a final cycle of 72°C for 10 min. PCR products were analyzed on a 1% agarose gel stained with SYBR Safe Dye (Invitrogen).

### RNA libraries and sequencing

Total RNA was extracted from cells using the RNeasy mini kit (Qiagen). RNA quality control was performed using the 2100 Bioanalyzer platform (Agilent). A total of 500 ng total RNA was used as a starting material for the RNA sequencing libraries, which were prepared using the TruSeq strand-unspecific protocol with Ribo-Zero Gold (Illumina) and sequenced on the Illumina HiSeq 2000 platform with 2 × 51 cycles according to the manufacturer’s instructions. In order to minimize batch effects, samples were processed on a Beckman Biomek FX robot following the manufacturers’ instructions.

### RNA sequencing analyses

RNAseq reads were aligned to the reference built by bowtie based on Ensembl v. 62 exons, build GRCh37.p3. Gene-specific read counts were obtained with the Genomic Alignments package in R, using a strand-unspecific model together with the Ensembl transcript database. Differential expression was assessed with the R package DESeq.

### Statistical testing

For the analysis of non-random distributed samples, permutation testing was applied. The labels of the two groups switched, and the mean difference was computed 10,000 times and then compared to the observed difference. For the analysis of breakpoint clustering, a KS test was applied testing for statistically significant deviation from the null hypothesis of no breakpoint clustering. For the analysis of random distribution of SR types, multinomial testing against the null hypothesis “equal distribution of joins” was applied using the R package “EMT.”

### Fluorescence *in situ* hybridization

Cells were cultured in 225-cm flasks (Sigma Aldrich, CLS431082) up to a confluence of 95% and then treated with 100 ng/ml of colcemid (Sigma Aldrich, D1925) for 6 h. Cells were collected by mitotic shake-off and transferred to a freshly prepared hypotonic solution (KCl 0.55% (Merck-Milipore) and Na-citrate (Merck-Milipore) 1% mixed 1:1) following centrifugation. Cells were swollen in hypotonic solution and subsequently fixed by methanol–acetic acid mixture (3:1, Merck-Milipore). Fixed cells were dropped on glass slides prewarmed with steam, which were previously treated with methanol (Merck-Milipore). The slides were then dried, and the confluence and the quality of the spreads were checked under a microscope. For chromosome counts, cells were probed with PNA centromere probe coupled to Cy3 dye (Panagene) according to manufacturers’ instructions. DNA was stained with 0.2 g/ml Hoechst 33342 (Life Technologies).

### Microscopy

Imaging on most indirect immunofluorescence samples was performed at 25°C on a DeltaVision RT system (Applied Precision) with an Olympus IX71 microscope. This system was equipped with FITC, TRITC, and Cy5 filters (Chroma Technology), a plan-Apo 100× NA 1.4 and 60× NA 1.4 oil immersion objective (Olympus), a CoolSNAP HQ camera (Photometrics), a temperature controller (Precision Control), and Softworx software (Applied Precision).

### Live cell imaging

Cells were seeded into chambered cover glasses (LabTEK: Thermo Fisher Scientific), and the lids of the chambers were sealed with baysilone paste (Neolab). Approximately 30 min before imaging, culture medium was exchanged to prewarmed CO_2_-independent medium without phenol red, containing 20% FCS, 2 mM glutamine, and 100 mg/ml penicillin–streptomycin. Live cell microscopy was performed in 37°C microscope incubators (EMBL GP106) on a Zeiss 780 confocal microscope with a 63× PlanApochromat oil objective (NA 1.4, Carl Zeiss) and in-house temperature controller, controlled by the Zen 2010 Software. Six z stacks with 2.00-μm intervals were used for each position. Images were acquired with 5-min time resolution. When applicable, automated quantitative analysis of cells was pursued to monitor for mitotic progression in single cells. To this end, nuclei were detected in the H2B-mCherry channel and classified as previously described (Held *et al*, 2010; Walter *et al*, 2010) with an overall accuracy of > 90.0%. Cells were tracked with a constrained nearest-neighbor tracking procedure, and mitotic onset was detected as interphase–prophase or interphase–prometaphase transition. To reduce the effect of classification errors on phase length measurements, classification results were corrected using hidden Markov models (Held *et al*, 2010).

### Data deposition

Cell-line-based sequencing data (DNA and RNA based) are deposited at ENA, accession PRJEB8037, and patient sequencing data at EGA, accession EGAS00001000215.

## Supporting Information

**Appendix**

**Expanded View Figures PDF**

**Table EV1**

**Table EV2**

**Table EV3**

**Dataset EV1**

**Review Process File**
