# Supplementary material for: A cell-based model system links chromothripsis with hyperploidy
Source: Mol Syst Biol. 2015 Sep 28;11(9):828. doi: 10.15252/msb.20156505 (PMC4592670; doi:10.15252/msb.20156505)
Supplement: Supplementary file 1 [file msb0011-0828-sd1.pdf]

A cell-based model system links chromothripsis with hyperploidy

Balca R. Mardin[1], Alexandros P. Drainas[1], Sebastian M. Waszak[1], Joachim Weischenfeldt[1], Mayumi Isokane[2], Adrian M. Stütz[1], Benjamin Raeder[1], Theocharis Efthymiopoulos[1], Christopher Buccitelli[1], Maia Segura-Wang[1], Paul Northcott[3], Stefan M. Pfister[3], Peter Lichter[4], Jan Ellenberg[2], Jan O. Korbel[1]

Appendix

Appendix notes

Inference of Chromothripsis

In order to distinguish chromothripsis (CT) (one-off) events from DNA rearrangements occurring in a stepwise fashion, we investigated all cell lines and tumor samples described herein with previously established criteria[1, 2]. We performed manual curation of complex and clustered DNA rearrangements to enable CT inference (as well as the inference of breakage-fusion-bridge, BFBs) at high confidence.

1- Breakpoint clustering

CT generates highly clustered DNA breaks that may be followed by long tracts of intact DNA segments[3]. We performed statistical analysis for non-randomness of breakpoint distributions, under the assumption of an exponential distribution[1] (null hypothesis). For each sample and chromosome, a Kolmogorov-Smirnov (KS) test was used for testing against the null hypothesis. In all analyzed samples, the chromosomes that were predicted to have undergone CT displayed high significance.

2- Randomness of DNA rearrangement joins

In chromosomes undergoing CT, the shattered fragments are randomly stitched together according to the original model brought forward[3]. This implies that for each DNA break, the orientation of the two joined DNA fragment ends will be random. In order to test this, after filtering for high confident DNA rearrangement calls as described in the previous section, we performed a multinomial test (using the R package "EMT") for chromosomes presumed to undergo CT[1]. With this model we tested the observed distribution of rearrangement joins (tail to head, head to tail, head to head and tail to tail) against a "background model" of occurrence with equal probability of 0.25. In chromosomes inferred to undergo catastrophic DNA rearrangements, we did not observe a significant departure from equal probability of joins, consistent with the occurrence of CT[1].

3- Regularity of oscillating copy number segments

As "classical" outcome of CT, the resulting DNA fragments display oscillating copy

number states (with a magnitude of 1 in cases where only one chromosome is affected, and when CT is not followed by subsequent DNA rearrangements). As described recently, CT can be preceded by earlier genomic rearrangements, such as BFBs[2]. Since we also detected BFBs in conjunction with oscillating copy numbers (e.g. in BM175 and MB243) we chose to make use of the deductive approaches recently described by Li *et al.*[2] enabling investigation of the temporal ordering of different classes of complex structural rearrangements.

Namely we evaluated:

- *the copy number state distribution, i.e. the distribution of copy number states in a particular chromosome; if CT is not preceded or followed by any other sequential SRs, then the resulting DNA fragments will be distributed in 2 copy number states (for example, chromosomes exhibiting segments oscillating between copy number=2 and copy number=1).*
- *copy number step switches, i.e. the absolute value of the magnitude of change at each copy number switch when traversing a chromosome from lower to higher genomic coordinates; if CT occurs on an unrearranged chromosome, copy number switches resulting from CT will have a magnitude of one[2] (unless subsequent somatic DNA alterations, for example, whole genome duplications, occur after the CT event has happened).*
- *copy number jumps, i.e. the change in copy number between each pair of segment joints (SRs) detected by the DELLY tool[4] (head-to-head, tail-to-tail, head-to-tail, or tail-to-head); if CT occurs on a previously unrearranged chromosome copy number jumps will be of a magnitude of zero ('classical' CT) as all of the retained/rearranged segments exhibit the same copy number state[2] (notably, CT preceded by earlier SRs, for example BFBs, results in copy number jumps of a magnitude of one or greater).*

4- Interspersed loss and retention of heterozygosity

It was previously shown that many chromosomes undergoing CT show characteristic patterns of interspersed loss and retention of heterozygosity[3], an analysis that requires SNP genotype information and hence deep whole genome sequencing data. We performed tests for interspersed loss and retention of heterozygosity in two cases – BM175 and BM178 – the two clones for which ~25-fold coverage WGS data was available. Consistent with CT, patterns of interspersed loss and retention of heterozygosity were readily visible in BM178 (Appendix Fig. S8e), but more difficult to interpret for BM175 owing to the presence of other complex large-scale SRs (e.g. multiple BFBs).

5- Prevalence of rearrangements affecting a specific haplotype

According to the CT model DNA fragments resulting from chromosome shattering originate from a single parental haplotype[1]. Haplotype information can be retrieved via statistical phasing of bi-allelic single nucleotide polymorphisms using a haplotype reference panel[5, 6]. Haplotype-based allelic imbalance analysis of the deeply sequenced clones BM175 and BM178 indicated that the rearrangements map to a unique haplotype segments, in line with the occurrence of CT in both samples.

6- Ability to walk the derivative chromosome

Another consequence of CT being a one-off process is that derivative chromosomes forming upon chromosome shattering and erroneous fragment joining can be reconstructed, which form a coherent chain of segments whereby each retained segment occurs only once within that chain[1]. We applied this criterion for CT, also referred to as "ability to walk the derivative chromosome",[1] to BM178, since in that sample CT arose in the absence of other complex large-scale SRs affecting the same haplotype (with the reasoning that derivative chromosomes undergoing additional SRs prior to or after CT can retain certain segments more than once). Indeed, the SRs in BM178 detected on chromosome 12 and 22 form such a "walkable" chain, in keeping with CT. We additionally verified the SRs on chromosomes 12 and 22 by PCR (*i.e.*, 24 out of 30 tested SR junctions could be verified, 3 were inconclusive and the other 3 were negative).

7- Randomness of fragment order

With chromosome segments being randomly stitched together by DNA repair during CT, the relative positioning (i.e. ordering) of these segments along the rearranged derivative chromosome has been proposed to be approximately random[1]. We chose not to test for this criterion in the course of this study, however, in line with recent observations of departure from random reordering in the context of CT (implying spatial structure in the DNA repair process)[1, 2].

Appendix Figures

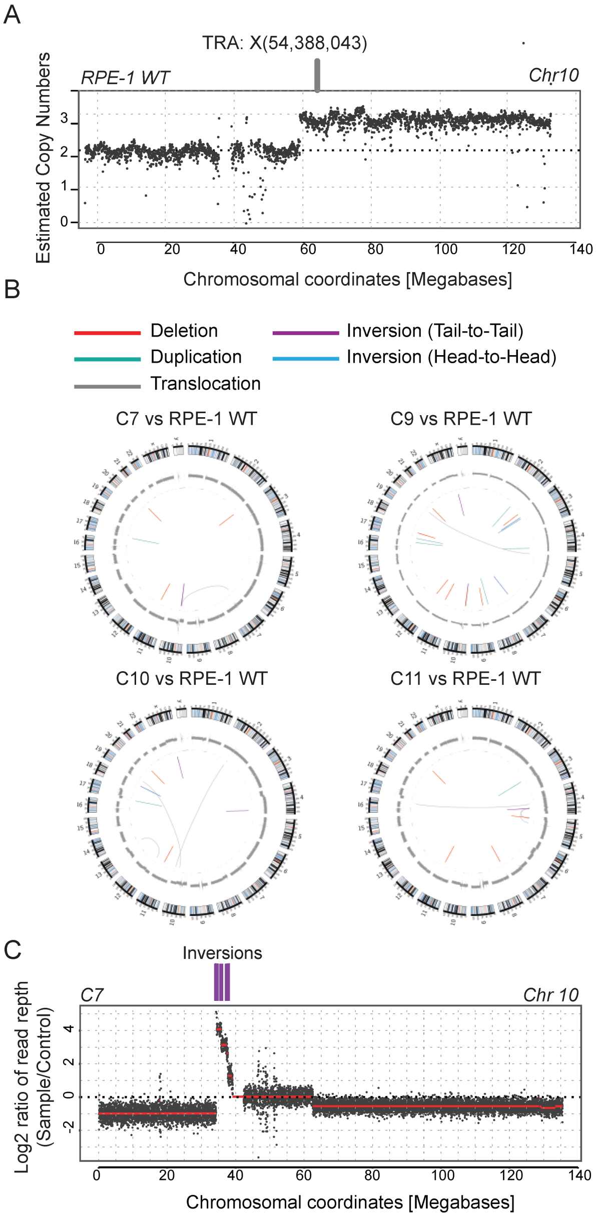

Appendix Figure S1- Analysis of RPE-1 cells using mate-pairs.

A- Confirmation of the previously reported translocation in RPE-1 WT cells. Raw read counts on chromosome 10 with estimated copy numbers are depicted. As previously reported by SKY karyotyping [7] we detected a translocation between chromosome 10 and chromosome X and a copy number increase in the long arm of chromosome 10 consistent with the extra copy of chromosome 10 being translocated to chromosome X.

B- Circos plots of 4 transformed RPE-1 cell lines provided to us from an earlier study[8] performed prior to the development of massively parallel sequencing technology. On the outer circle the chromosomal cytobands, and the coverage plot as compared to the hTERT-RPE (RPE-1 WT) are depicted. The connections between the breakpoints are color coded, as described in the legend.

C- Example SR pattern from clone C7. On chromosome 10 we have detected a step-wise increase in copy number around the centromeric region, which were demarcated by fold-back inversions, indicating the occurrence of BFBs on this chromosome.

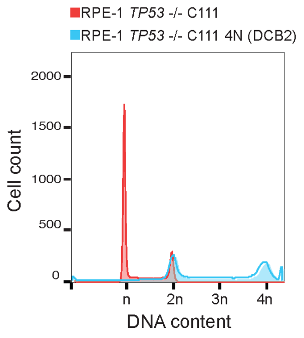

Appendix Figure S2 - Flow cytometry analysis of DNA content of DCB2.

The DCB2 cell line was created from the C111 clone by actin inhibition (DCB). Cells were fixed and stained with Hoechst and analysed by flow cytometry. The difference in DNA content of DCB2 relative to C111 demonstrates tetraploidy.

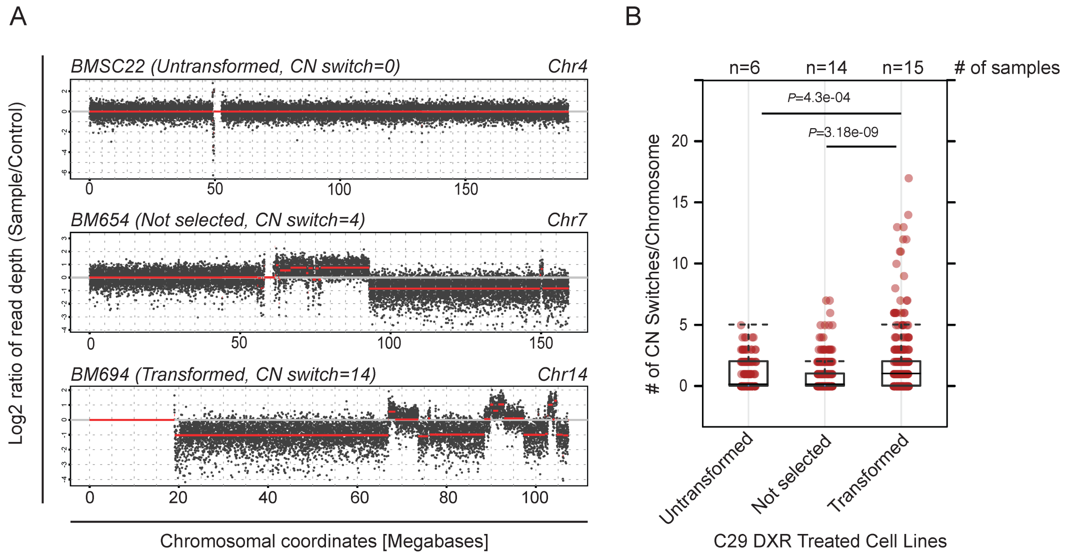

Appendix Figure S3- Requirement of soft agar selection for the enrichment of complex DNA rearrangements.

We subjected untransformed, non-selected and soft agar selected hyperploid cells, all of which were treated with DXR, to low coverage sequencing to detect chromosomal copy number switches.

A- Exemplary dead depth plots with circular binary segmentation are depicted in each case, with corresponding copy number (CN) switch counts. (Only copy number segments of ≥500 kb were taken into account in this analysis).

B- Copy number switches per chromosome are plotted for each group. Copy number switches ≥10 per chromosome, indicative for highly complex SRs (such as CT events), were observed only in the soft agar selected DXR treated samples. *P* values are derived from Welch two sample *t*-test following permutation analysis.

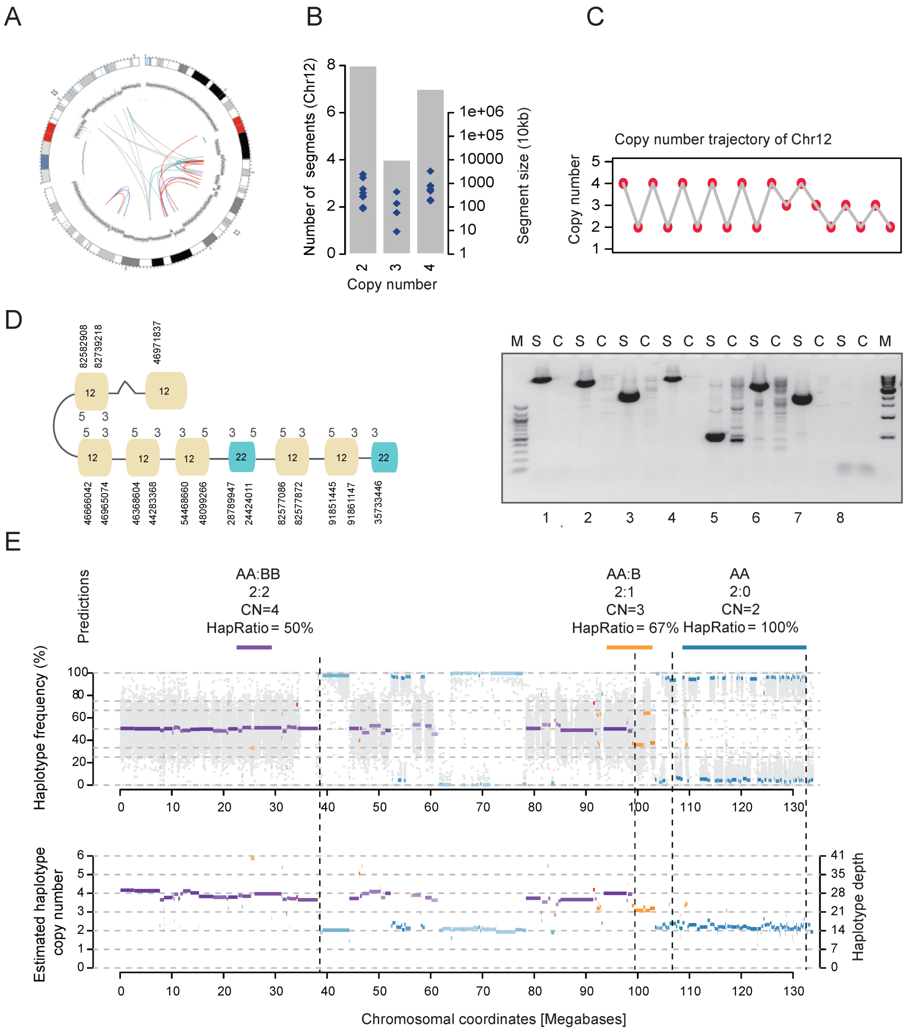

Appendix Figure S4- Further analysis of BM178 cell line.

As described in the Fig. 2a-e and based on the reasoning described below, in BM178 the walkable derivative chromosome consists of DNA segments from chromosomes 12 and 22, which we infer resulted from co-shattering by CT resulting in at least 48 breakpoints on chromosome 12. Breakpoint associated copy number jumps[2] were mostly at 0 in line with CT being the first event rearranging these chromosomes.

a- Circos plot depicting connections between chromosomes 12 and 22.

b- Segment copy number state distribution of chromosome 12. Blue diamonds represent the segment sizes.

c- Copy number trajectory of chromosome 12. It is important to note that in this and equivalent plots throughout this manuscript only segments with confidently ascertainable copy number are displayed within the copy number trajectory (this is, due to highly clustered breakpoints in CT, creating extremely short fragments[1, 2], and frequently preventing confident copy number state assignment).

d- Inference of a section of the derivative chromosome resulting from CT based on SR joins and SR validation by PCR. (C=control, S=sample, M=marker). 8 exemplary PCR results are shown, 7 of which confirm our SR predictions (by the presence of a detectable product in the sample and the absence of the product in the control).

e- Evidence for patterns of interspersed loss and retention of heterozygosity on chromosome 12 and CT occurring on one haplotype (another criterion that strongly supports the occurrence of CT). Inferred haplotype information is depicted in the above panels. To derive haplotype information we analysed copy number states and phased single nucleotide polymorphisms. For simplicity, we define the two haplotypes of each chromosome as A and B haplotypes. A disomic state, for example, is denoted as AB, and a tetrasomic state, for example, as AABB. The haplotype ratios are presented as frequency of A/B.

The copy number state of chromosome 12 in the parental cell line is 3, yet the estimated copy number states of the unrearranged segment (p arm of chromosome 12) as well as the segments with associated SRs are 4 with equal haplotypes (AABB). From the haplotype distribution we predict that this chromosome had 3 copies pre-CT (AAB). CT occurred on the "B" haplotype, which was most likely followed by a chromosome duplication event (presumably due to a chromosome segregation error) resulting in the cell acquiring both B haplotypes of chromosome 12 (the abundance of copy number step sizes of a magnitude of two further support this inference[2]).

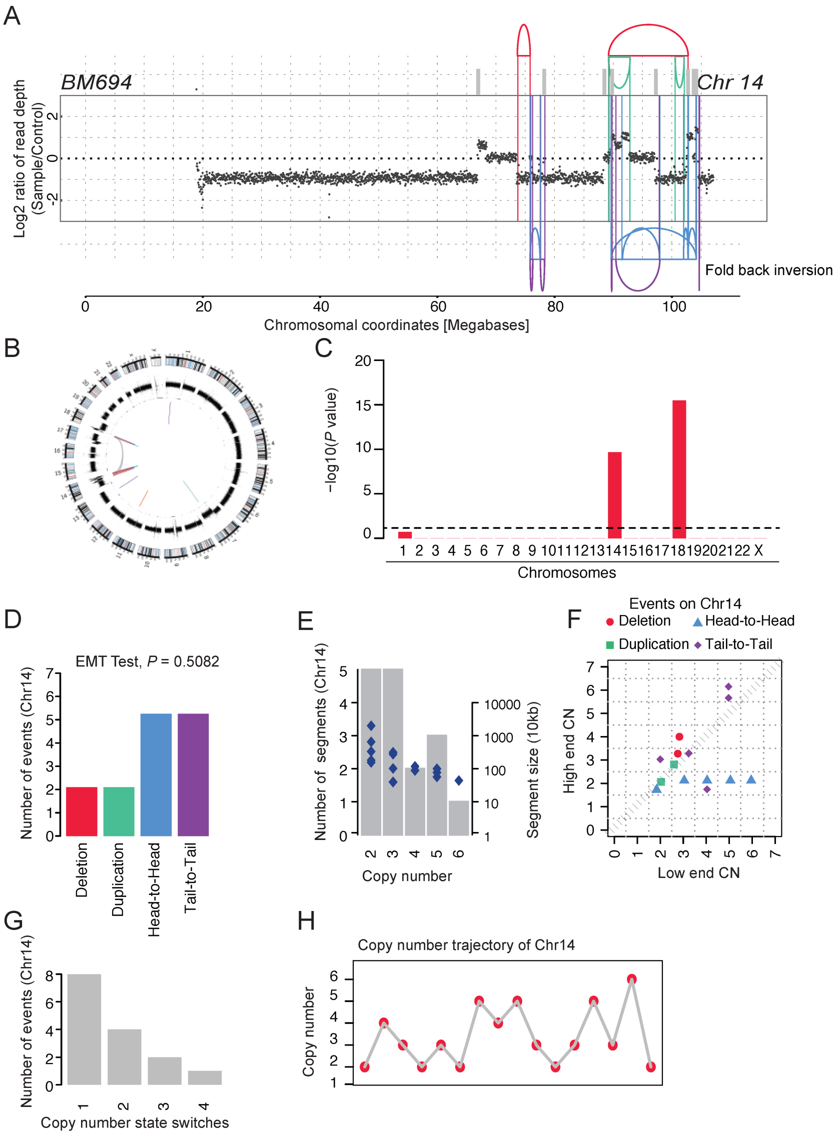

Appendix Figure S5- Analysis of BM694 cell line.

In BM694, derived from the sub-tetraploid C29 clone, CT occurred on chromosome 14, a chromosome highly connected to chromosome 18 with 9 high-confidence translocation calls made by the DELLY tool[4]. Similar to BM175, a stepwise increase of copy number segments followed by a sharp drop towards the chromosome end was observed, suggesting a preceding BFB cycle. As additional support for the BFB event, we observed a fold-back inversion on this chromosome and breakpoint associated copy number jumps of >0. Furthermore, readily visible oscillating copy number states bear the hallmarks of CT.

A- SR and oscillating copy number alteration patterns in BM694 based on mate-pair data. Altogether 37 breakpoints were detected on chromosome 14. SRs are color coded based on their orientation: red, deletion type (T-H); green, duplication type (H-

T); blue, head-to-head (H-H); purple, tail-to-tail (T-T) type; gray, inter-chromosomal. A fold-back inversion indicative of a BFB is highlighted.

B- Circos plot depicting depth of coverage and SRs at genome-wide scale. Note the connections between chromosomes 14 and 18.

C- Statistically significant deviation from null hypothesis of no rearrangement breakpoint clustering in BM694, consistent with the occurrence of CT[1, 2]. Y axis shows the -log10(*P*-value) of KS-test applied to each chromosome.

D- Randomness of joins for rearranged segments on chromosome 14, in line with the occurrence of CT[8]. (EMT test, $P = 0.7018$)

E- Segment copy number state distribution of chromosome 14. Blue diamonds denote segment sizes.

F- Copy number jump distribution between joined fragments of chromosome 14. Most points are off the diagonal, which indicates that CT occurred on a previously rearranged chromosome.

G- Copy number step distribution of rearrangements on chromosome 14. Copy number state oscillations, which frequently exhibit a copy number step size of a magnitude of one, indicate that CT was preceded by a BFB cycle.

H- Copy number trajectory of chromosome 14.

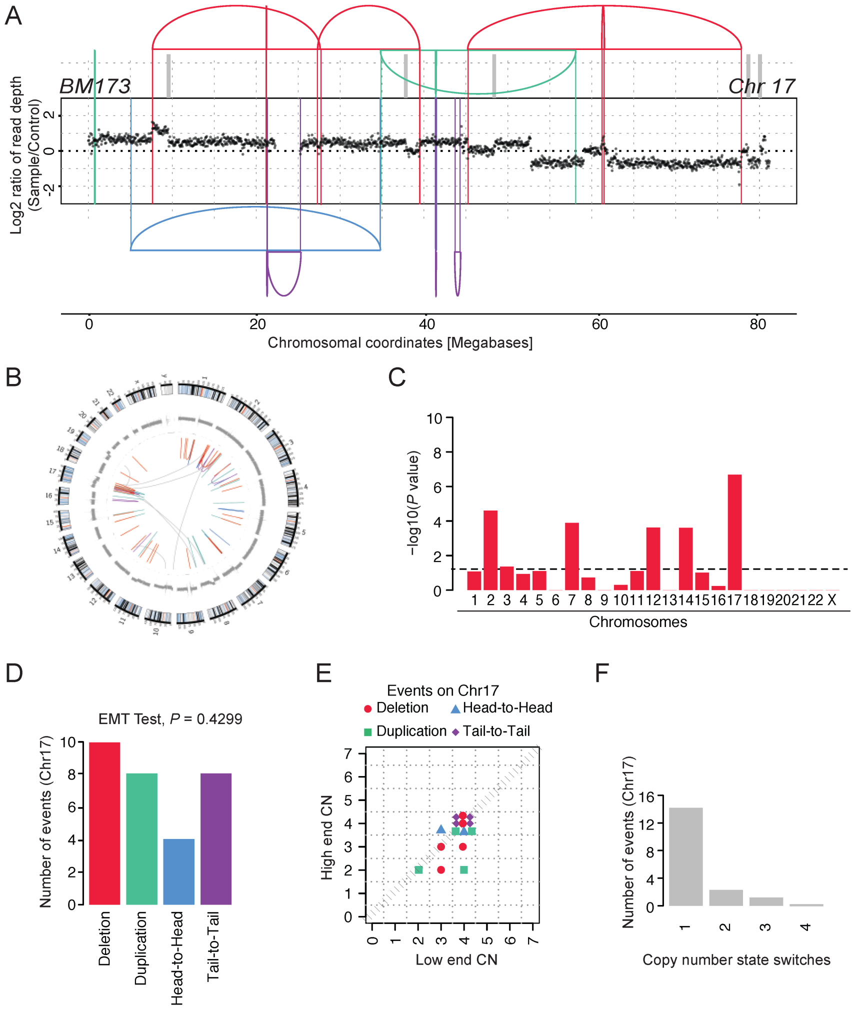

Appendix Figure S6- Analysis of BM173 cell line.

In BM173, derived from the sub-tetraploid C29 clone, CT occurred on chromosome 17. This chromosome shows typical oscillating copy number patterns bearing the hallmarks of a CT on a previously unrearranged chromosome. We observed that the shattered chromosome is connected to chromosomes 2 and 8, indicating the potential co-shattering of three chromosomes.

A- SR and oscillating copy number alteration patterns in BM173 based on mate-pair data. Altogether 30 breakpoints were detected on chromosome 17. SRs are color coded based on their orientation: red, deletion type (T-H); green, duplication type (H-T); blue, head-to-head (H-H); purple, tail-to-tail (T-T) type; gray, inter-chromosomal.

B- Circos plot depicting depth of coverage and SRs at genome-wide scale. Note the connections from chromosome 17 to chromosomes 2 and 8.

C- Statistically significant deviation from null hypothesis of no rearrangement breakpoint clustering in BM173, consistent with the occurrence of CT[1, 2]. Y axis

shows the -log10(*P*-value) of KS-test applied to each chromosome. Note that the lowest p value is observed on chromosome 17.

D- Randomness of joins for rearranged segments on chromosome 17, in line with the occurrence of CT[8]. (EMT test, $P = 0.4299$)

E- Copy number jump distribution between joined fragments of chromosome 17. Diagonal points indicate that CT occurred on a previously unrearranged chromosome.

F- Copy number step distribution of rearrangements on chromosome 17. Copy number state oscillations, mostly exhibit a copy number step size of a magnitude of one follow the typical patterns of CT.

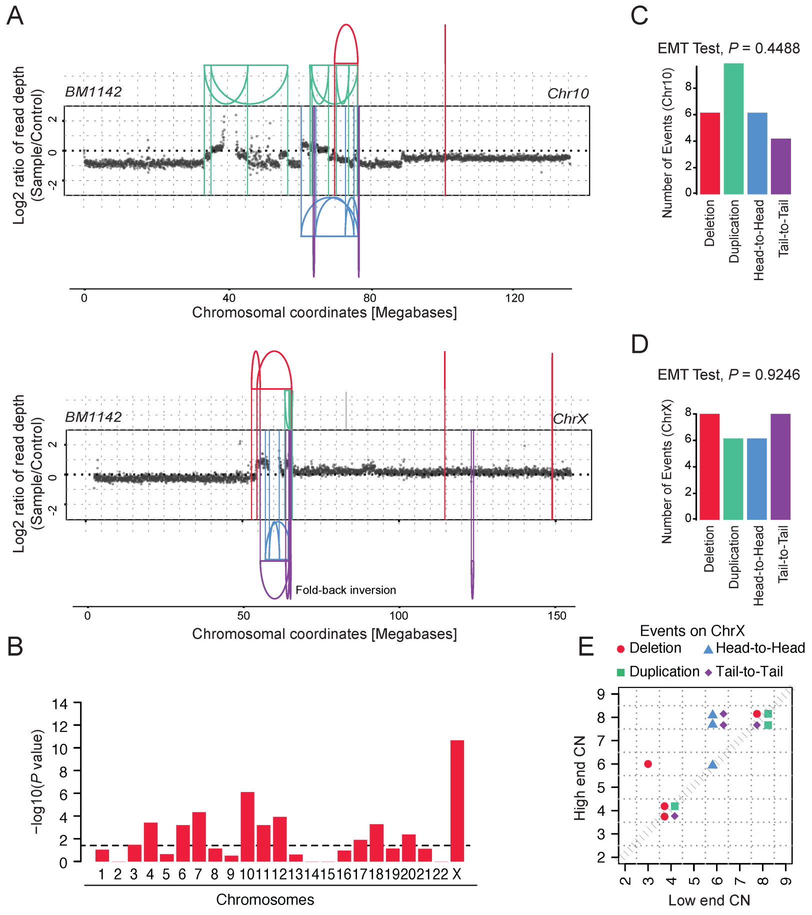

Appendix Figure S7- Analysis of BM1142 cell line.

For BM1142 derived from hyperploid C29 cell line our analysis revealed 26 and 28 clustered SR breakpoints resulting from CT on chromosomes 10 and X, respectively. We failed to detect any inter-chromosomal SRs between these two chromosomes; hence two independent CT events may have occurred in this cell line. In one case, a fold-back inversion stemming from a BFB event was observed on the rearranged chromosome.

A- DNA alteration patterns of chromosomes 10 and X in BM1142 based on mate-pair data, with highly oscillating copy number profiles consistent with the occurrence of CT. SRs are color-coded: red, deletion type (T-H); green, duplication type (H-T); blue, head-to-head (H-H); purple, tail-to-tail (T-T) type; gray, inter-chromosomal. A fold-back inversion, consistent with BFB occurrence, is highlighted.

B- Statistically significant deviation from null hypothesis of no breakpoint clustering in BM1142 (y-axis depicts -log10(*P*-value) for KS-test applied to each chromosome), in line with the occurrence of CT.

C- Randomness of DNA fragment joins[1] for BM1142 chromosome 10 (EMT test, *P* = 0.4488), in keeping with the occurrence of CT. *P*-value derived from multinomial testing against null hypothesis ”equal distribution of joins”.

D- Randomness of DNA fragment joins[1] for BM1142 chromosome X (EMT test, *P* = 0.9246).

E- Copy number jump distribution[2] between joined fragments of chromosome X in BM1142 (displayed for segments with confident copy number ascertainment), indicating that CT occurred on a previously rearranged chromosome with at least 1 BFB cycle.

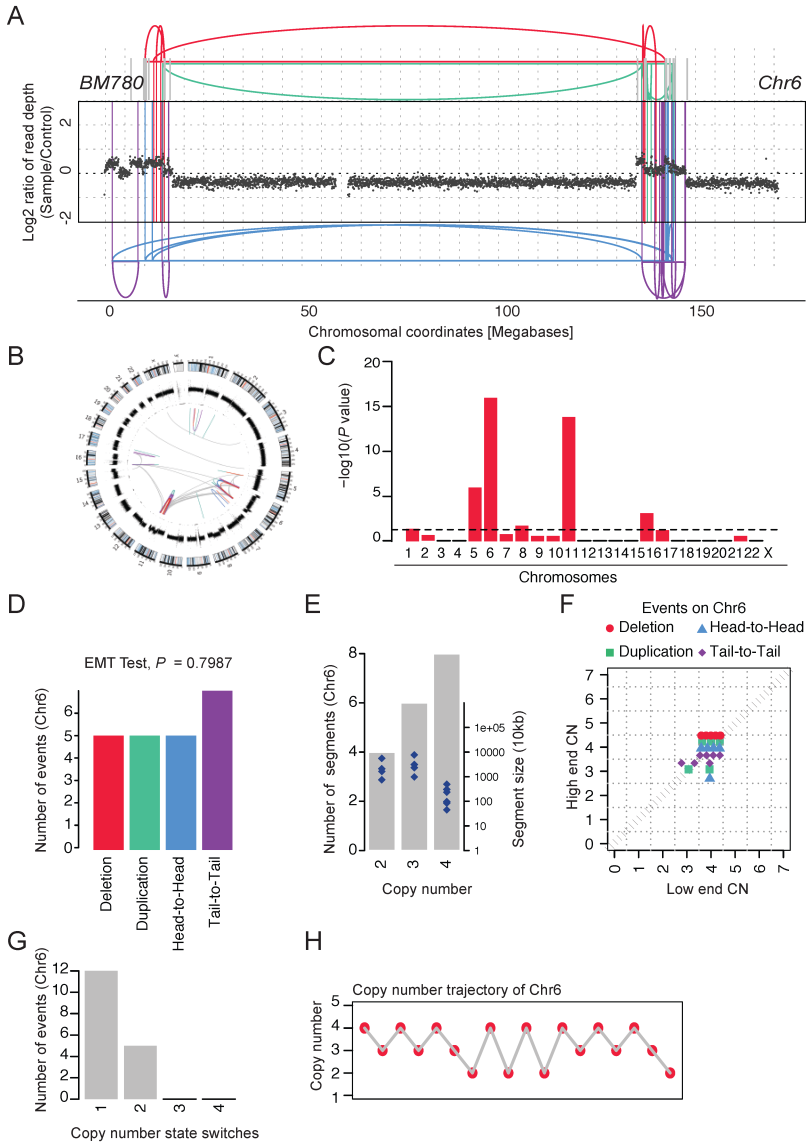

Appendix Figure S8- Analysis of BM780 cell line.

In BM780, derived from the DCB2 clone, CT was observed on chromosome 6, with at least 56 breakpoints. This chromosome is highly connected to chromosome 11 (with 12 high confidence translocation calls), indicating yet another co-shattering event. In this sample the breakpoints are highly clustered on two segments of chromosome 6 (approximately 0-17Mb and 135-147Mb). The retained segments are connected to each other by randomly oriented DNA segment joins, consistent with the CT model. On chromosome 6, the vast majority of copy number jumps[2] associated with breakpoints were of a magnitude of 0, which indicates that CT occurred on a previously unrearranged chromosome (“classical” CT).

A- SR and copy number alteration graphs of chromosome 6 based on mate-pairs. SRs are color coded based on their orientation: red, deletion type (T-H); green, duplication type (H-T); blue, head-to-head (H-H); purple, tail-to-tail (T-T) type; gray, inter-chromosomal.

B- Circos plot depicting depth-of-coverage and genome-wide SRs. Note the abundant connections between chromosomes 6 and 11.

C- Statistically significant deviation from null hypothesis of no rearrangement breakpoint clustering in BM694. Y axis shows the -log10 (*P*-value) of KS-test applied to each chromosome. *P*-values observed for chromosomes 6 and 11 are consistent with the occurrence of CT.

D- Randomness of fragment joins, in keeping with the occurrence of CT on chromosome 6. (EMT test, $P = 0.7967$)

E- Segment copy number state distribution of chromosome 6. Blue diamonds denote segment sizes.

F- Copy number jump distribution between joined fragments of chromosome 6.

G- Copy number step distribution of rearrangements on chromosome 6.

H- Copy number trajectory of chromosome 6.

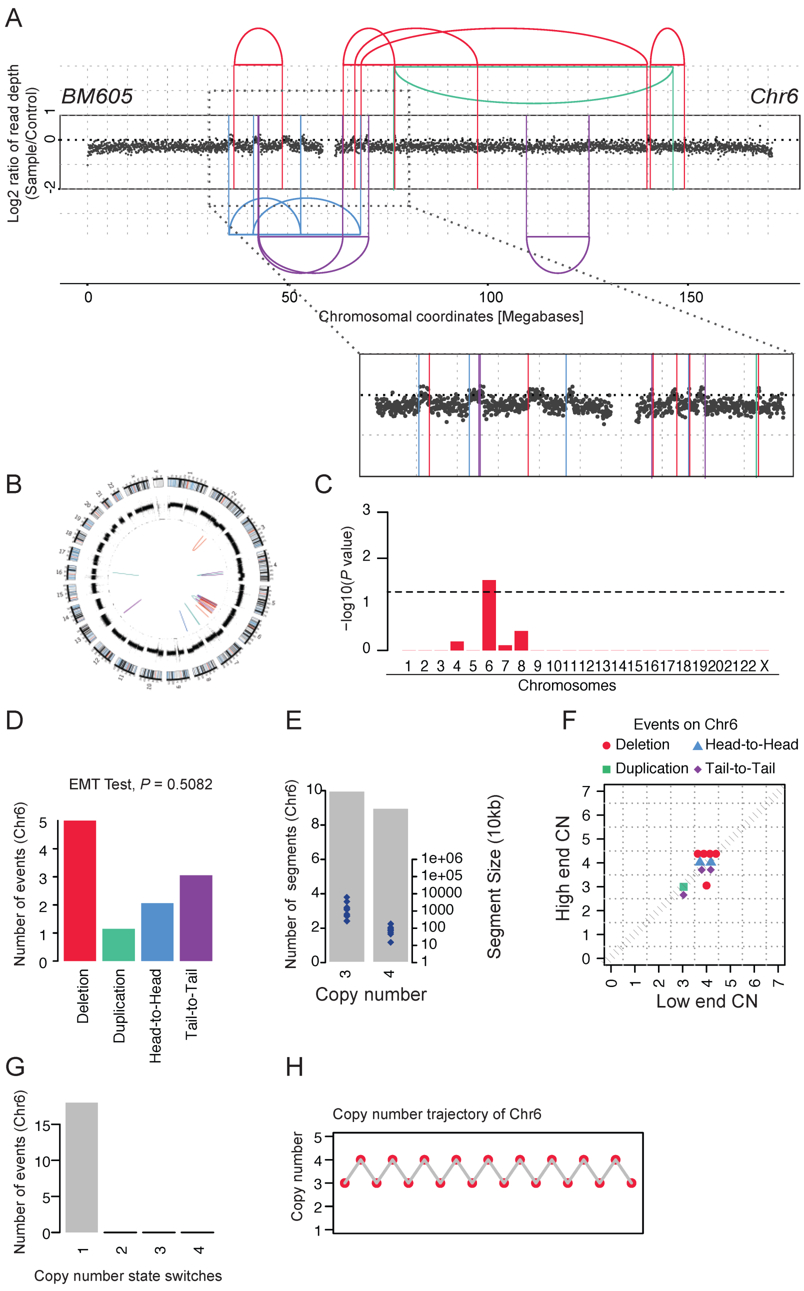

Appendix Figure S9- Analysis of BM605 cell line.

In BM605, derived from tetraploid C111 clone, CT was observed on chromosome 6. The oscillating pattern of copy numbers and the copy number jumps of 0 resulted from a ‘classical’ CT event with 22 breakpoints, which occurred on a previously unrearranged chromosome.

A- SR and copy number alteration pattern of chromosome 6 based on mate-pairs with a zoom-in of the region covering 30-80Mbs. SRs are color coded based on their orientation: red, deletion type (T-H); green, duplication type (H-T); blue, head-to-head (H-H); purple, tail-to-tail (T-T) type; gray, inter-chromosomal

B- Circos plot depicting depth-of-coverage and SRs at genome-wide scale.

C- Statistically significant deviation from null hypothesis of no rearrangement breakpoint clustering in BM694, in line with the occurrence of CT on chromosome 6. The y axis shows the -log10 (*P*-value) of KS-test applied to each chromosome.

D- Randomness of SR joins on chromosome 6, additionally supporting the occurrence of CT. (EMT test, $P = 0.5082$)

E- Segment copy number state distribution of chromosome 6. Blue diamonds denote segment sizes.

F- Copy number jump distribution between joined fragments of chromosome 6, indicating that CT occurred on a previously unrearranged chromosome[2].

G- Copy number step distribution of rearrangements on chromosome 6, indicating that no rearrangements occurred after the CT event.

h- Copy number trajectory of chromosome 6.

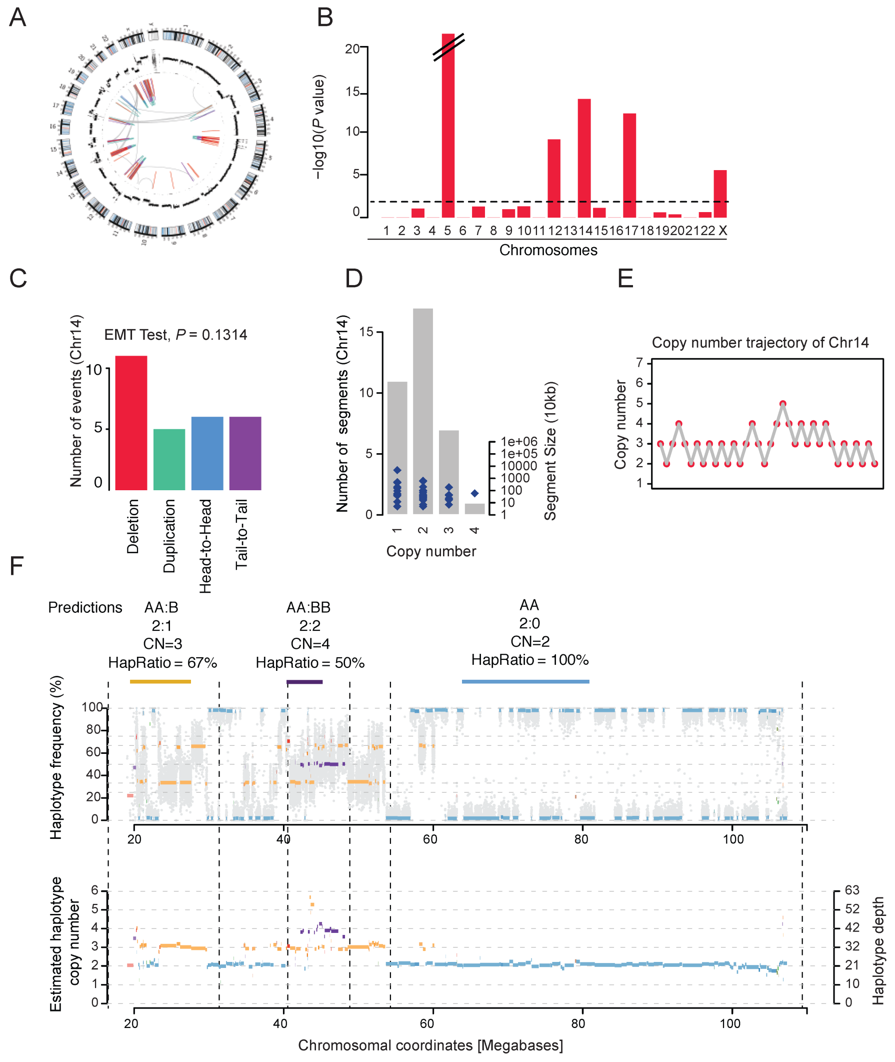

Appendix Figure S10- Analysis of MB34

We analysed CT in MB34, a tetraploid tumor sample carrying a somatic *TP53* mutation, with chromosome 14 exhibiting highly oscillating copy number states resulting from CT. Based on the observation that copy number switches when traversing the chromosome from lower to higher coordinates are always of a magnitude of one[2], we deduce that whole genome duplication preceded CT. We cannot, however, formally exclude a second scenario whereby CT of one chromosome was followed by whole genome duplication, and subsequently followed by the loss of one of the two resulting copies of the CT-derived marker chromosome.

A- Circos plot depicting copy number states and genome-wide SRs.

B- Statistically significant deviation from null hypothesis of no rearrangement breakpoint clustering in BM694, consistent with CT. Y axis shows the -log10 (*P*-value) of KS-test applied to each chromosome. Chromosome 5 showed extreme high-level amplifications, indicting the presence of CT-derived double minute

chromosomes similar to other SHH-MB samples undergoing CT that we described previously[9].

C- Randomness of SR joins on chromosome 14, consistent with CT (EMT test, P = 0.1314).

D- Segment copy number state distribution of chromosome 14. Blue diamonds denote segment sizes.

E- Copy number trajectory of chromosome 14.

F- Analysis of phased SNPs and copy numbers to assess the temporal ordering of events. Inferred haplotype segments are depicted in the above panel.

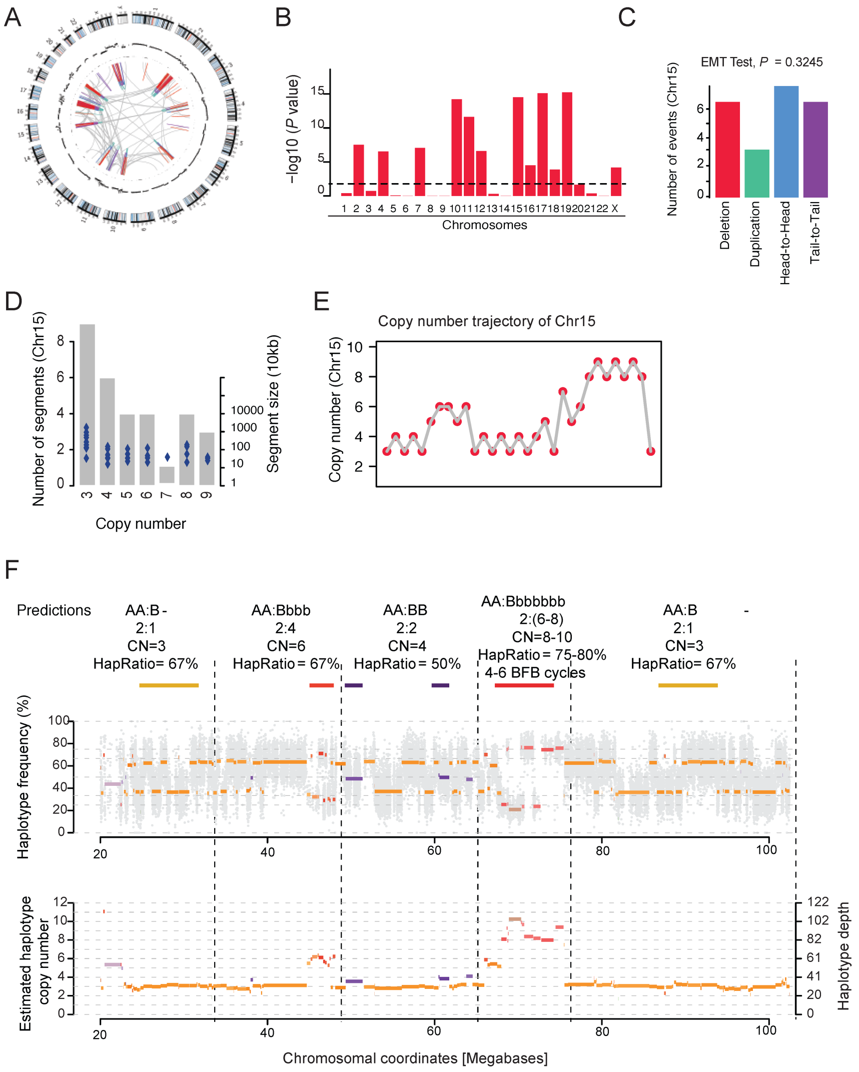

Appendix Figure S11- Analysis of MB243

We detected CT on chromosome 15 from MB243, a tetraploid tumor sample from a SHH-MB patient carrying a germline stop-gain mutation in *TP53*. A step-wise increase in copy number segments before a sharp drop at the chromosome end is indicative for BFB cycles. Furthermore, highly oscillating copy number alterations, embedded within the BFB pattern, bear the hallmarks of CT. In addition to chromosome 15, further chromosomes are heavily rearranged in this sample and connected with chromosome 15, indicating co-shattering. We chose to evaluate chromosome 15 due to its robustly inferred copy number segments. Together with the presented evidence in Figure 3, our data indicate that CT occurred on a previously rearranged chromosome 15. Furthermore, haplotype profiles for this sample are highly consistent with tetraploidy preceding CT. The majority of chromosome 15 exists in 3 intact copies of haplotype ratio 67%(AAB). All the

remaining rearrangements were inferred to occur on the 4th haplotype ("B", *i.e.* the highly rearranged haplotype), including 4-6 predicted BFB cycles and a CT event.

A- Circos plot depicting SRs and copy number at genome-wide scale.

B- Statistically significant deviation from null hypothesis of no rearrangement breakpoint clustering in MB243, consistent with CT occurring. Y axis shows the -log10 (*P*-value) of KS-test applied to each chromosome.

C- Randomness of SRs types joining DNA fragments on chromosome 15, further supporting the occurrence of CT. (EMT test, *P* = 0.3245)

D- Segment copy number state distribution of chromosome 8. Blue diamonds denote segment sizes.

E- Copy number trajectory of chromosome 15.

F- Analysis of phased SNPs and copy numbers to assess the temporal ordering of events. Inferred haplotype segments are depicted in the above panel.

A

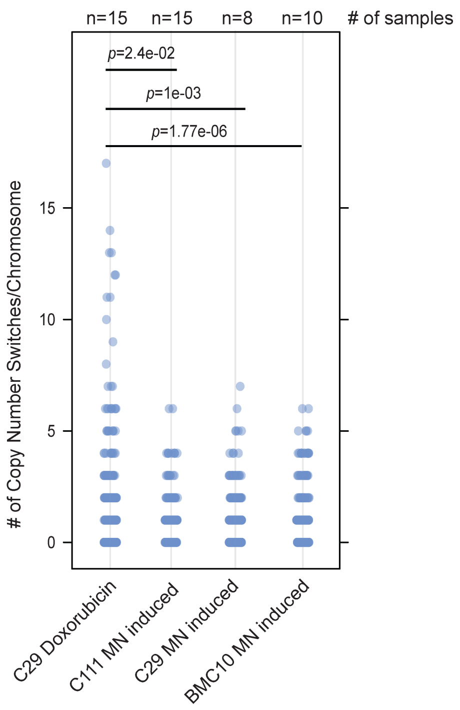

B

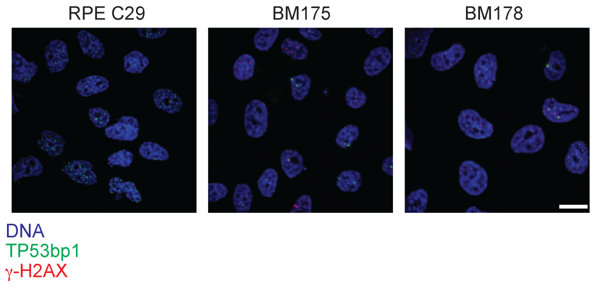

Appendix Figure S12

A- A set of RPE-1 cells treated with micronuclei (MN) inducing chemicals (STLC, nocodazole and Hydroxyurea) were subjected to soft agar selection and subsequently to low-pass whole genome sequencing. Copy number switches per chromosome were plotted and compared to the hyperploid C29 cell line treated with doxorubicin. In MN induced cell lines, comparatively fewer copy number switches were observed, indicating that in this context global DNA double strand breaks induced by doxorubicin are more efficient in generating complex DNA rearrangements.

B- Lack of MN in chromothripsis cell lines. The BM175 and BM178 chromothripsis cell lines generated by our approach, as well as their parental control (C29 hyperploid cell line), were fixed and stained using those antibodies indicated in the legend. Nuclei were counterstained by Hoechst.

Appendix References

1. Korbel, J.O. & Campbell, P.J. Criteria for inference of chromothripsis in cancer genomes. *Cell* **152**, 1226-1236 (2013).
2. Li, Y. et al. Constitutional and somatic rearrangement of chromosome 21 in acute lymphoblastic leukaemia. *Nature* **508**, 98-102 (2014).
3. Stephens, P.J. et al. Massive genomic rearrangement acquired in a single catastrophic event during cancer development. *Cell* **144**, 27-40 (2011).
4. Rausch, T. et al. DELLY: structural variant discovery by integrated paired-end and split-read analysis. *Bioinformatics* **28**, i333-i339 (2012).
5. Delaneau, O., Marchini, J. & Zagury, J.F. A linear complexity phasing method for thousands of genomes. *Nature methods* **9**, 179-181 (2012).
6. Delaneau, O., Zagury, J.F. & Marchini, J. Improved whole-chromosome phasing for disease and population genetic studies. *Nature methods* **10**, 5-6 (2013).
7. Janssen, A., van der Burg, M., Szuhai, K., Kops, G.J. & Medema, R.H. Chromosome segregation errors as a cause of DNA damage and structural chromosome aberrations. *Science* **333**, 1895-1898 (2011).
8. Riches, A. et al. Neoplastic transformation and cytogenetic changes after Gamma irradiation of human epithelial cells expressing telomerase. *Radiation research* **155**, 222-229 (2001).
9. Rausch, T. et al. Genome sequencing of pediatric medulloblastoma links catastrophic DNA rearrangements with TP53 mutations. *Cell* **148**, 59-71 (2012).
