# Supplementary material for: A cell-based model system links chromothripsis with hyperploidy
Source: Mol Syst Biol. 2015 Sep 28;11(9):828. doi: 10.15252/msb.20156505 (PMC4592670; doi:10.15252/msb.20156505)
Supplement: Supplementary file 2 [file msb0011-0828-sd2.pdf]

Expanded View Figures

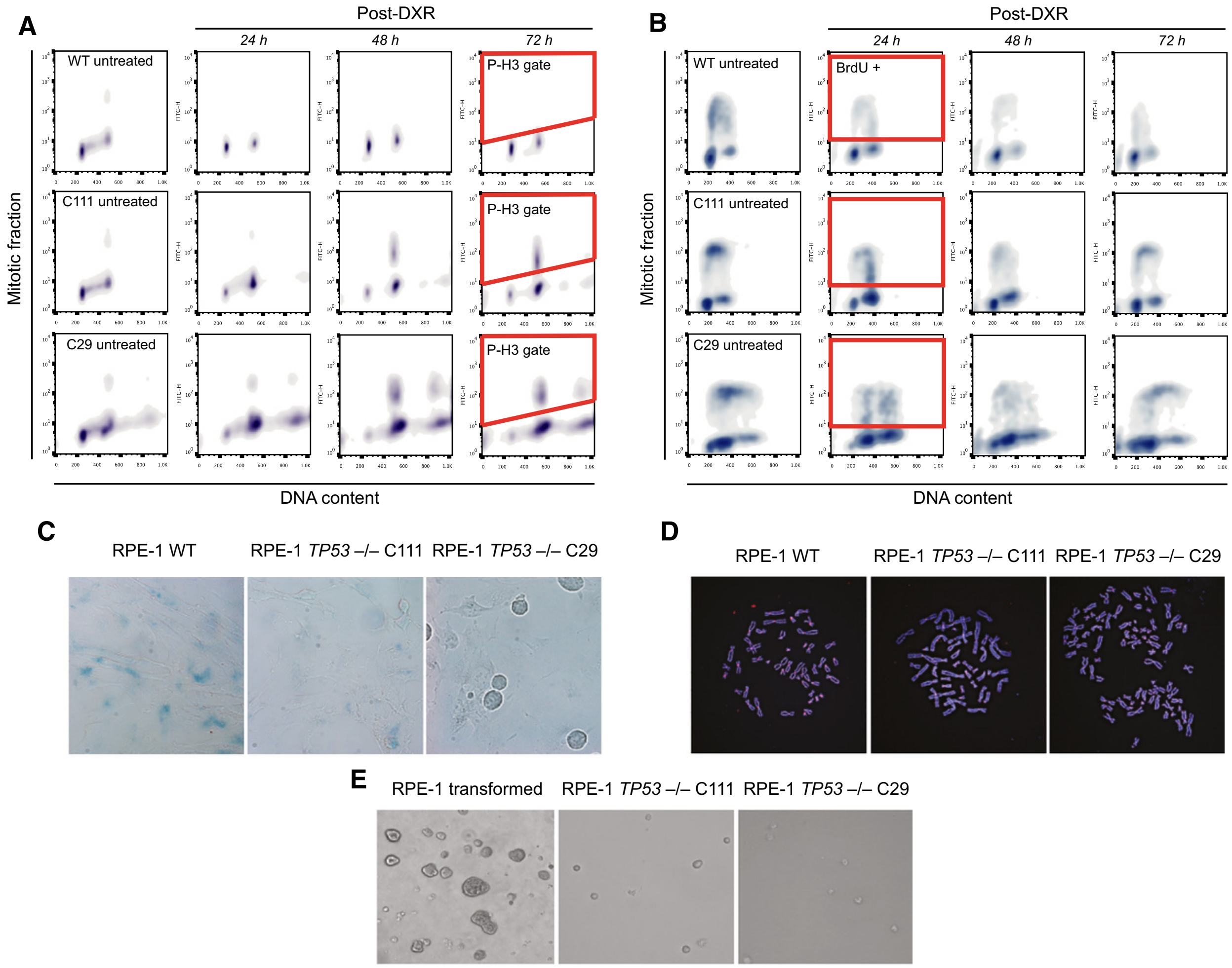

Figure EV1. Characteristics of the RPE-1 *TP53*$^{-/-}$ cell lines.

Assays for loss of function of p53 in RPE-1 WT and the *TP53*$^{-/-}$ cell lines, C29 and C111. Cells were challenged with doxorubicin (DXR, 1.5 μM) for 1 h and then released into fresh media.

- A The cells were released into medium containing 100 ng/ml nocodazole. Since p53 is a mediator of the DNA damage response in G2/M transition, cells with functional p53 are expected to be arrested in G2 due to massively damaged DNA (Bunz *et al*, 1998). When the DNA damage response is lost due to lack of p53 function, cells can enter mitosis where they are trapped with nocodazole. After 24, 48, and 72 h of incubation, cells were collected and stained with phosphohistone H3 antibody, a marker for mitotic cells. Cells were then analyzed by flow cytometry for their DNA and mitotic content. The gating for mitotic cells positive for P-H3 is exemplified in red after 72 h post-doxorubicin.
- B The cells were released into fresh medium. Since p53 also functions during G1/S transition, cells with functional p53 are expected to be arrest in G1 while the cells lacking p53 function continue cycling. After 24, 48, and 72 h of incubation, cells were pulsed for 1 h with BrdU in order to detect ongoing DNA replication. Cells were then analyzed by flow cytometry for their DNA and S phase content. The gating for mitotic cells positive for BrdU-FITC is exemplified in red after 24 h post-DXR. Please note the decrease in BrdU-positive cells in RPE-1 WT as well as the increase in G1 cell populations. In stark contrast, *TP53*$^{-/-}$ cells have ongoing DNA replication even after massive DNA damage.
- C Detection of senescent cells through measurement of β-galactosidase activity at pH 6, reflecting a known characteristic of senescent cells not found in dividing, quiescent or immortal cells. Cells were released into fresh media. After 72 h, the activity of β-galactosidase was assessed. Experiments were done in triplicate, and exemplary images are shown. Note that the RPE-1 WT cells were stained positive for β-galactosidase indicating senescence. In contrast, very few cells in *TP53*$^{-/-}$ cell lines were stained positive. Moreover, we frequently found dividing cells indicating a cycling population only in cells with non-functional p53.
- D Exemplary images of the metaphase spreads of the RPE-1 WT and the *TP53*$^{-/-}$ cell lines. Cells were fixed and stained with the pan-centromere probe for probing the centromeric regions of all chromosomes, and Hoechst to mark chromosomes. Experiments were done in triplicate. The mean count distribution is plotted in Fig 1C.
- E Exemplary images of RPE-1 cell lines after soft agar assay. Transformed RPE-1 cells as well as the two *TP53*$^{-/-}$ cell lines were incubated in soft agar for 25 days and imaged afterwards. Experiments were done in triplicate. The transformed cells were able to form colonies in agar, whereas the untransformed C29 and the C111 clones were not.

 © 2015 The Authors

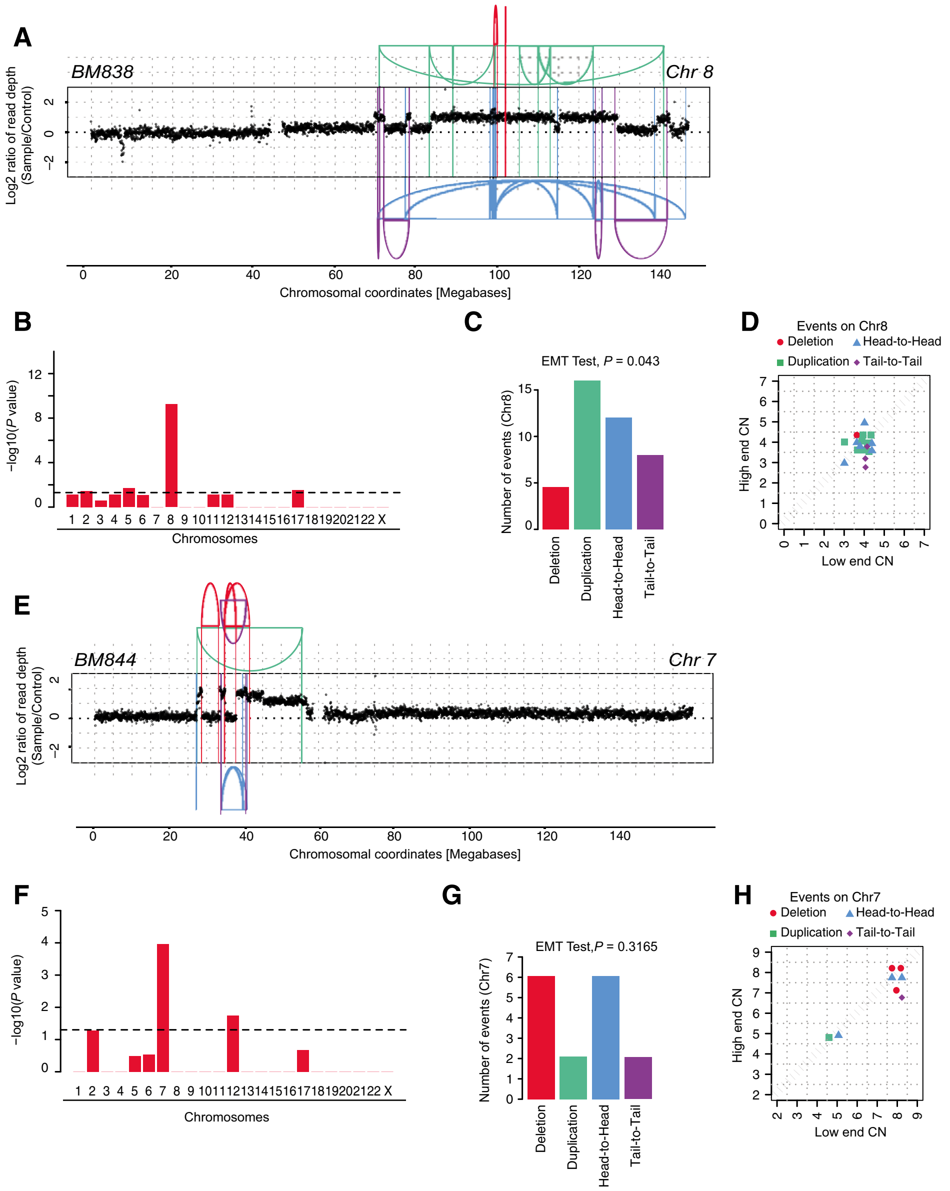

Figure EV2. Analysis of chromothripsis cell lines identified post-Zeocin treatment and agar selection.

A In BM838, derived from hyperploid C29 clone, chromothripsis was observed on chromosome 8. The oscillating pattern of copy numbers and mostly copy number jumps of 0 resulted from a "classical" chromothripsis event with 40 breakpoints, which occurred on a previously unrearranged chromosome. SRs are color-coded based on their orientation: red, deletion type (T-H); green, duplication type (H-T); blue, head-to-head (H-H) type; purple, tail-to-tail (T-T) type.

B Statistically significant deviation from null hypothesis of no rearrangement breakpoint clustering in BM838, in line with the occurrence of chromothripsis on chromosome 8. The *y*-axis shows the -$\log_{10}$(*P*-value) of KS test applied to each chromosome.

C Randomness of SR joins on chromosome 8 (EMT test, *P* = 0.043). In this sample, we found an apparent tendency of rearrangements to be slightly biased toward duplication-type rearrangements. In the context of the classical pattern of oscillating copy numbers with mostly zero copy number jumps and the strong genome-wide concentration of rearrangements to only this chromosome arm, we did not regard this apparent tendency as evidence against the occurrence of chromothripsis.

D Copy number jump distribution between joined fragments of chromosome 8, indicating that chromothripsis occurred on a previously unrearranged chromosome (Li *et al*, 2014).

E In BM844 derived from hyperploid clone C29, we detect chromothripsis on chromosome 7 with 16 breakpoints. A stepwise increase in copy number segments followed by a sharp drop toward the chromosome end was additionally observed, suggesting occurrence of a BFB cycle. SRs are color-coded based on their orientation: red, deletion type (T-H); green, duplication type (H-T); blue, head-to-head (H-H) type; purple, tail-to-tail (T-T) type; gray, inter-chromosomal.

F Statistically significant deviation from null hypothesis of no rearrangement breakpoint clustering in BM844, in line with the occurrence of chromothripsis on chromosome 7. The *y*-axis shows the -$\log_{10}$(*P*-value) of KS test applied to each chromosome.

G Randomness of SR joins on chromosome 7 (EMT test, *P* = 0.3165) further supporting evidence for chromothripsis.

H Copy number jump distribution between joined fragments of chromosome 7. The high CN step distribution might suggest that a number of duplications occurred following chromothripsis, presumably involving 1-2 BFB cycles.

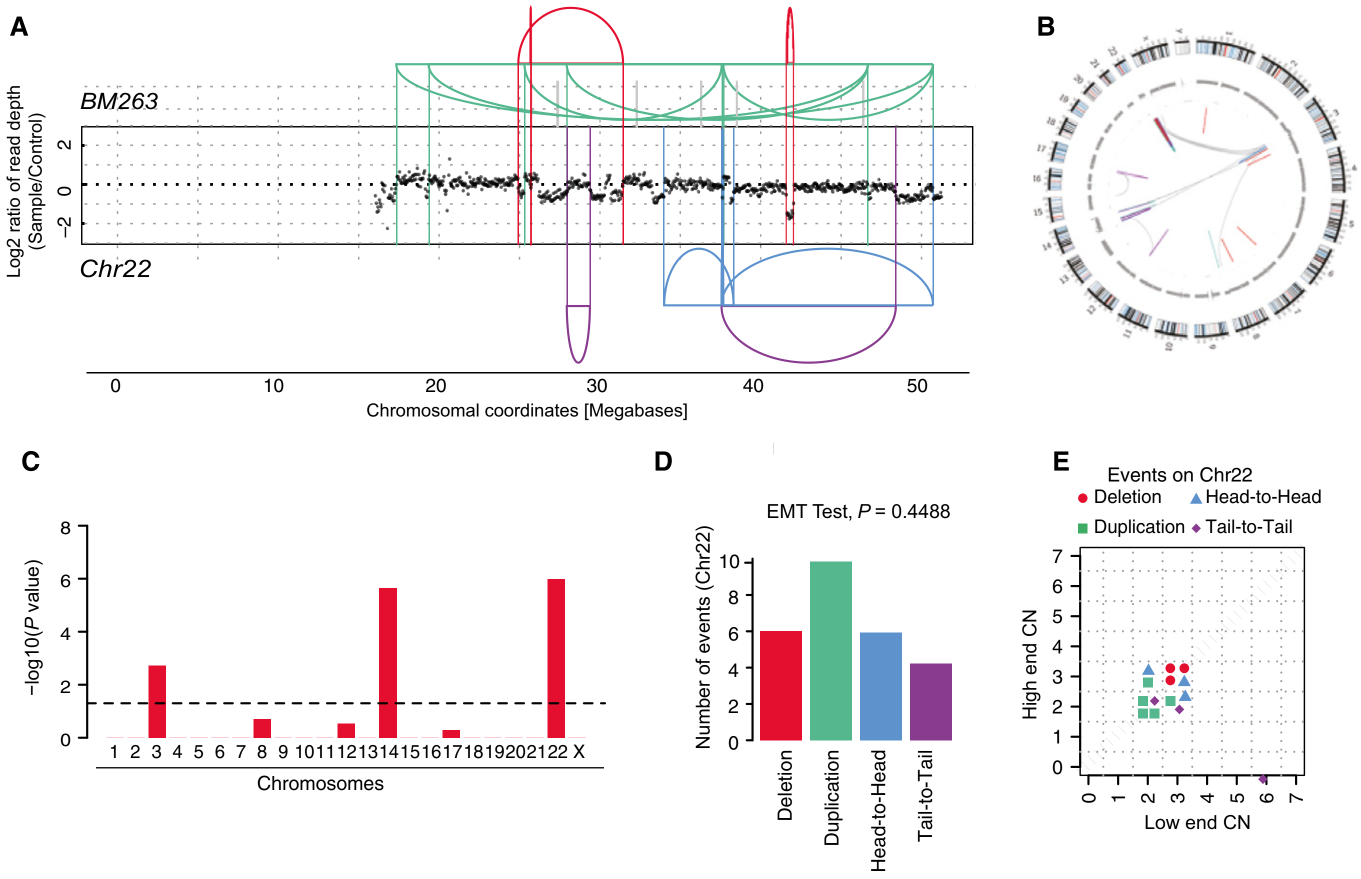

Figure EV3. Further evidence for chromothripsis in TRF2-depleted cells.

A DNA alteration patterns of chromosome 22 in BM263 based on mate-pair data. Highly oscillating copy number profiles are consistent with the occurrence of chromothripsis. SRs are color-coded: red, deletion type (T-H); green, duplication type (H-T); blue, head-to-head (H-H) type; purple, tail-to-tail (T-T) type; gray, inter-chromosomal.

B Circos plot depicting inter- and intra-chromosomal connections in BM263.

C Statistically significant deviation from null hypothesis of no breakpoint clustering in BM263 (y-axis depicts -$\log_{10}$(P-value) for KS test applied to each chromosome), in line with the occurrence of chromothripsis.

D Randomness of DNA fragment joins for BM263, additionally supporting the occurrence of chromothripsis. P-value derived from multinomial testing against null hypothesis "equal distribution of joins."

E Copy number jump distribution between joined fragments of chromosomes undergoing chromothripsis (displayed for segments with confident copy number ascertainment). On chromosome 22 in BM263, chromothripsis occurred most likely in isolation.

 © 2015 The Authors

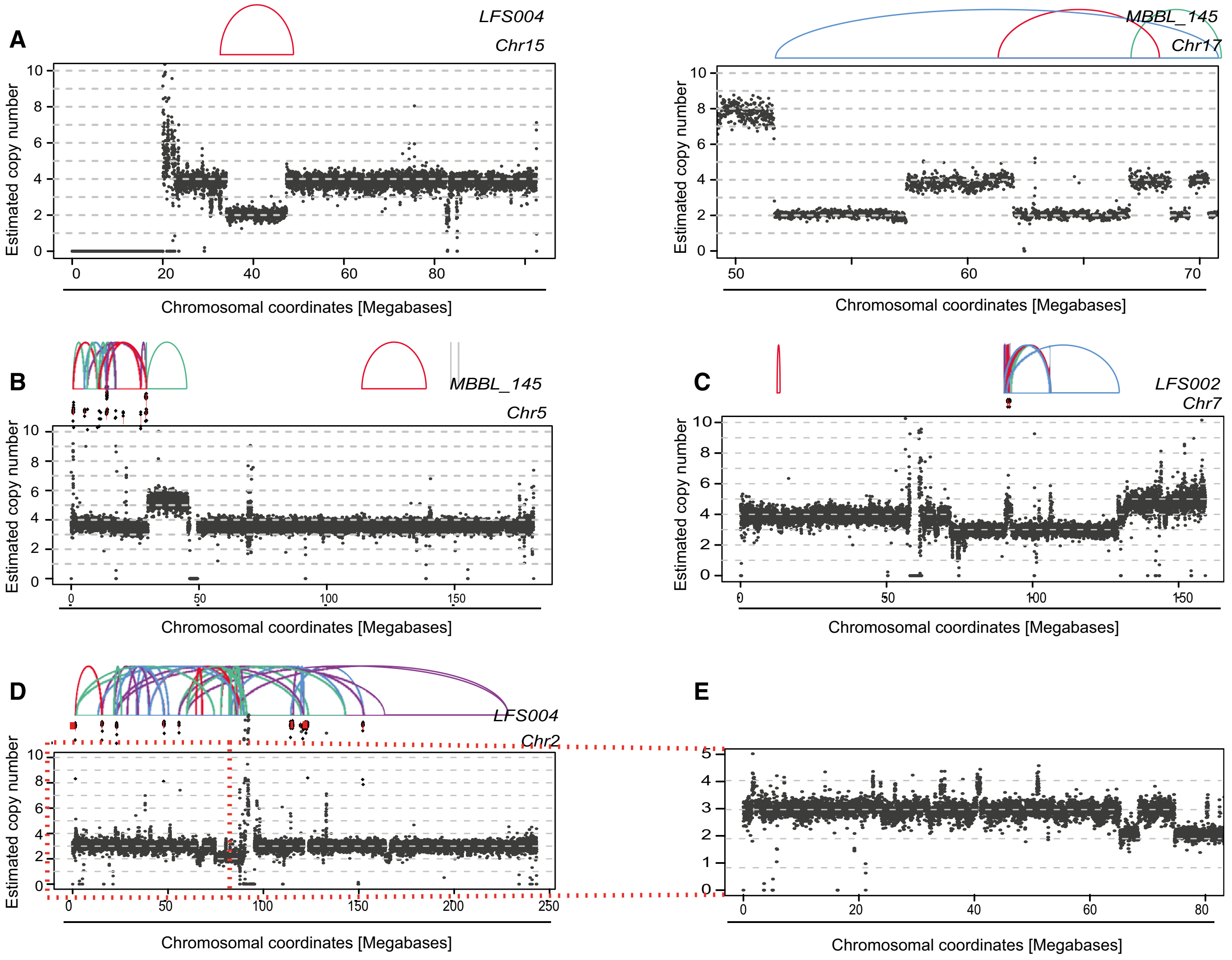

Figure EV4. Evidence for tetraploidy being an initiating event in SHH-MBs.

The plots display read depth from raw read counts with estimated copy numbers together with SR graphs. SRs are color-coded as indicated in Fig EV3 earlier.

A Two exemplary SR patterns, underlying presumably sequential SRs, exhibiting copy number state switches of 2 (indicating that these sequential SRs arose before tetraploidy).

B Estimated copy number of MB145, chromosome 5 together with SRs. Double minute chromosomes resulting from chromothripsis are indicated off-scale.

C Estimated copy number of LFS02, chromosome 7 together with SRs. (Double minute chromosomes resulting from chromothripsis indicated off-scale).

D Estimated copy number of LFS04, chromosome 2 together with SRs. (Double minute chromosomes resulting from chromothripsis indicated off-scale). The region with numerous copy number switches of magnitude of 1 is highlighted and further examined in (E).

E Zoom-in of chromosome 2, 0–80-Mb region. (Note the copy number switches of magnitude of 1).

Data information: These three cases (B–D) all show examples of tumors bearing the hallmarks of chromothripsis with double minute formation (Stephens *et al*, 2011; Rausch *et al*, 2012a). Except the massively amplified double minute chromosomes (which typically undergo repeated duplication after chromothripsis has occurred (Rausch *et al*, 2012a; Stephens *et al*, 2011)), all SRs were associated with copy number segment switches of 1, based on which we predict that tetraploidy (hyperploidy) preceded chromothripsis.

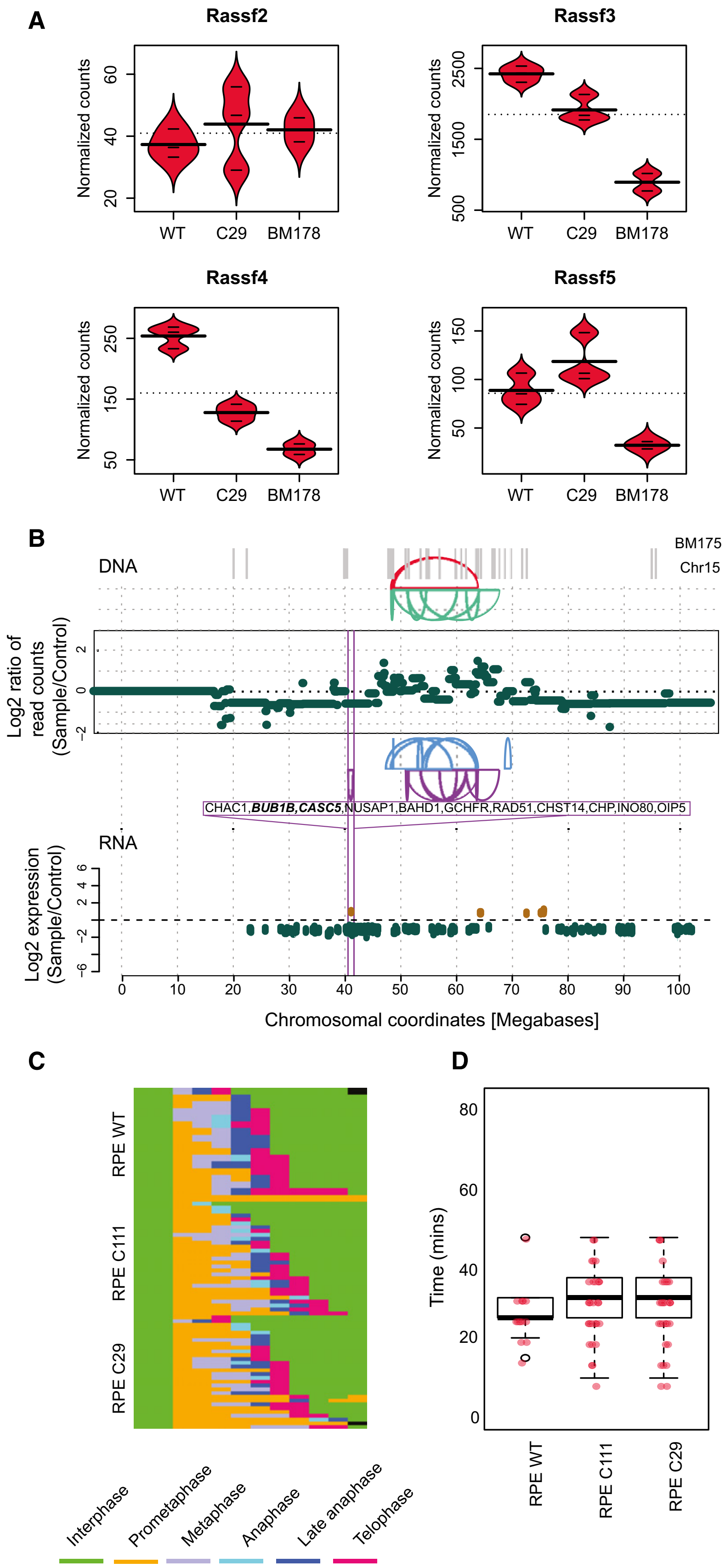

Figure EV5. Transcriptional consequences of chromothripsis.

A Transcriptional regulation of RASSF2/3/4/5. Bean plots from normalized counts of each mRNA are shown for RPE-WT, RPE-C29, and BM178. Horizontal lines represent values from individual samples, whereas thick horizontal lines indicate average values. Experiments were done in triplicates. Except for RASSF2, which exhibited very low read counts in our cell lines, all other members showed significant downregulation in BM178 ($P_{adj} < 0.1$).

B Significantly differentially expressed genes on chromosome 15 of BM175 (compared to the parental cell line C29) at an FDR of 10%. Log_2-fold changes of each gene are shown. In the above panel, copy number segments and SRs affecting chromosome 15 are depicted. Genes significantly affected by this SR are highlighted in green (downregulation) and brown (upregulation).

C Individual tracks of mitotic cells that were imaged and analyzed automatically by CellCognition (www.cellcognition.org). Each mitotic class is color-coded as indicated in the legend ($n^{WT} = 15$, $n^{C111} = 31$, $n^{C29} = 33$).

D Distribution of timing of mitotic progression (prometaphase, metaphase and anaphase in minutes) in RPE-1 WT and RPE-1 *TP53*$^{-/-}$ cell lines derived from Fig EV5C. We did not detect any significant changes between WT and *TP53*$^{-/-}$ cell lines. Box-and-whiskers plots: boxes show the upper and lower quartiles (25–75%) with a line at the median, whiskers extend from the 10 to the 90 percentile and transparent dots correspond to the outliers. Red dots represent individual samples.

 © 2015 The Authors
