# Supplementary material for: A cell-based model system links chromothripsis with hyperploidy
Source: Mol Syst Biol. 2015 Sep 28;11(9):828. doi: 10.15252/msb.20156505 (PMC4592670; doi:10.15252/msb.20156505)
Supplement: Supplementary file 3 [file msb0011-0828-sd3.docx]

### Table EV1

Sequence statistics for cell lines sequenced by low-pass WGS

| Parental Clone | Sample ID | Mapped read count | Coverage |
| --- | --- | --- | --- |
| C111 | BM673 | 2757095 | 0.038 |
| C111 | BM674 | 3405356 | 0.047 |
| C111 | BM675 | 2610111 | 0.036 |
| C111 | BM676 | 3472852 | 0.048 |
| C111 | BM766 | 2877943 | 0.039 |
| C111 | BM767 | 4084681 | 0.056 |
| C111 | BM768 | 3382286 | 0.046 |
| C111 | BM769 | 3361669 | 0.046 |
| C111 | BM770 | 3633568 | 0.050 |
| C111 | BM771 | 3291862 | 0.045 |
| C111 | BM772 | 3591420 | 0.049 |
| C111 | BM773 | 3614402 | 0.050 |
| C111 | BM774 | 3611990 | 0.050 |
| C111 | BM775 | 4339151 | 0.060 |
| C111 | BM776 | 3426482 | 0.047 |
| C111 | BM777 | 3682261 | 0.051 |
| C111 | BM778 | 4860130 | 0.067 |
| C111  C111  C111  C111  C111  C111  C111  C111  C111  C111  C111  C111  C111  C111  C111  C111  C111  C111  C111  C111  C111  C111  C111  C111 | BM779  BM1122  BM1123  BM1124  BM1125  BM1126  BM1127  BM1128  BM1129  BM1130  BM1131  BM1132  BM1133  BM1134  BM1135  BM1144  BM1145  BM1146  BM1147  BM1148  BM1149  BM1150  BM1151  BM1152 | 2635958  4613311  4668612  5203507  5698208  4110137  4606346  4256252  5131656  4948932  3872398  4024477  4639379  3833998  4750231  3485068  3104086  4031598  4608315  4774851  4878650  4210254  3693835  4118072 | 0.036  0.06  0.066  0.07  0.08  0.059  0.065  0.060  0.072  0.070  0.055  0.057  0.066  0.054  0.067  0.049  0.044  0.057  0.065  0.067  0.069  0.059  0.052  0.058 |
| C29 | BM173 | 3826816 | 0.054 |
| C29 | BM175 | 2956440 | 0.042 |
| C29 | BM178 | 2981012 | 0.042 |
| C29 | BM677 | 3225464 | 0.044 |
| C29 | BM678 | 2809047 | 0.039 |
| C29 | BM679 | 3501598 | 0.048 |
| C29 | BM680 | 3009412 | 0.041 |
| C29 | BM681 | 3545752 | 0.049 |
| C29 | BM682 | 2877320 | 0.039 |
| C29 | BM683 | 2832878 | 0.039 |
| C29 | BM684 | 3384943 | 0.047 |
| C29 | BM694 | 3671790 | 0.051 |
| C29 | BM695 | 3295285 | 0.045 |
| C29  C29  C29  C29  C29  C29  C29  C29  C29  C29  C29  C29  C29  C29  C29  C29  C29  C29  C29  C29  C29  C29  C29 | BM696  BM1136  BM1137  BM1138  BM1139  BM1141  BM1142  BM1143  BM1153  BM1154  BM1155  BM1156  BM1157  BM1158  BM1159  BM1160  BM1161  BM1162  BM1163  BM1164  BM1165  BM1166  BM1167 | 3746845  4778137  4549844  4832868  4988994  4511049  3095318  3004723  4470968  4540043  4096720  3858064  4375039  4029167  4115570  4471199  4018173  3737873  4171730  3834091  4208279  4096551  3151112 | 0.052  0.068  0.065  0.068  0.070  0.064  0.043  0.042  0.063  0.064  0.058  0.054  0.062  0.057  0.058  0.063  0.057  0.053  0.059  0.054  0.059  0.058  0.044 |
| DCB2 | BM597 | 5318398 | 0.075 |
| DCB2 | BM601 | 5296802 | 0.075 |
| DCB2 | BM605 | 3605336 | 0.051 |
| DCB2 | BM606 | 3970703 | 0.056 |
| DCB2 | BM610 | 4396837 | 0.062 |
| DCB2 | BM615 | 4064317 | 0.057 |
| DCB2 | BM619 | 3933359 | 0.055 |
| DCB2 | BM622 | 4929836 | 0.070 |
| DCB2 | BM625 | 4051764 | 0.057 |
| DCB2 | BM630 | 3920815 | 0.055 |
| DCB2 | BM634 | 3801777 | 0.054 |
| DCB2 | BM638 | 4232751 | 0.060 |
| DCB2 | BM642 | 3895891 | 0.055 |
| DCB2 | BM645 | 3638228 | 0.051 |
| DCB2 | BM647 | 2706051 | 0.038 |
| DCB2 | BM651 | 4742077 | 0.067 |
| DCB2 | BM780 | 3277537 | 0.046 |
| DCB2 | BM781 | 3067153 | 0.043 |
| DCB2 | BM782 | 2162614 | 0.030 |
| DCB2 | BM783 | 2085109 | 0.029 |
| DCB2 | BM784 | 3245220 | 0.046 |
| DCB2 | BM785 | 4194113 | 0.059 |
| DCB2  C29(not transformed)  C29(not transformed)  C29(not transformed)  C29(not transformed)  C29(not transformed)  C29(not transformed)  C29(not agar selected)  C29(not agar selected)  C29(not agar selected)  C29(not agar selected)  C29(not agar selected)  C29(not agar selected)  C29(not agar selected)  C29(not agar selected)  C29(not agar selected)  C29(not agar selected)  C29(not agar selected)  C29(not agar selected)  C29(not agar selected)  C29(not agar selected) | BM786  BMSCR22  BMSCR23  BMSCR24  BMSCR25  BMSCR26  BMSCR27  BM954  BM955  BM956  BM957  BM958  BM961  BM962  BM963  BM964  BM965  BM969  BM970  BM971  BM972 | 4173967  5162912  5215626  5128784  10578081  11344760  11206208  3770490  3869308  3882490  3645472  4013068  3402504  3804359  3147465  3784939  3286274  3958557  3241076  3962737  3270641 | 0.059  0.156  0.158  0.155  0.317  0.38  0.36  0.052  0.053  0.053  0.050  0.055  0.047  0.052  0.043  0.052  0.045  0.054  0.045  0.055  0.045 |
