# Supplementary material for: A cell-based model system links chromothripsis with hyperploidy
Source: Mol Syst Biol. 2015 Sep 28;11(9):828. doi: 10.15252/msb.20156505 (PMC4592670; doi:10.15252/msb.20156505)
Supplement: Supplementary file 4 [file msb0011-0828-sd4.docx]

### Table EV2

Sequence statistics for cell lines sequenced with mate-pairs

| Sample ID | Treatment | Read count (MP) | Sequencing coverage | Spanning coverage |
| --- | --- | --- | --- | --- |
| RPE WT |  | 27557062 | 0.91 | 23.49 |
| RPE C29 |  | 27551002 | 0.91 | 23.27 |
| RPE C111 |  | 22847232 | 0.81 | 18.76 |
| RPE DCB2 |  | 24610258 | 0.87 | 16.75 |
| RPE C7 |  | 28399526 | 0.93 | 22.78 |
| RPE C9 |  | 56829630 | 1.87 | 48.29 |
| RPE C10 |  | 28690248 | 0.94 | 22.90 |
| RPE C11 |  | 29666004 | 0.97 | 26.51 |
| BM673 | Doxorubicin | 32119542 | 1.12 | 24.49 |
| BM674 | Doxorubicin | 28834164 | 1.02 | 24.89 |
| BM675 | Doxorubicin | 30408756 | 1.07 | 27.82 |
| BM676 | Doxorubicin | 23544504 | 0.83 | 21.71 |
| BM766 | Doxorubicin | 32977444 | 1.16 | 24.15 |
| BM767 | Doxorubicin | 29923598 | 1.06 | 23.37 |
| BM768 | Doxorubicin | 34804232 | 1.23 | 26.03 |
| BM769 | Doxorubicin | 27650754 | 0.98 | 22.75 |
| BM770 | Doxorubicin | 23394850 | 0.83 | 20.28 |
| BM771 | Doxorubicin | 28756330 | 1.02 | 23.41 |
| BM772 | Doxorubicin | 27625640 | 0.98 | 23.01 |
| BM773 | Doxorubicin | 29793292 | 1.05 | 21.48 |
| BM774 | Doxorubicin | 24489128 | 0.86 | 16.46 |
| BM775 | Doxorubicin | 30874778 | 1.09 | 20.65 |
| BM776 | Doxorubicin | 23986544 | 0.85 | 16.38 |
| BM777 | Doxorubicin | 31576434 | 1.11 | 21.79 |
| BM779 | Doxorubicin | 27044610 | 0.95 | 19.64 |
| BM173 | Doxorubicin | 46651782 | 1.65 | 39.97 |
| BM175 | Doxorubicin | 38623522 | 0.69 | 34.04 |
| BM178 | Doxorubicin | 35251424 | 0.63 | 29.86 |
| BM678 | Doxorubicin | 27630068 | 0.97 | 20.95 |
| BM694 | Doxorubicin | 32186416 | 1.14 | 25.21 |
| BM696 | Doxorubicin | 35708222 | 1.56 | 30.95 |
| BM597 | Doxorubicin | 26225596 | 0.93 | 23.19 |
| BM601 | Doxorubicin | 25097432 | 0.89 | 22.24 |
| BM605 | Doxorubicin | 36011764 | 1.27 | 27.02 |
| BM606 | Doxorubicin | 29121134 | 1.03 | 22.75 |
| BM610 | Doxorubicin | 34530036 | 1.22 | 25.26 |
| BM619 | Doxorubicin | 35522038 | 1.25 | 27.16 |
| BM625 | Doxorubicin | 26786452 | 0.95 | 20.93 |
| BM642 | Doxorubicin | 33142760 | 1.17 | 27.21 |
| BM647 | Doxorubicin | 33353950 | 1.18 | 28.11 |
| BM780 | Doxorubicin | 35662482 | 1.26 | 33.22 |
| BM782 | Doxorubicin | 26308094 | 1.05 | 18.06 |
| BM783 | Doxorubicin | 25240376 | 0.89 | 18.10 |
| BM786  BM838  BM844  BM943D  BM948D  BM1110  BM1111  BM1112  BM1113  BM1114  BM1116  BM1117  BM1119  BM1120  BM1121  BM1124  BM1125  BM1127  BM1129  BM1131  BM1134  BM1135  BM1136  BM1138  BM1142  BM1144  BM1145  BM1146  BM1148  BM1151  BM1154  BM1158  BM1159  BM1163  BM1166  BM237  BM263 | Doxorubicin  Zeocin  Zeocin  Doxorubicin  Doxorubicin  Zeocin  Zeocin  Zeocin  Zeocin  Zeocin  Zeocin  Zeocin  Zeocin  Zeocin  Zeocin  Doxorubicin  Doxorubicin  Doxorubicin  Doxorubicin  Doxorubicin  Doxorubicin  Doxorubicin  Doxorubicin  Doxorubicin  Doxorubicin  Doxorubicin  Doxorubicin  Doxorubicin  Doxorubicin  Doxorubicin  Doxorubicin  Doxorubicin  Doxorubicin  Doxorubicin  Doxorubicin  siTRF2  siTRF2 | 30489986  27510048  30325596  54366156  32018490  30051444  30014604  28178560  34012124  36534610  32320894  31739020  36243174  38293116  33815996  37451096  42981410  39770984  33179922  38399842  41210288  32227630  32310856  32501264  36006328  26853166  40775274  33298608  33398030  38233098  44860686  42379786  38406132  36588000  42523198  32759168  36337696 | 1.07  0.97  1.06  1.92  1.13  1.05  1.05  0.98  1.19  1.28  1.13  1.11  1.27  1.34  1.18  1.32  1.52  1.40  1.17  1.36  1.45  1.14  1.14  1.15  1.17  0.95  1.78  1.45  1.46  1.67  1.96  1.85  1.36  1.60  1.86  1.16  1.28 | 22.80  21.30  19.14  44.04  26.06  25.35  24.44  21.82  27.34  29.62  25.32  24.75  28.38  30.25  27.89  28.52  33.25  31.96  28.04  31.98  31.83  24.71  26.01  26.11  21.69  21.70  33.28  26.72  27.68  31.71  36.15  35.78  30.74  31.71  35.94  30.95  35.30 |
