# Supplementary material for: A cell-based model system links chromothripsis with hyperploidy
Source: Mol Syst Biol. 2015 Sep 28;11(9):828. doi: 10.15252/msb.20156505 (PMC4592670; doi:10.15252/msb.20156505)
Supplement: Supplementary file 5 [file msb0011-0828-sd5.docx]

### Table EV3

Ploidy and CT inference in the SHH-MB samples used in this study.

| Sample ID | Ploidy | *TP53* mutation | CT |
| --- | --- | --- | --- |
| ICGC_MB1 | Diploid | No | No |
| ICGC_MB3 | Diploid | No | No |
| ICGC_MB12 | Diploid | No | No |
| ICGC_MB21 | Diploid | No | No |
| ICGC_MB23 | Diploid | Yes(germline) | No |
| ICGC_MB28 | Diploid | No | No |
| ICGC_MB35 | Diploid | No | No |
| ICGC_MB37 | Diploid | No | No |
| ICGC_MB56 | Diploid | No | No |
| ICGC_MB59 | Diploid | No | No |
| ICGC_MB60 | Diploid | No | No |
| ICGC_MB61 | Diploid | No | No |
| ICGC_MB66 | Diploid | No | No |
| ICGC_MB67 | Diploid | No | No |
| ICGC_MB68 | Diploid | No | No |
| ICGC_MB69 | Diploid | No | No |
| ICGC_MB74 | Diploid | No | No |
| ICGC_MB75 | Diploid | No | No |
| ICGC_MB78 | Diploid | No | No |
| ICGC_MB79 | Diploid | No | No |
| ICGC_MB81 | Diploid | No | No |
| ICGC_MB82 | Diploid | No | No |
| ICGC_MB88 | Diploid | No | No |
| ICGC_MB102 | Diploid | No | No |
| ICGC_MB125 | Diploid | No | No |
| ICGC_MB132 | Diploid | No | No |
| ICGC_MB137 | Diploid | Yes(germline) | Yes |
| ICGC_MB143 | Diploid | No | No |
| ICGC_MB155 | Diploid | No | No |
| ICGC_MB171 | Diploid | No | No |
| ICGC_MB178 | Diploid | No | No |
| ICGC_MB181 | Diploid | No | No |
| ICGC_MB34 | Tetraploid | Yes(somatic) | Yes |
| ICGC_MB53 | Tetraploid | No | No |
| ICGC_MB63 | Tetraploid | No | No |
| ICGC_MB77 | Tetraploid | No | No |
| ICGC_MB101 | Tetraploid | No | No |
| ICGC_MB104 | Tetraploid | No | No |
| ICGC_MB126 | Tetraploid | No | No |
| ICGC_MB145 | Tetraploid | Yes(germline) | Yes |
| ICGC_MB243 | Tetraploid | Yes(germline) | Yes |
| LFS_MB1 | Diploid | Yes(germline) | Yes |
| LFS_MB2 | Tetraploid | Yes(germline) | Yes |
| LFS_MB4 | Tetraploid | Yes(germline) | Yes |
