# Supplementary material for: A cell-based model system links chromothripsis with hyperploidy
Source: Mol Syst Biol. 2015 Sep 28;11(9):828. doi: 10.15252/msb.20156505 (PMC4592670; doi:10.15252/msb.20156505)
Supplement: Supplementary file 6 [file msb0011-0828-sd6.zip › png plots/readme.docx]

## DataSet EV1:

a zip file containing all circos plots of the Mate-pair sequenced samples.
