# Supplementary figures and images for: A cell-based model system links chromothripsis with hyperploidy
Source: Mol Syst Biol. 2015 Sep 28;11(9):828. doi: 10.15252/msb.20156505 (PMC4592670; doi:10.15252/msb.20156505)

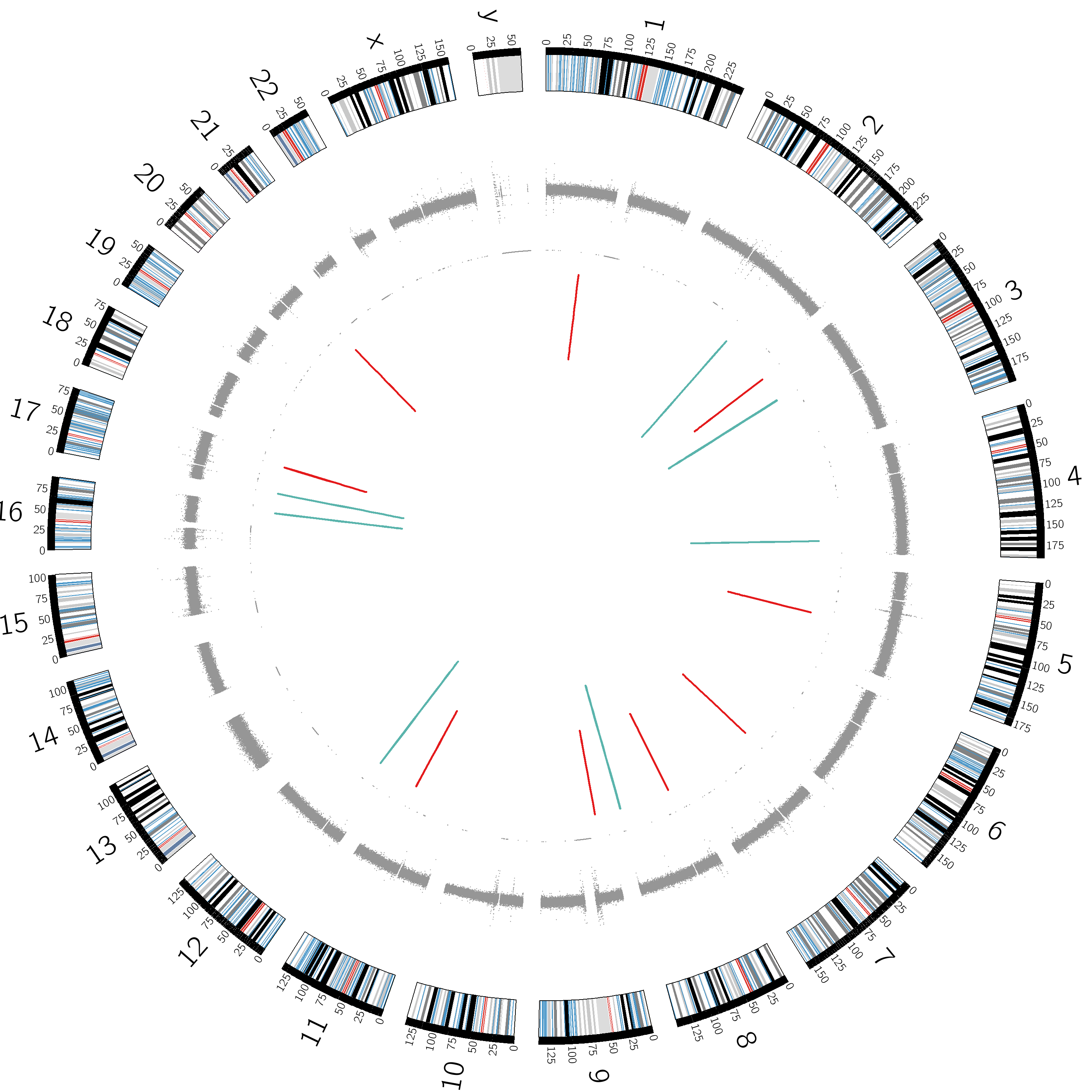

Supplement: Supplementary file 6 [file msb0011-0828-sd6.zip › png plots/BM1110.png]

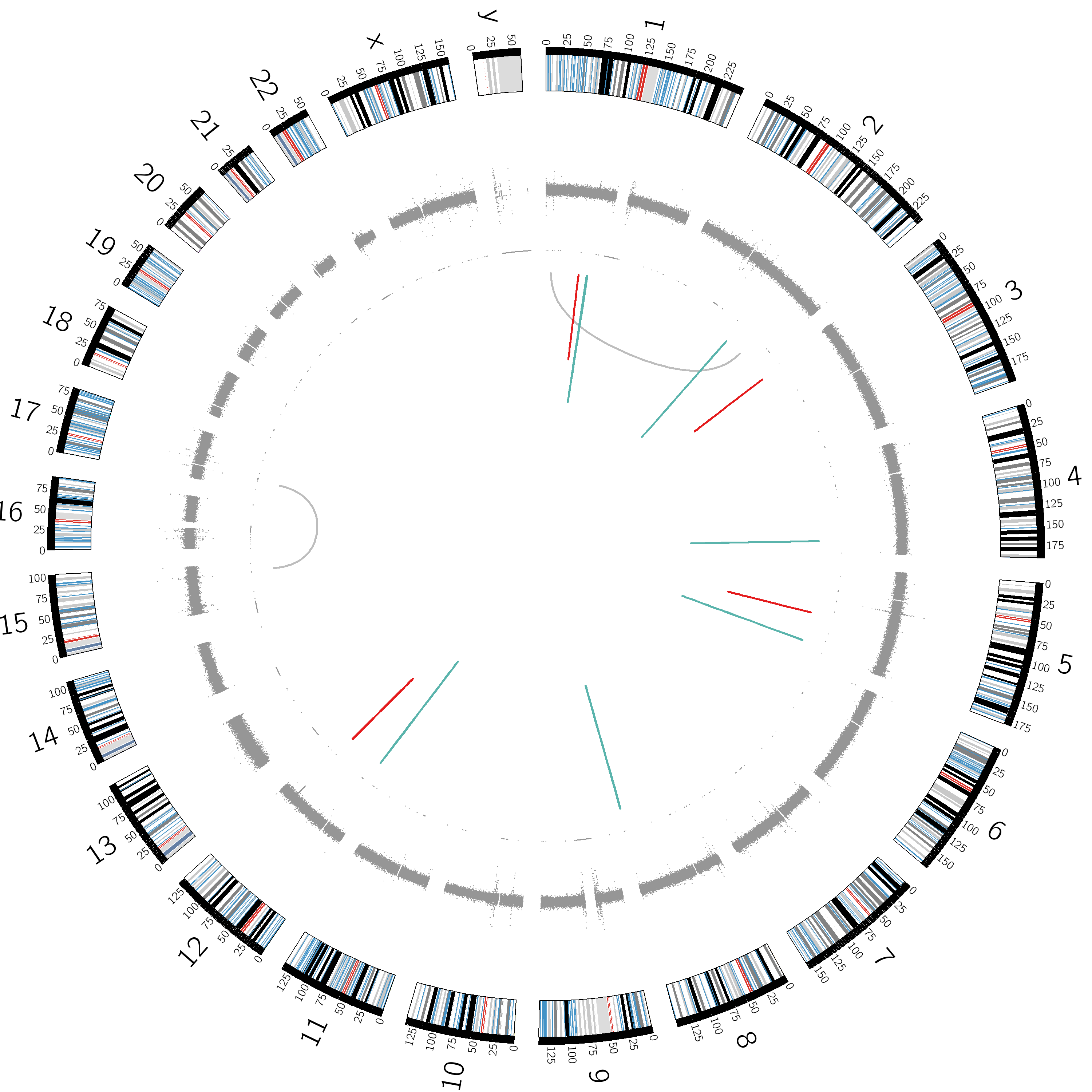

Supplement: Supplementary file 6 [file msb0011-0828-sd6.zip › png plots/BM1111.png]

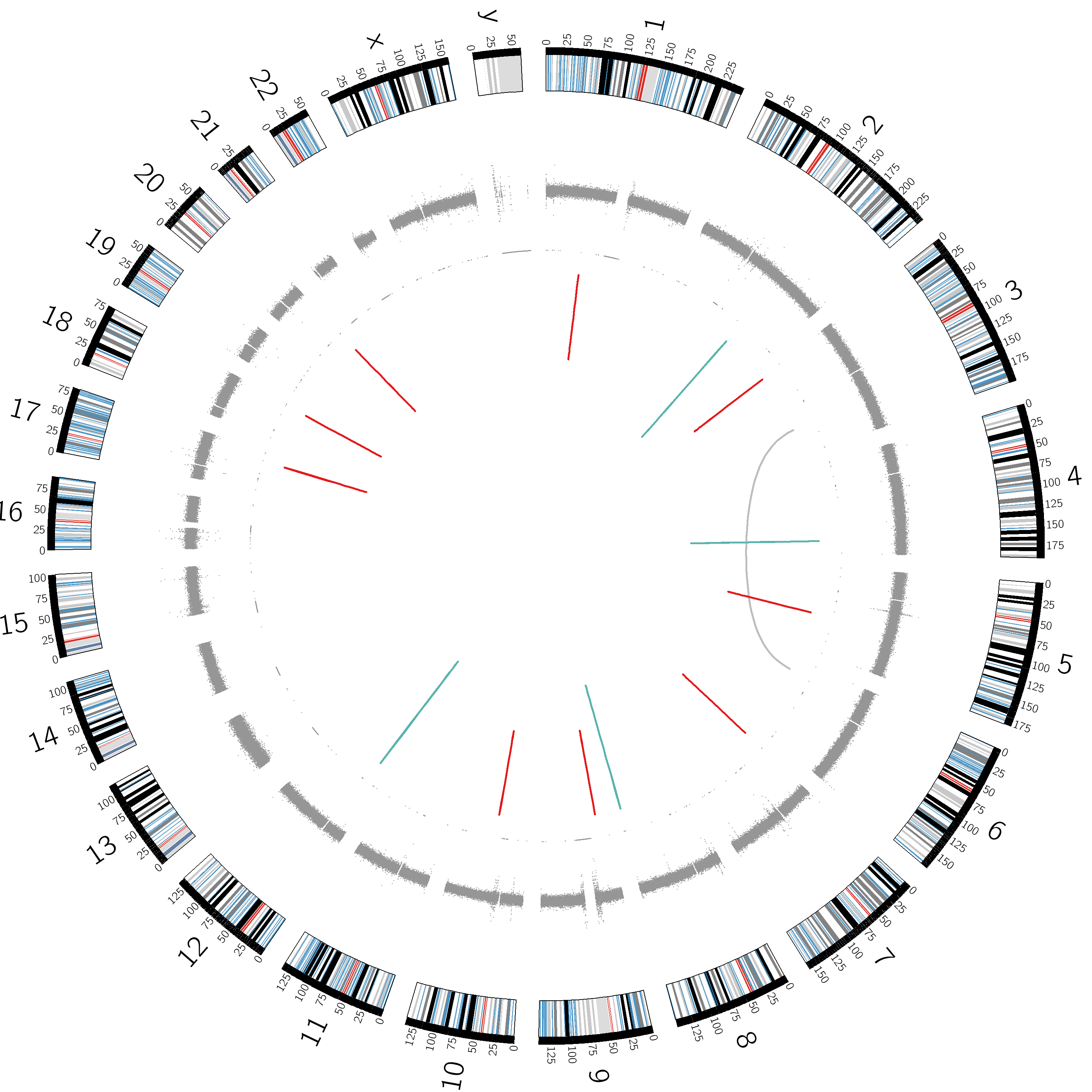

Supplement: Supplementary file 6 [file msb0011-0828-sd6.zip › png plots/BM1112.png]

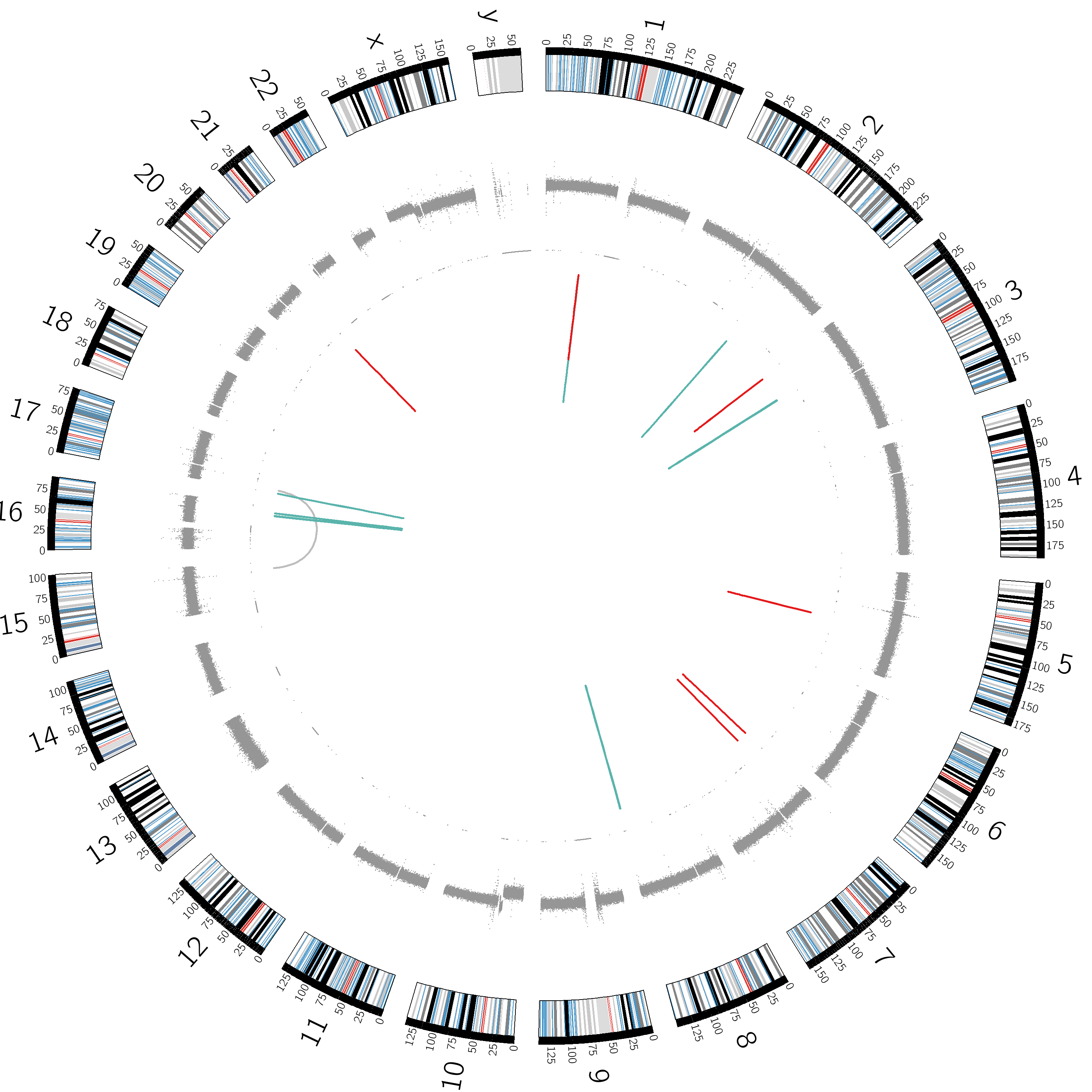

Supplement: Supplementary file 6 [file msb0011-0828-sd6.zip › png plots/BM1113.png]

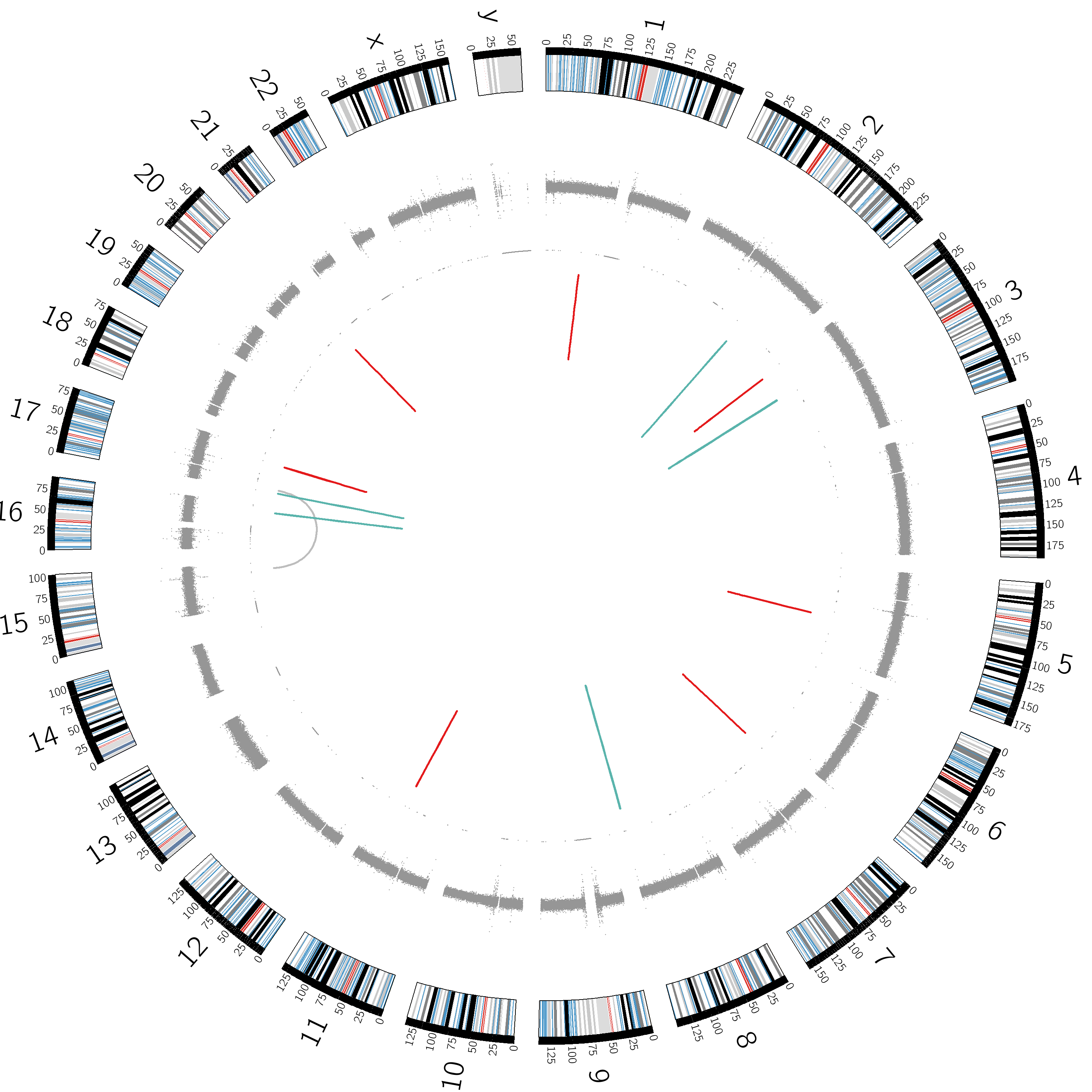

Supplement: Supplementary file 6 [file msb0011-0828-sd6.zip › png plots/BM1114.png]

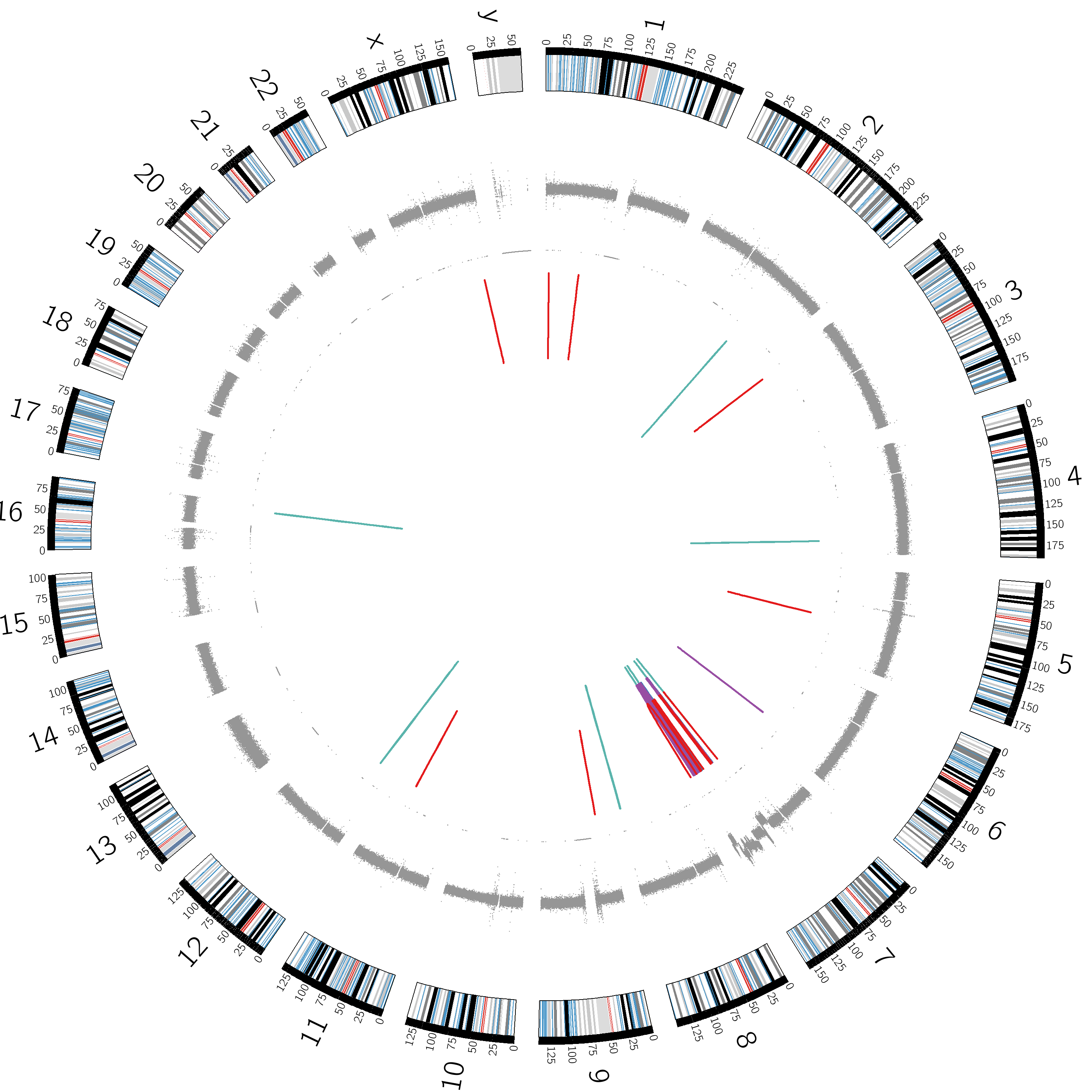

Supplement: Supplementary file 6 [file msb0011-0828-sd6.zip › png plots/BM1116.png]

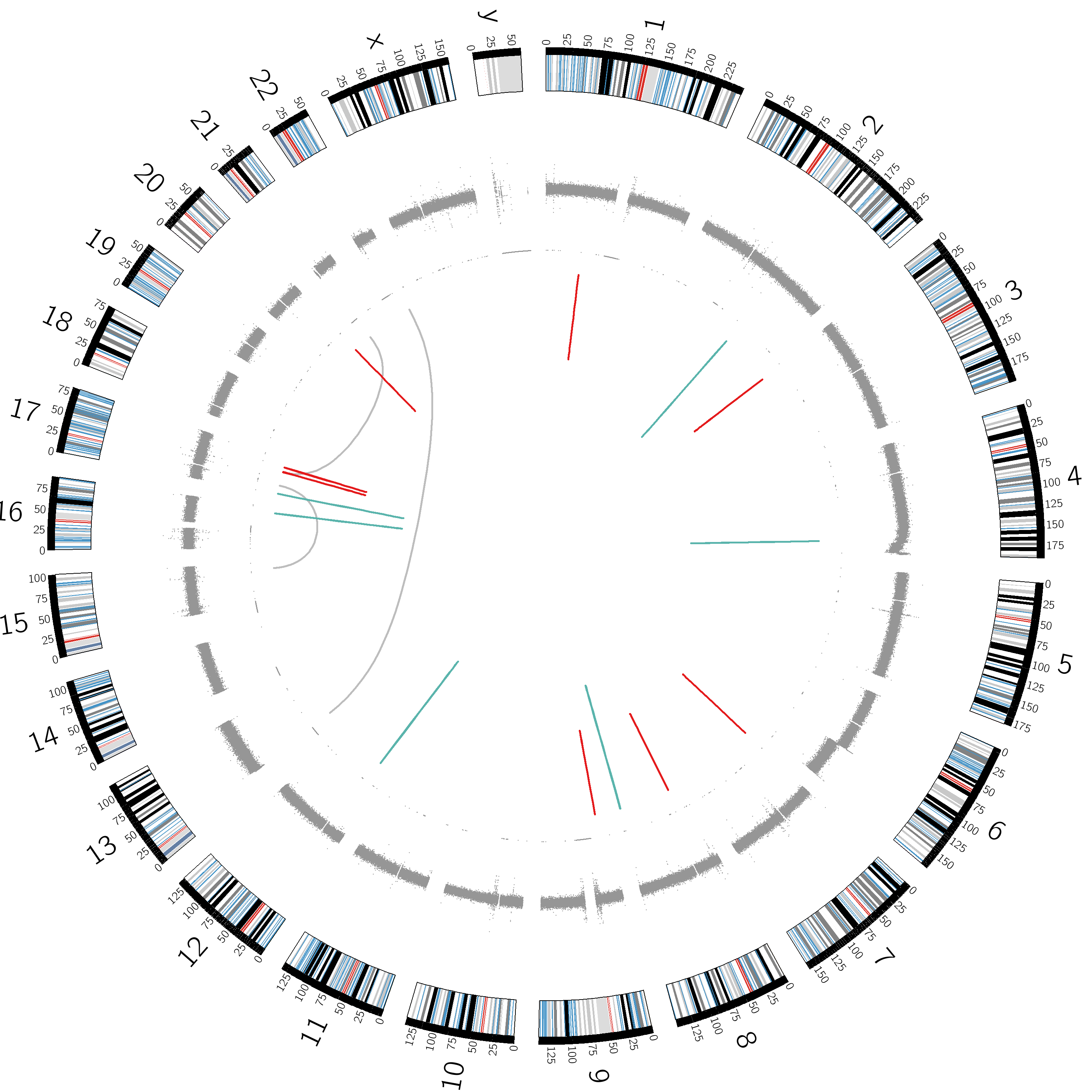

Supplement: Supplementary file 6 [file msb0011-0828-sd6.zip › png plots/BM1117.png]

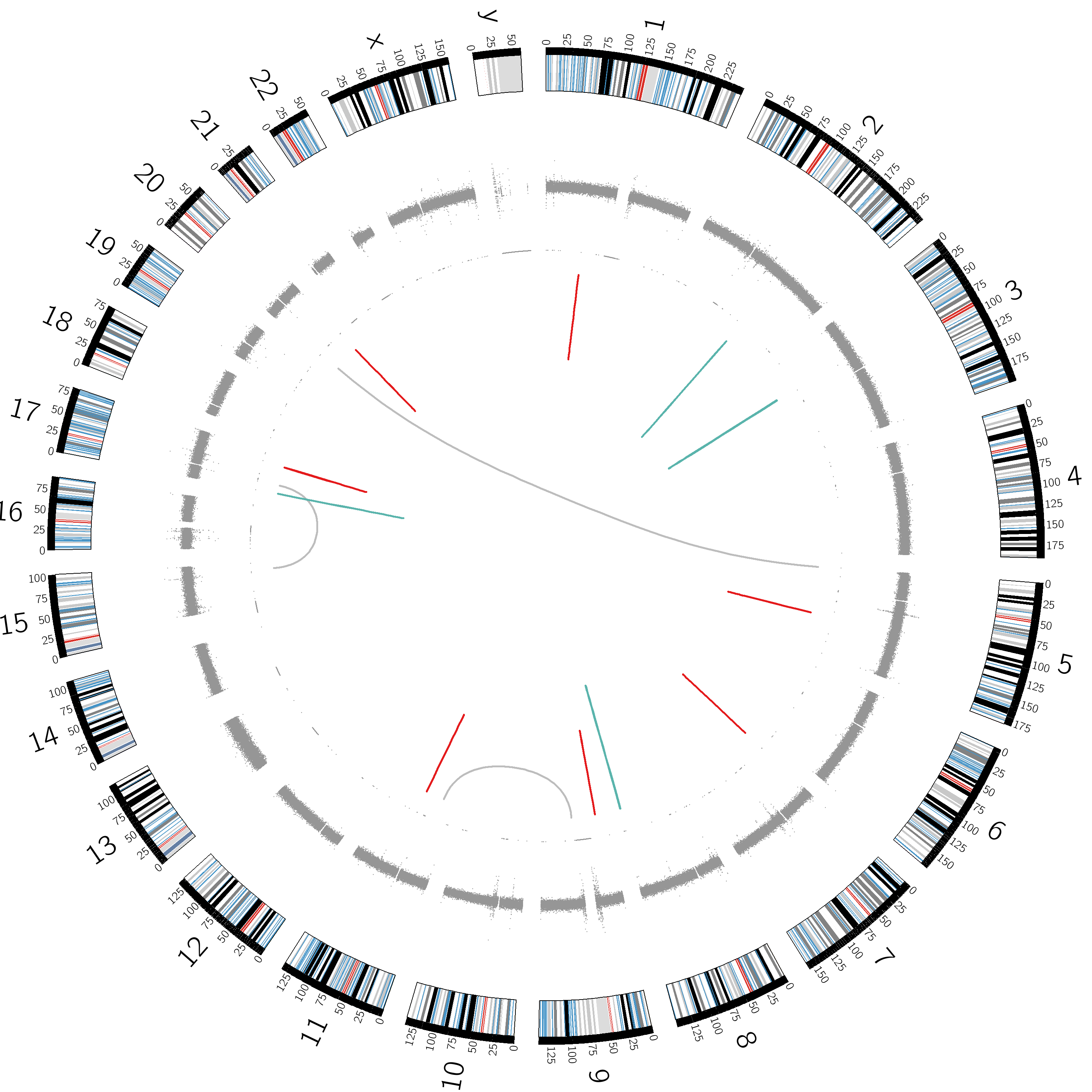

Supplement: Supplementary file 6 [file msb0011-0828-sd6.zip › png plots/BM1119.png]

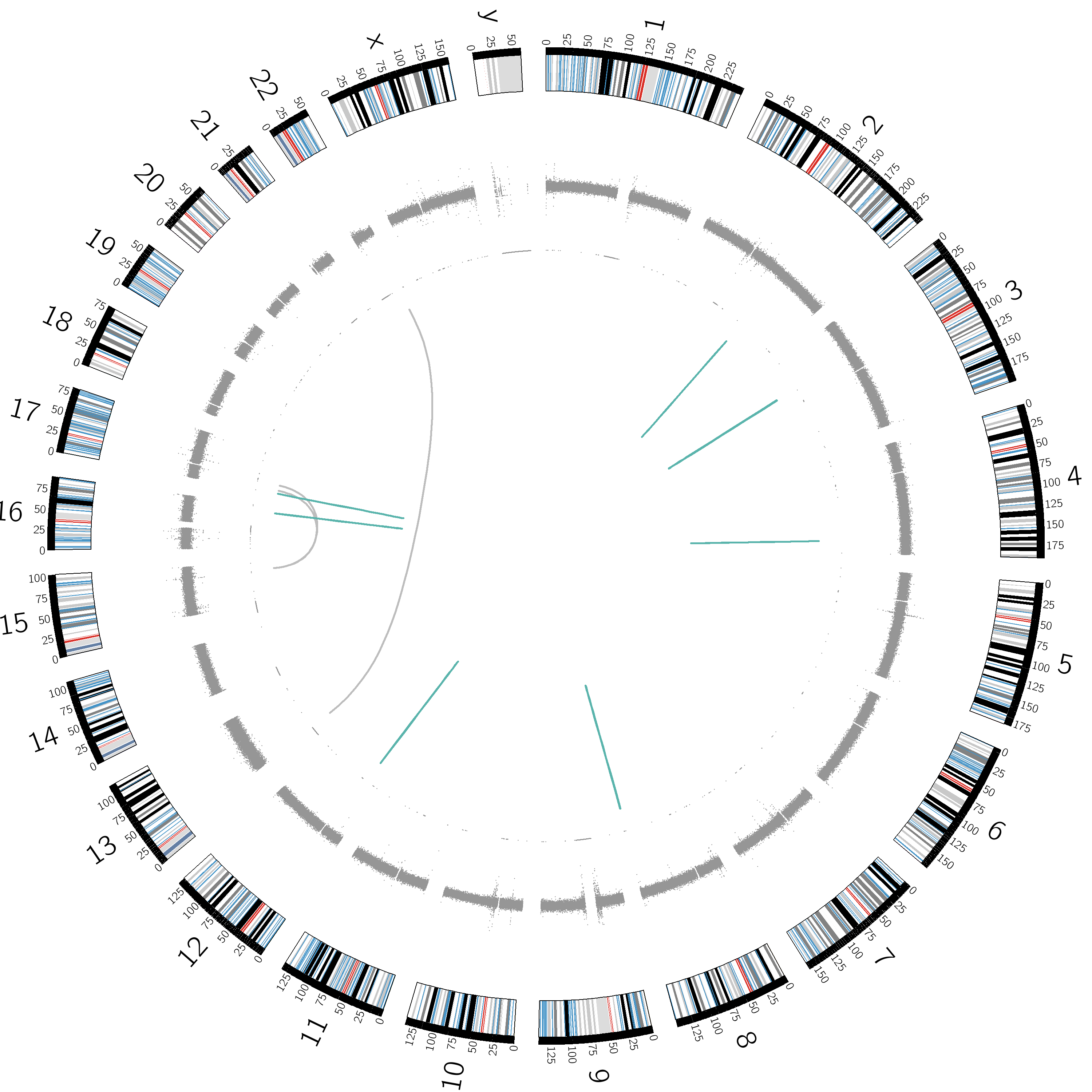

Supplement: Supplementary file 6 [file msb0011-0828-sd6.zip › png plots/BM1120.png]

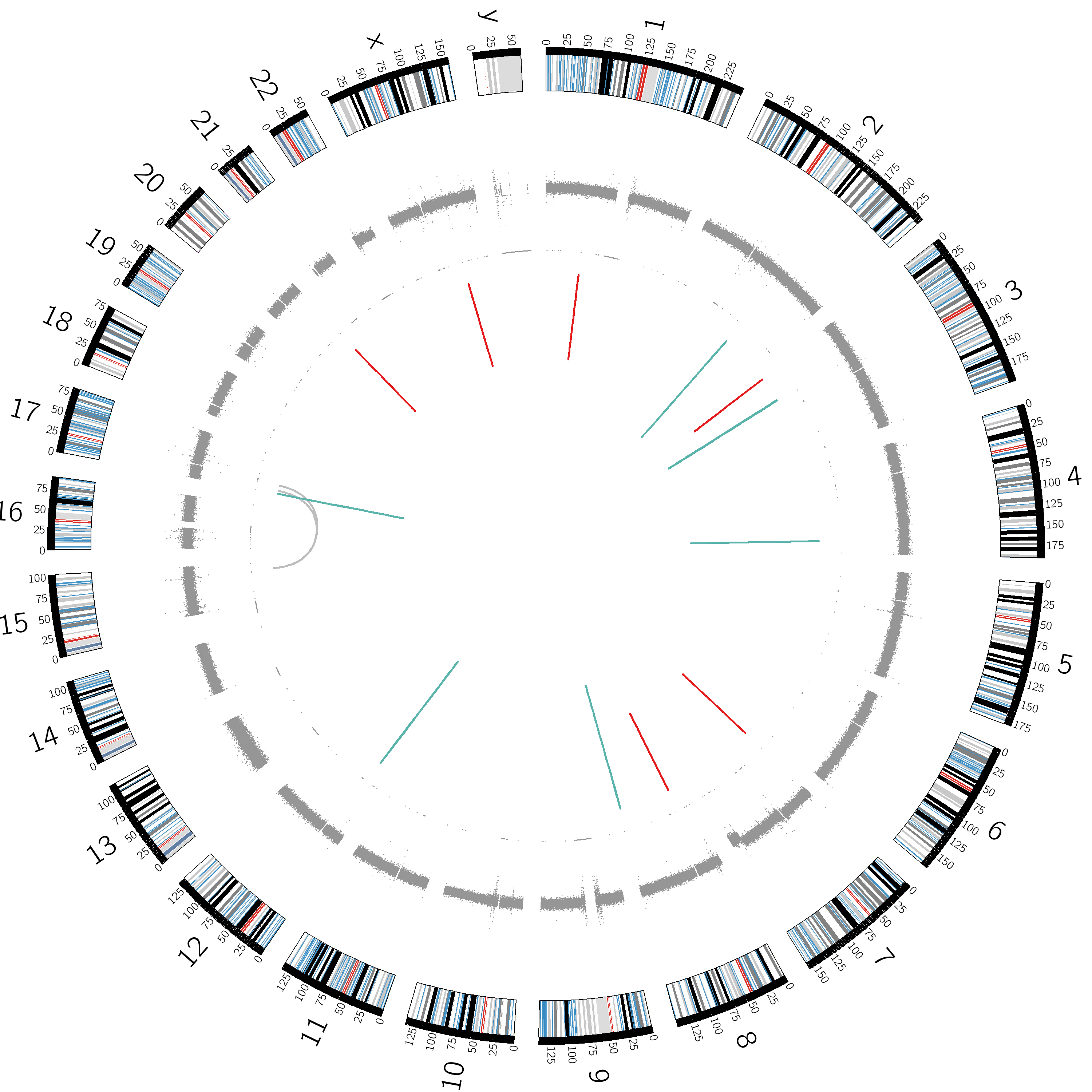

Supplement: Supplementary file 6 [file msb0011-0828-sd6.zip › png plots/BM1121.png]

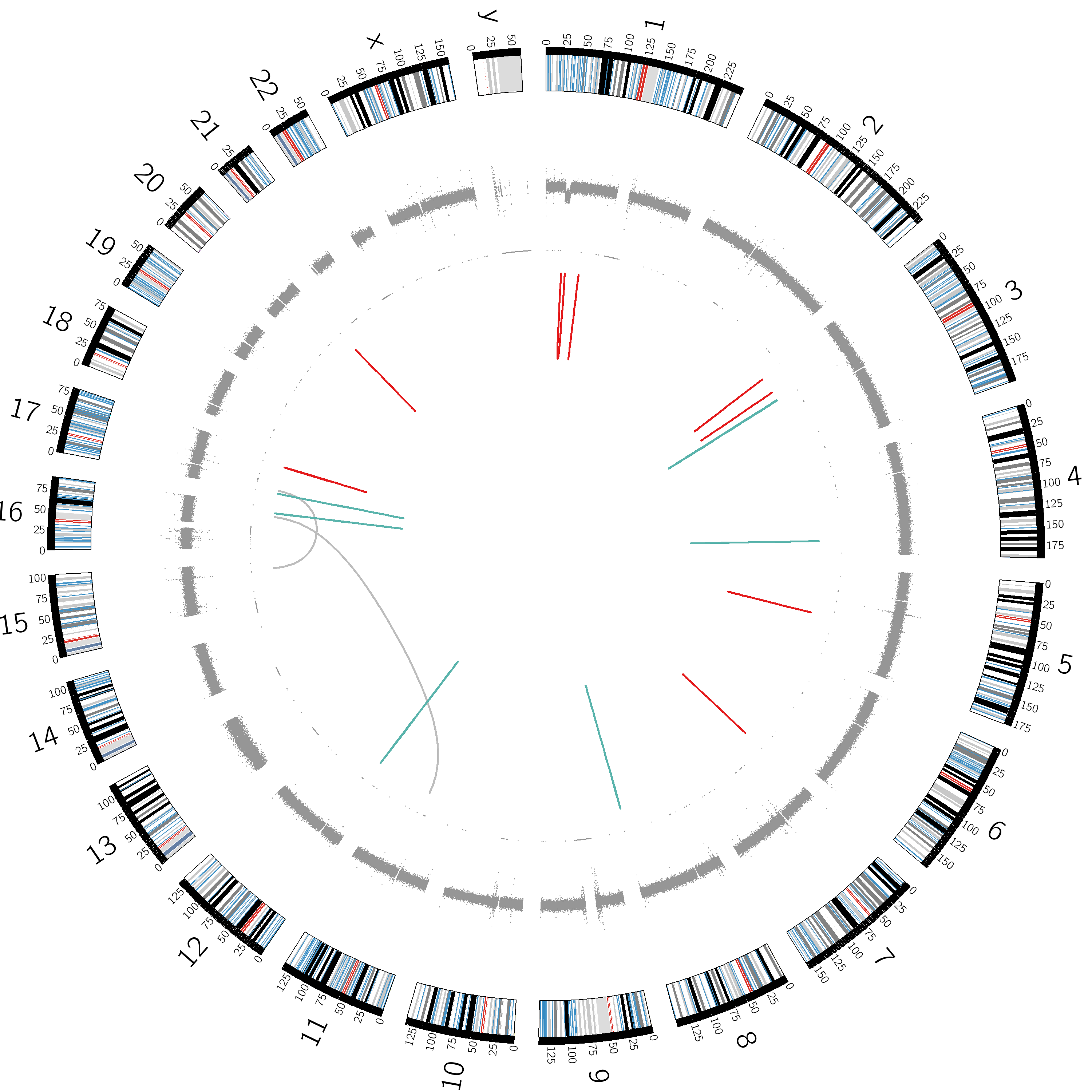

Supplement: Supplementary file 6 [file msb0011-0828-sd6.zip › png plots/BM1124.png]

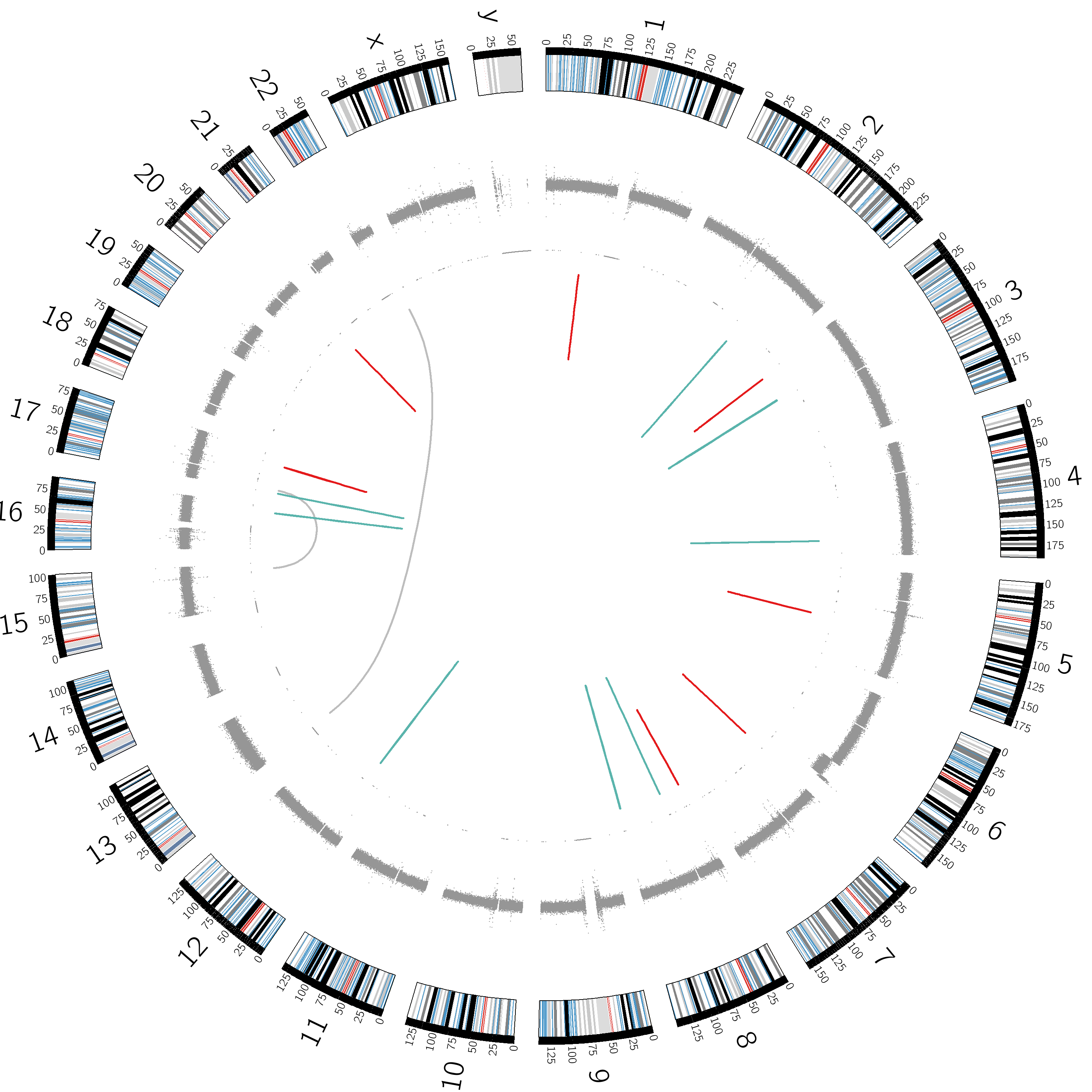

Supplement: Supplementary file 6 [file msb0011-0828-sd6.zip › png plots/BM1125.png]

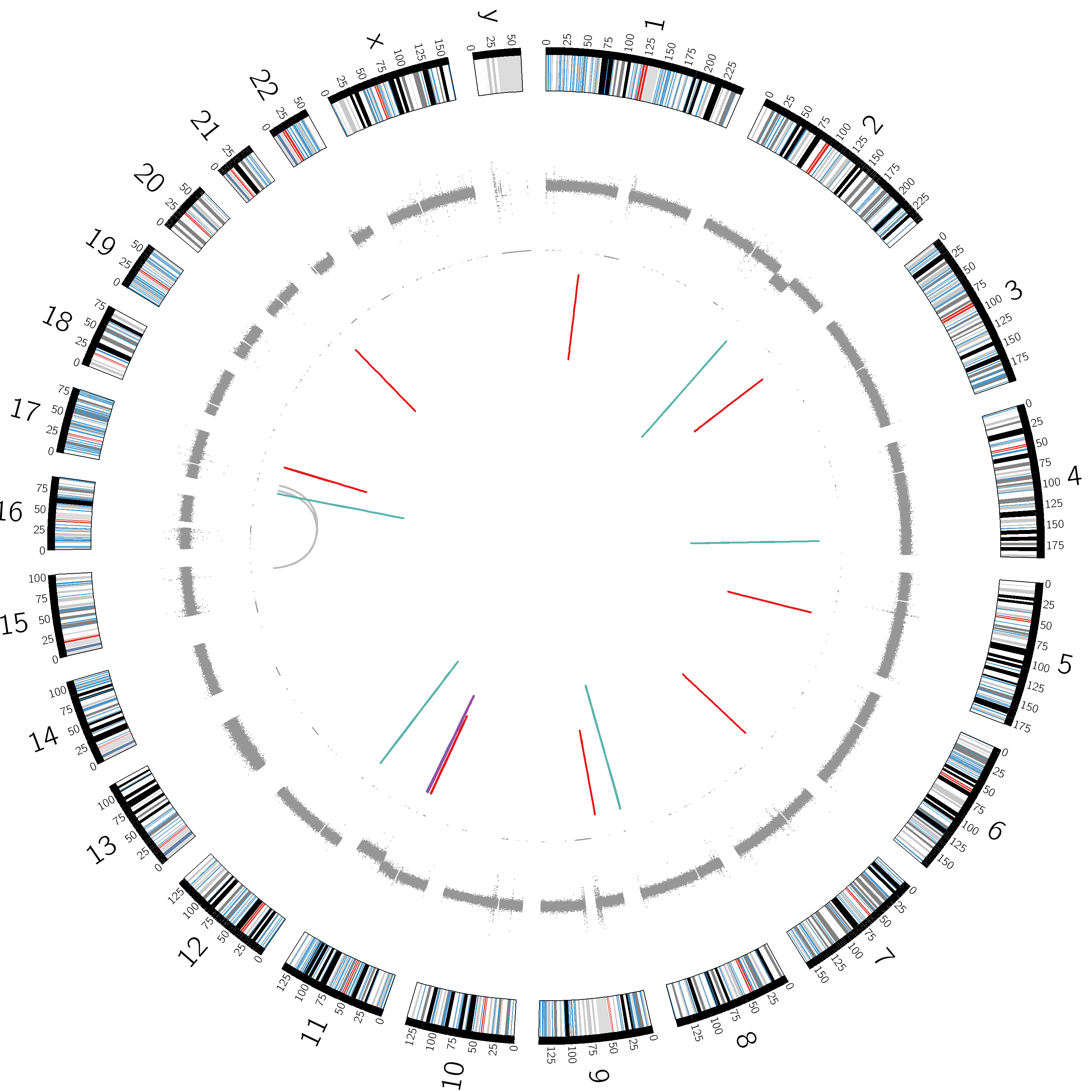

Supplement: Supplementary file 6 [file msb0011-0828-sd6.zip › png plots/BM1127.png]

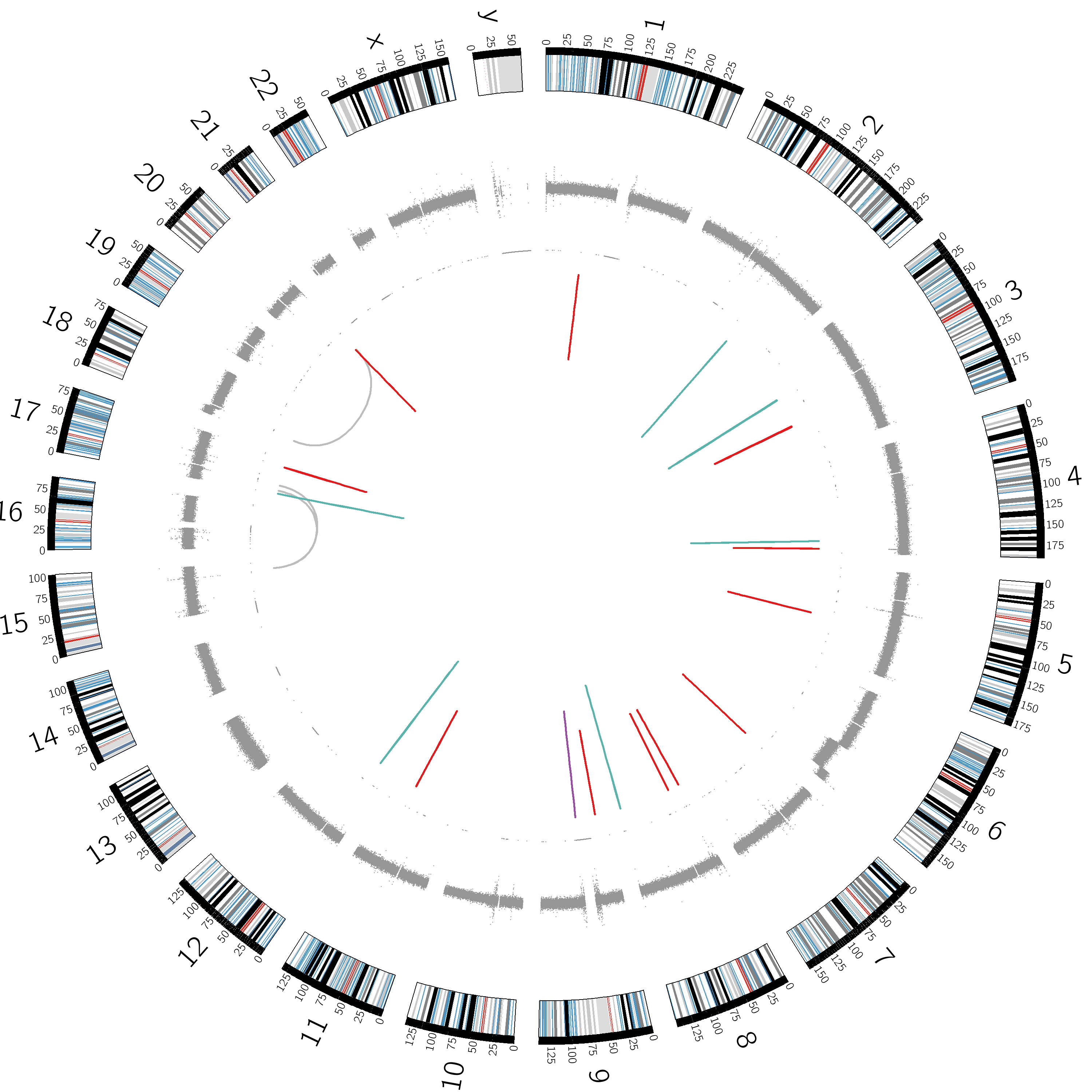

Supplement: Supplementary file 6 [file msb0011-0828-sd6.zip › png plots/BM1129.png]

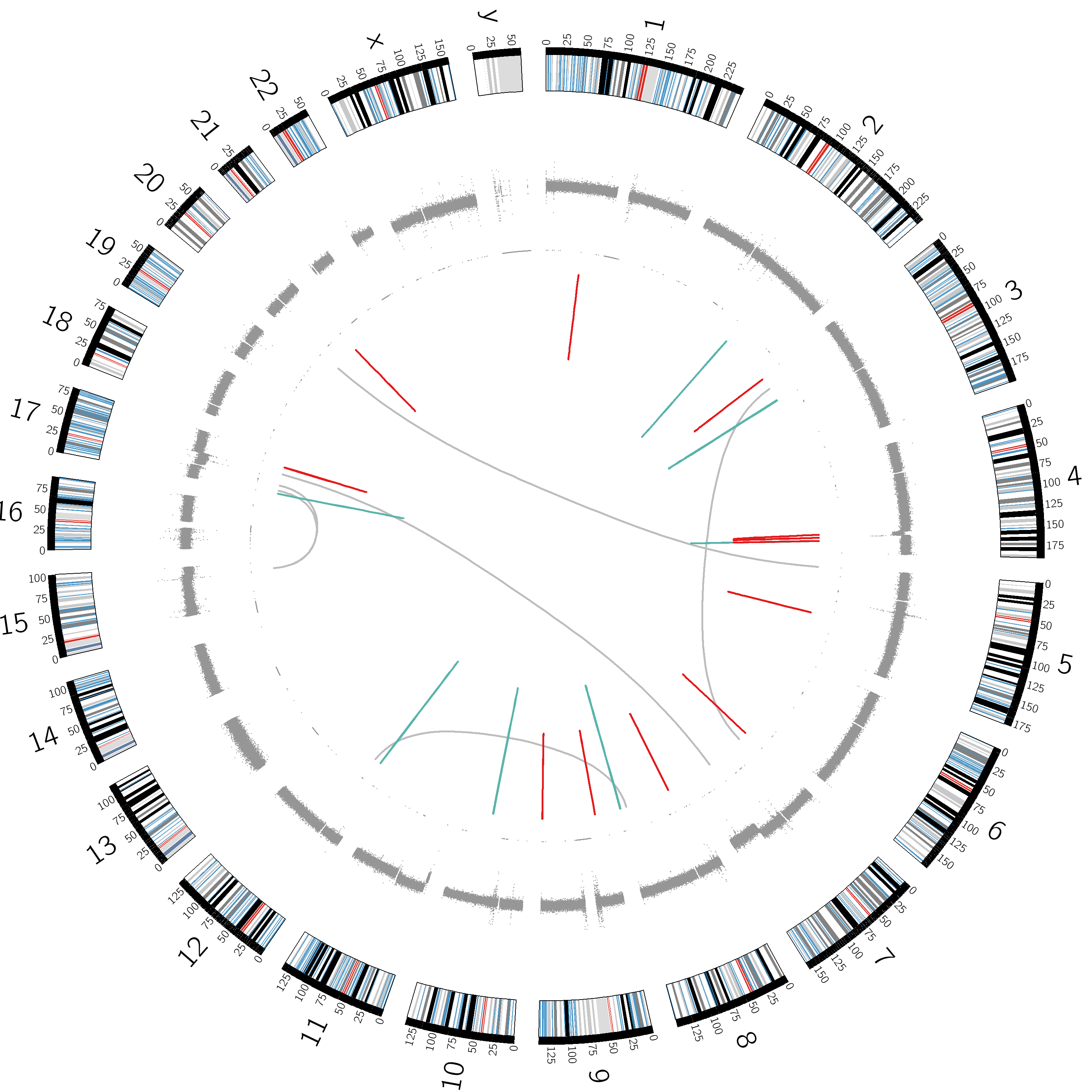

Supplement: Supplementary file 6 [file msb0011-0828-sd6.zip › png plots/BM1131.png]

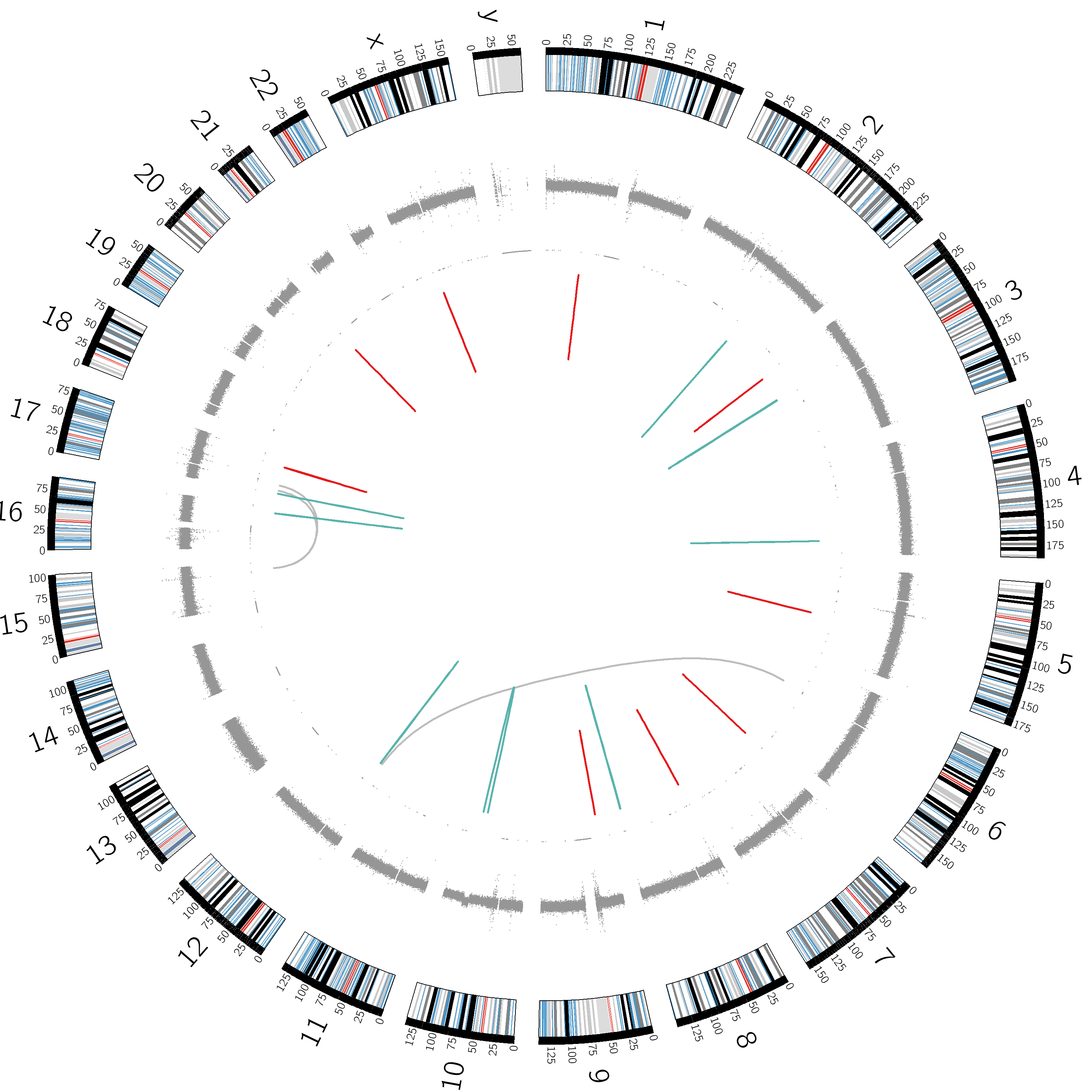

Supplement: Supplementary file 6 [file msb0011-0828-sd6.zip › png plots/BM1134.png]

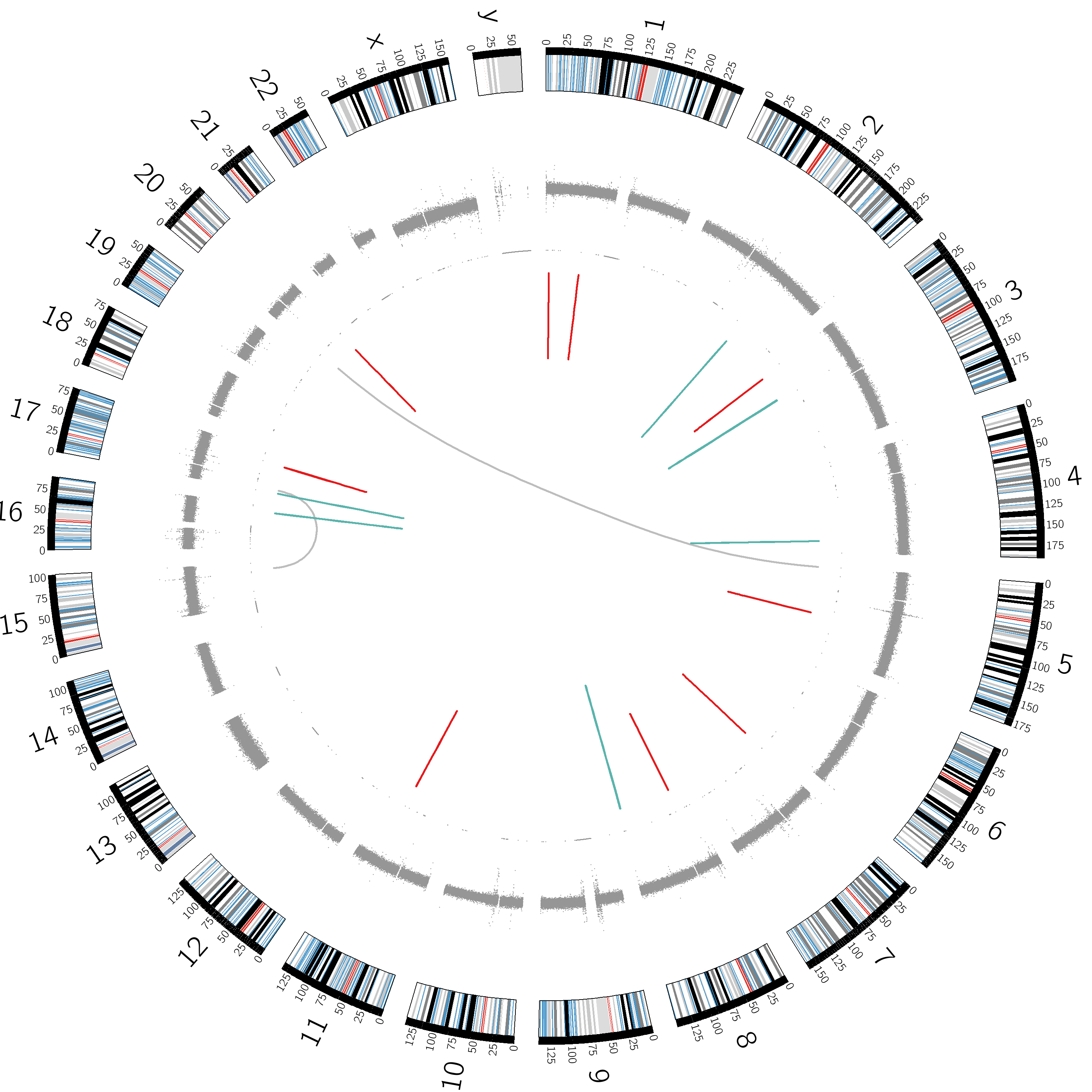

Supplement: Supplementary file 6 [file msb0011-0828-sd6.zip › png plots/BM1135.png]

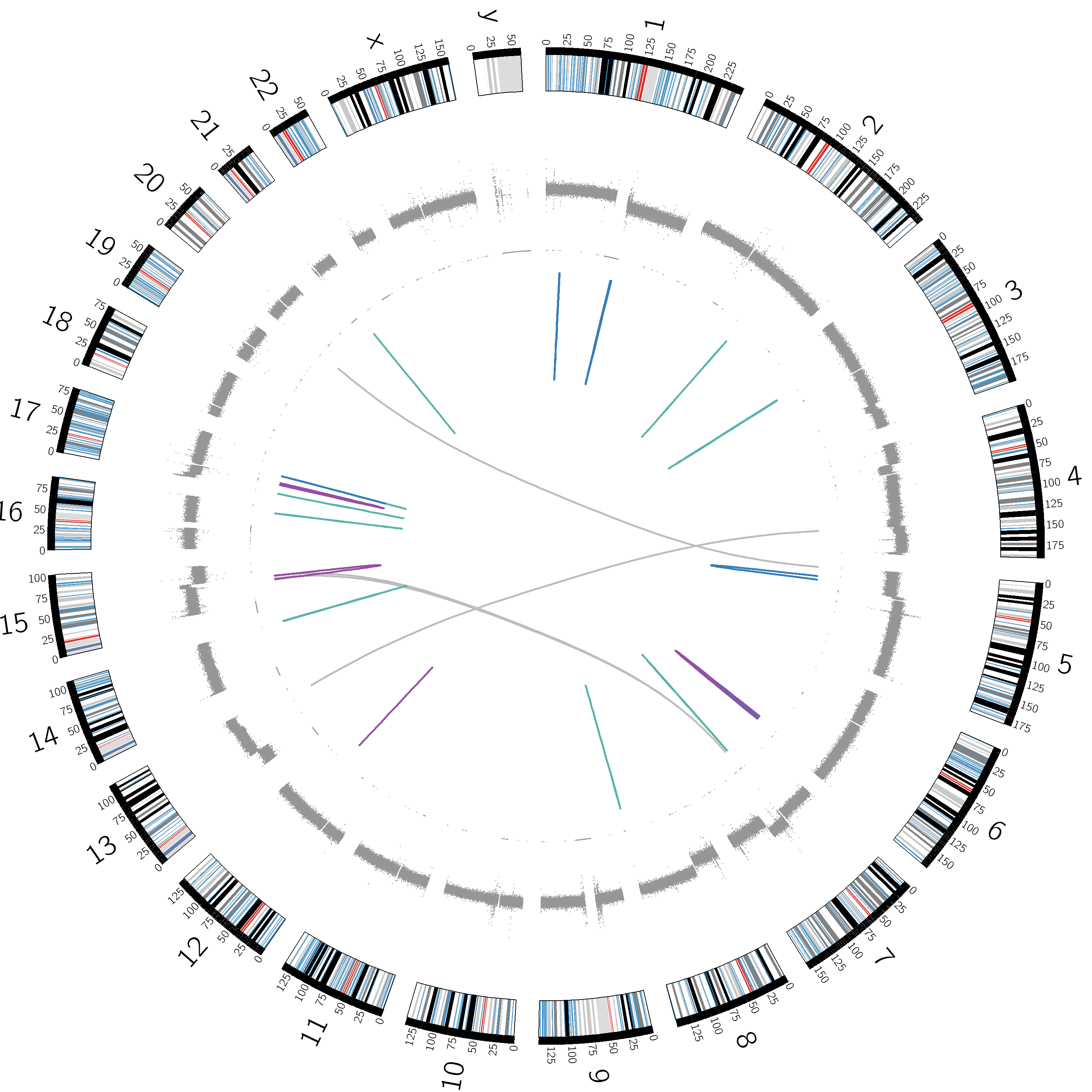

Supplement: Supplementary file 6 [file msb0011-0828-sd6.zip › png plots/BM1136.png]

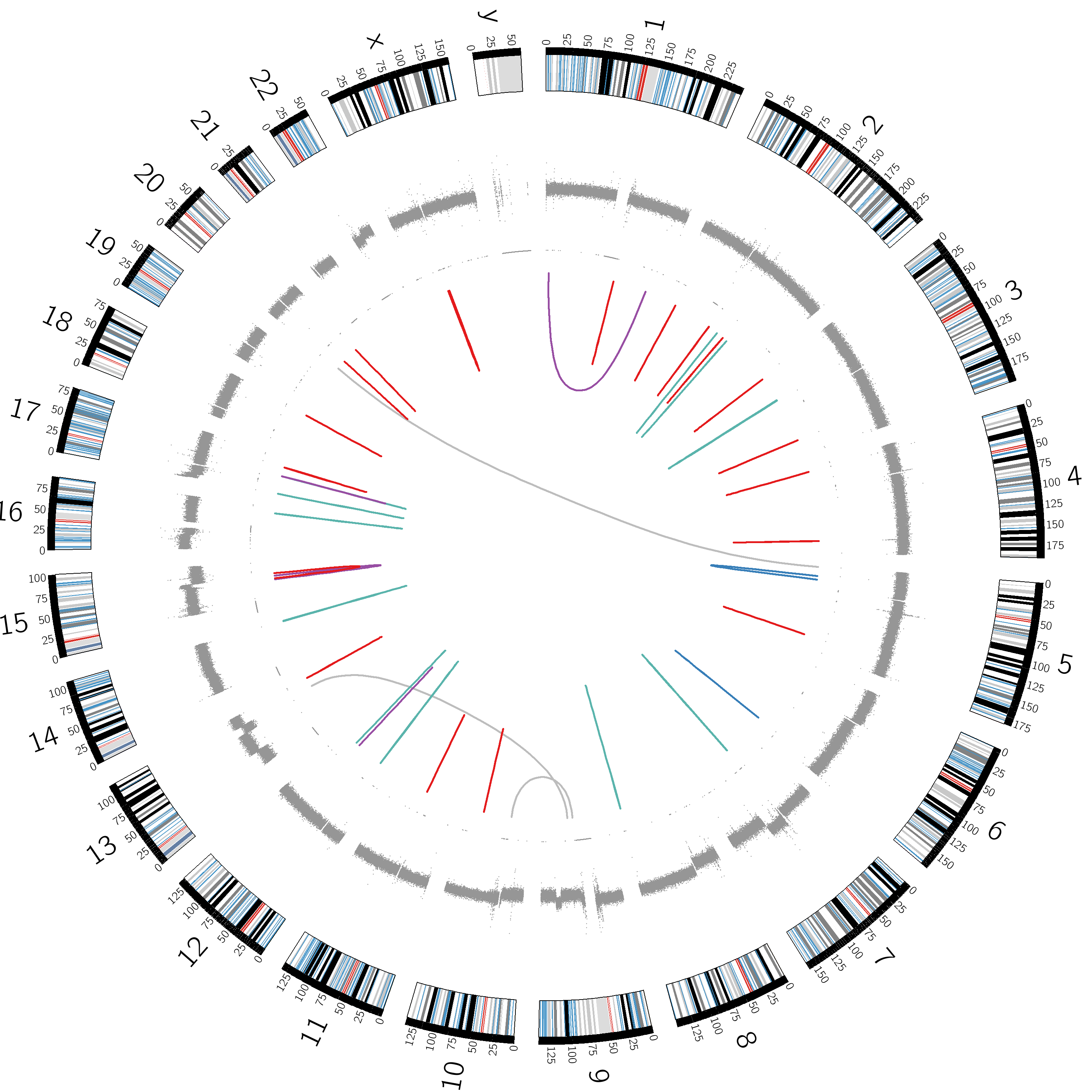

Supplement: Supplementary file 6 [file msb0011-0828-sd6.zip › png plots/BM1138.png]

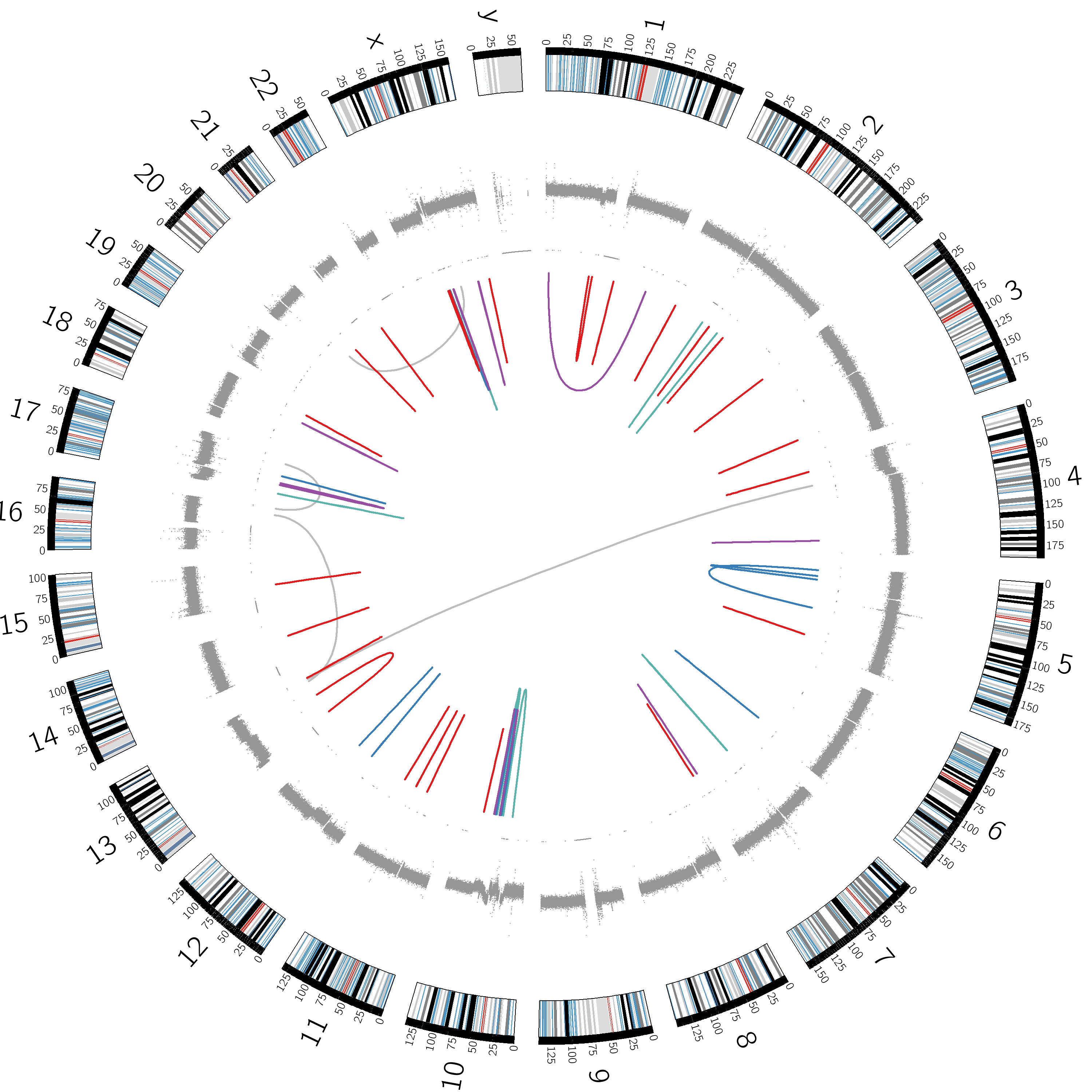

Supplement: Supplementary file 6 [file msb0011-0828-sd6.zip › png plots/BM1142.png]

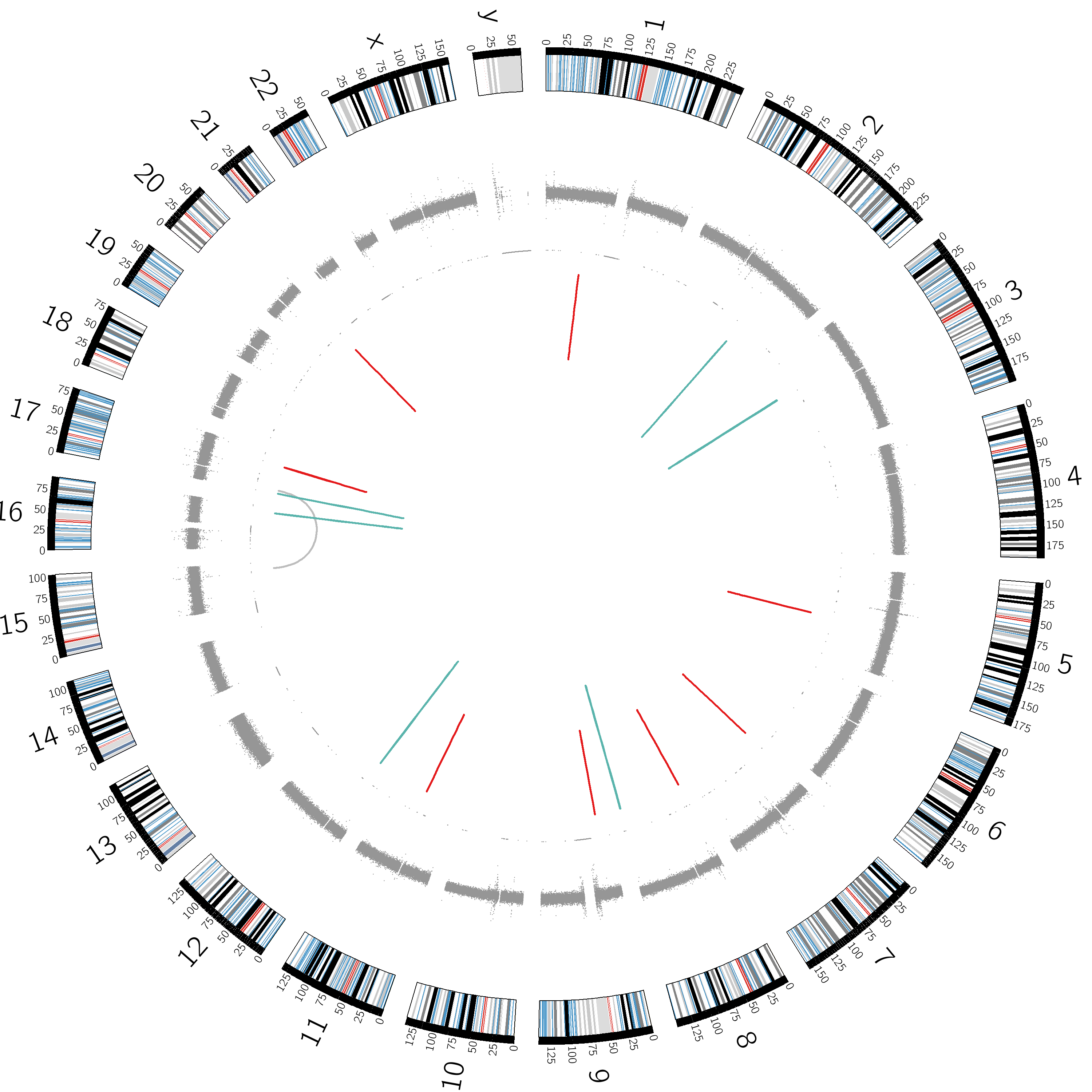

Supplement: Supplementary file 6 [file msb0011-0828-sd6.zip › png plots/BM1144.png]

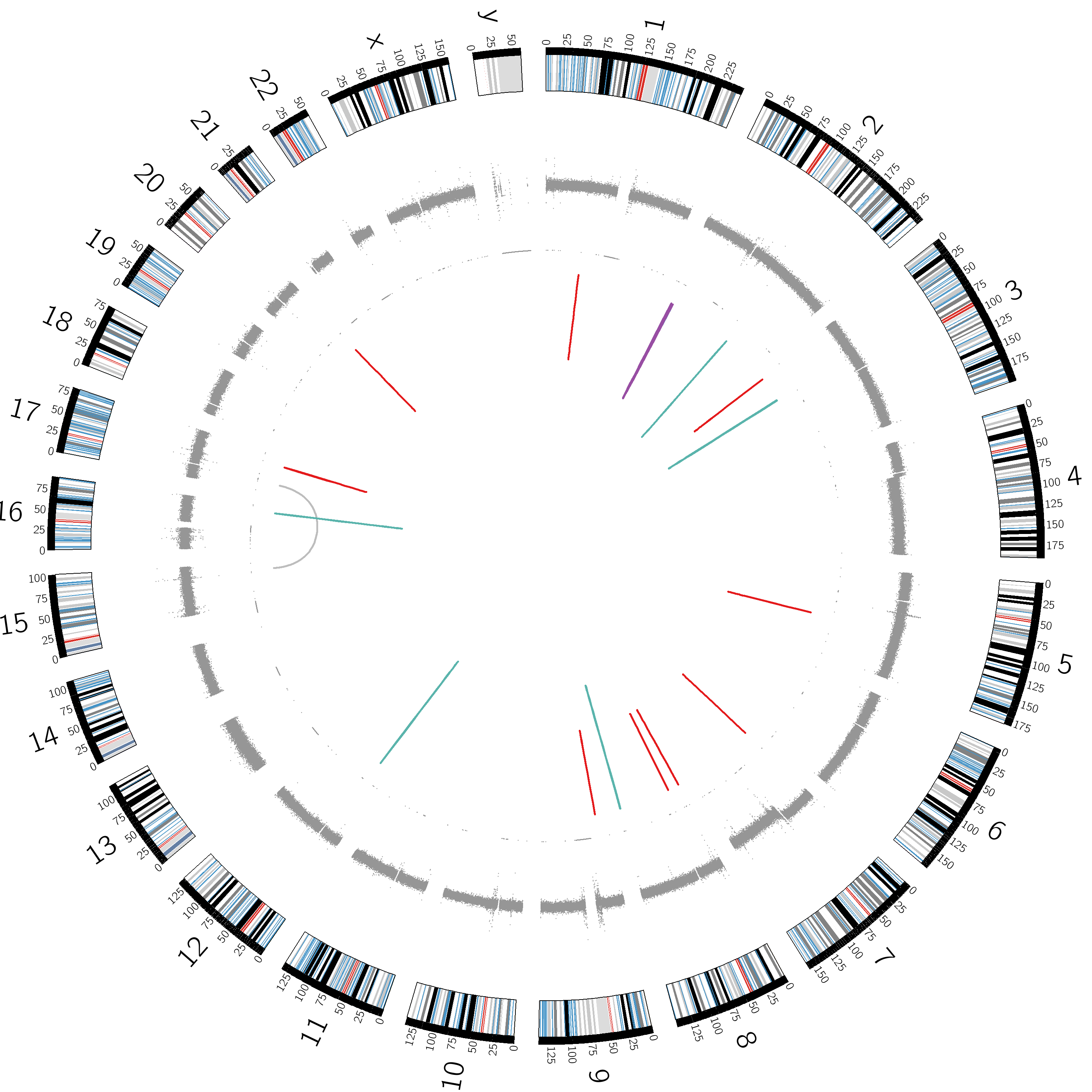

Supplement: Supplementary file 6 [file msb0011-0828-sd6.zip › png plots/BM1145.png]

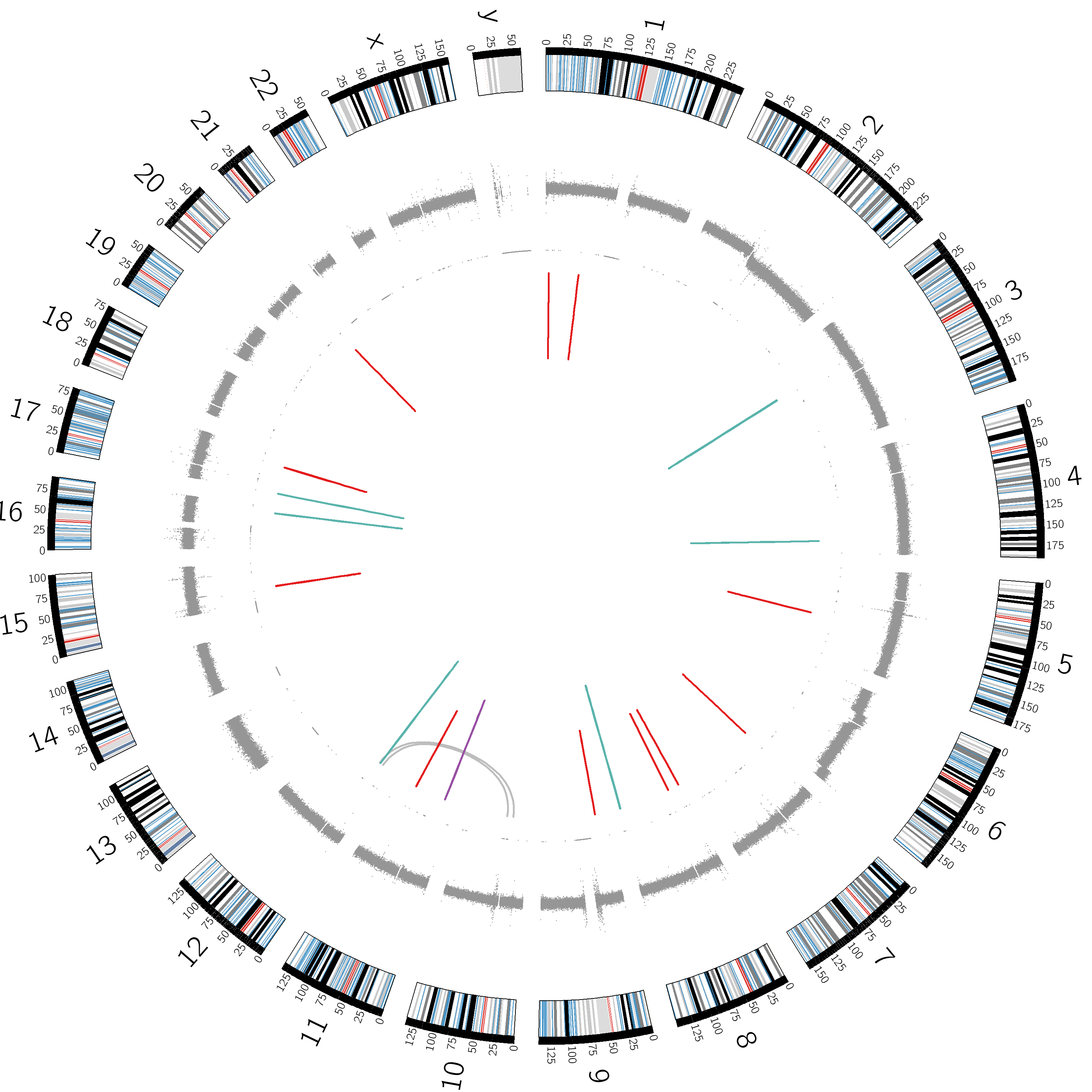

Supplement: Supplementary file 6 [file msb0011-0828-sd6.zip › png plots/BM1146.png]

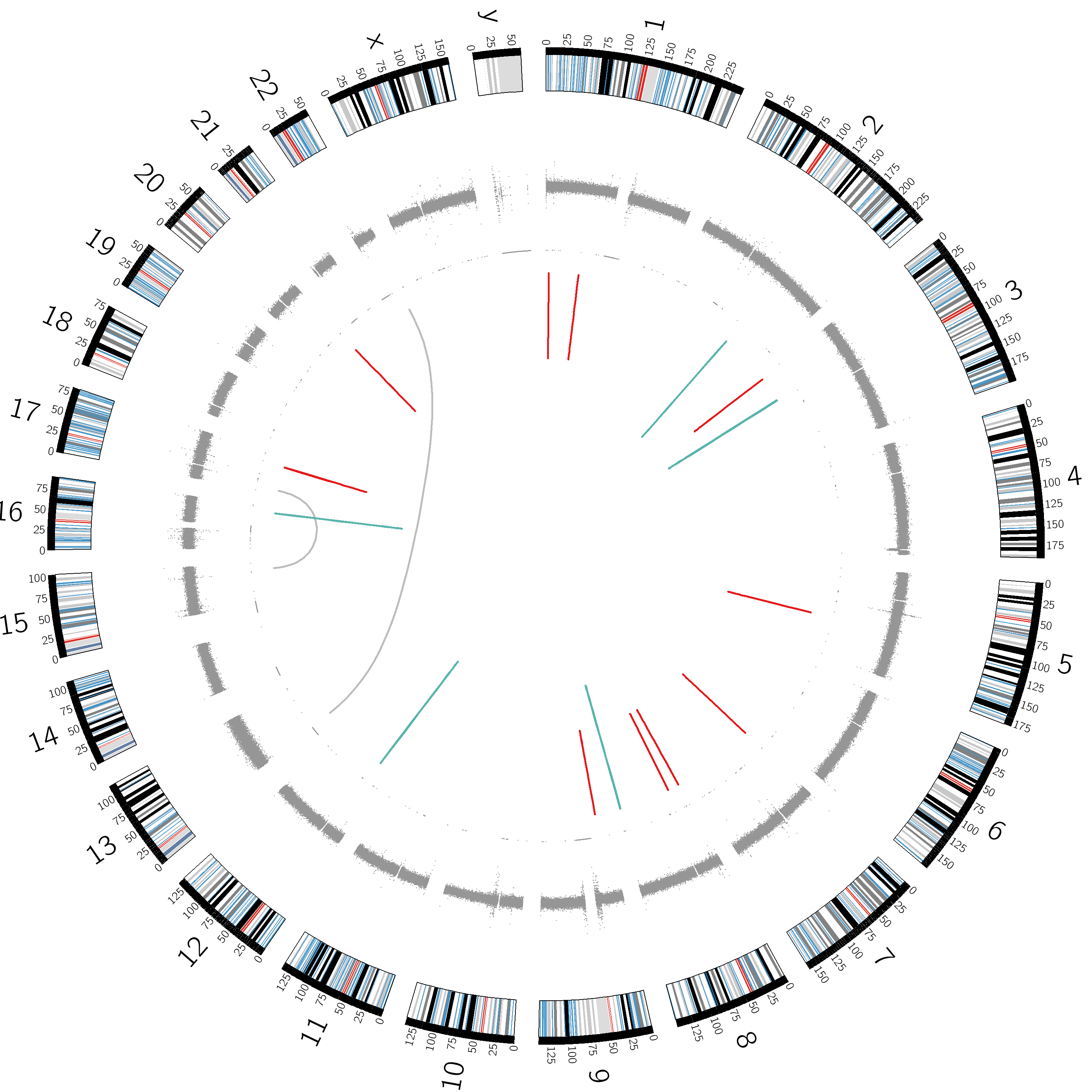

Supplement: Supplementary file 6 [file msb0011-0828-sd6.zip › png plots/BM1148.png]

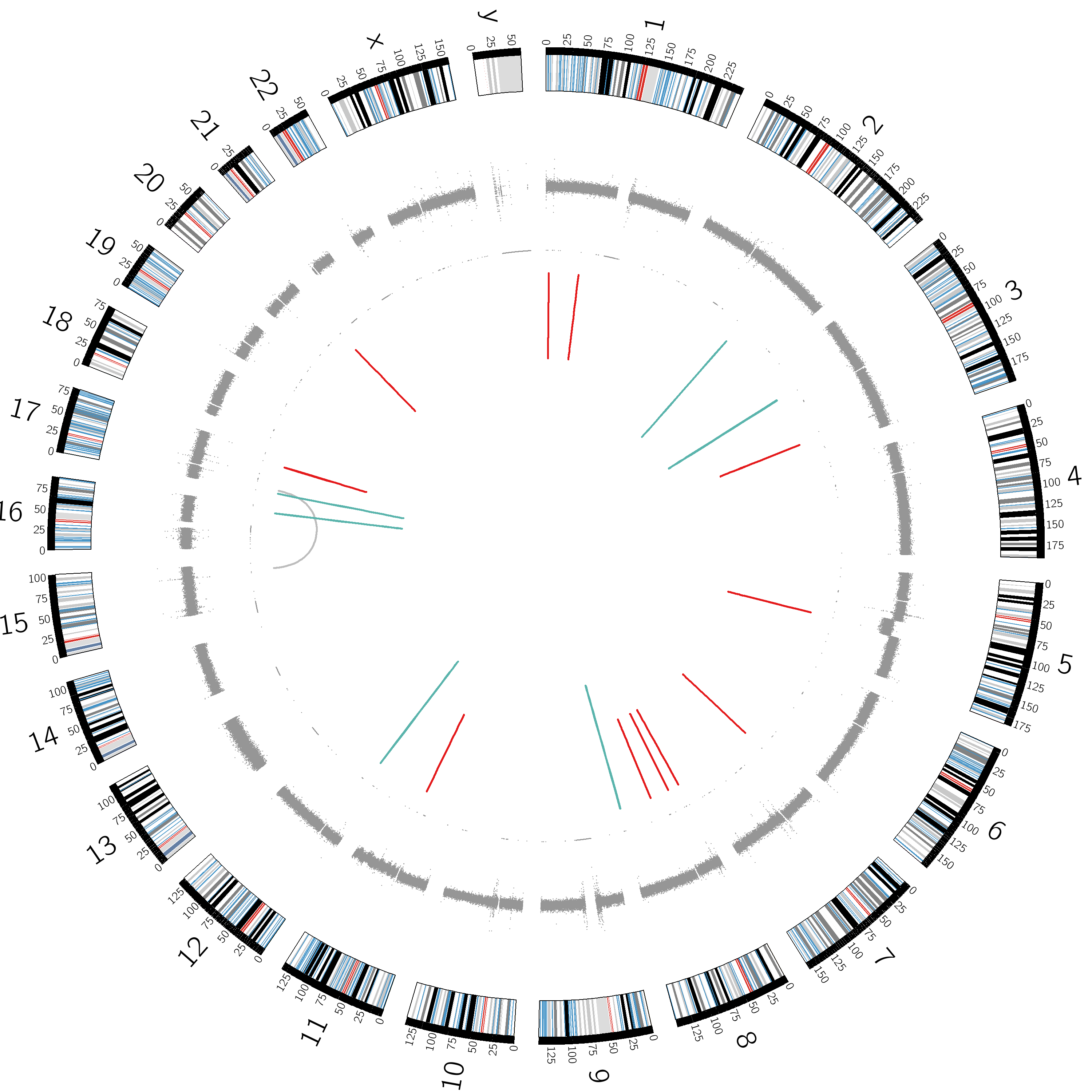

Supplement: Supplementary file 6 [file msb0011-0828-sd6.zip › png plots/BM1151.png]

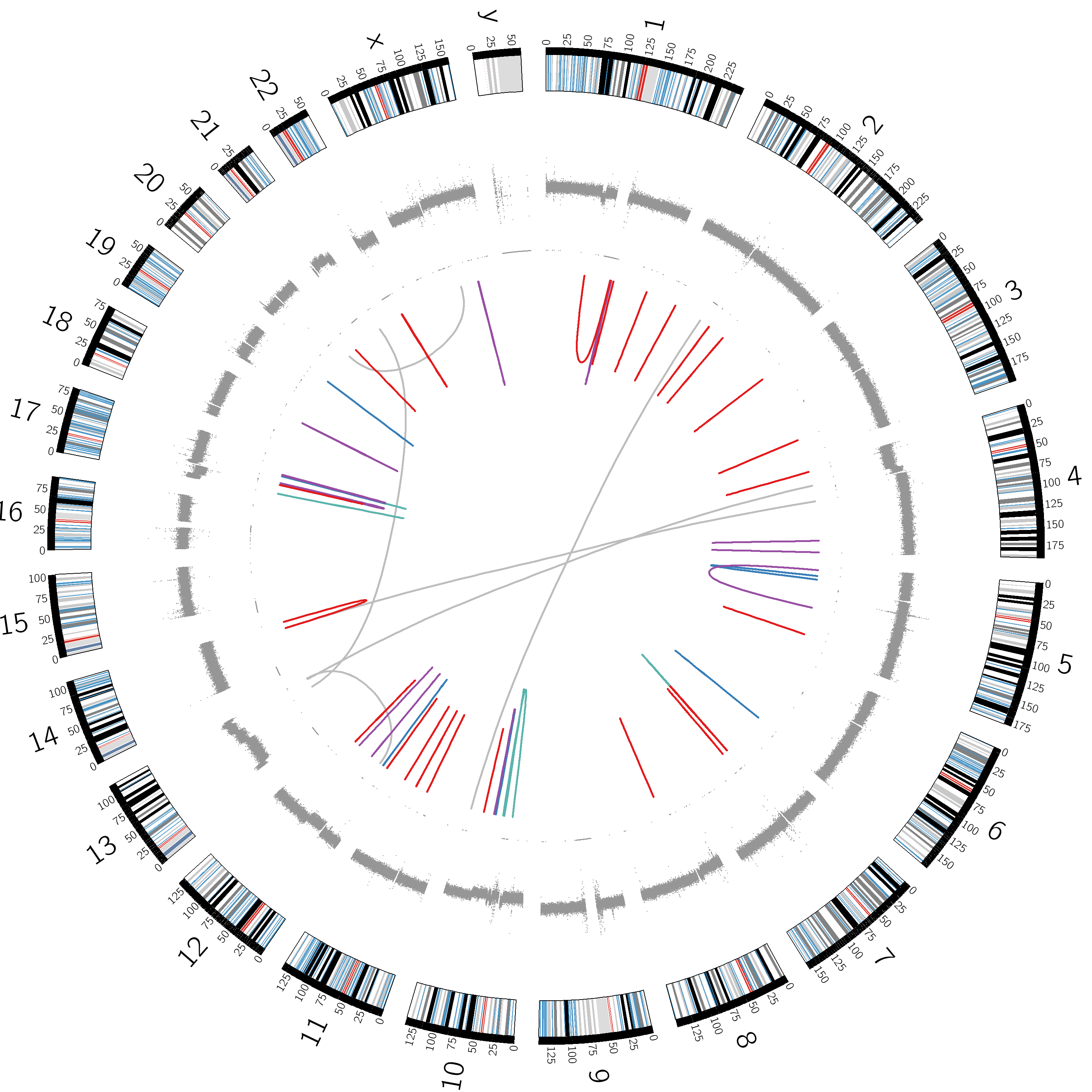

Supplement: Supplementary file 6 [file msb0011-0828-sd6.zip › png plots/BM1154.png]

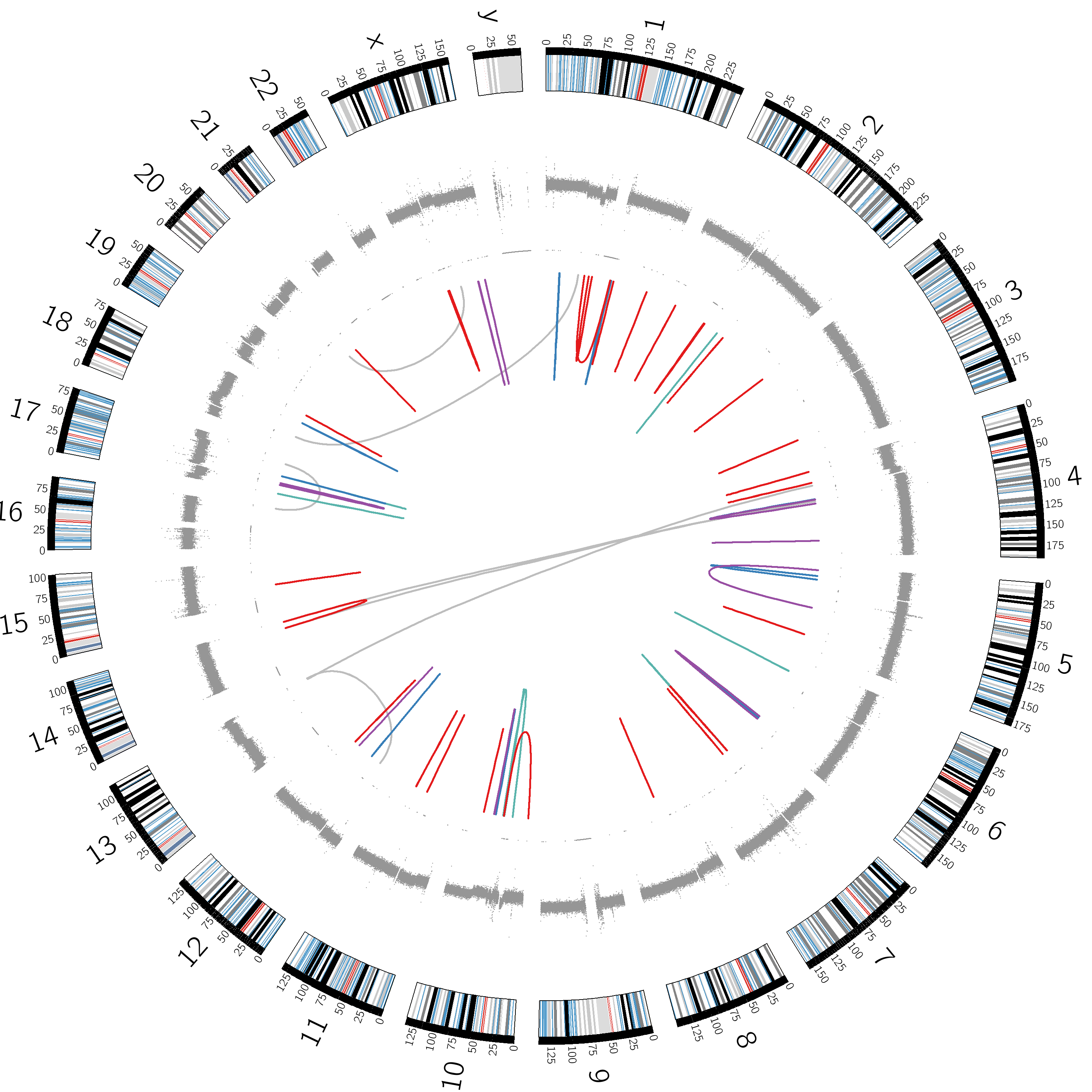

Supplement: Supplementary file 6 [file msb0011-0828-sd6.zip › png plots/BM1158.png]

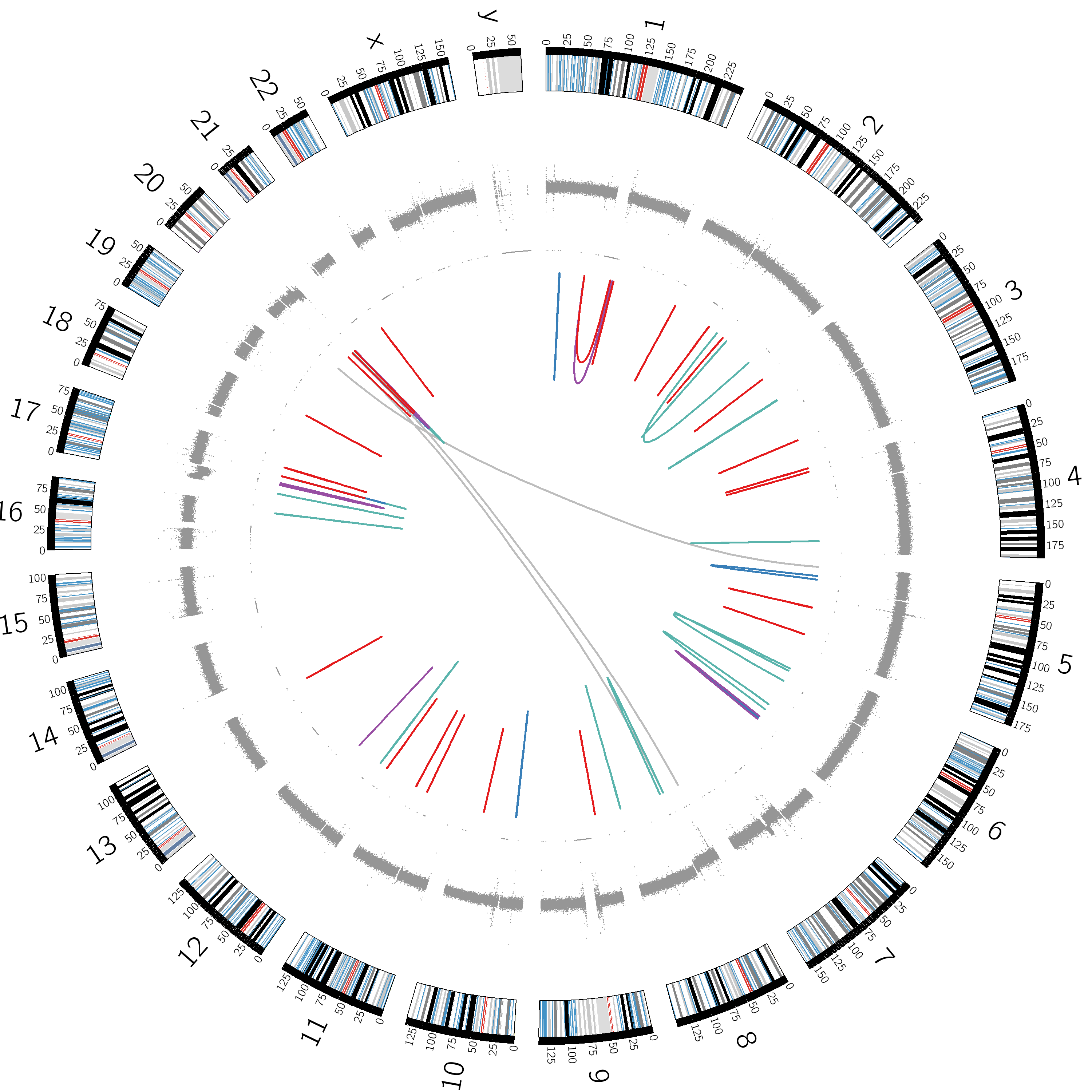

Supplement: Supplementary file 6 [file msb0011-0828-sd6.zip › png plots/BM1159.png]

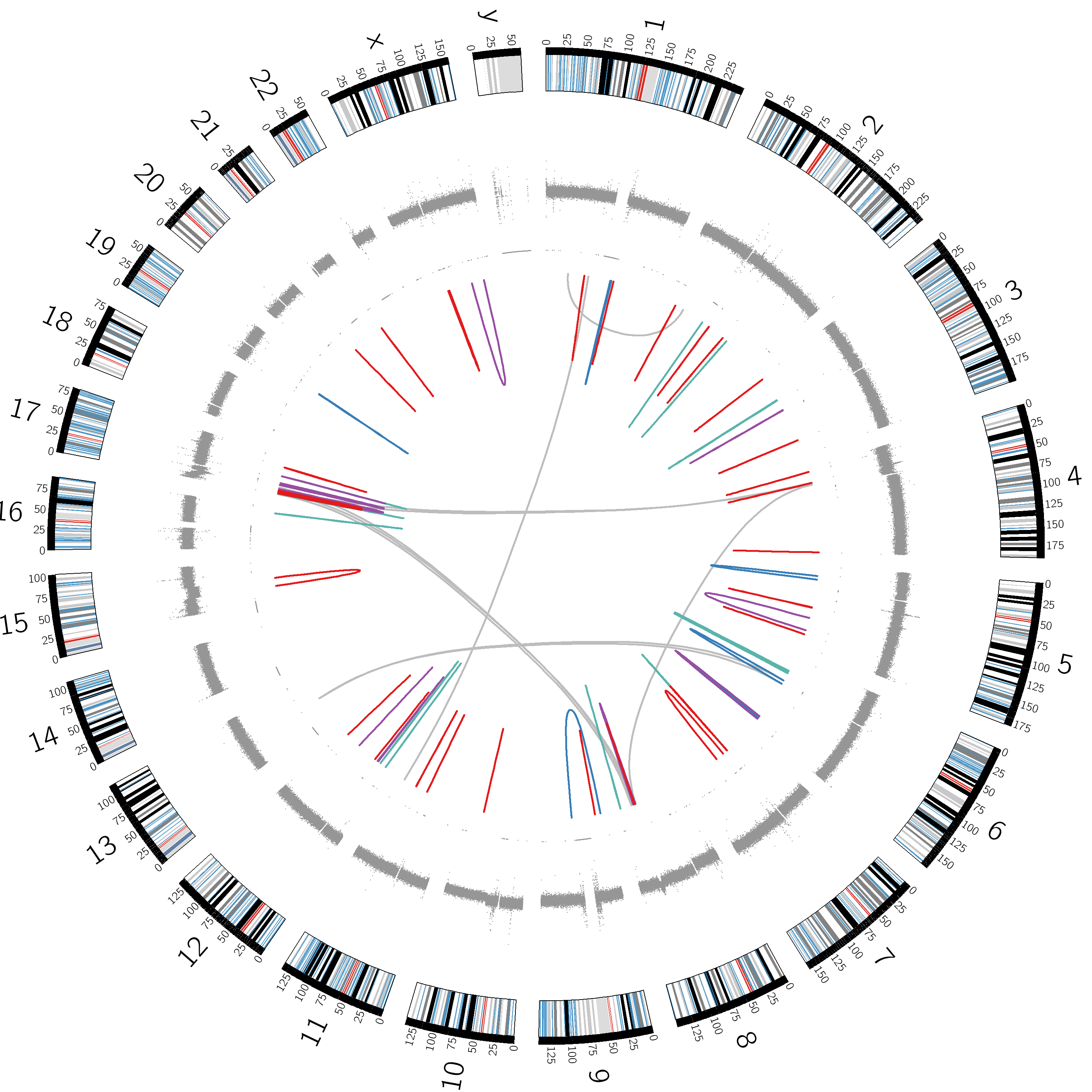

Supplement: Supplementary file 6 [file msb0011-0828-sd6.zip › png plots/BM1163.png]

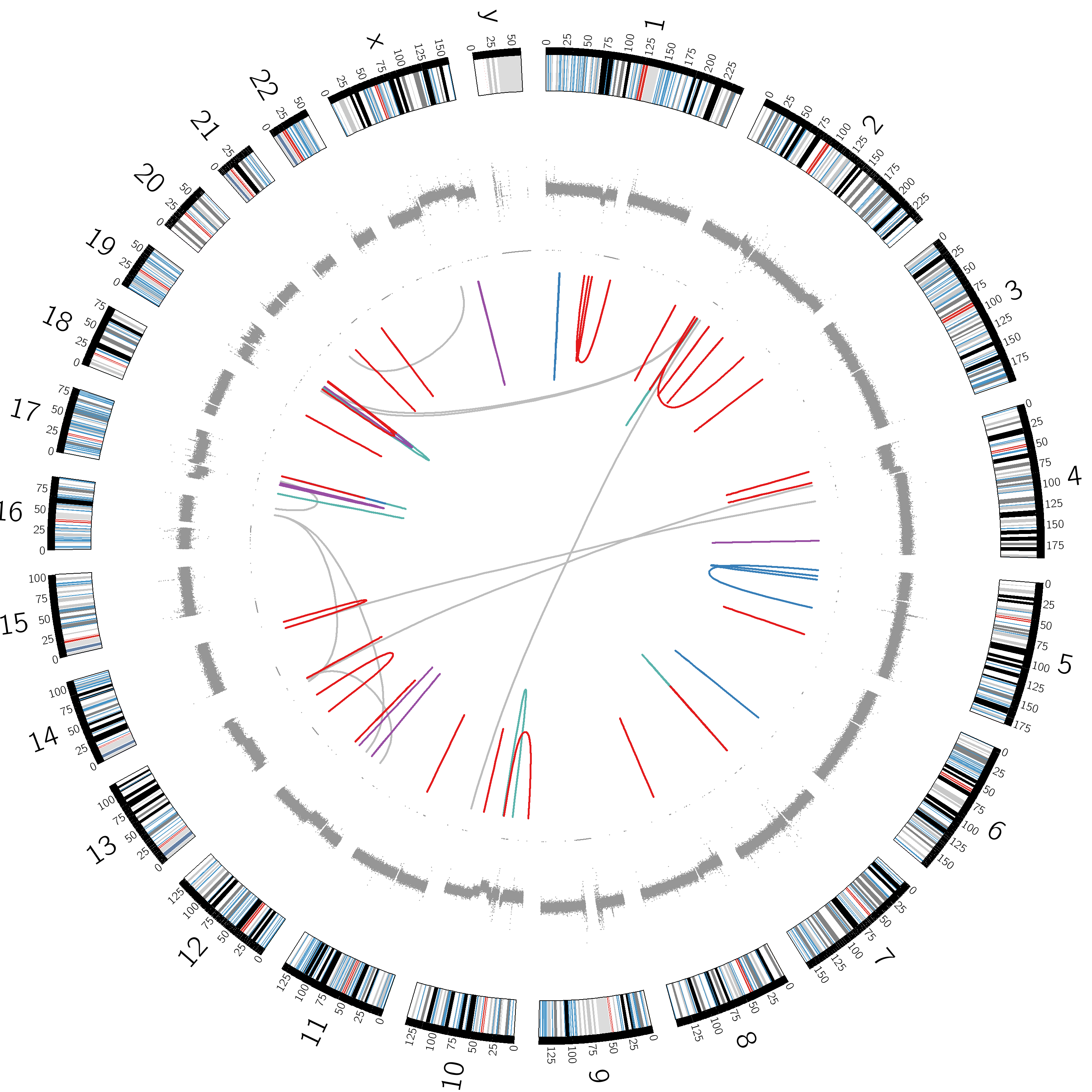

Supplement: Supplementary file 6 [file msb0011-0828-sd6.zip › png plots/BM1166.png]

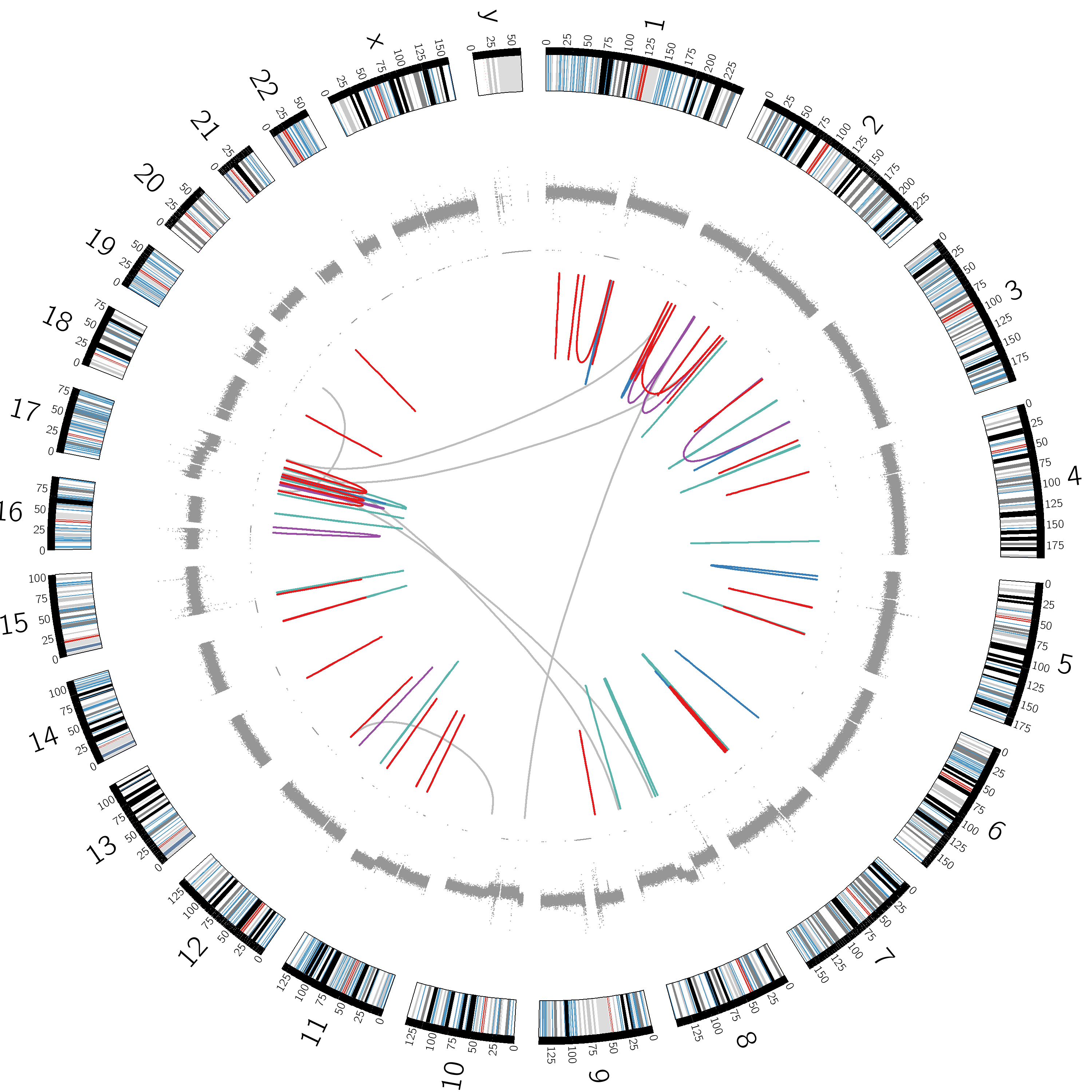

Supplement: Supplementary file 6 [file msb0011-0828-sd6.zip › png plots/BM173.png]

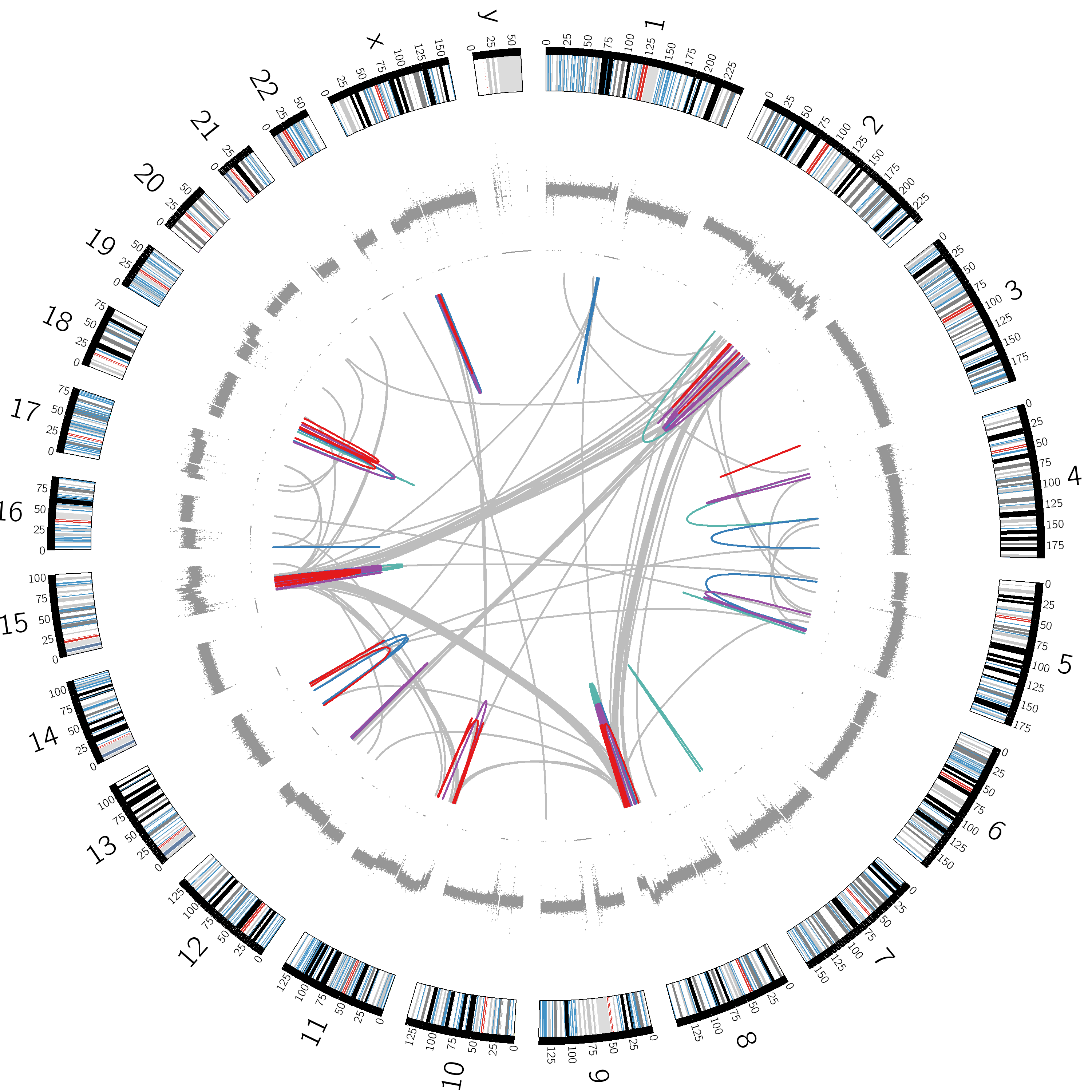

Supplement: Supplementary file 6 [file msb0011-0828-sd6.zip › png plots/BM175.png]

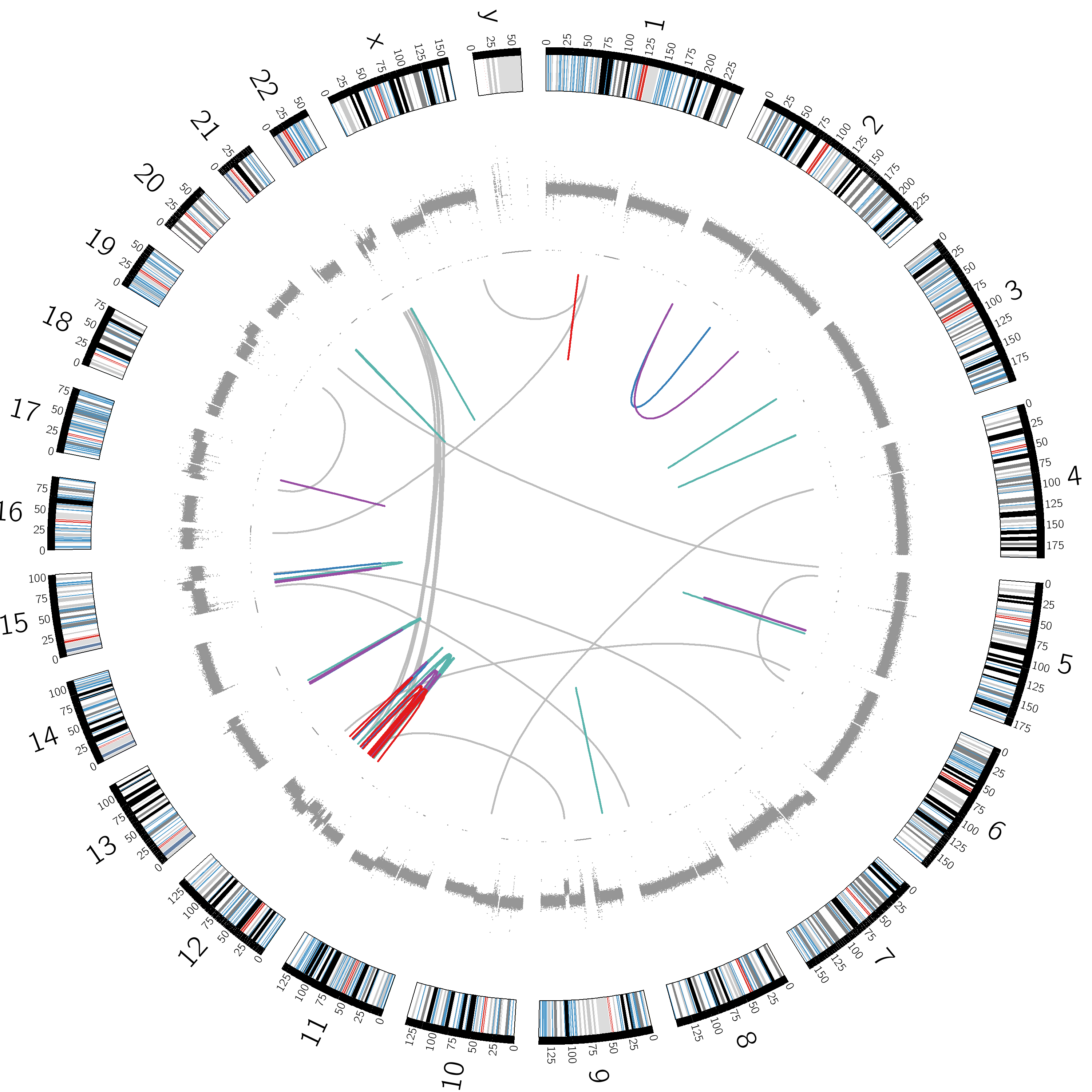

Supplement: Supplementary file 6 [file msb0011-0828-sd6.zip › png plots/BM178.png]

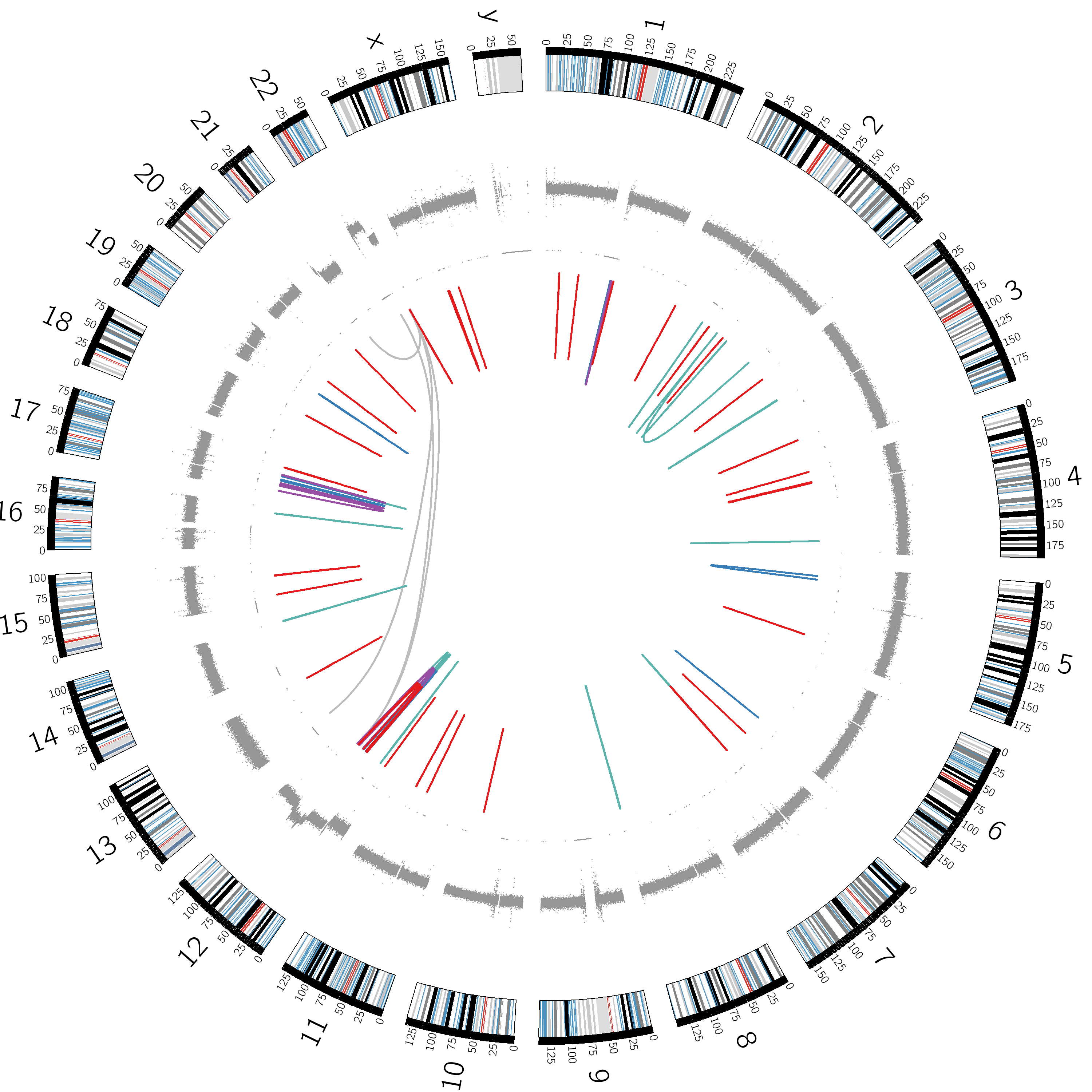

Supplement: Supplementary file 6 [file msb0011-0828-sd6.zip › png plots/BM237.png]

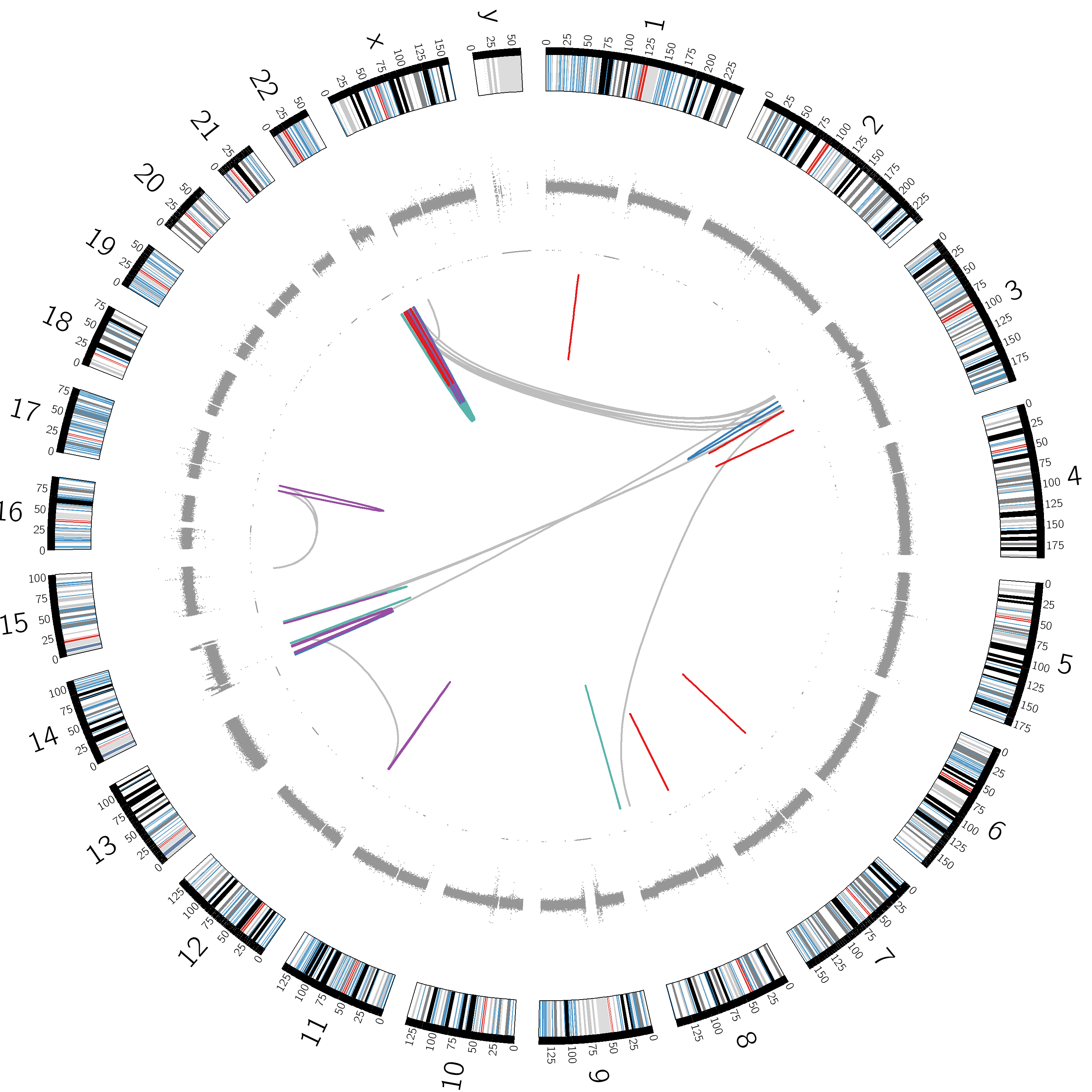

Supplement: Supplementary file 6 [file msb0011-0828-sd6.zip › png plots/BM263.png]

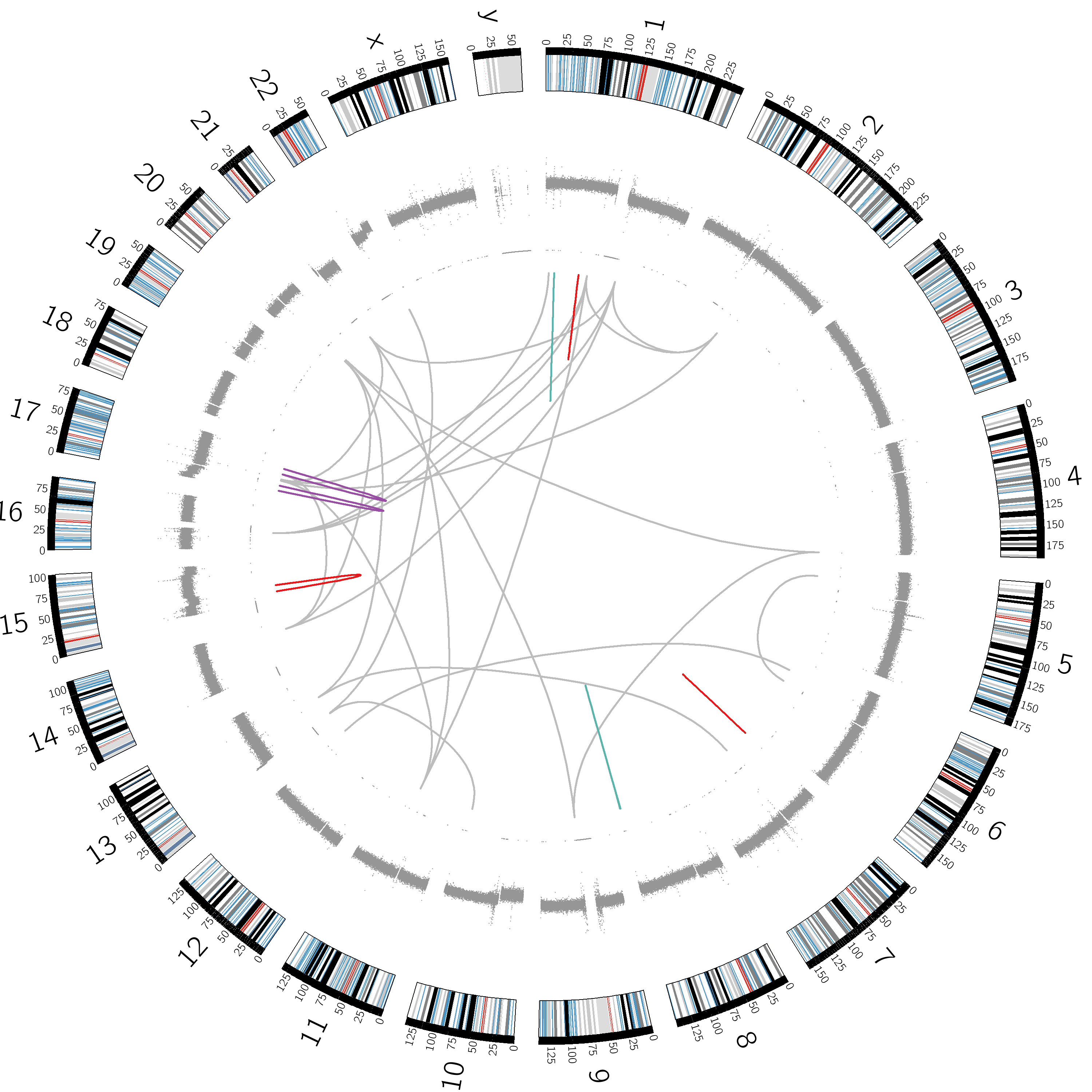

Supplement: Supplementary file 6 [file msb0011-0828-sd6.zip › png plots/BM597.png]

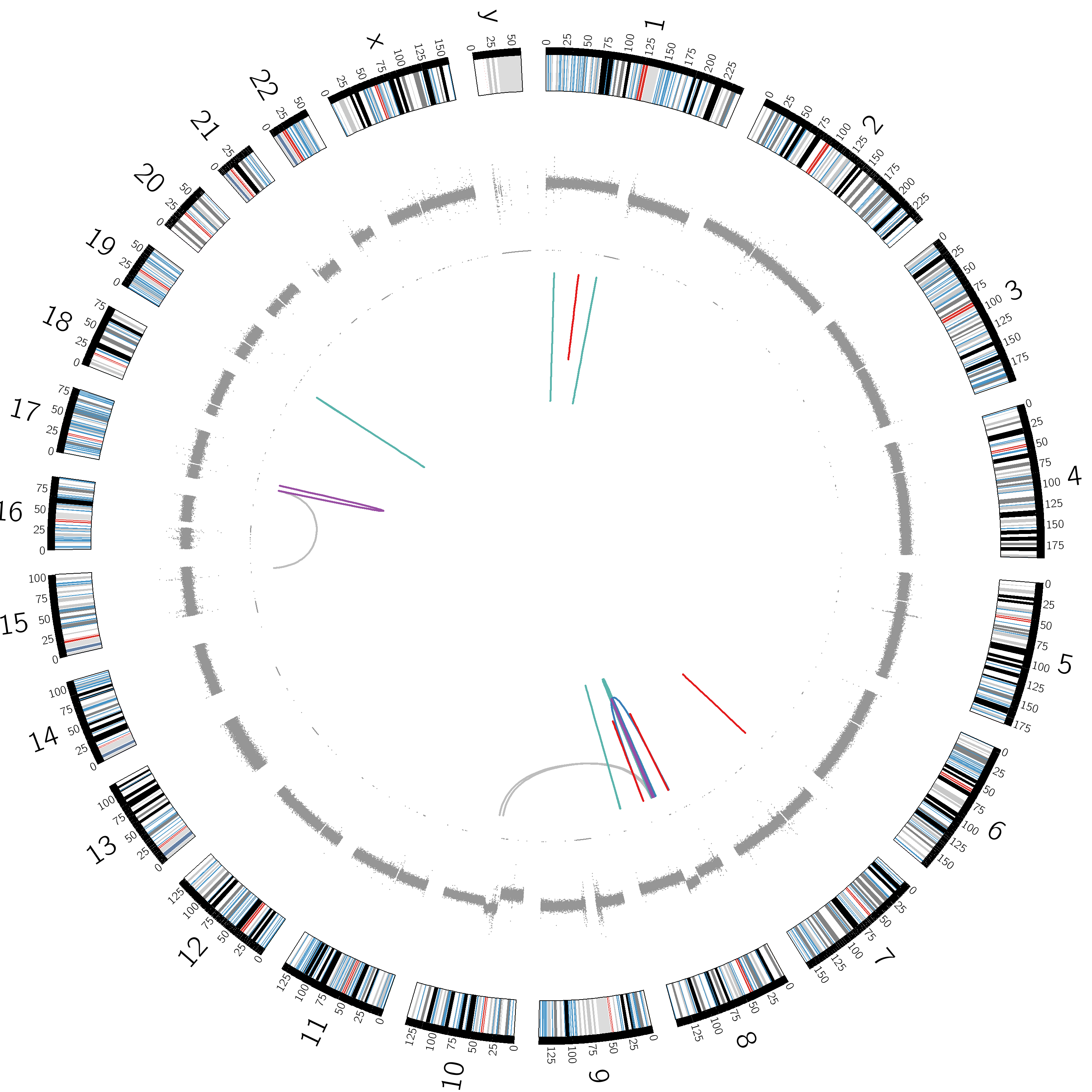

Supplement: Supplementary file 6 [file msb0011-0828-sd6.zip › png plots/BM601.png]

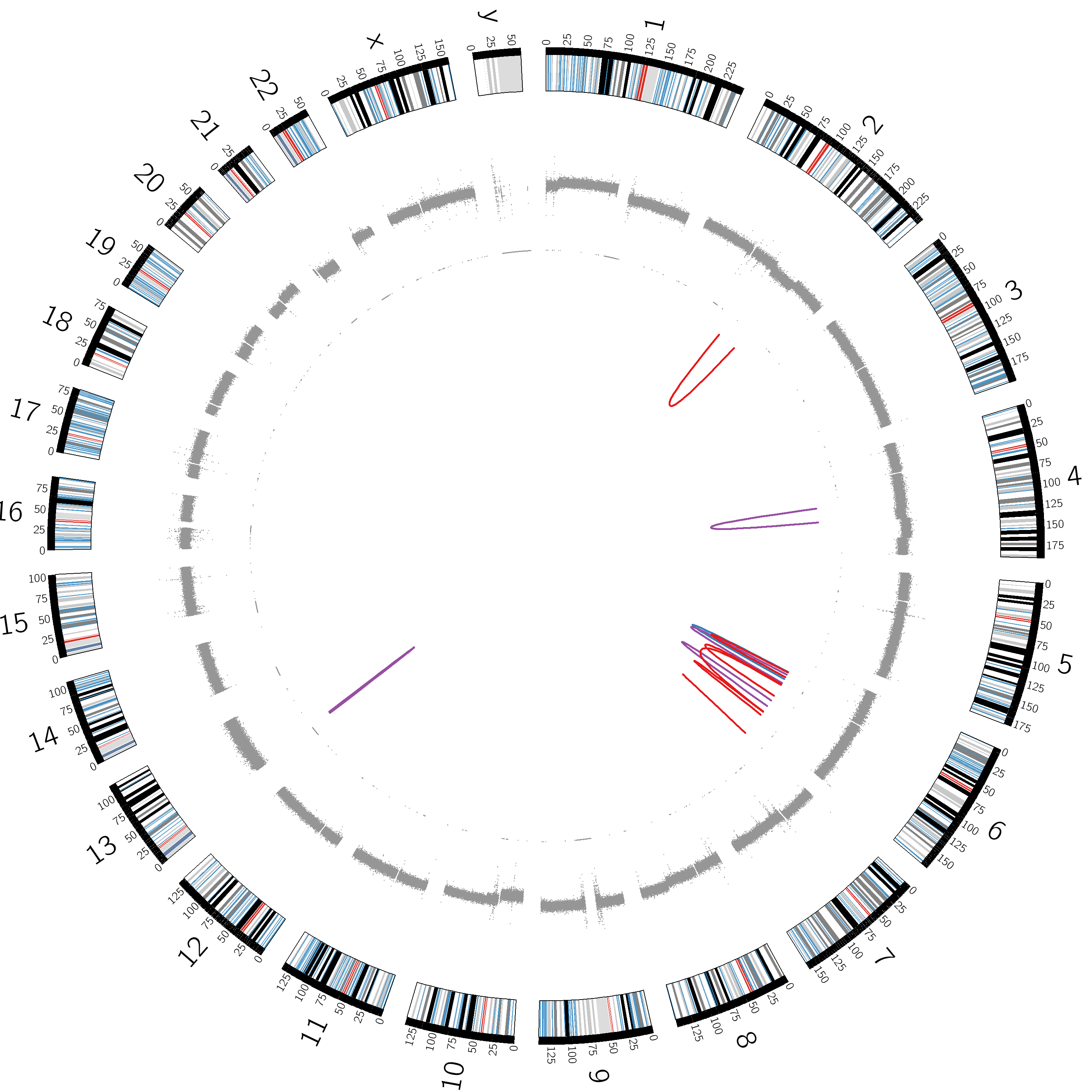

Supplement: Supplementary file 6 [file msb0011-0828-sd6.zip › png plots/BM605.png]

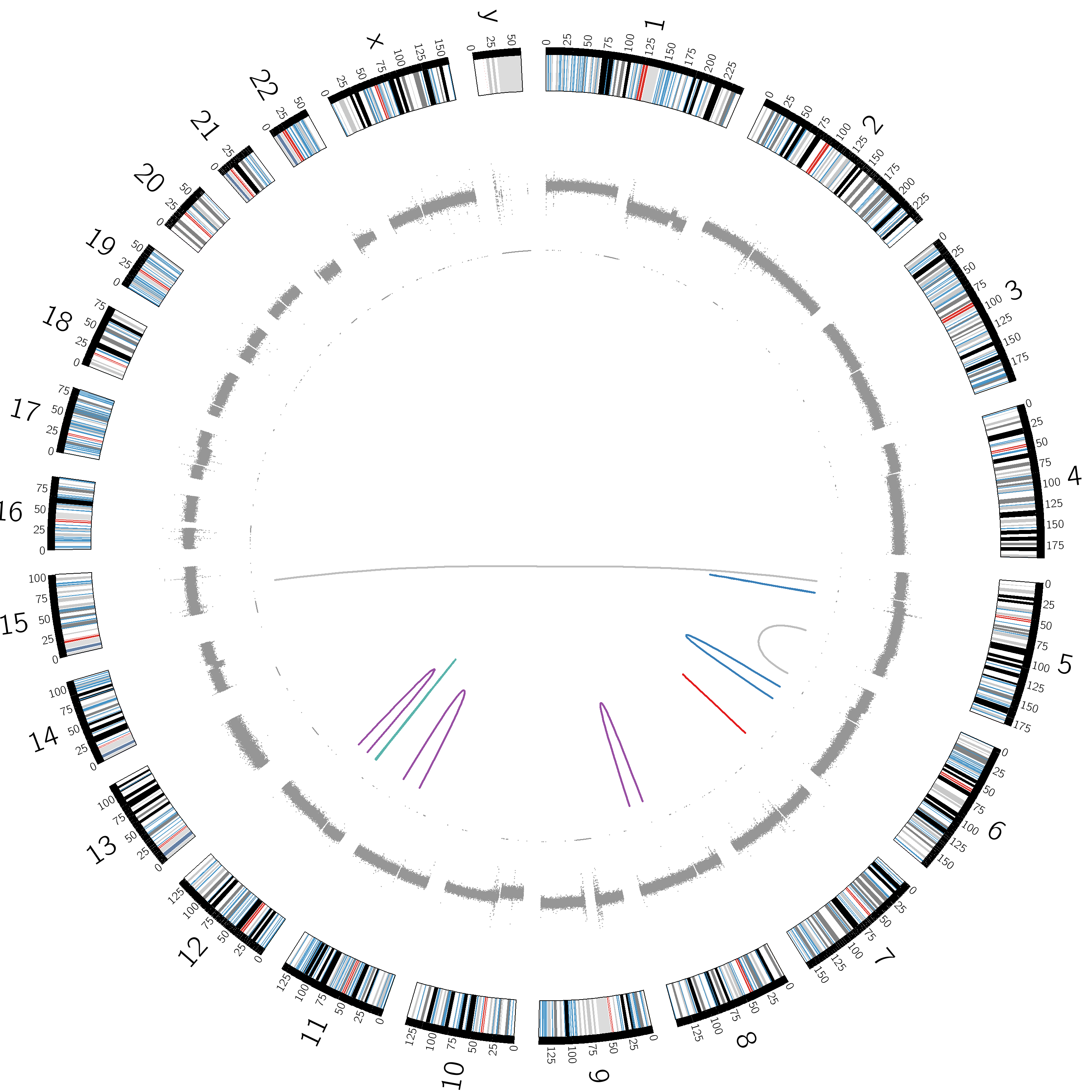

Supplement: Supplementary file 6 [file msb0011-0828-sd6.zip › png plots/BM606.png]

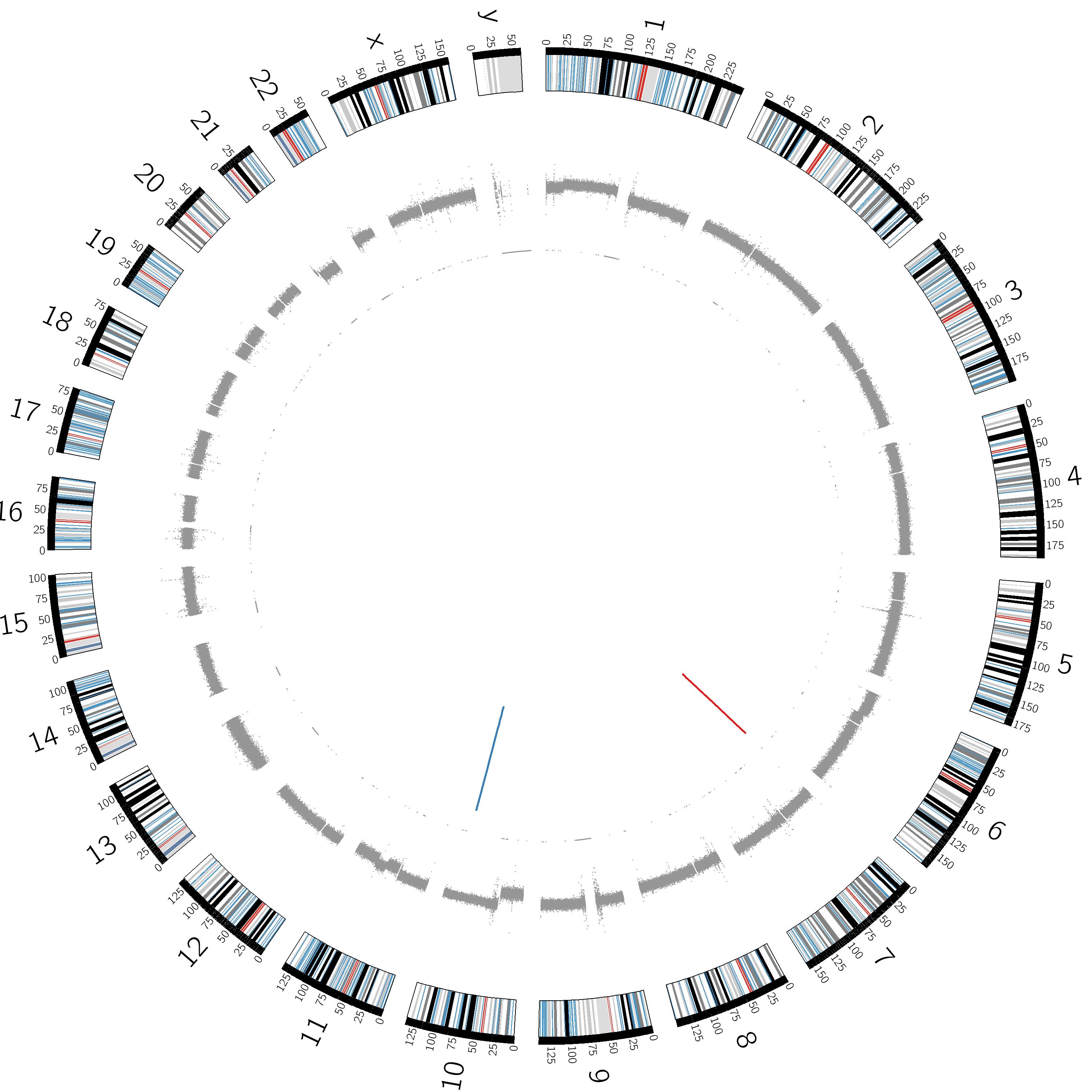

Supplement: Supplementary file 6 [file msb0011-0828-sd6.zip › png plots/BM610.png]

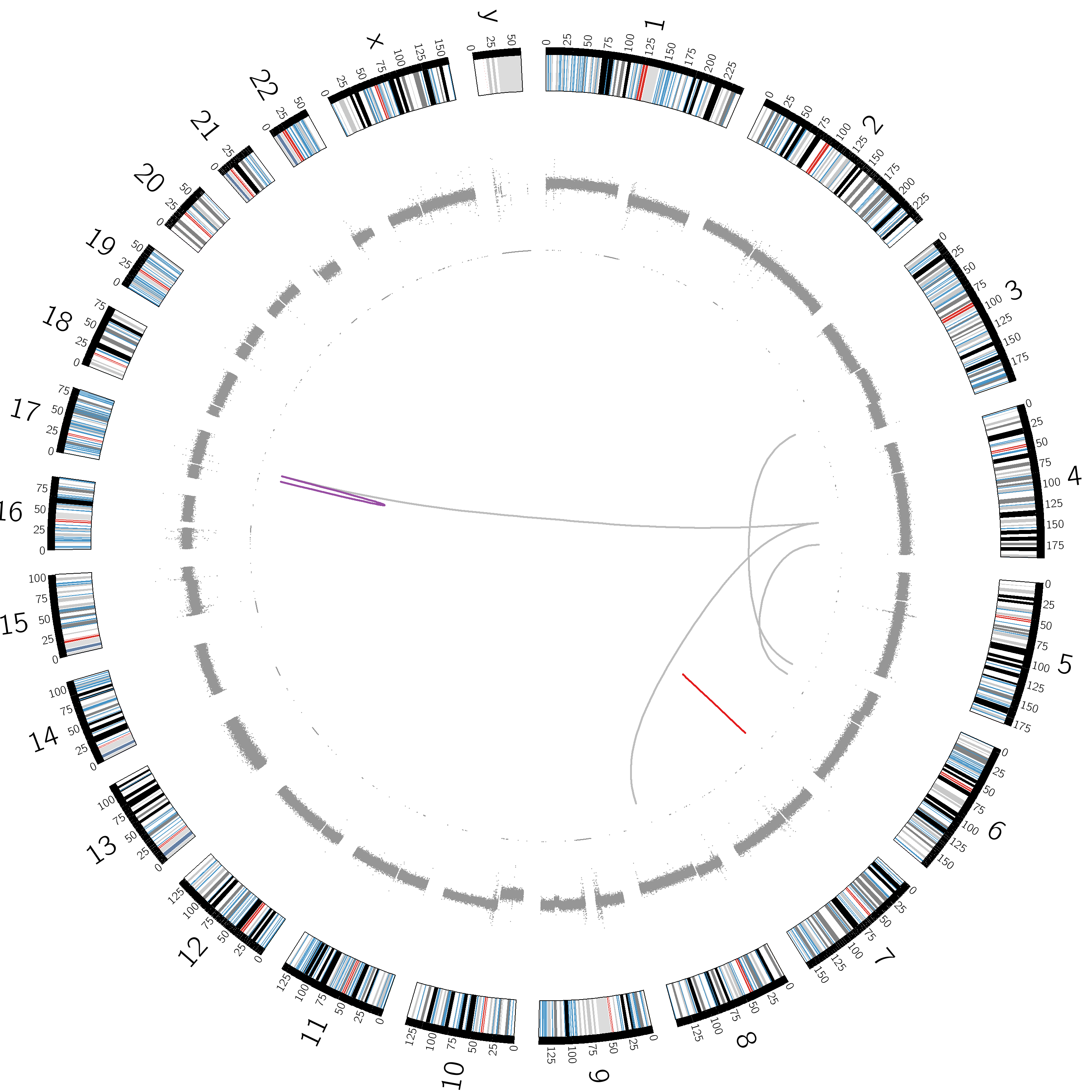

Supplement: Supplementary file 6 [file msb0011-0828-sd6.zip › png plots/BM619.png]

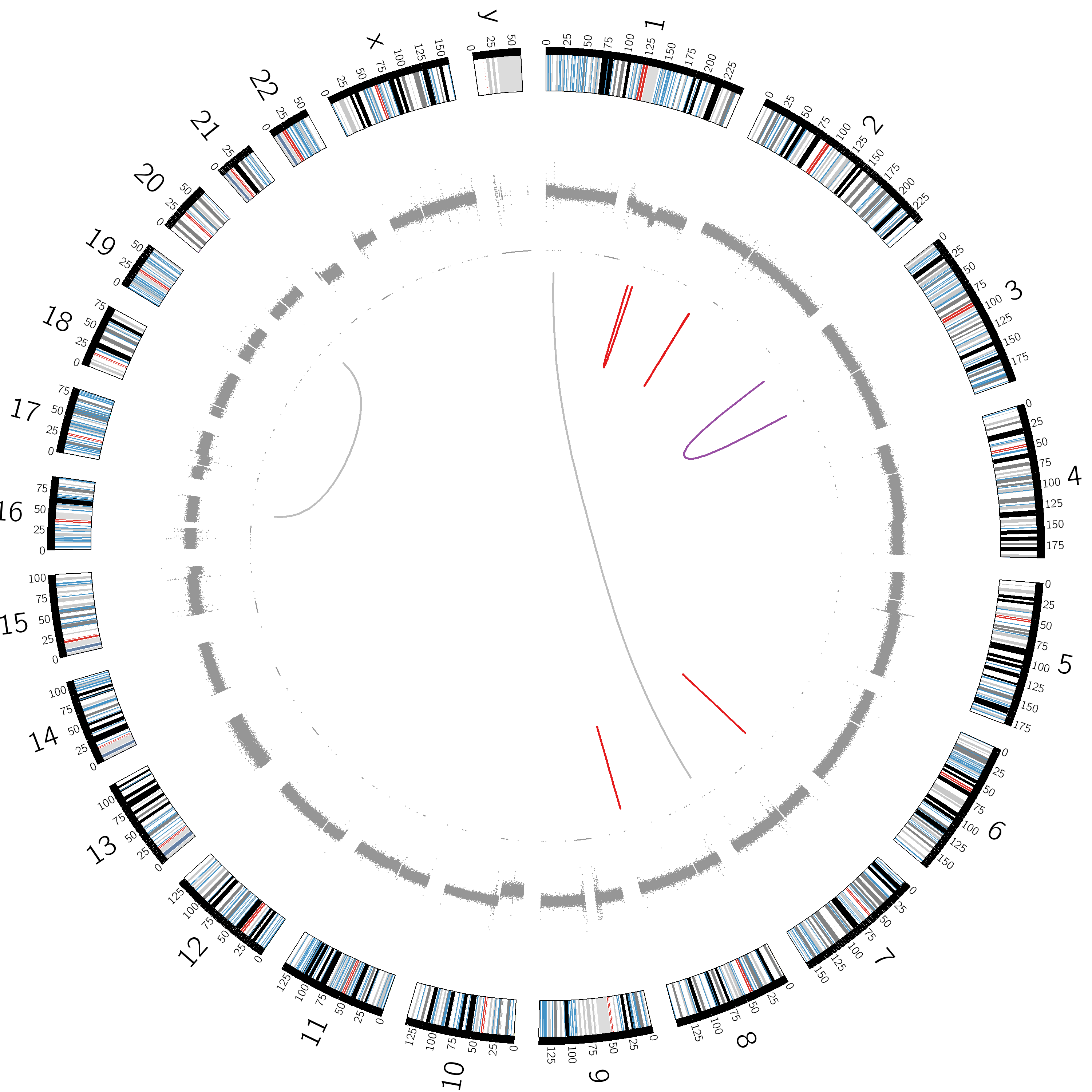

Supplement: Supplementary file 6 [file msb0011-0828-sd6.zip › png plots/BM625.png]

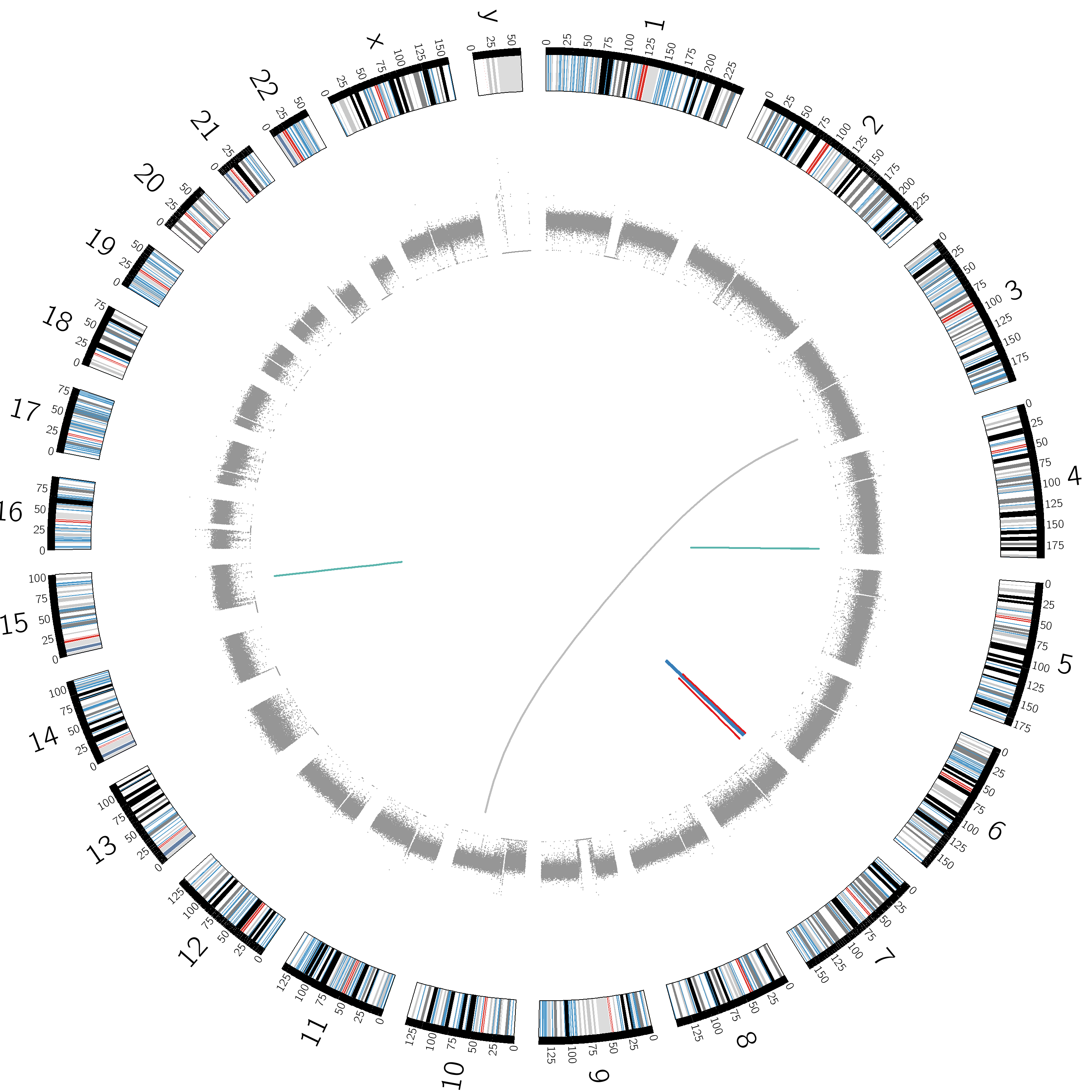

Supplement: Supplementary file 6 [file msb0011-0828-sd6.zip › png plots/BM642.png]

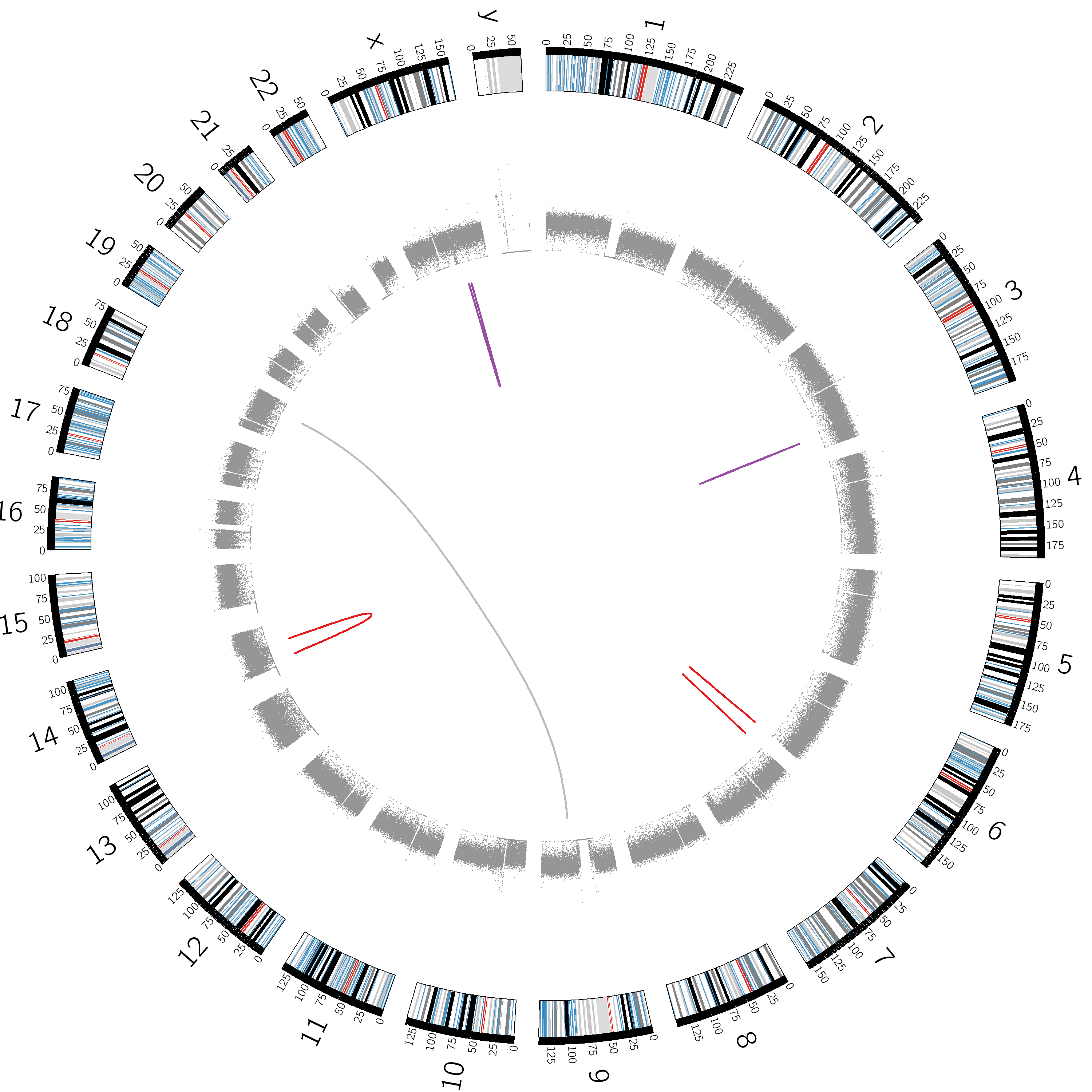

Supplement: Supplementary file 6 [file msb0011-0828-sd6.zip › png plots/BM647.png]

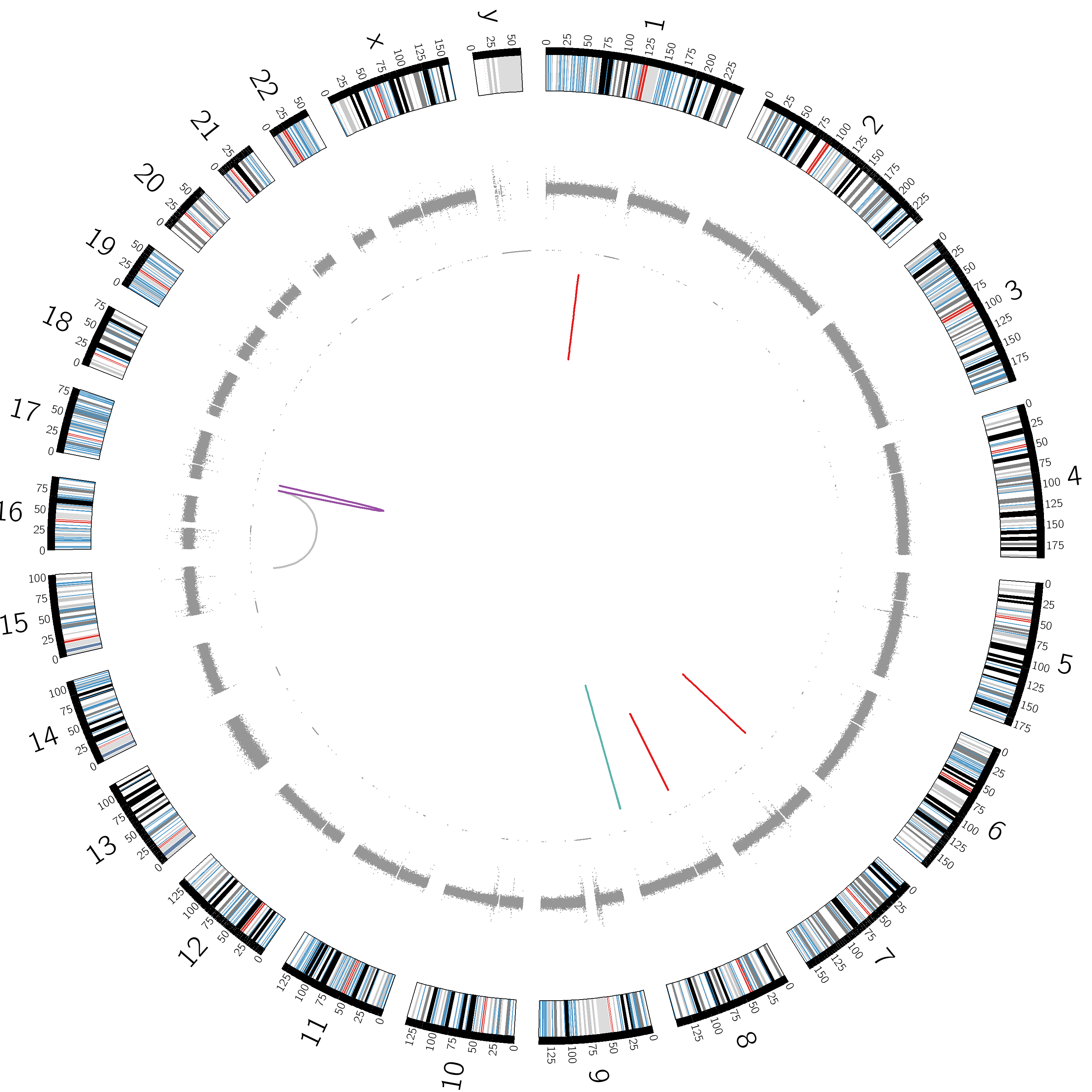

Supplement: Supplementary file 6 [file msb0011-0828-sd6.zip › png plots/BM673.png]

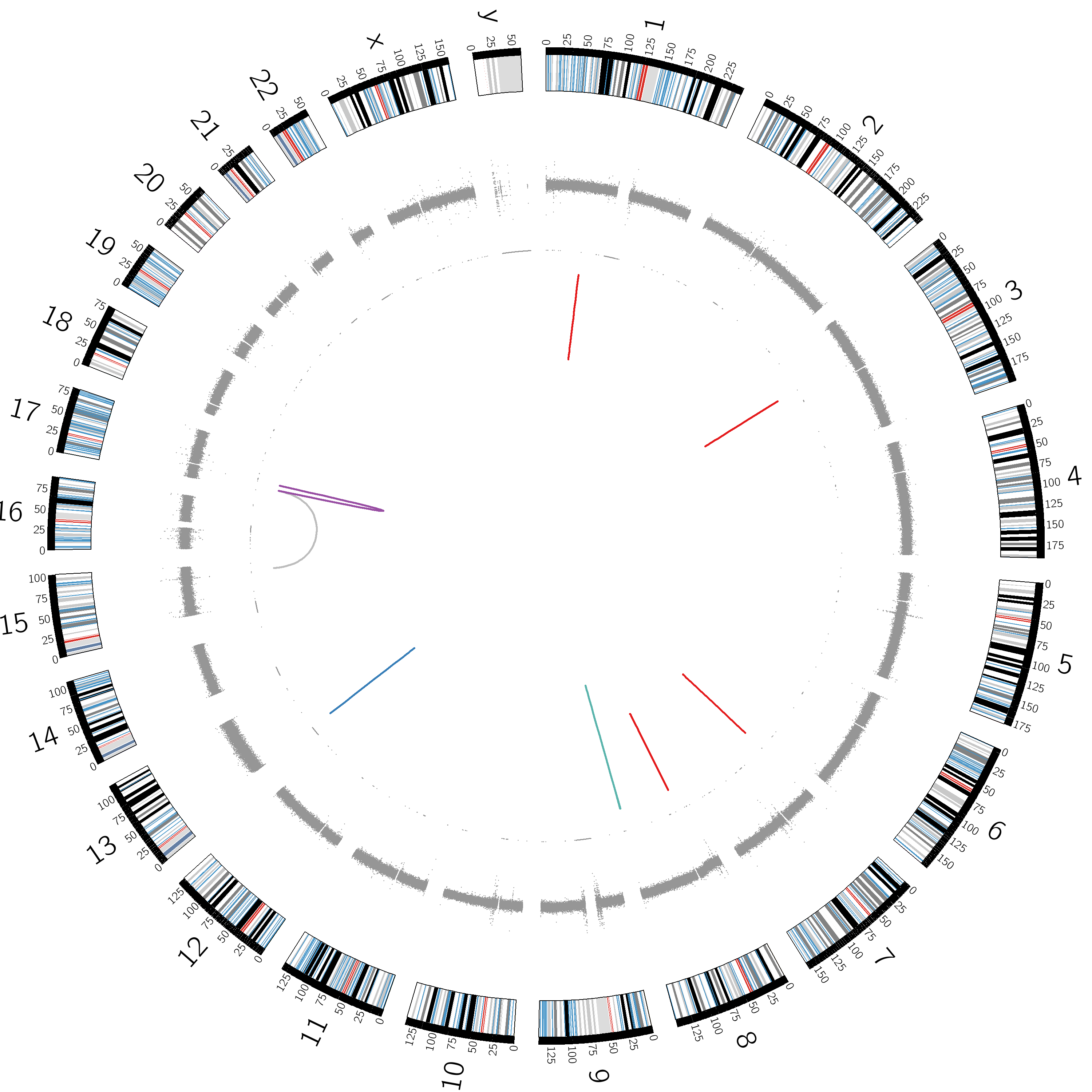

Supplement: Supplementary file 6 [file msb0011-0828-sd6.zip › png plots/BM674.png]

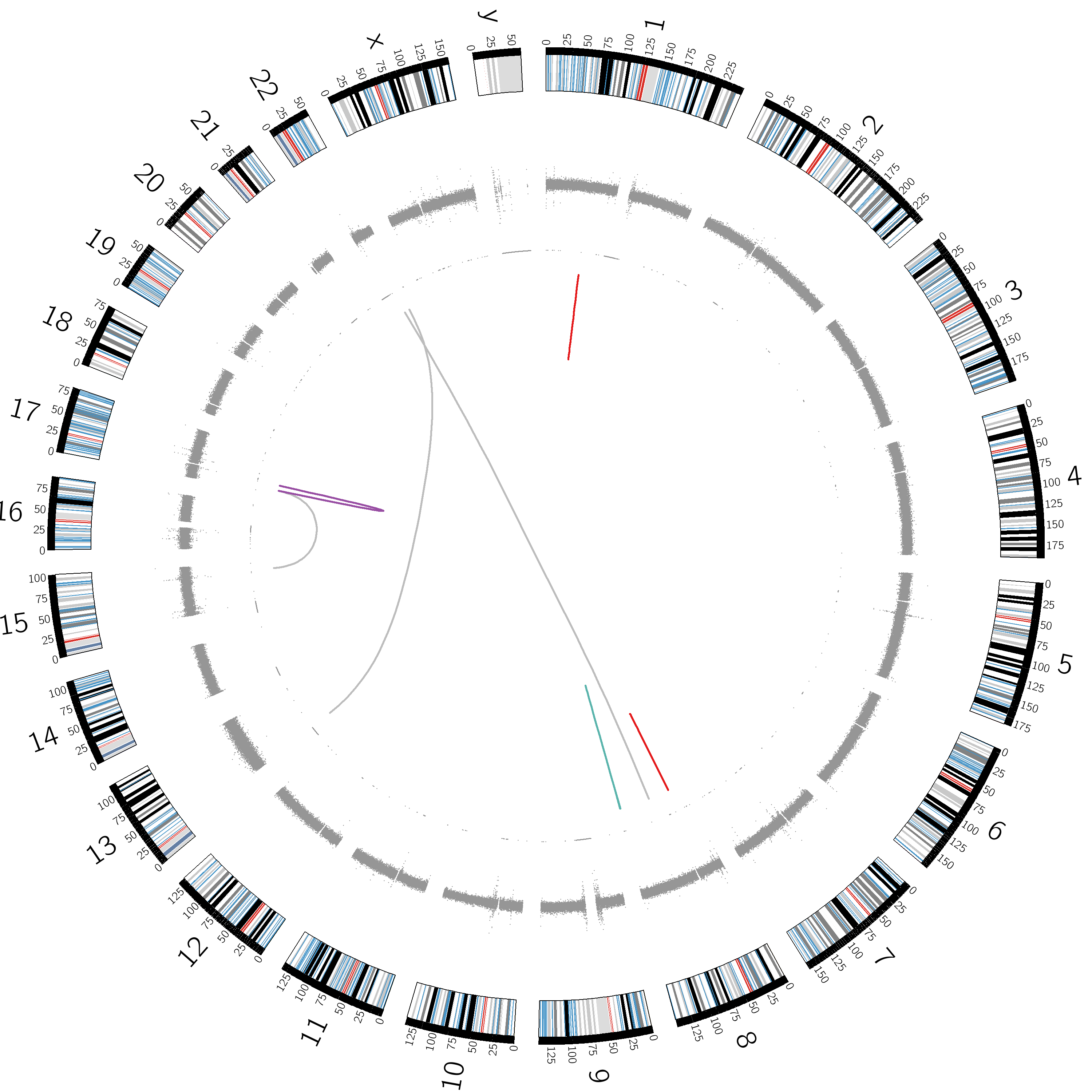

Supplement: Supplementary file 6 [file msb0011-0828-sd6.zip › png plots/BM675.png]

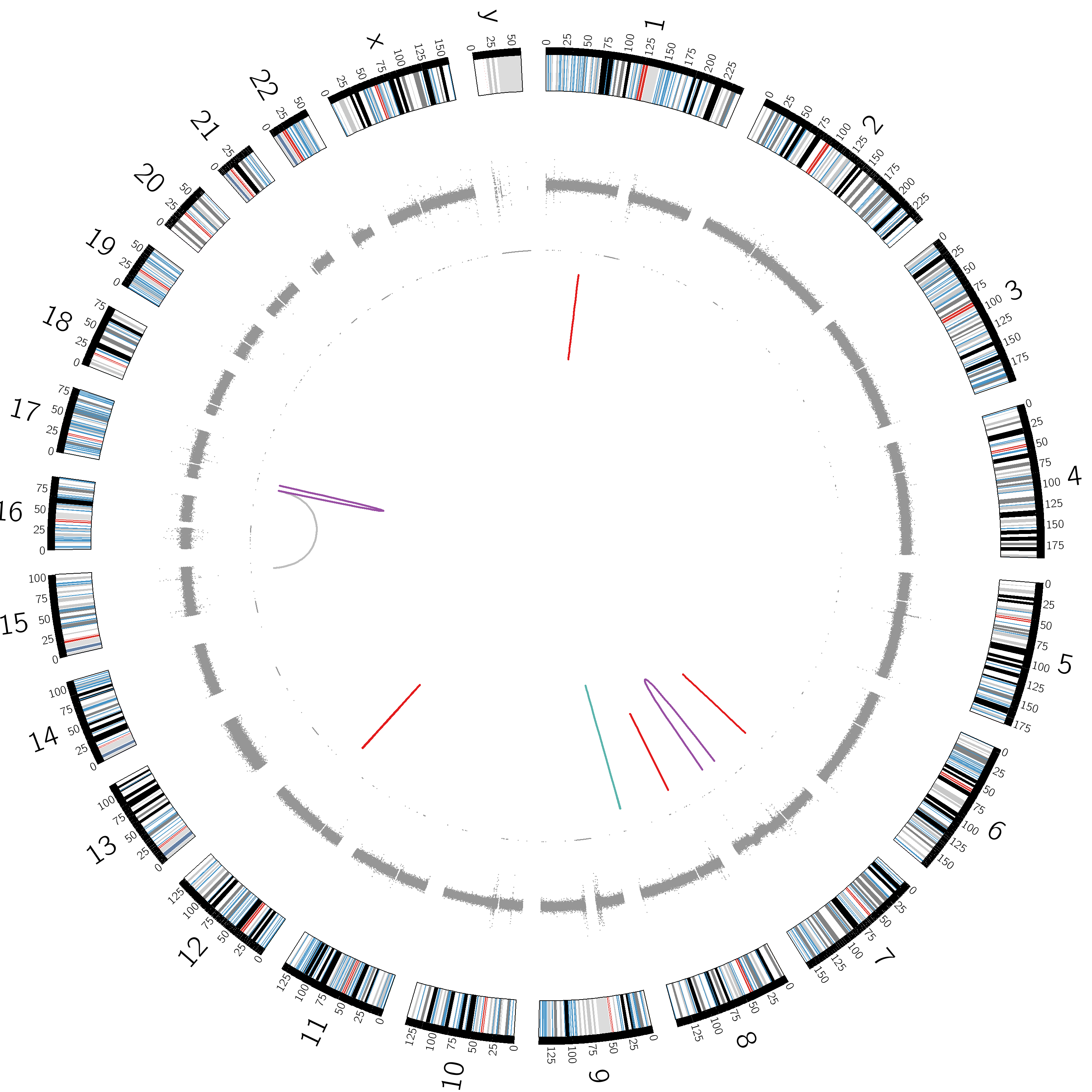

Supplement: Supplementary file 6 [file msb0011-0828-sd6.zip › png plots/BM676.png]

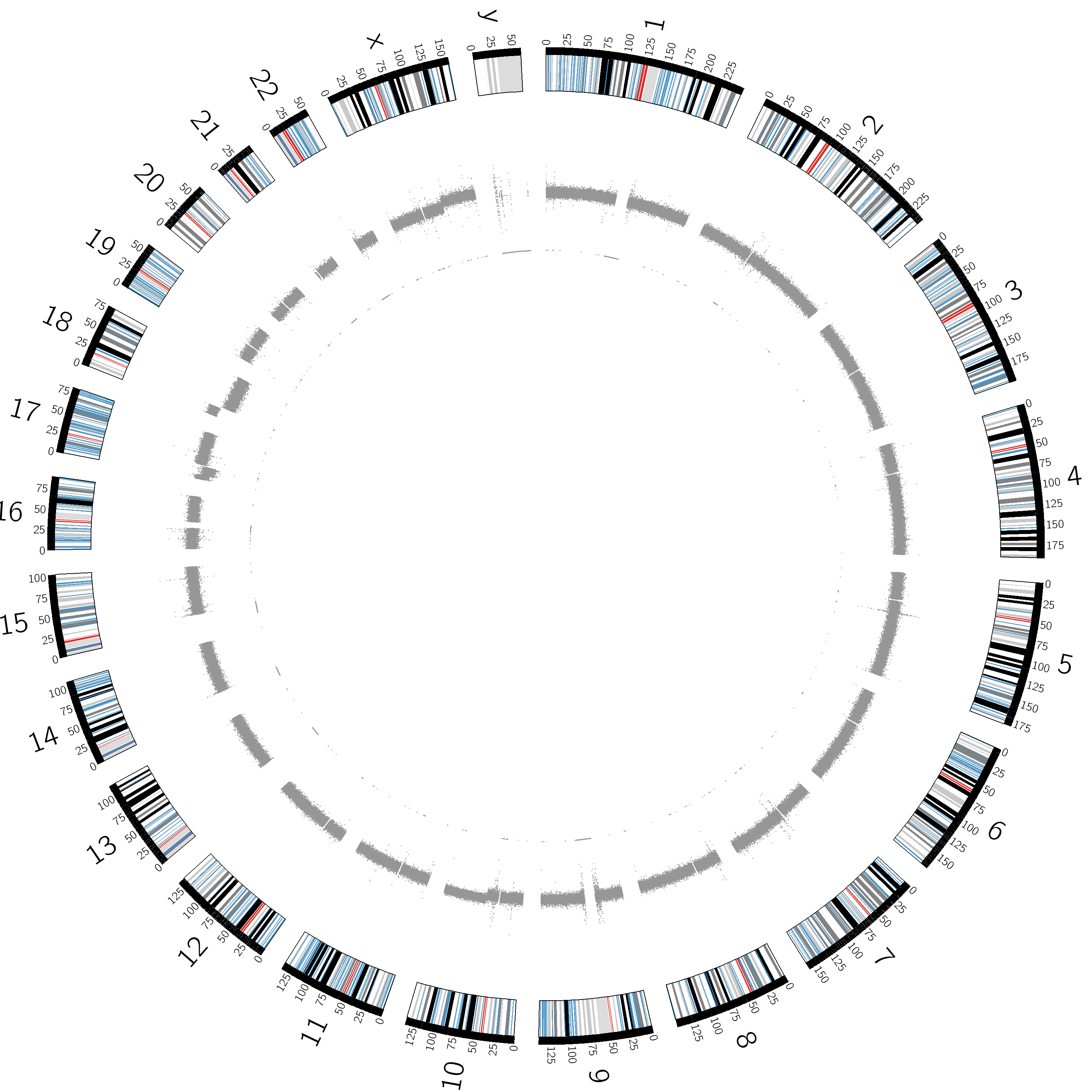

Supplement: Supplementary file 6 [file msb0011-0828-sd6.zip › png plots/BM678.png]

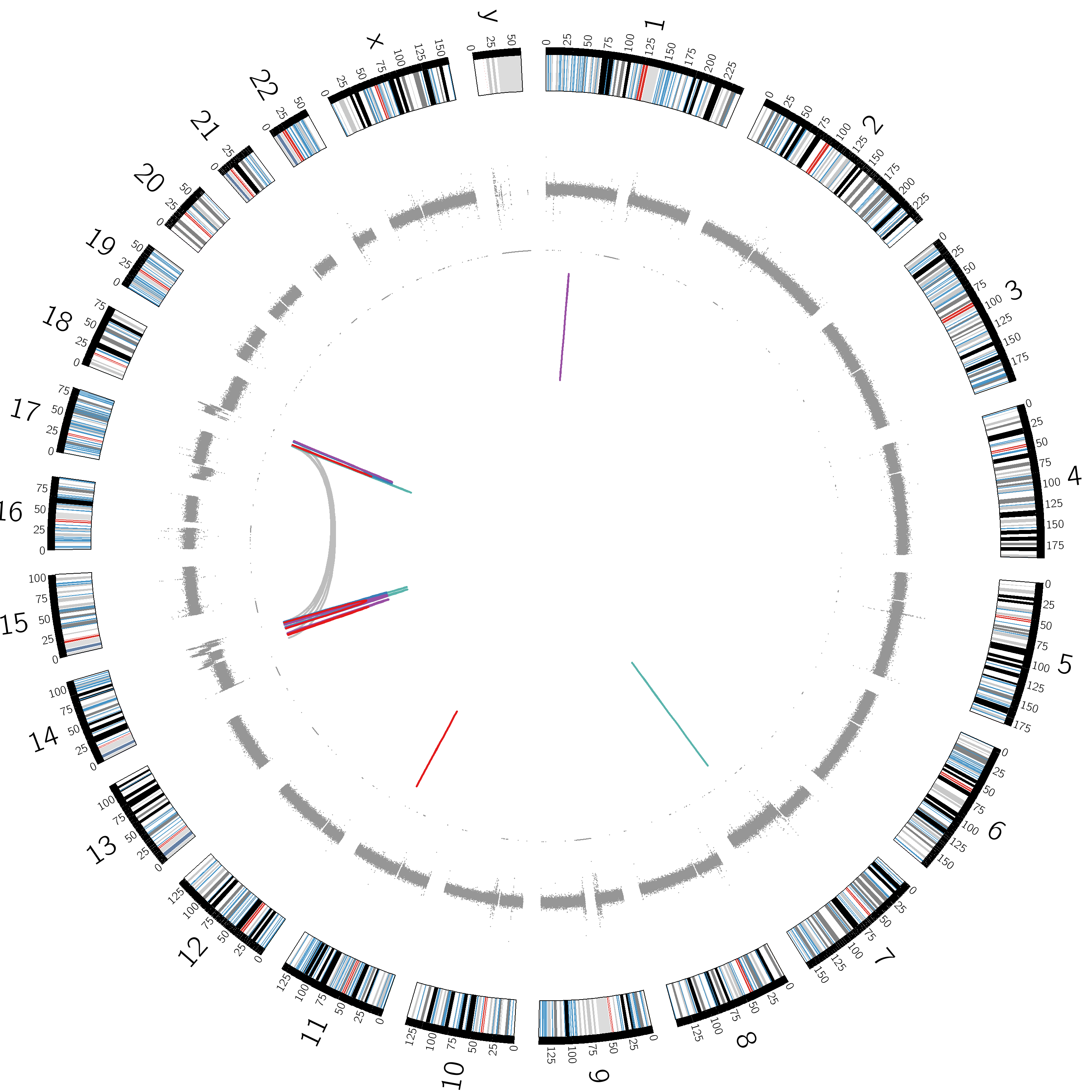

Supplement: Supplementary file 6 [file msb0011-0828-sd6.zip › png plots/BM694.png]

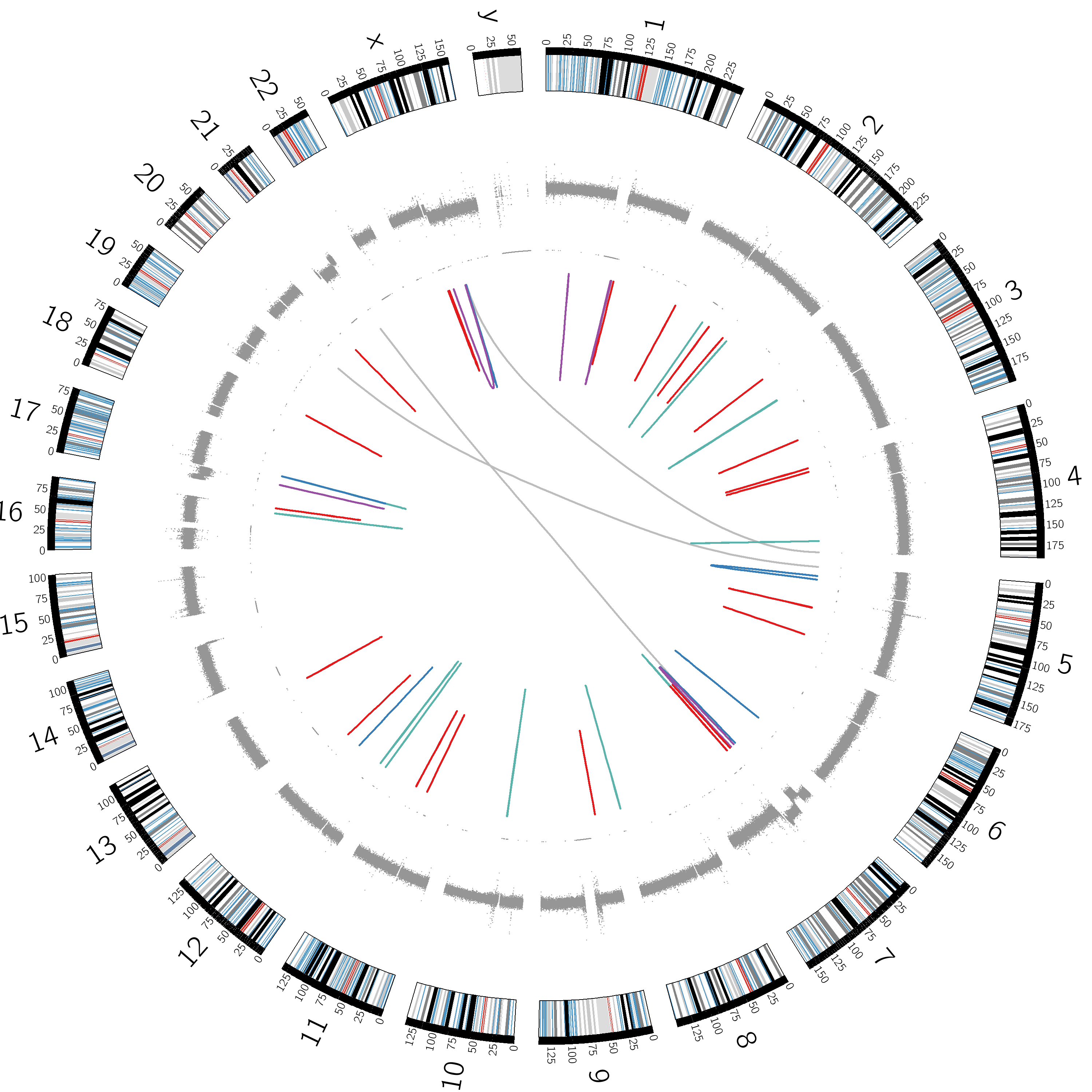

Supplement: Supplementary file 6 [file msb0011-0828-sd6.zip › png plots/BM696.png]

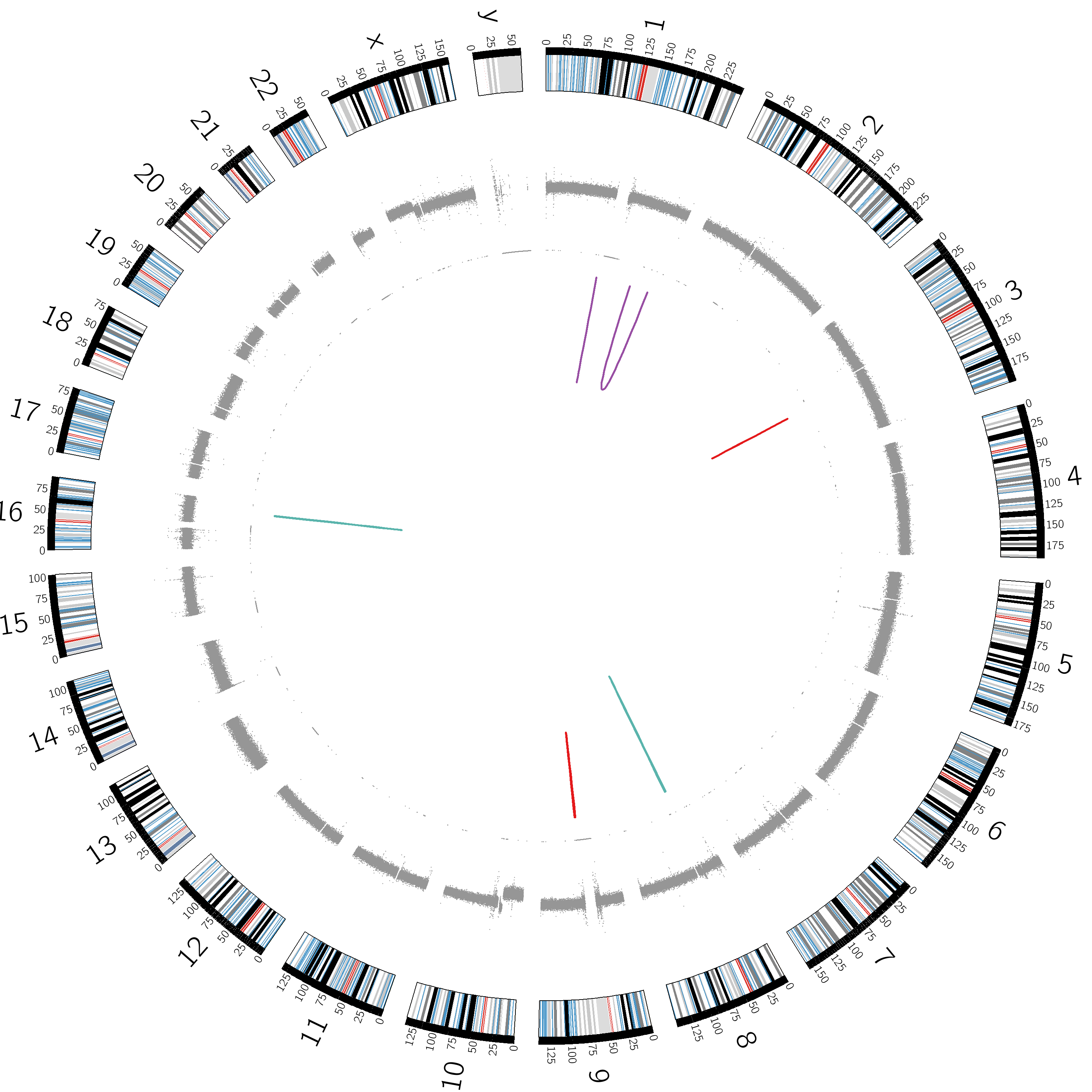

Supplement: Supplementary file 6 [file msb0011-0828-sd6.zip › png plots/BM766.png]

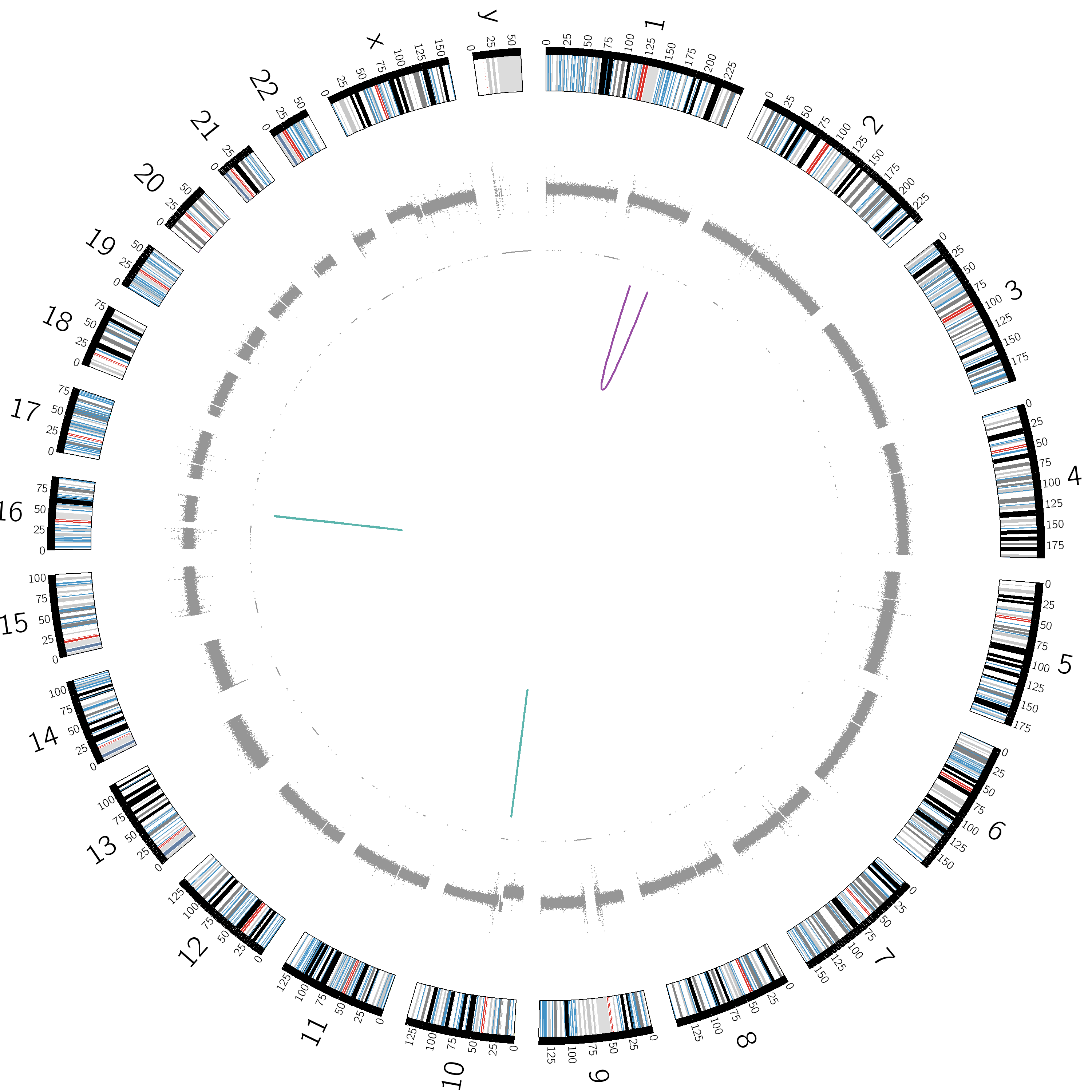

Supplement: Supplementary file 6 [file msb0011-0828-sd6.zip › png plots/BM767.png]

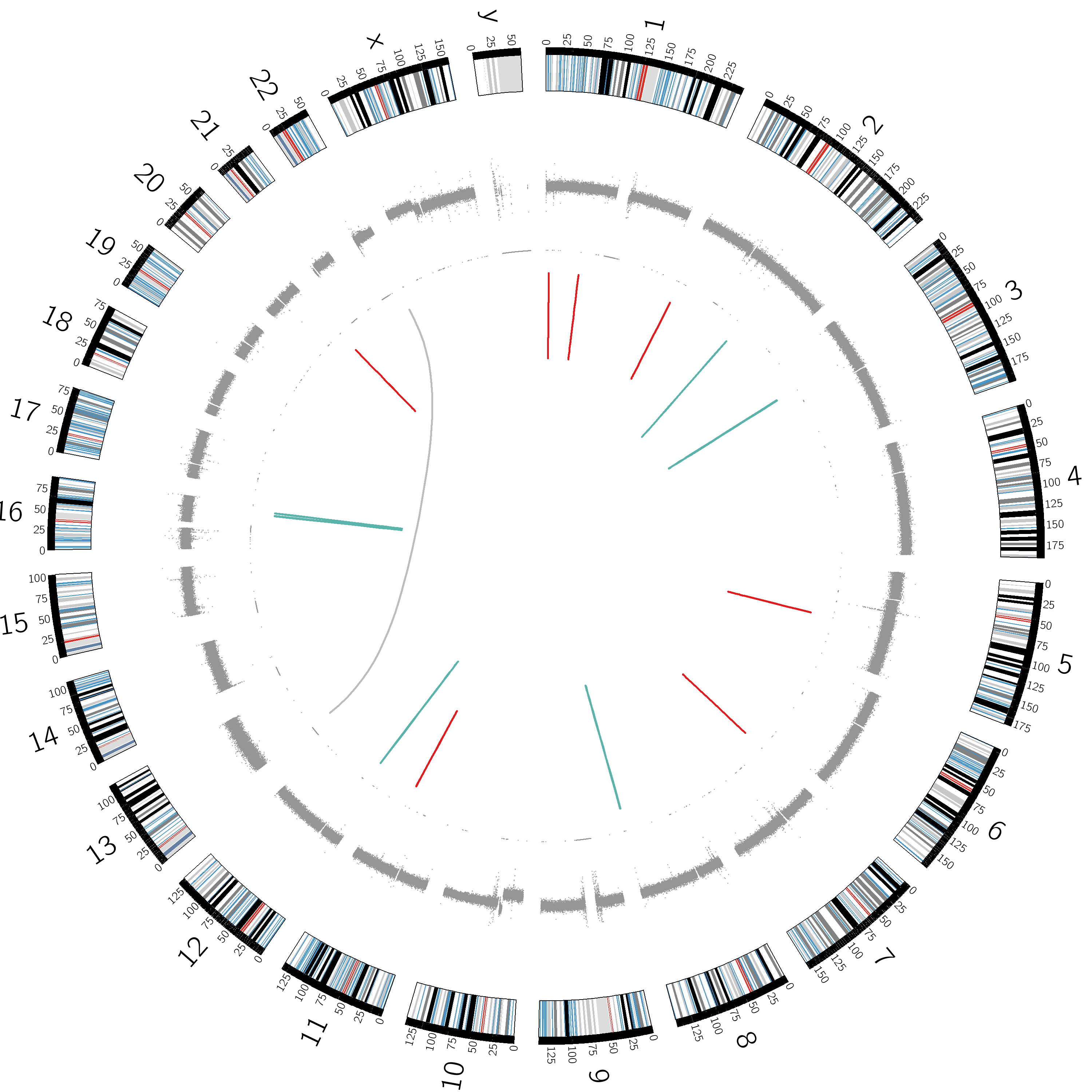

Supplement: Supplementary file 6 [file msb0011-0828-sd6.zip › png plots/BM768.png]

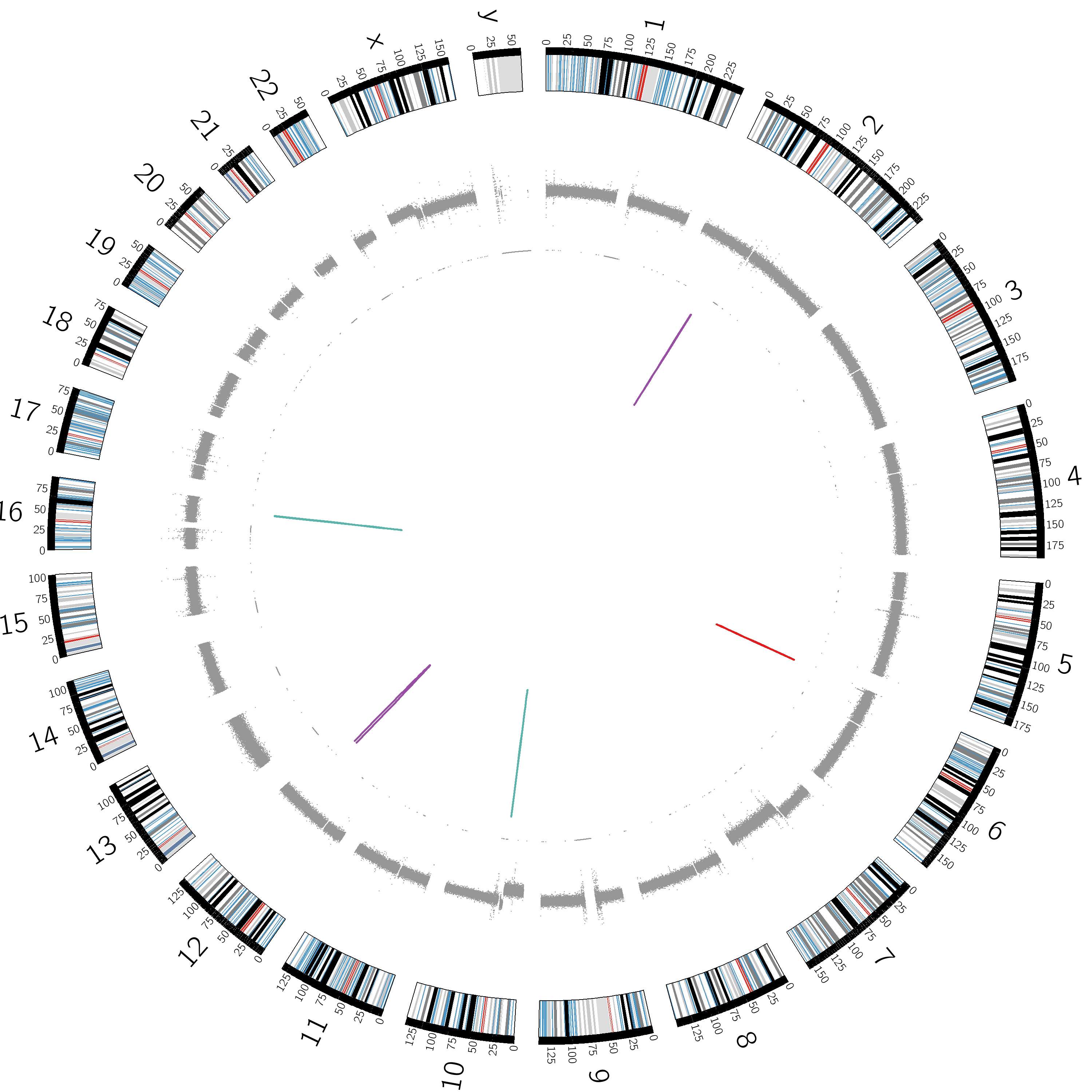

Supplement: Supplementary file 6 [file msb0011-0828-sd6.zip › png plots/BM769.png]

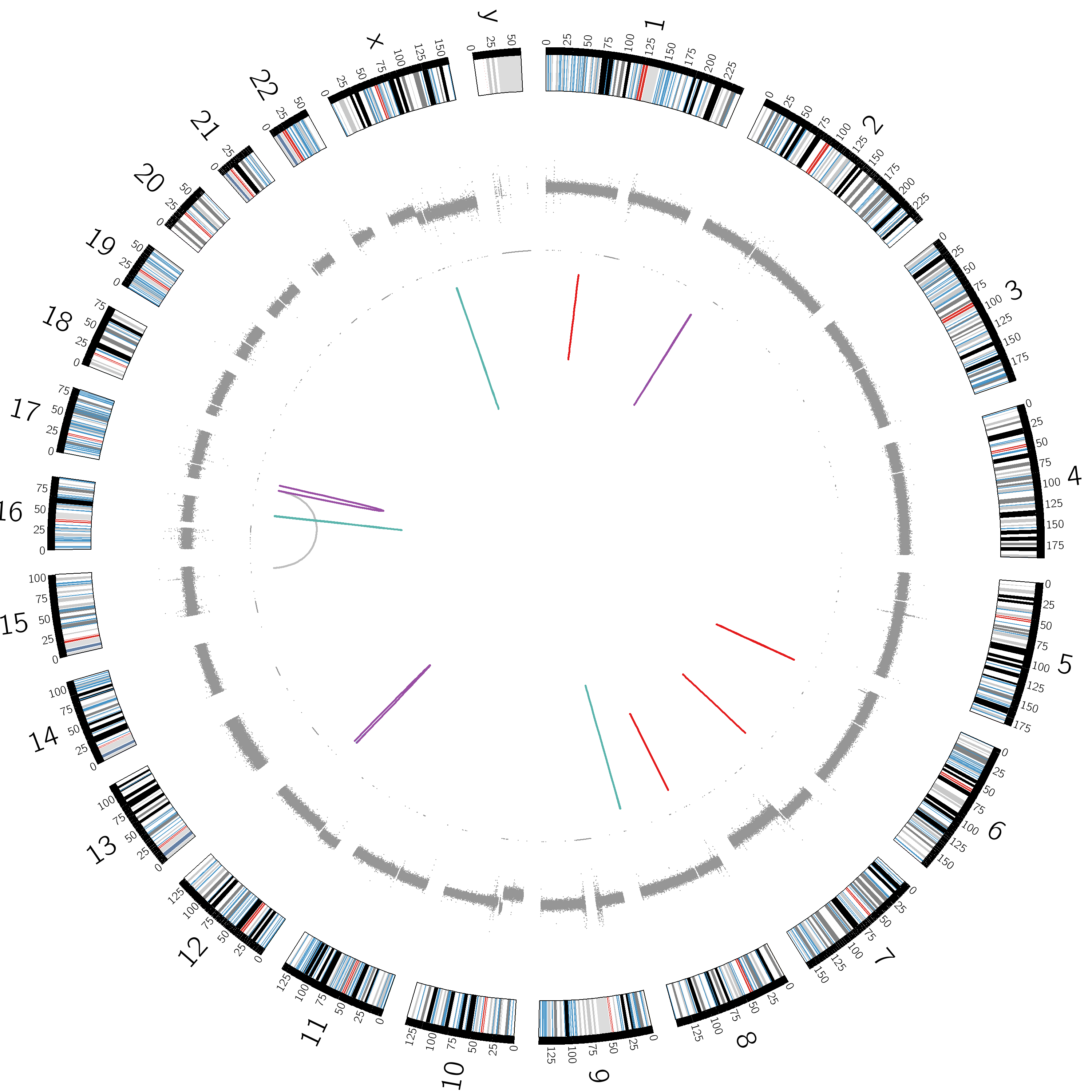

Supplement: Supplementary file 6 [file msb0011-0828-sd6.zip › png plots/BM770.png]

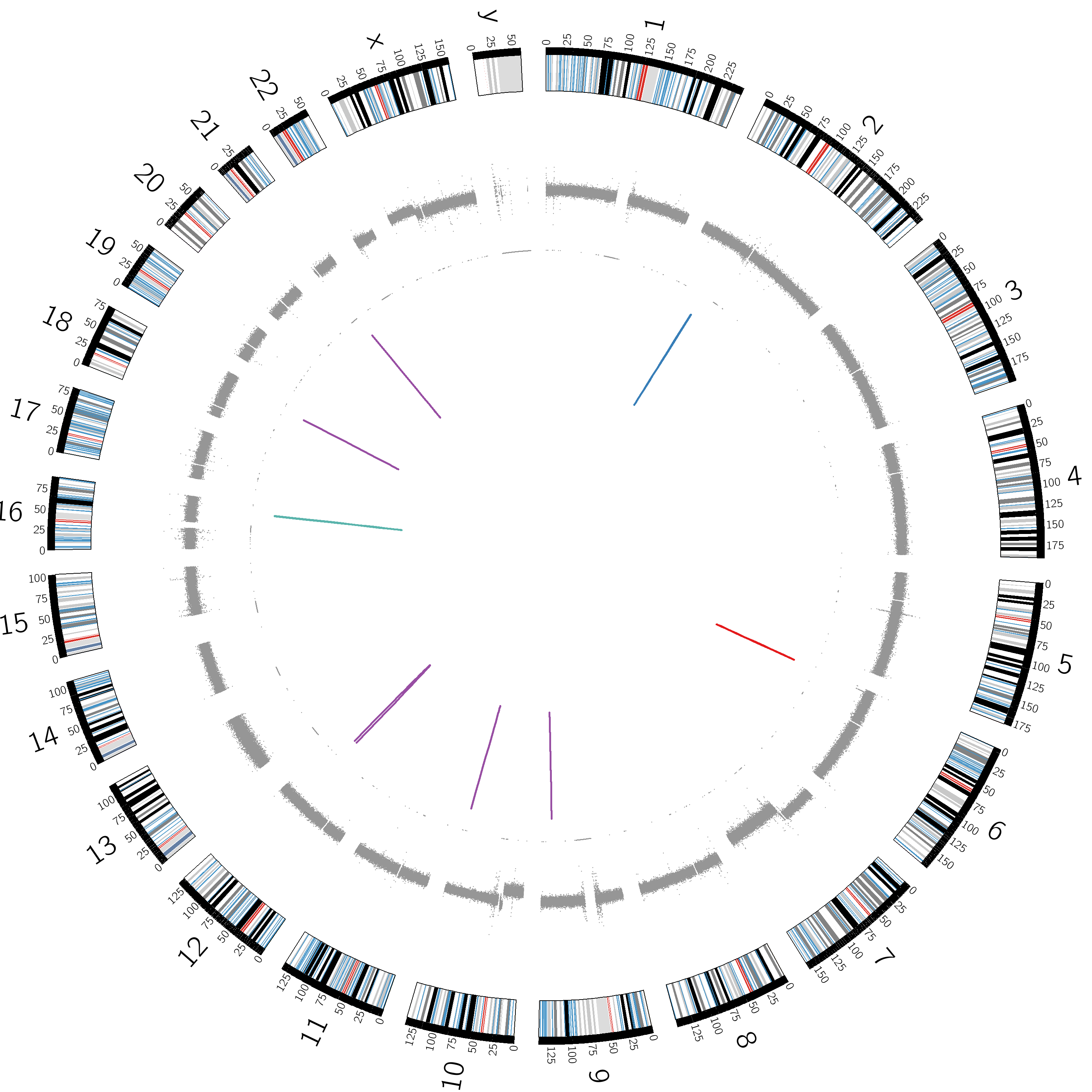

Supplement: Supplementary file 6 [file msb0011-0828-sd6.zip › png plots/BM771.png]

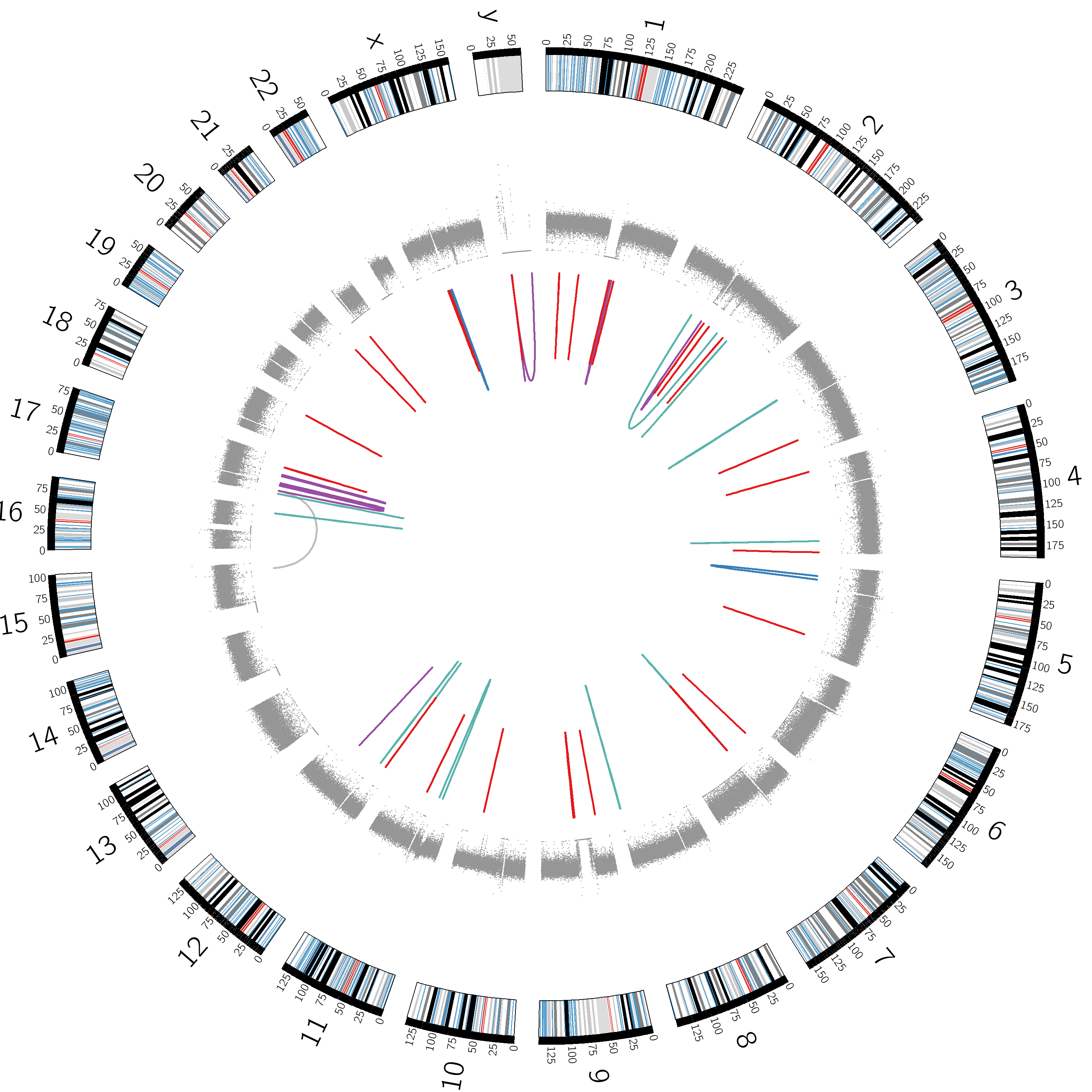

Supplement: Supplementary file 6 [file msb0011-0828-sd6.zip › png plots/BM772.png]

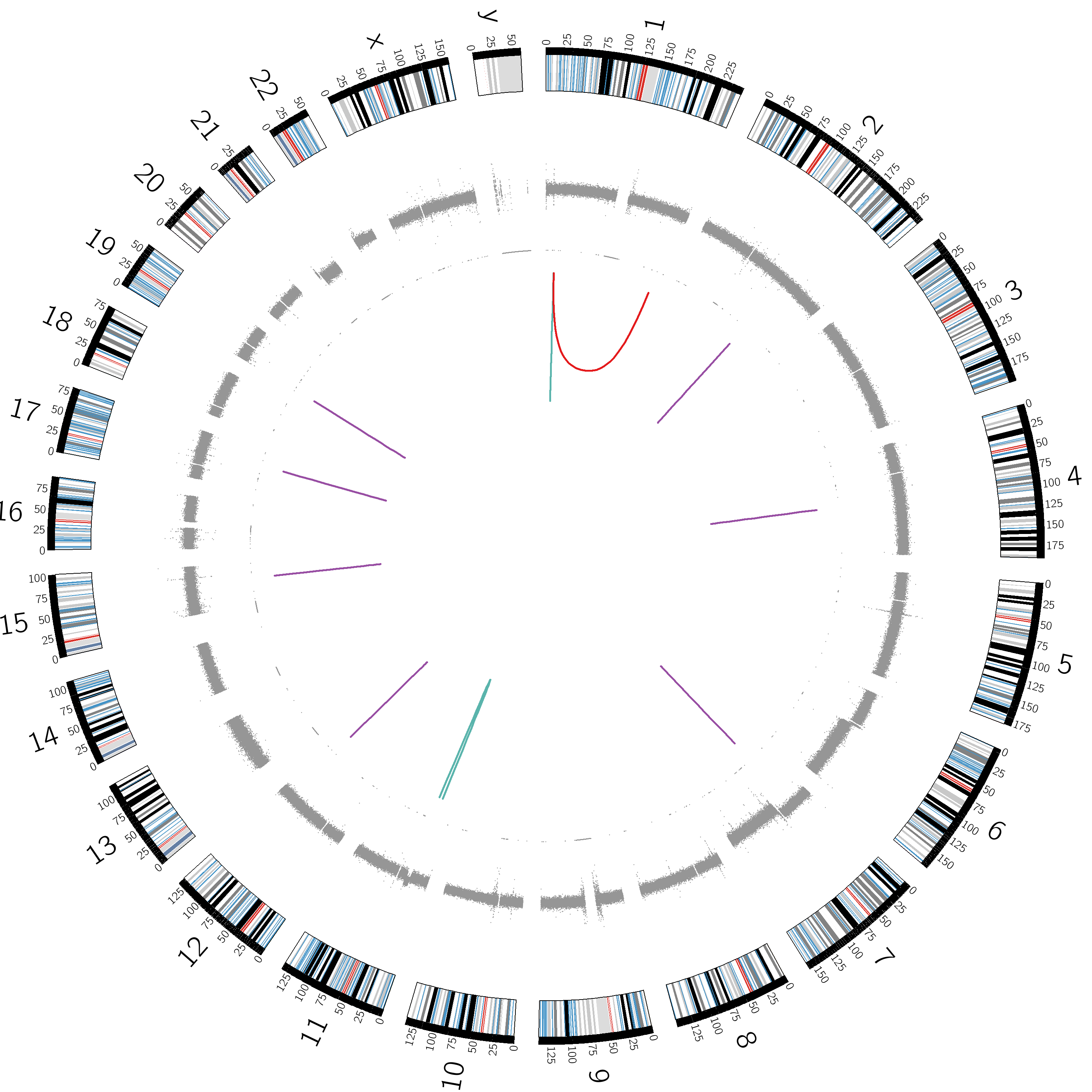

Supplement: Supplementary file 6 [file msb0011-0828-sd6.zip › png plots/BM773.png]

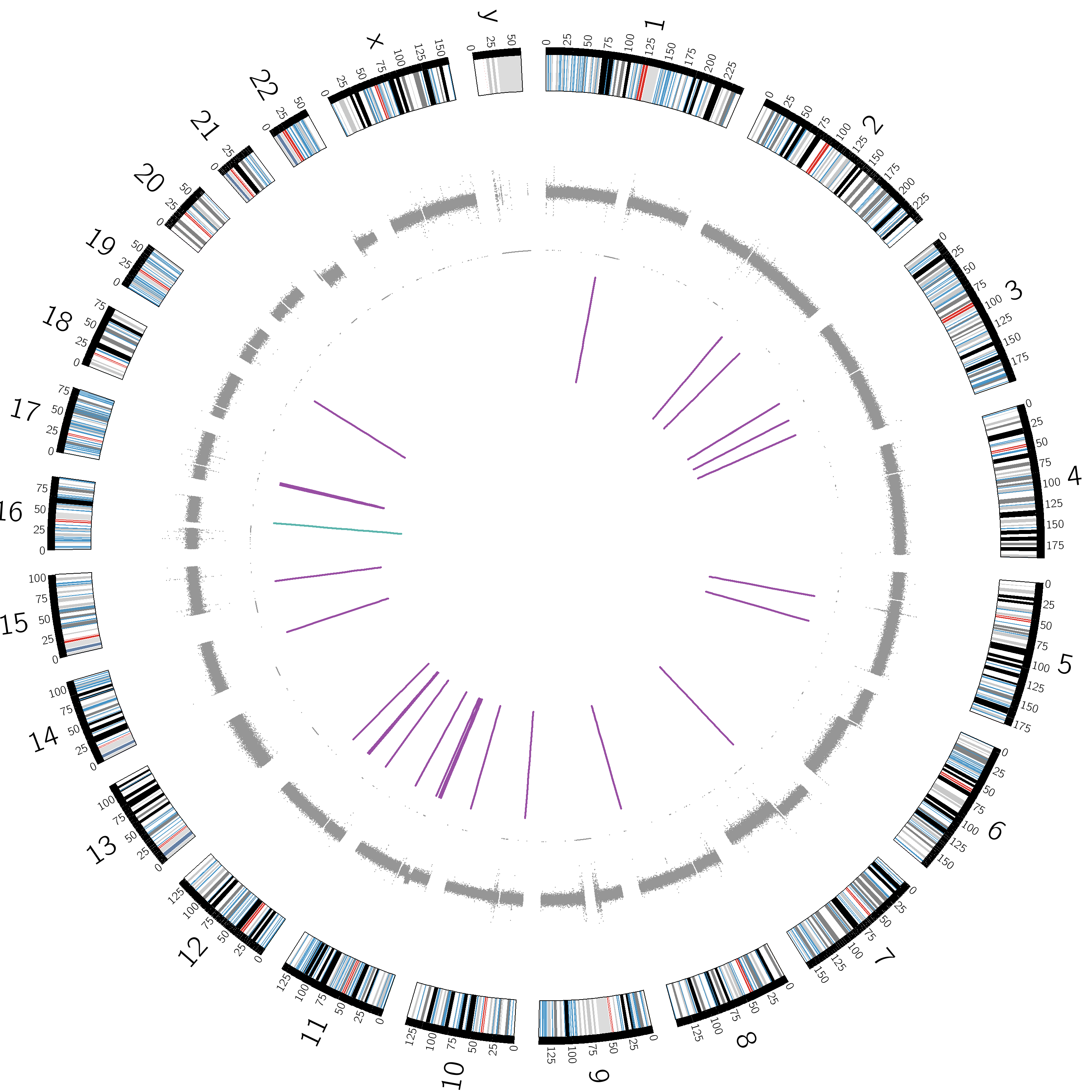

Supplement: Supplementary file 6 [file msb0011-0828-sd6.zip › png plots/BM774.png]

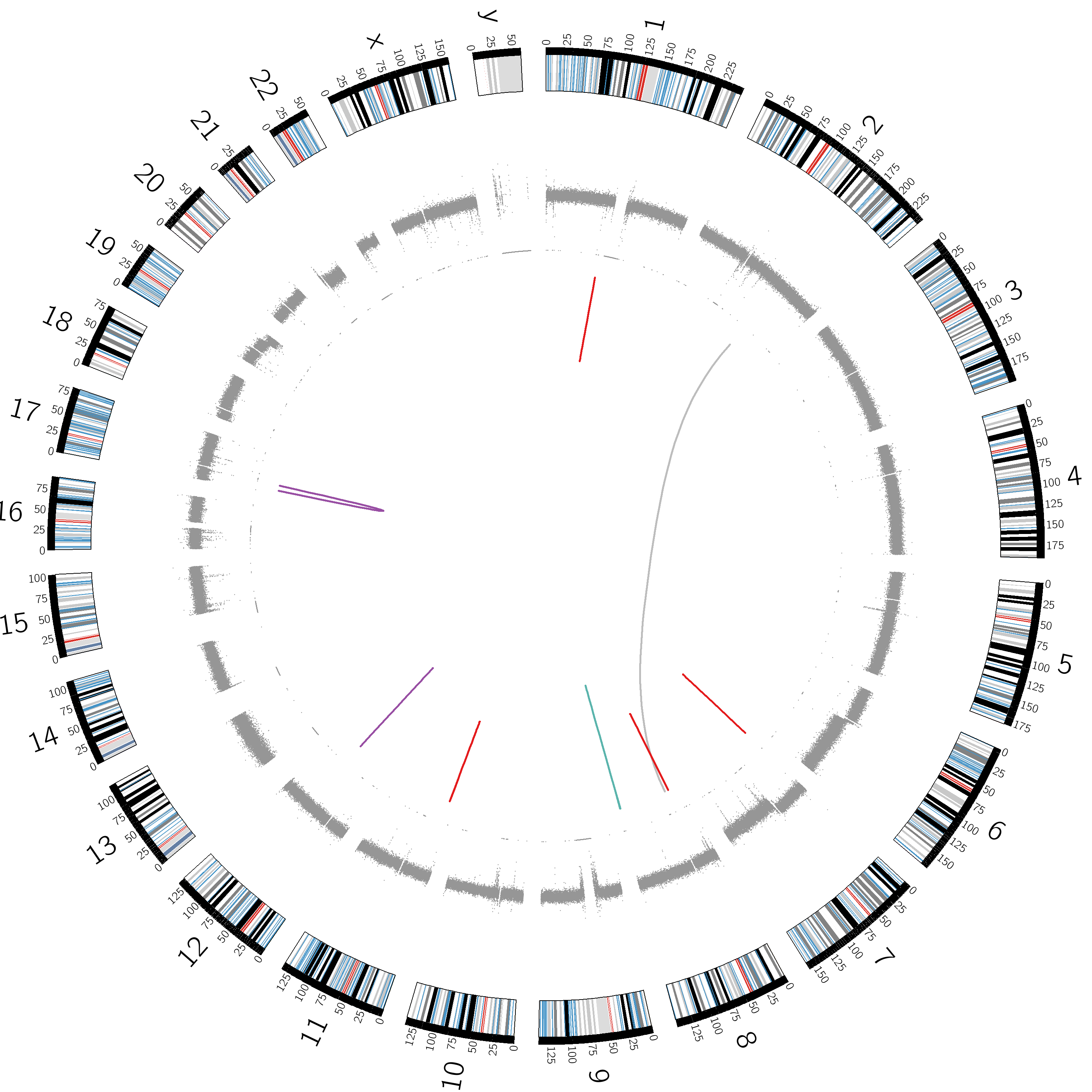

Supplement: Supplementary file 6 [file msb0011-0828-sd6.zip › png plots/BM775.png]

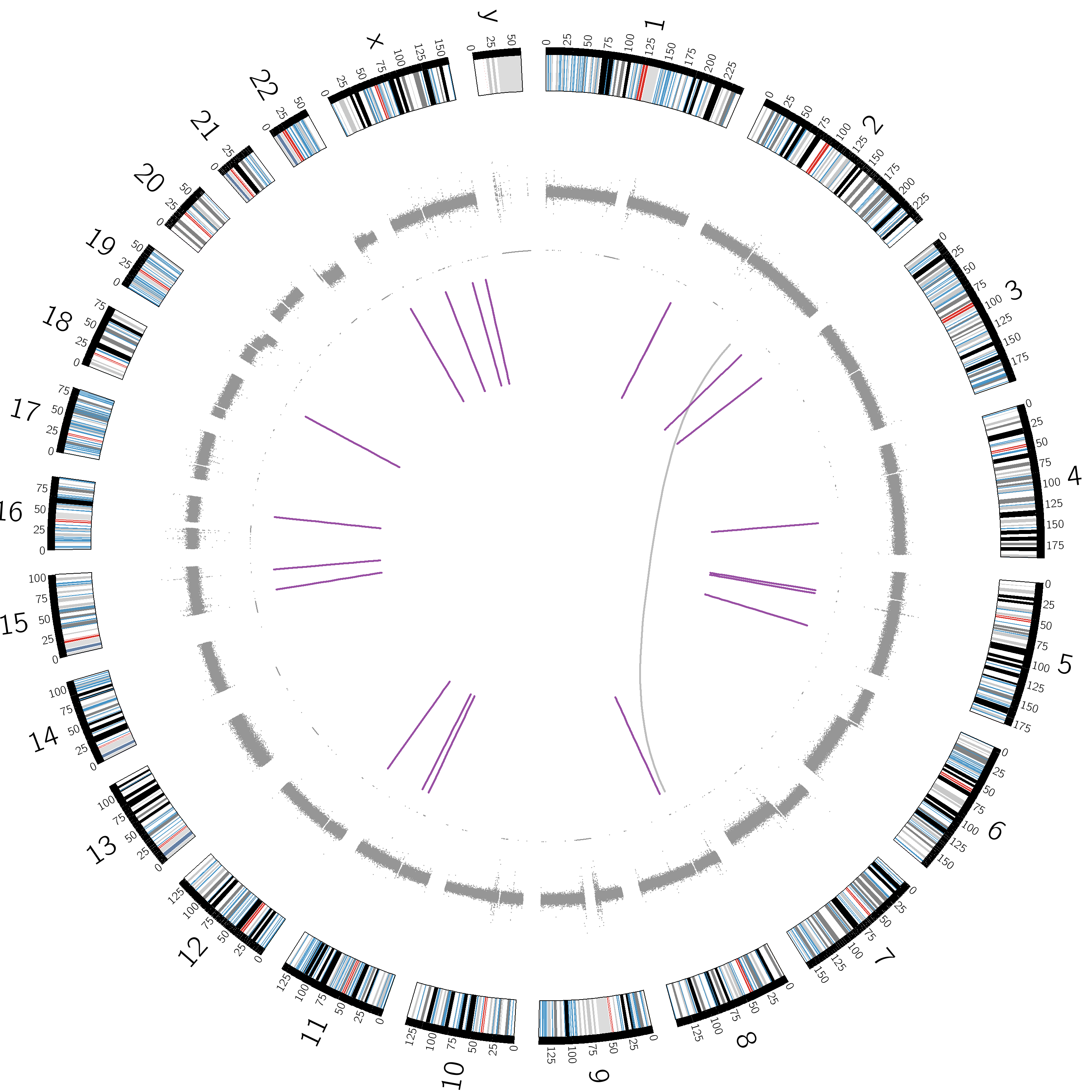

Supplement: Supplementary file 6 [file msb0011-0828-sd6.zip › png plots/BM776.png]

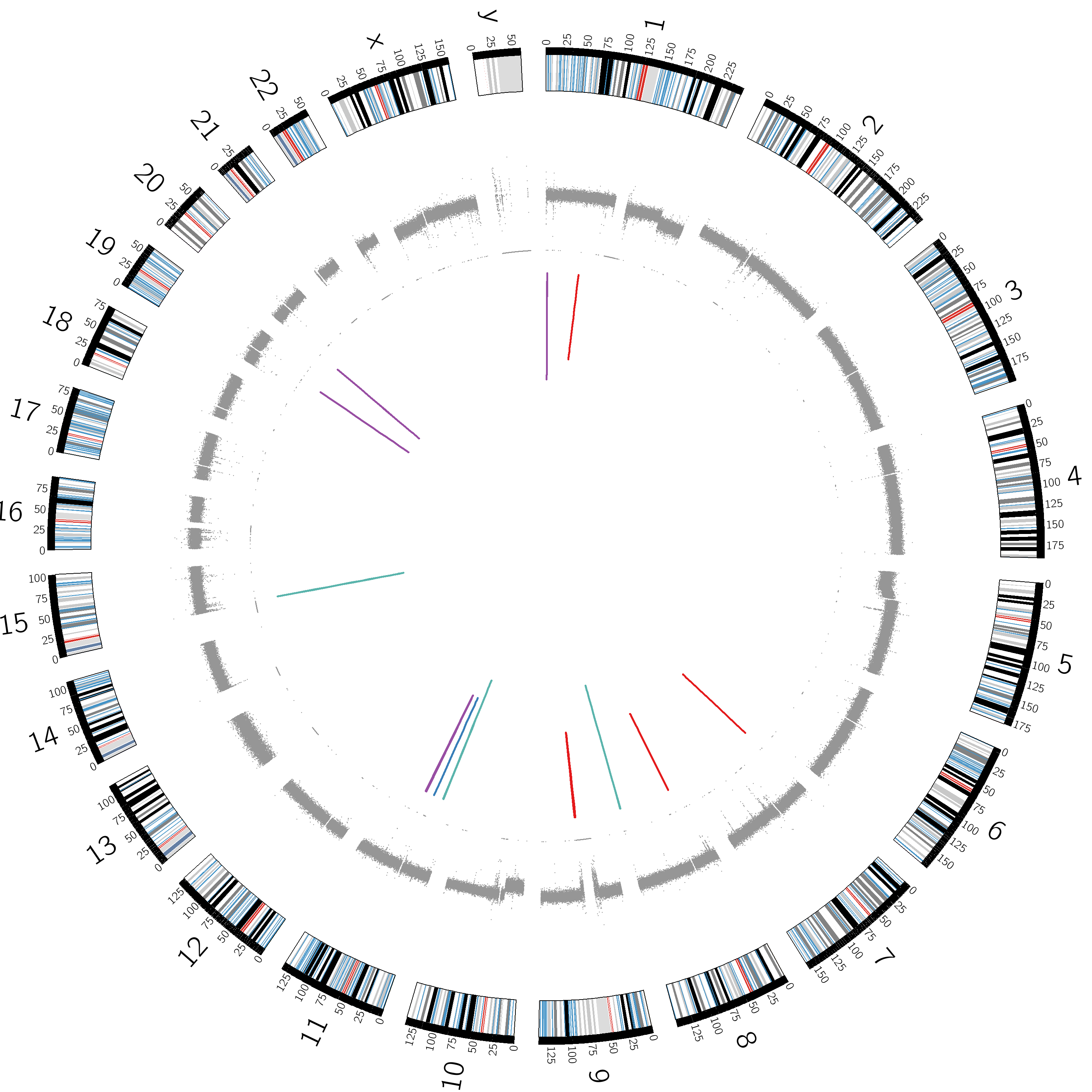

Supplement: Supplementary file 6 [file msb0011-0828-sd6.zip › png plots/BM777.png]

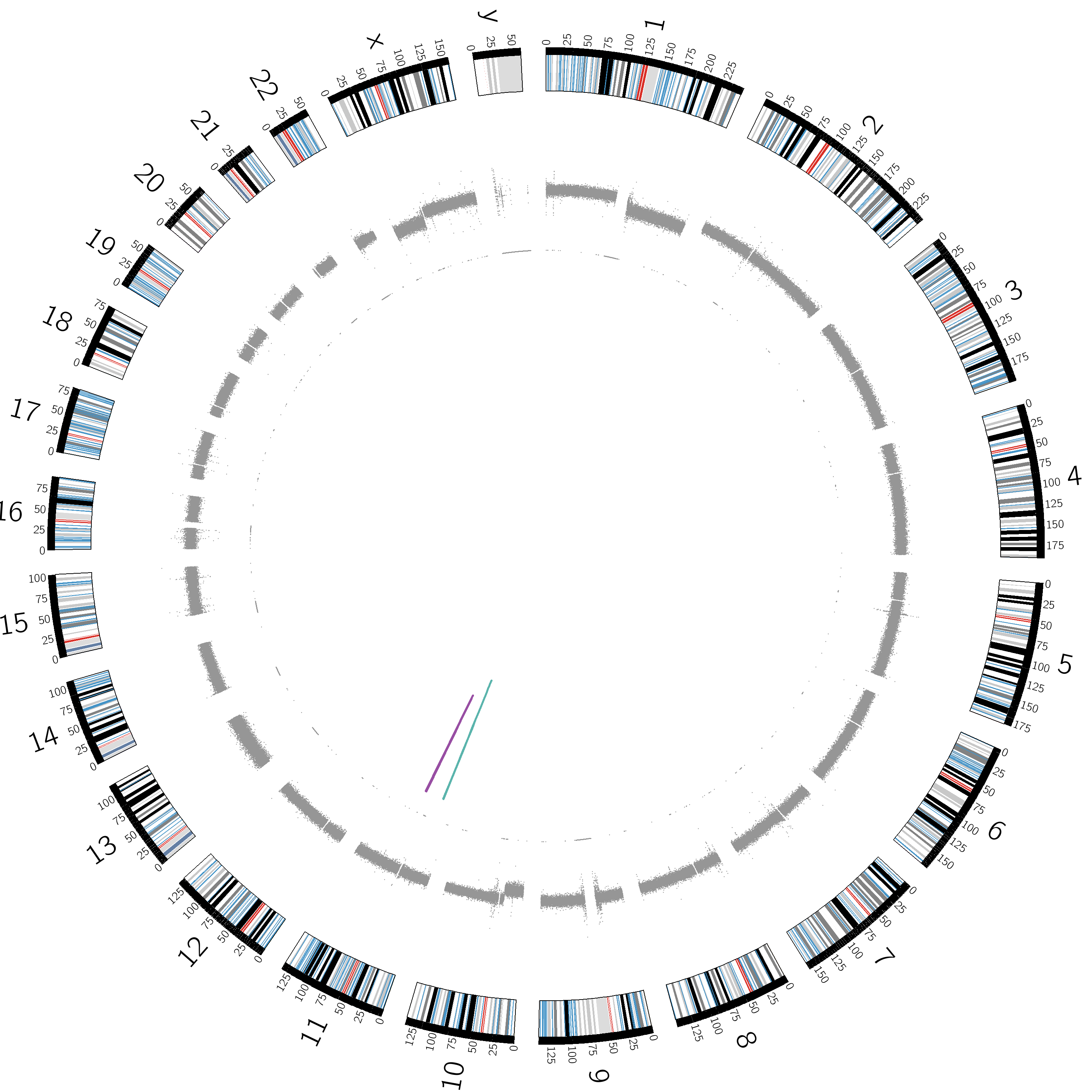

Supplement: Supplementary file 6 [file msb0011-0828-sd6.zip › png plots/BM779.png]

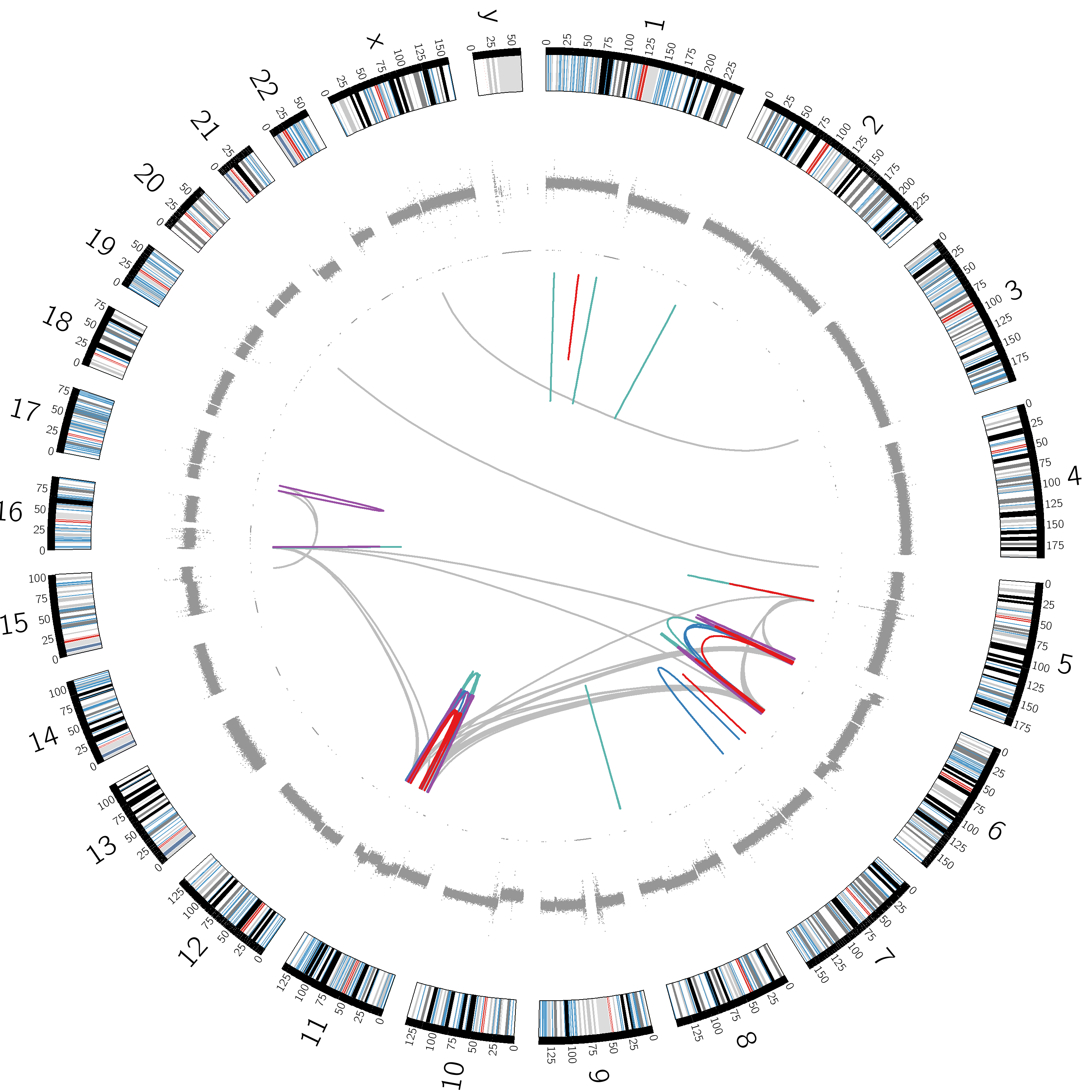

Supplement: Supplementary file 6 [file msb0011-0828-sd6.zip › png plots/BM780.png]

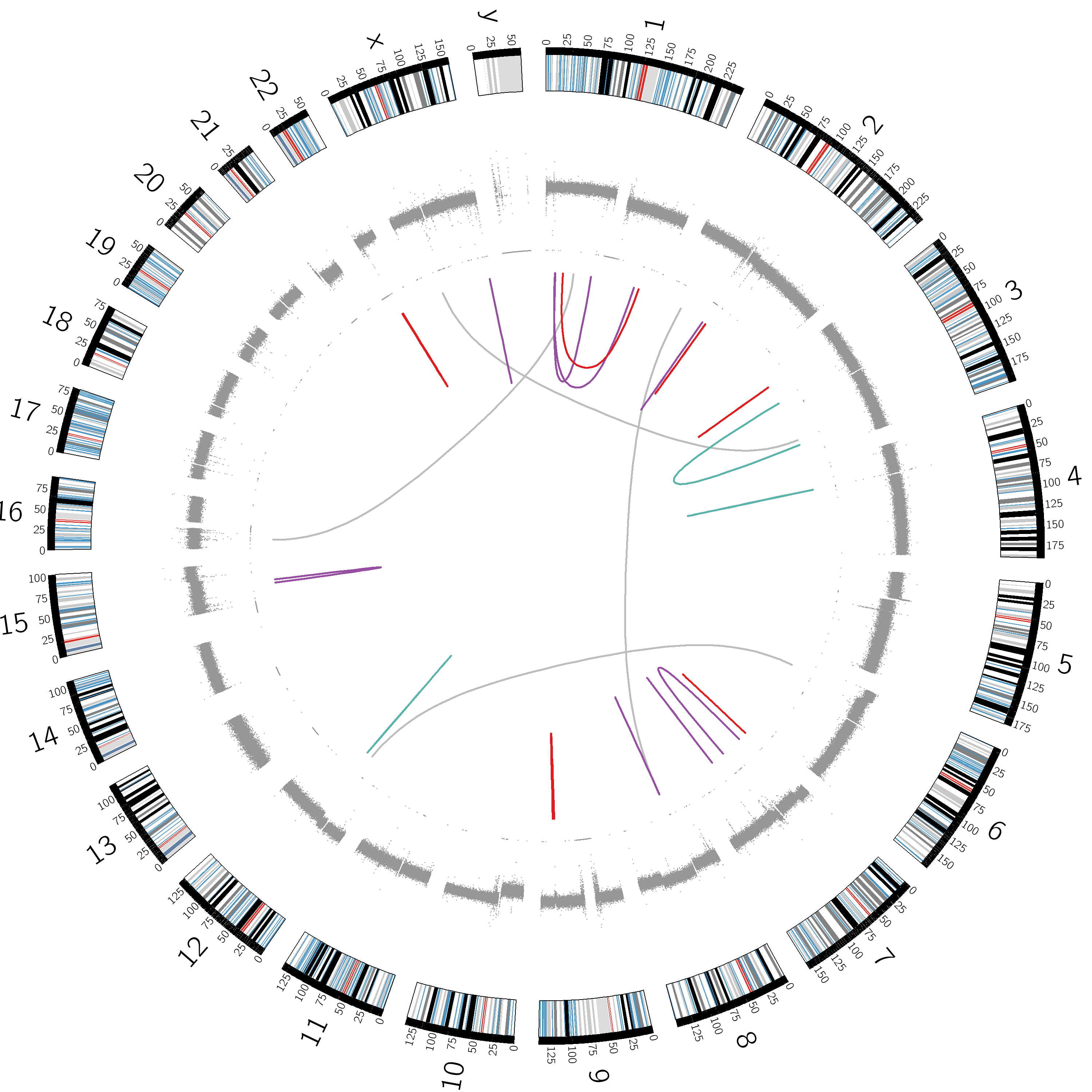

Supplement: Supplementary file 6 [file msb0011-0828-sd6.zip › png plots/BM782.png]

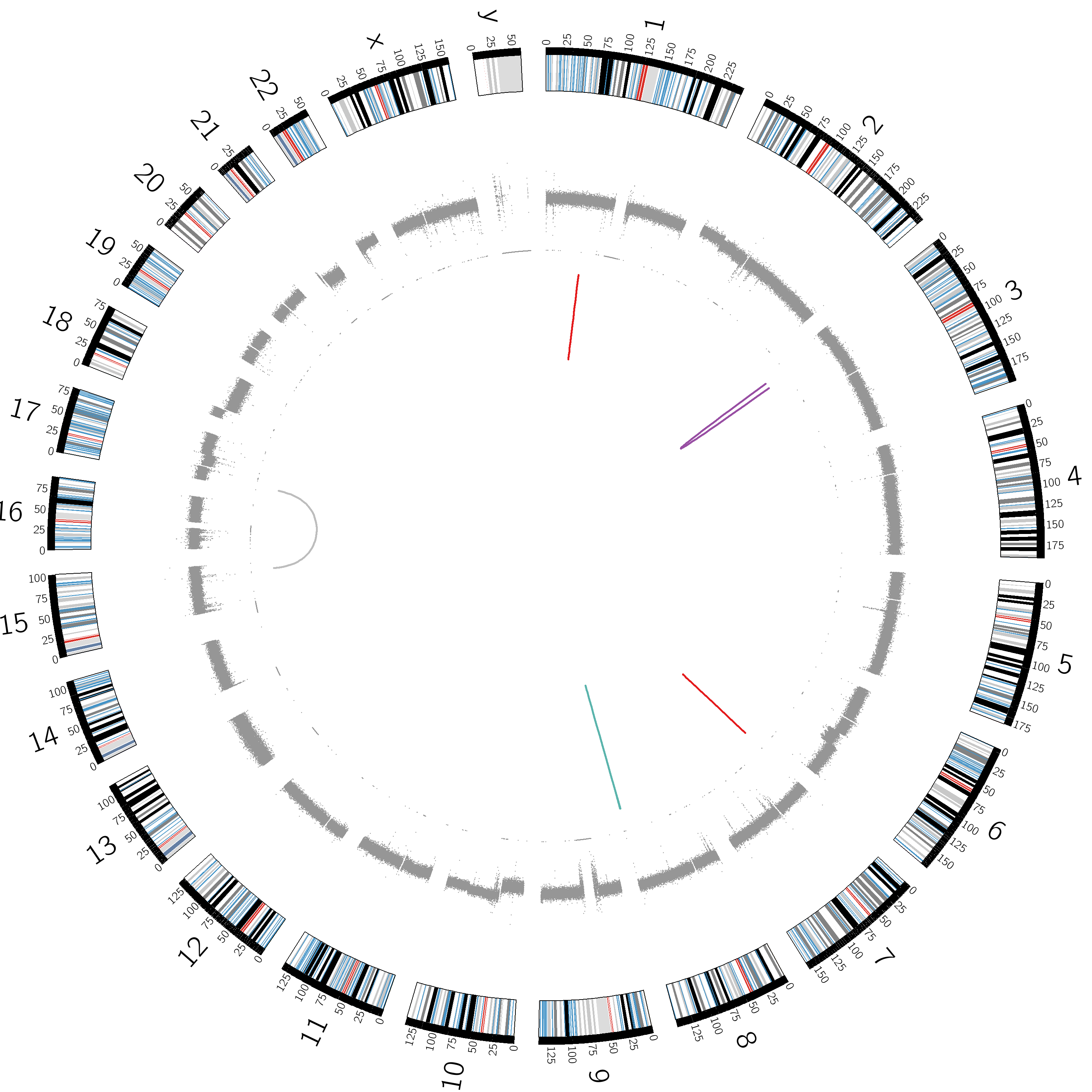

Supplement: Supplementary file 6 [file msb0011-0828-sd6.zip › png plots/BM783.png]

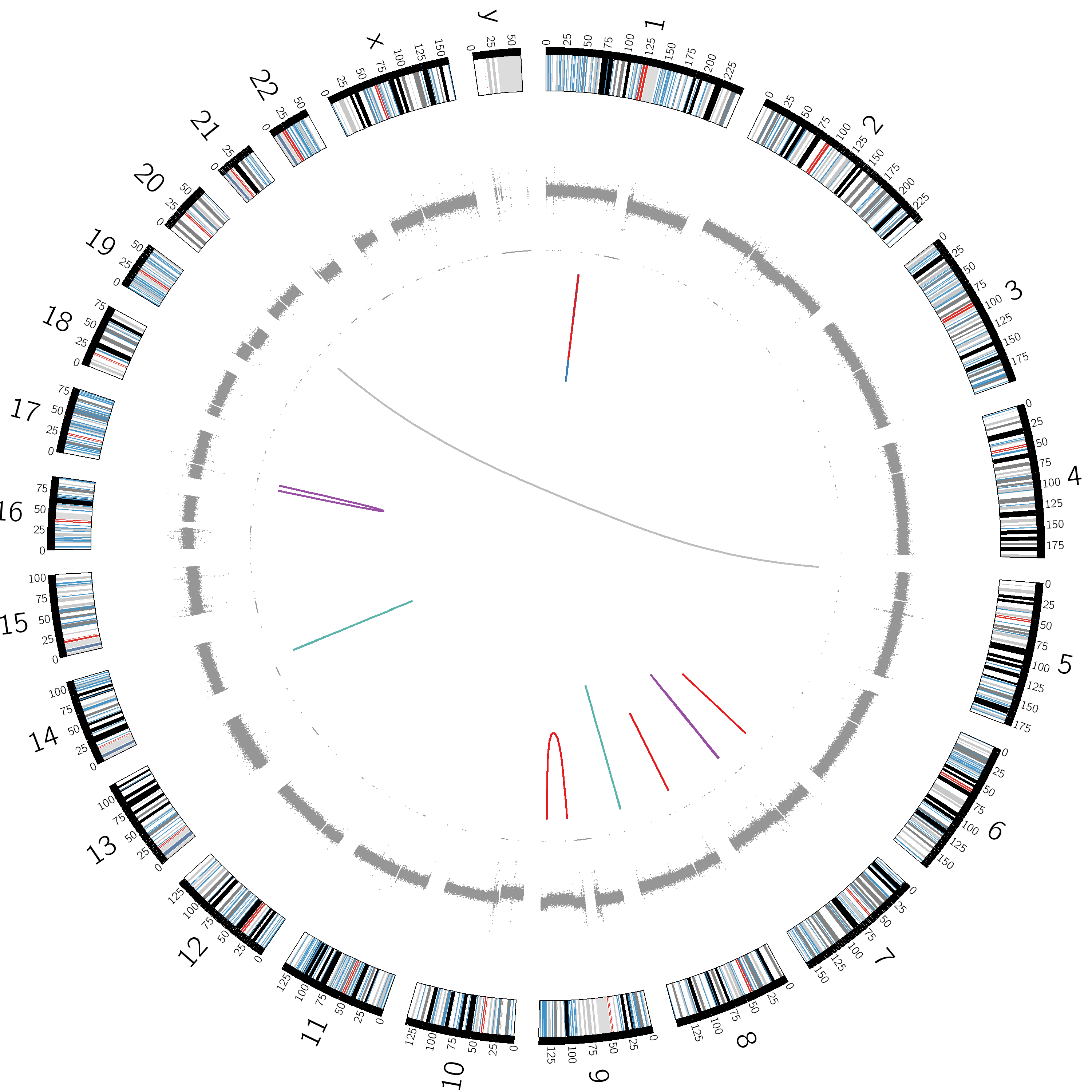

Supplement: Supplementary file 6 [file msb0011-0828-sd6.zip › png plots/BM786.png]

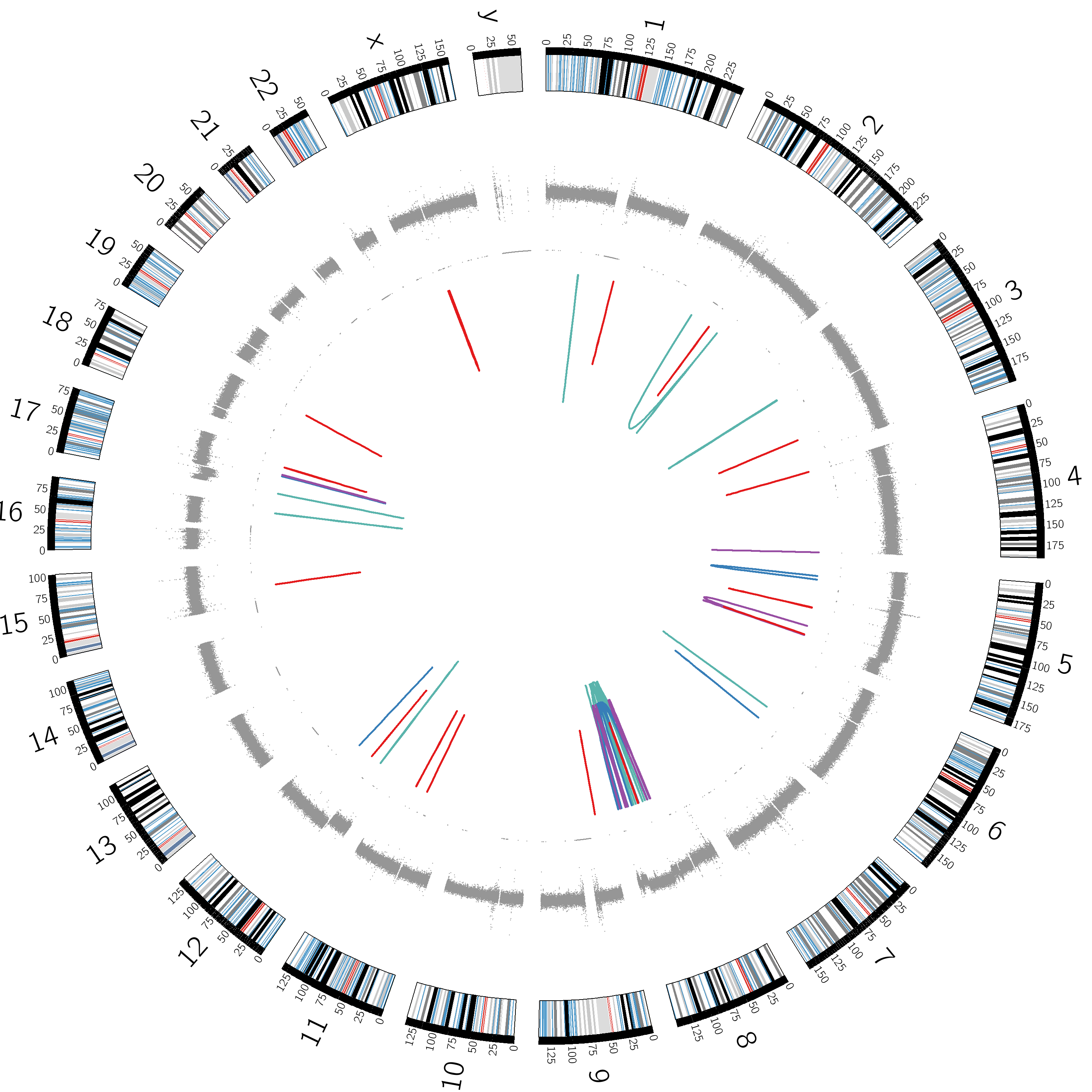

Supplement: Supplementary file 6 [file msb0011-0828-sd6.zip › png plots/BM838.png]

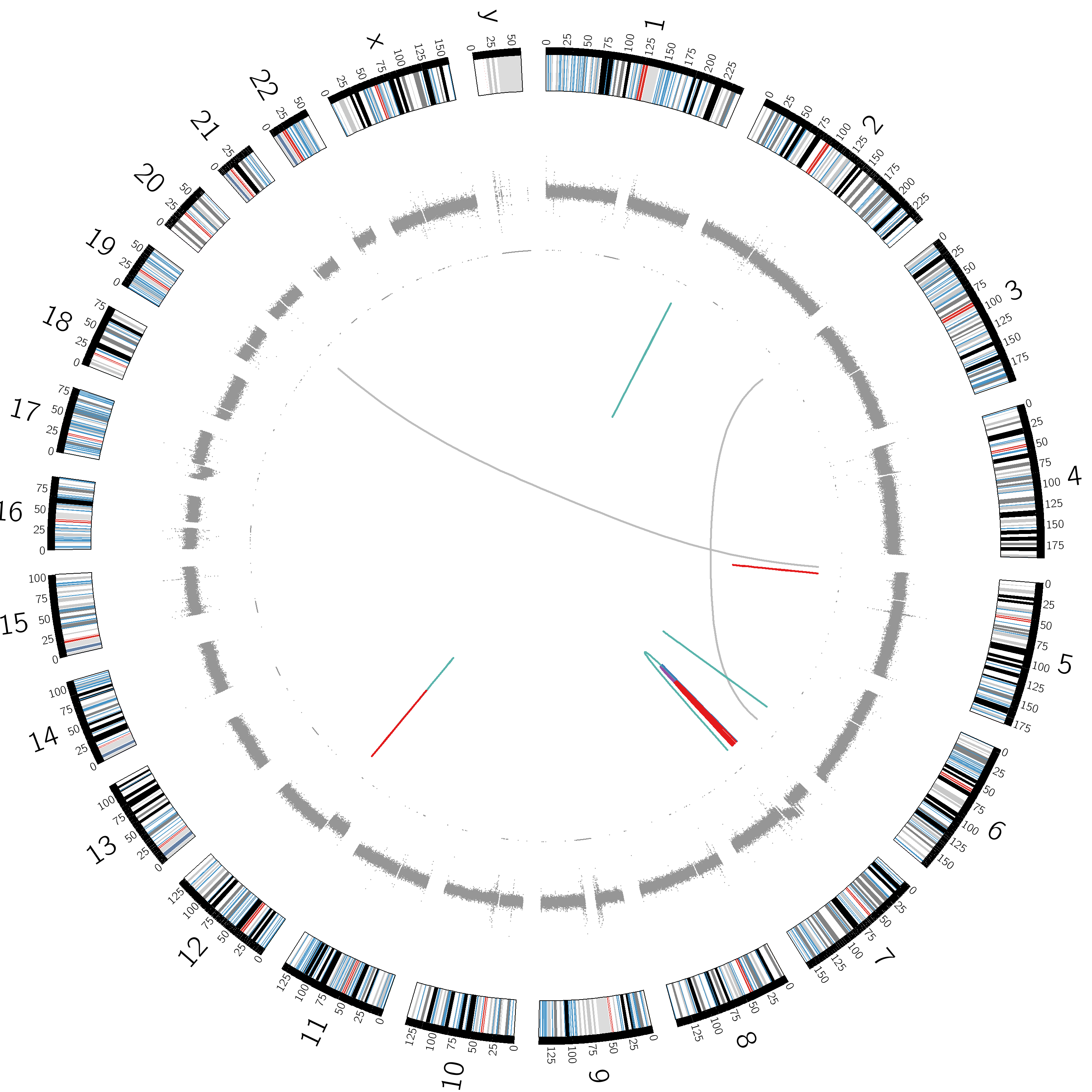

Supplement: Supplementary file 6 [file msb0011-0828-sd6.zip › png plots/BM844.png]

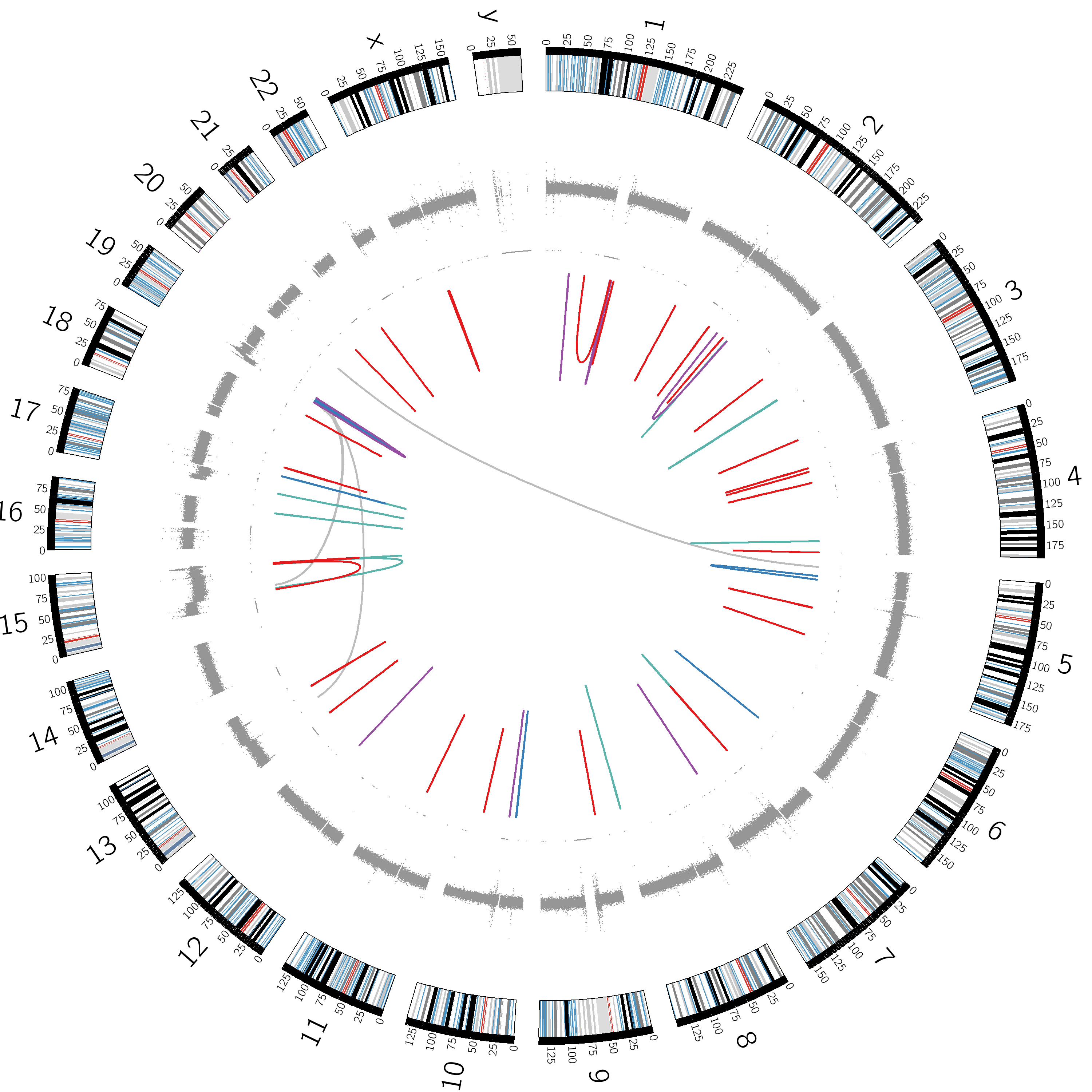

Supplement: Supplementary file 6 [file msb0011-0828-sd6.zip › png plots/BM943D.png]

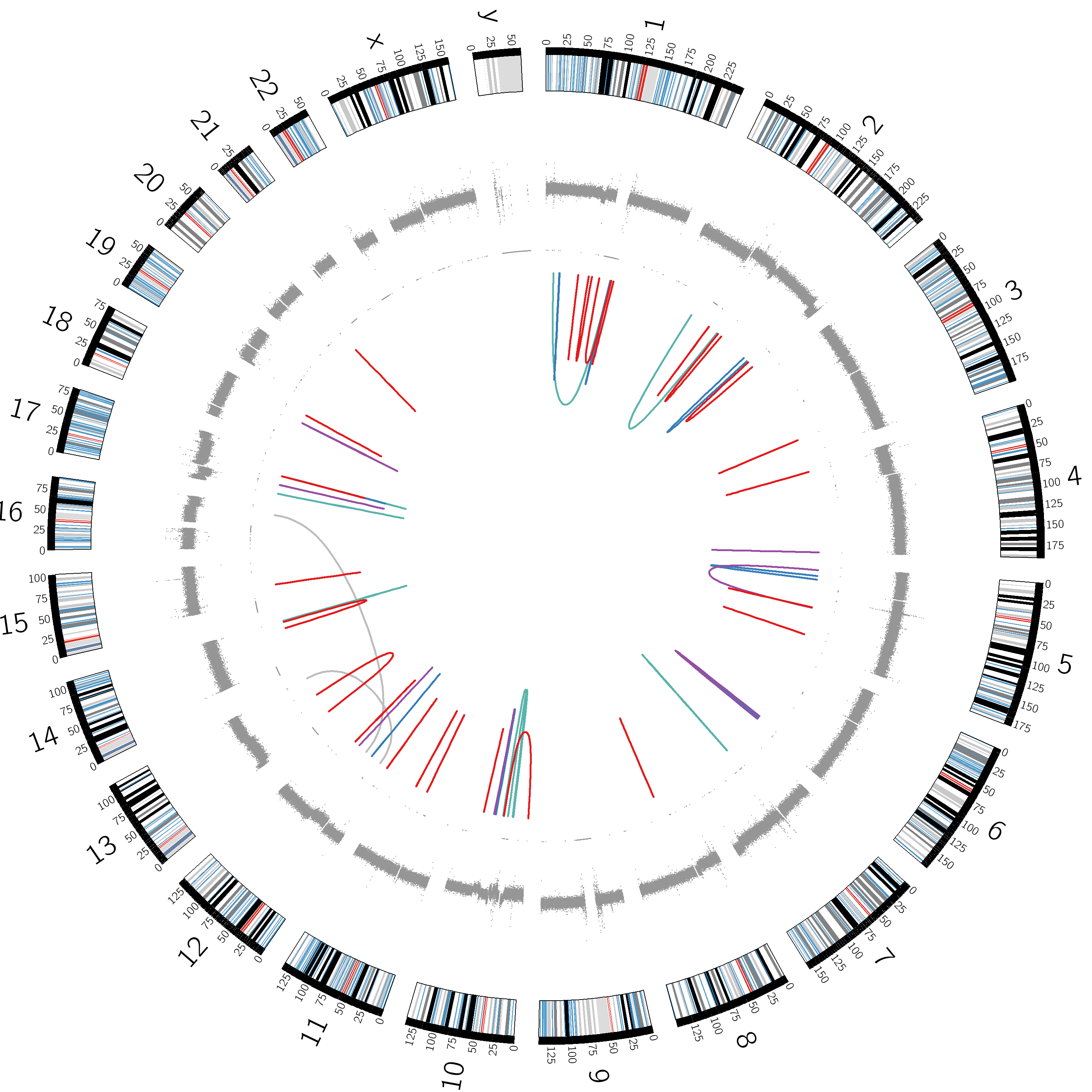

Supplement: Supplementary file 6 [file msb0011-0828-sd6.zip › png plots/BM948D.png]

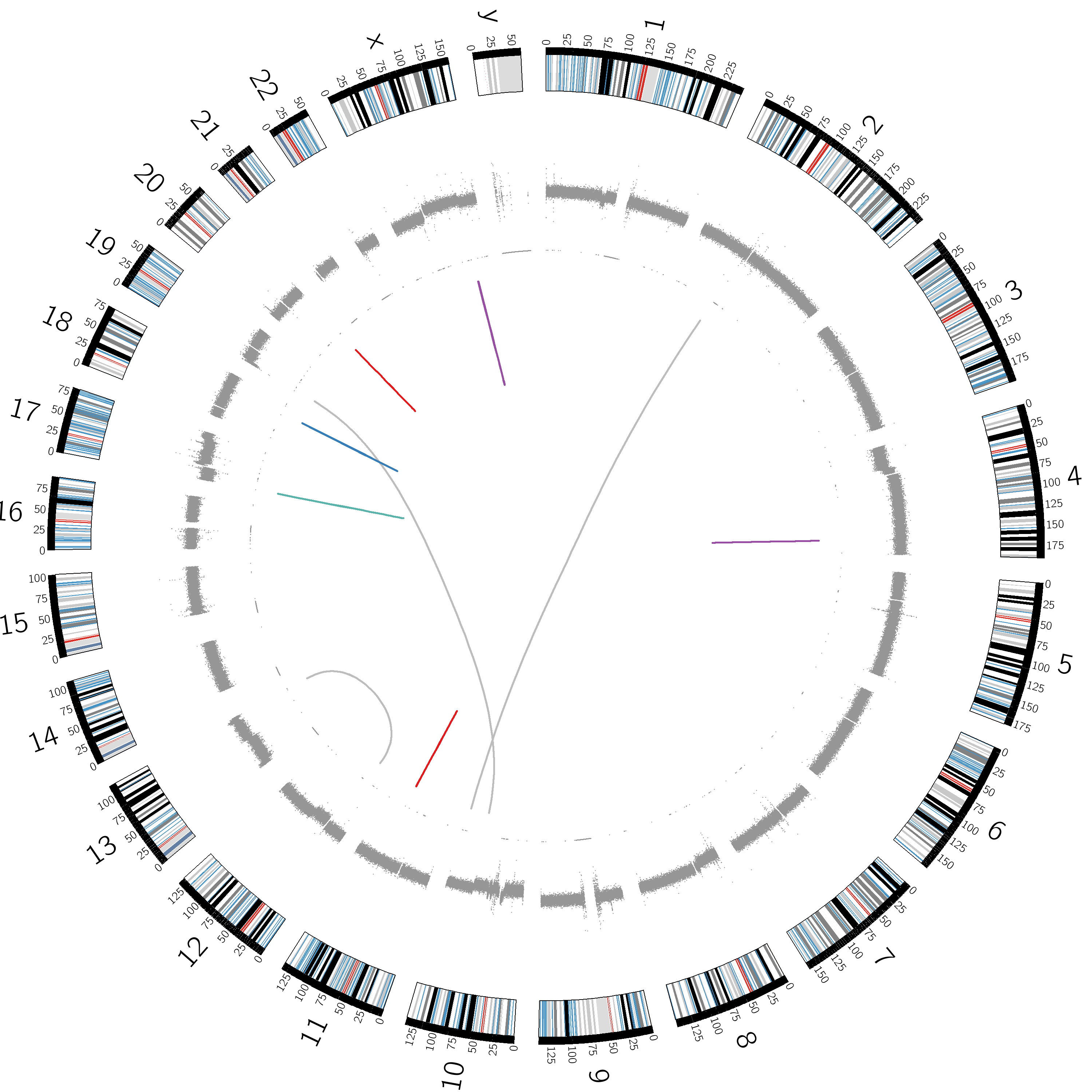

Supplement: Supplementary file 6 [file msb0011-0828-sd6.zip › png plots/BMC10.png]

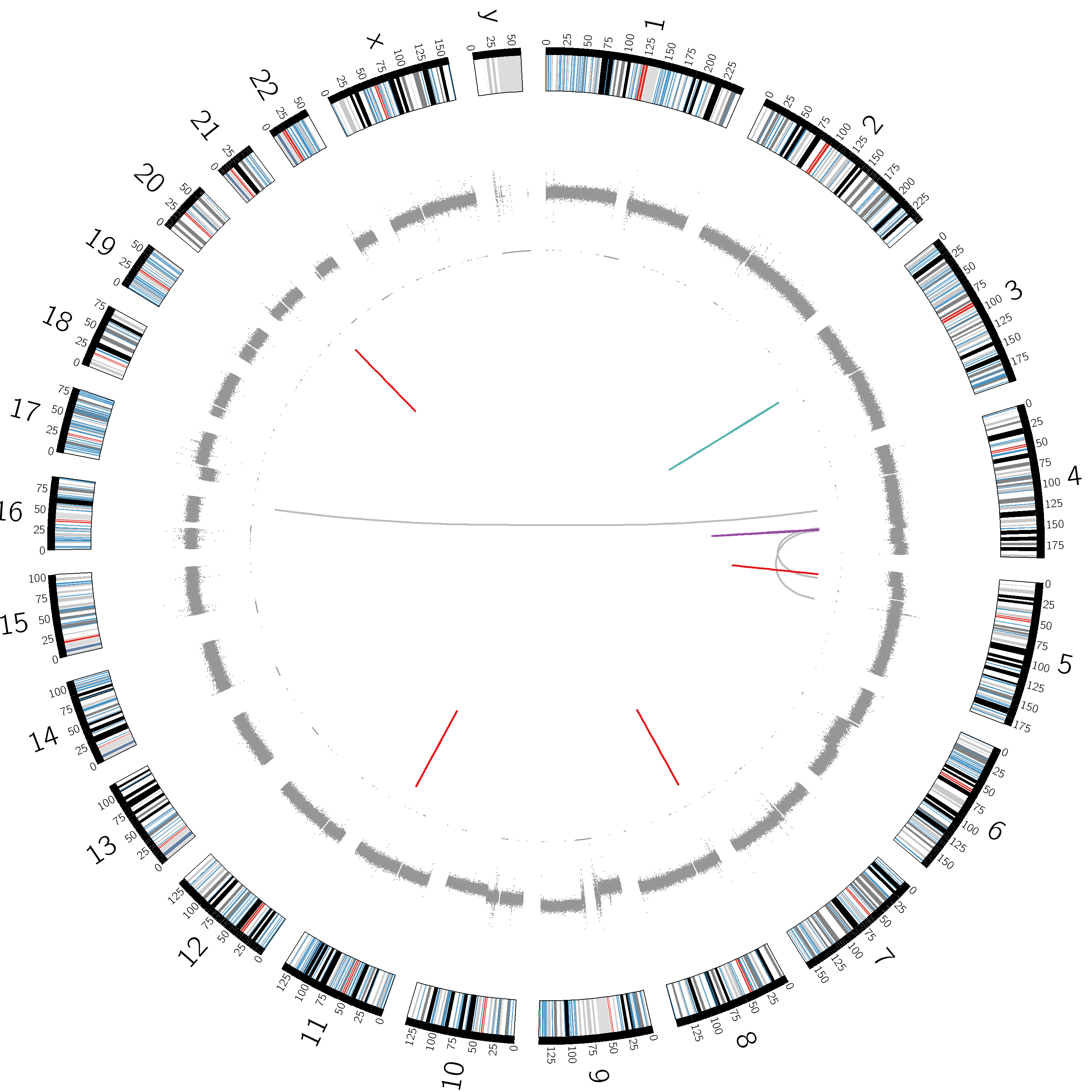

Supplement: Supplementary file 6 [file msb0011-0828-sd6.zip › png plots/BMC11.png]

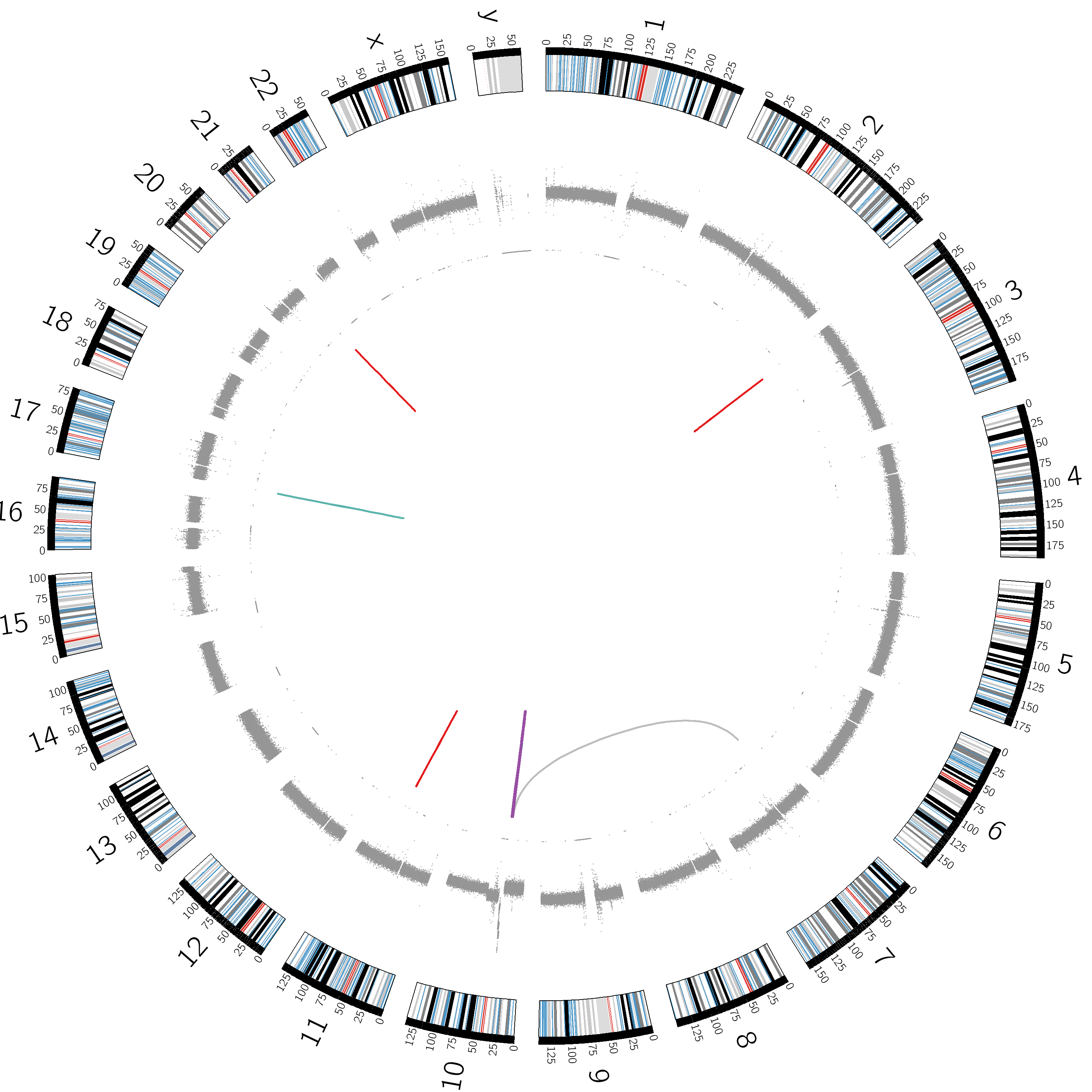

Supplement: Supplementary file 6 [file msb0011-0828-sd6.zip › png plots/BMC7.png]

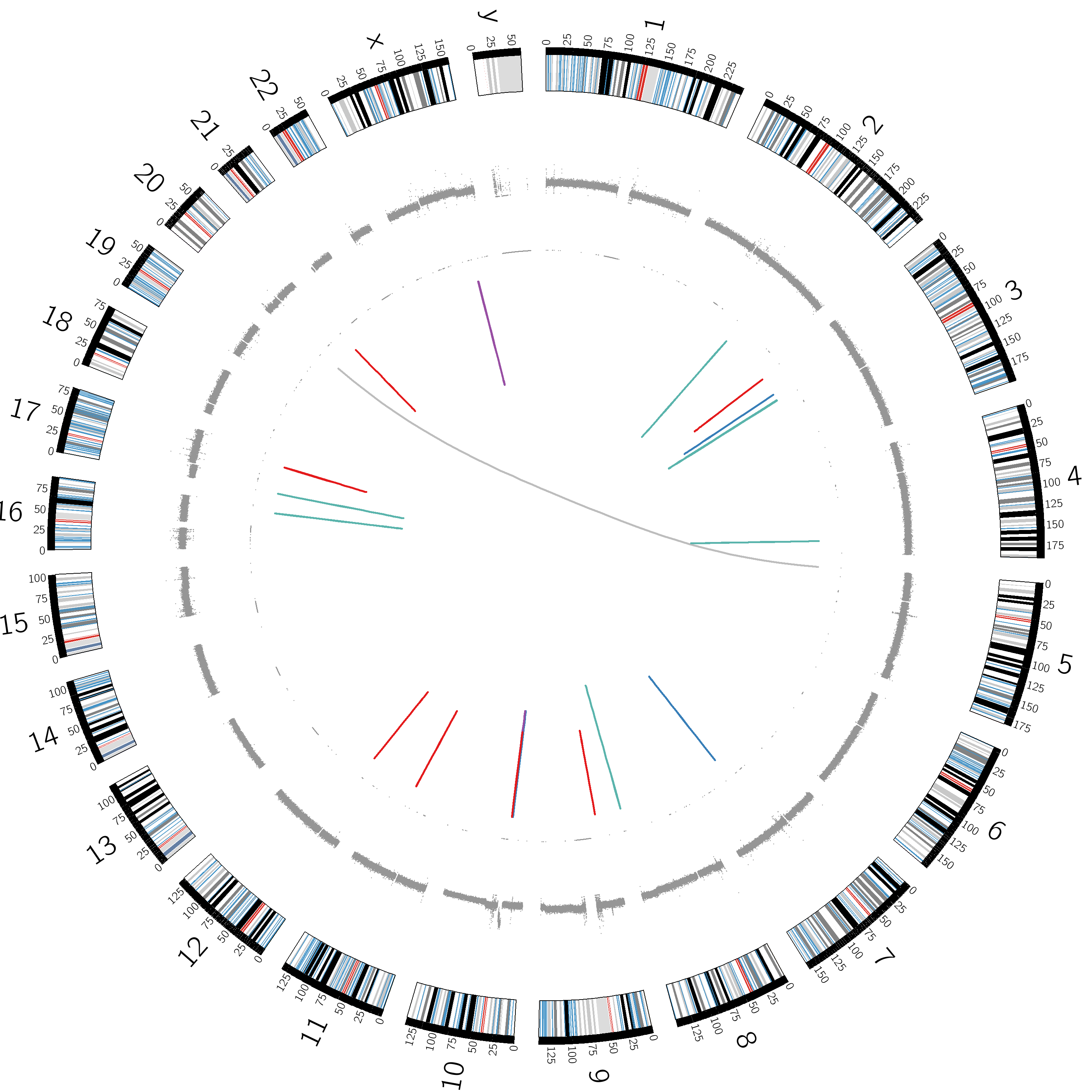

Supplement: Supplementary file 6 [file msb0011-0828-sd6.zip › png plots/BMC9.png]
