# Supplementary material for: A cell-based model system links chromothripsis with hyperploidy
Source: Mol Syst Biol. 2015 Sep 28;11(9):828. doi: 10.15252/msb.20156505 (PMC4592670; doi:10.15252/msb.20156505)
Supplement: Supplementary file 7 [file msb0011-0828-sd7.pdf]

A cell-based model system links chromothripsis with hyperploidy

Balca R. Mardin, Alexandros P. Drainas, Sebastian M. Waszak, Joachim Weischenfeldt, Mayumi Isokane, Adrian M. St¸tz, Benjamin Raeder, Theocharis Efthymiopoulos, Christopher Buccitelli, Maia Segura-Wang, Paul Northcott, Stefan M. Pfister, Peter Lichter, Dr. Jan Ellenberg and Jan O Korbel

Corresponding author: Dr. Jan Korbel, EMBL

Review timeline:

Submission date:	13 August 2015
Editorial Decision:	18 August 2015
Revision received:	24 August 2015
Accepted:	28 August 2015

Editor: Maria Polychronidou

Please note that the manuscript was previously reviewed at another journal and the reports were taken into account in the decision making process at MSB. Since the original reviews are not subject to MSB's transparent review process policy, the reports and author response cannot be published

© European Molecular Biology Organization
